# Supplementary material for: A Pilot Longitudinal Clinical Reasoning Curriculum for Pediatric Residents
Source: MedEdPORTAL. 2024 Sep 25;20:11447. doi: 10.15766/mep_2374-8265.11447 (PMC11422513; doi:10.15766/mep_2374-8265.11447)
Supplement: Supplementary file 1 — Preimplementation Survey.docxCurriculum Goals, Objectives, and Timeline.docxSession 1 - Illness Scripts.pptxSession 1 - Small-Group Facilitator Guide.docxSession 2 - Illness Scripts 2.pptxSession 2 - Small-Group Facilitator Guide.docxSession 3 - Script Concordance.pptxSession 3 - Small-Group Facilitator Guide.docxSession 3 - Small-Group Handout.docxSession 4 - Pathophysiology.pptxSession 4 - Small-Group Facilitator Guide.docxSession 4 - Small-Group Handout.docxSession 5 - Review Game.pptxPostimplementation Survey.docx [file mep_2374-8265.11447-s001.zip › J. Session 4 - Pathophysiology.pptx]

## Slide 1
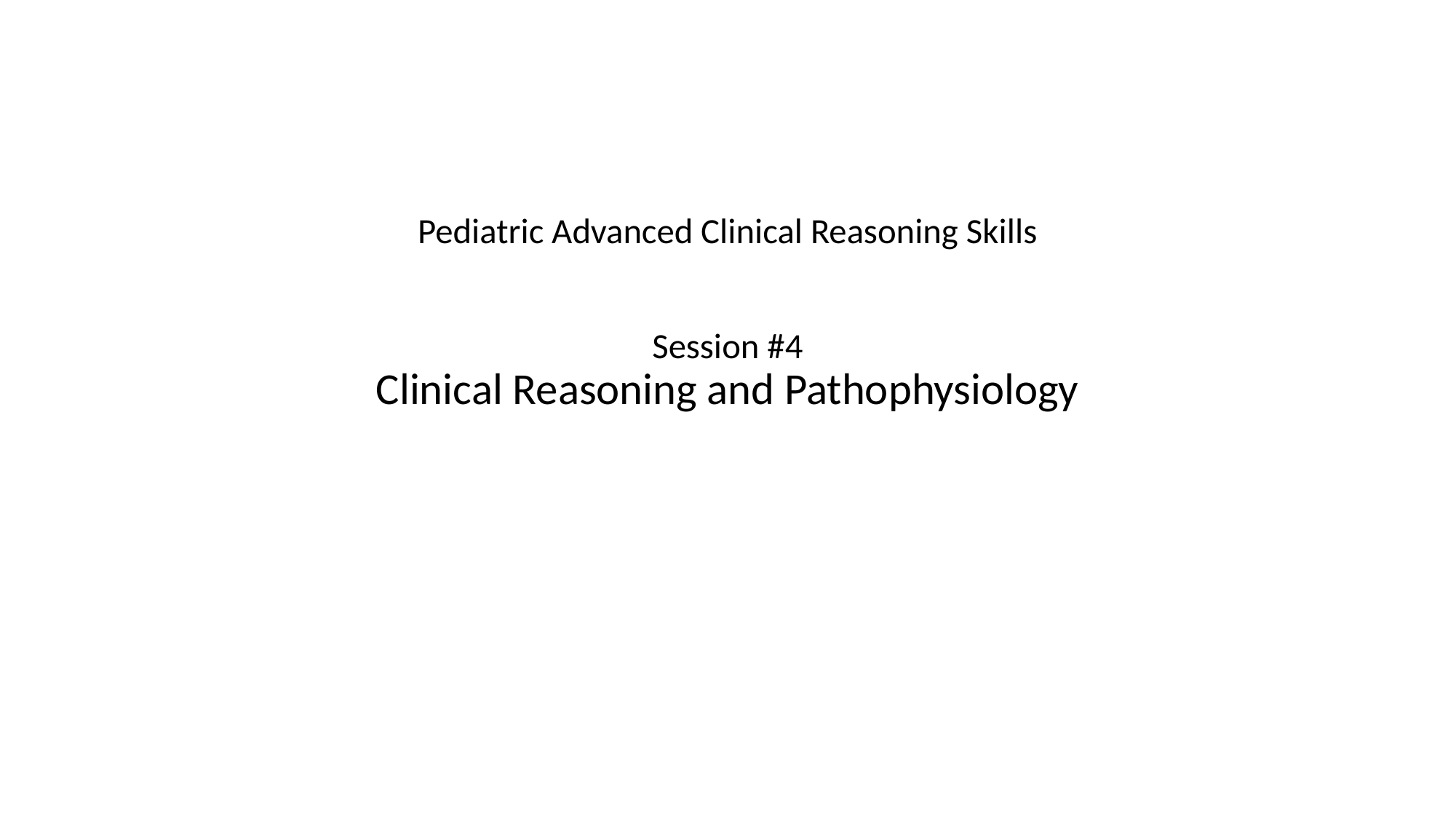

# Pediatric Advanced Clinical Reasoning Skills Session #4 Clinical Reasoning and Pathophysiology

## Slide 2
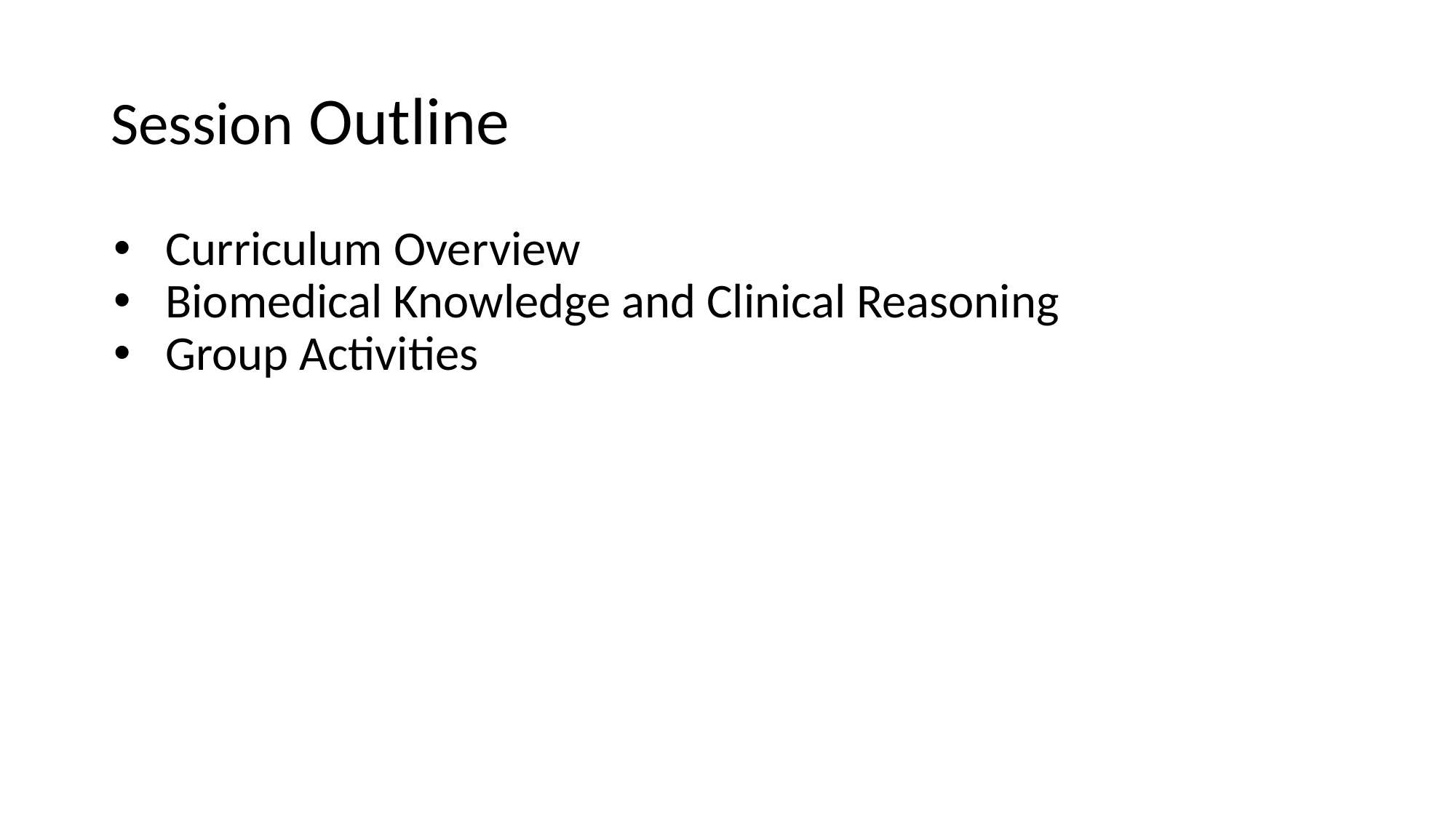

# Session Outline
Curriculum Overview
Biomedical Knowledge and Clinical Reasoning
Group Activities

## Slide 3
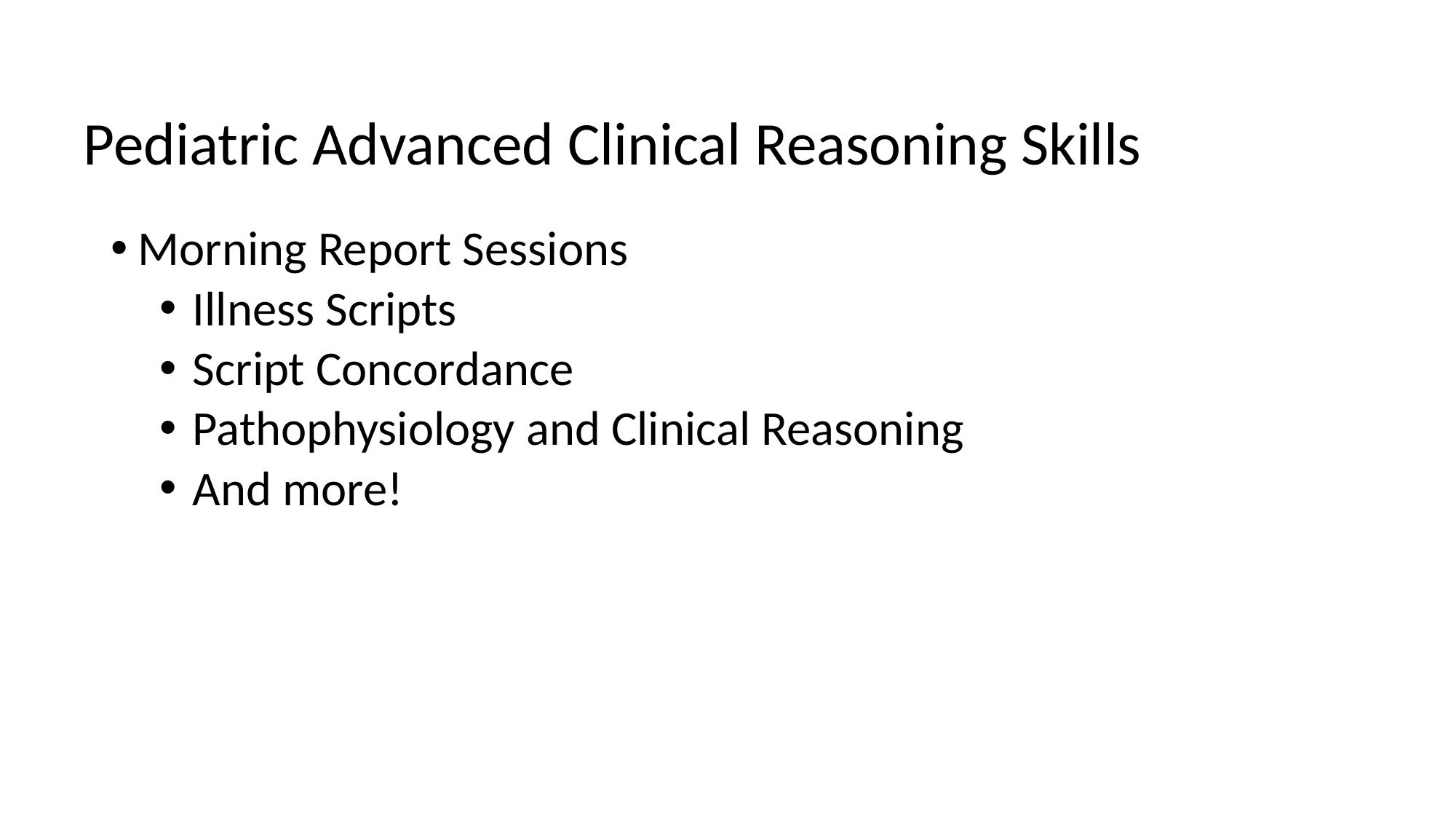

# Pediatric Advanced Clinical Reasoning Skills
Morning Report Sessions
Illness Scripts
Script Concordance
Pathophysiology and Clinical Reasoning
And more!

## Slide 4
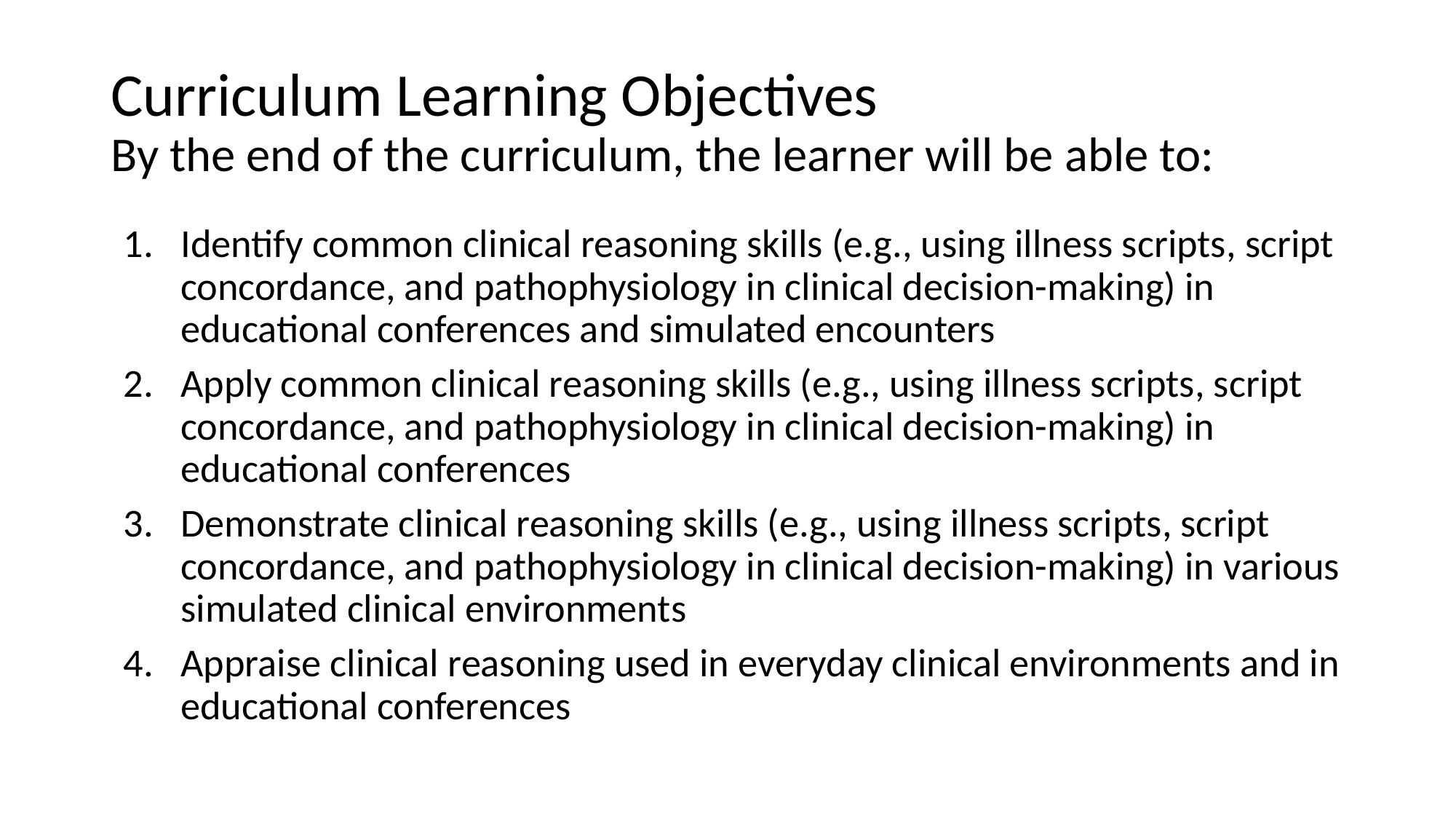

# Curriculum Learning Objectives By the end of the curriculum, the learner will be able to:
Identify common clinical reasoning skills (e.g., using illness scripts, script concordance, and pathophysiology in clinical decision-making) in educational conferences and simulated encounters
Apply common clinical reasoning skills (e.g., using illness scripts, script concordance, and pathophysiology in clinical decision-making) in educational conferences
Demonstrate clinical reasoning skills (e.g., using illness scripts, script concordance, and pathophysiology in clinical decision-making) in various simulated clinical environments
Appraise clinical reasoning used in everyday clinical environments and in educational conferences

## Slide 5
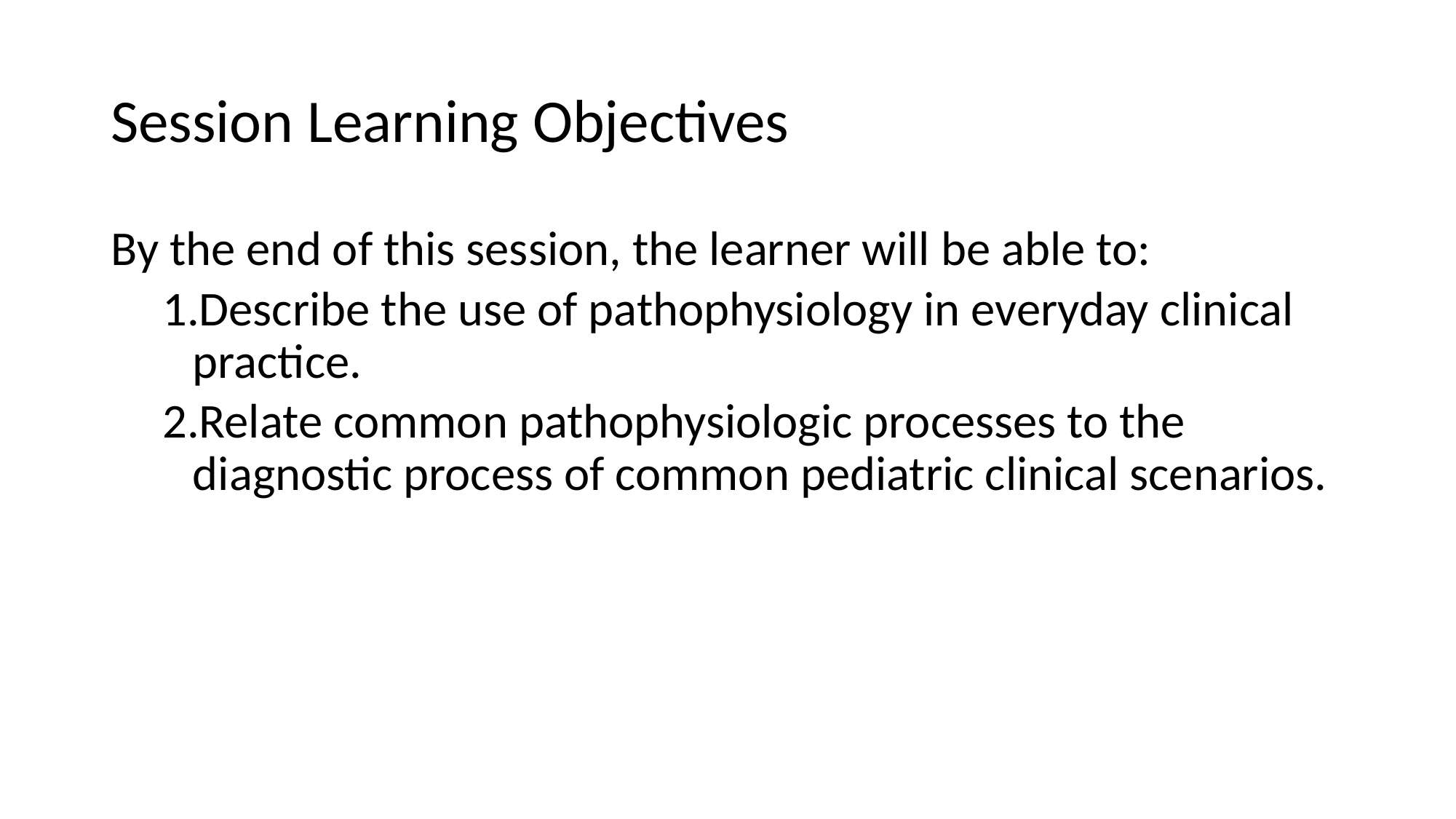

# Session Learning Objectives
By the end of this session, the learner will be able to:
Describe the use of pathophysiology in everyday clinical practice.
Relate common pathophysiologic processes to the diagnostic process of common pediatric clinical scenarios.

## Slide 6
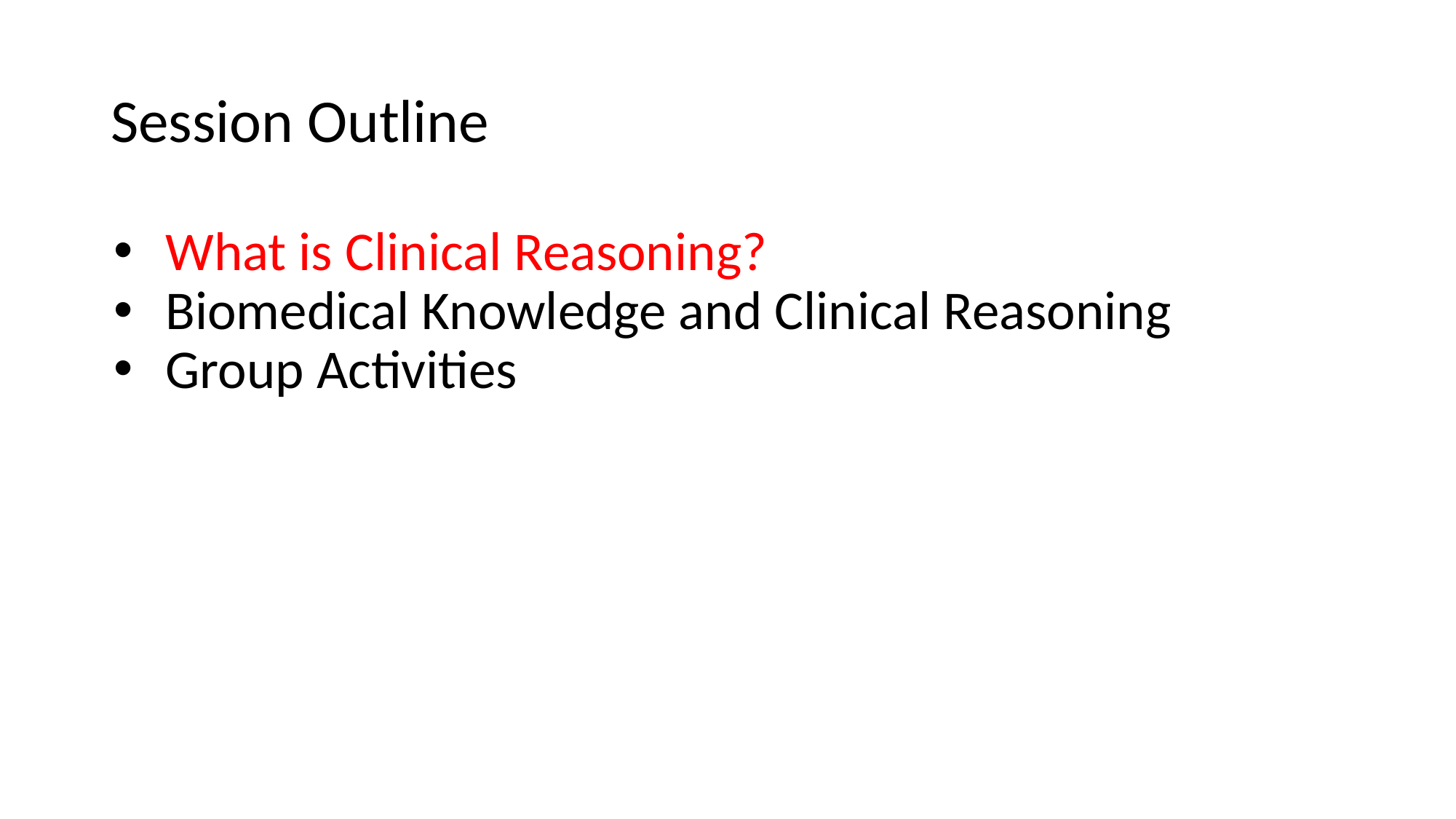

# Session Outline
What is Clinical Reasoning?
Biomedical Knowledge and Clinical Reasoning
Group Activities

## Slide 7
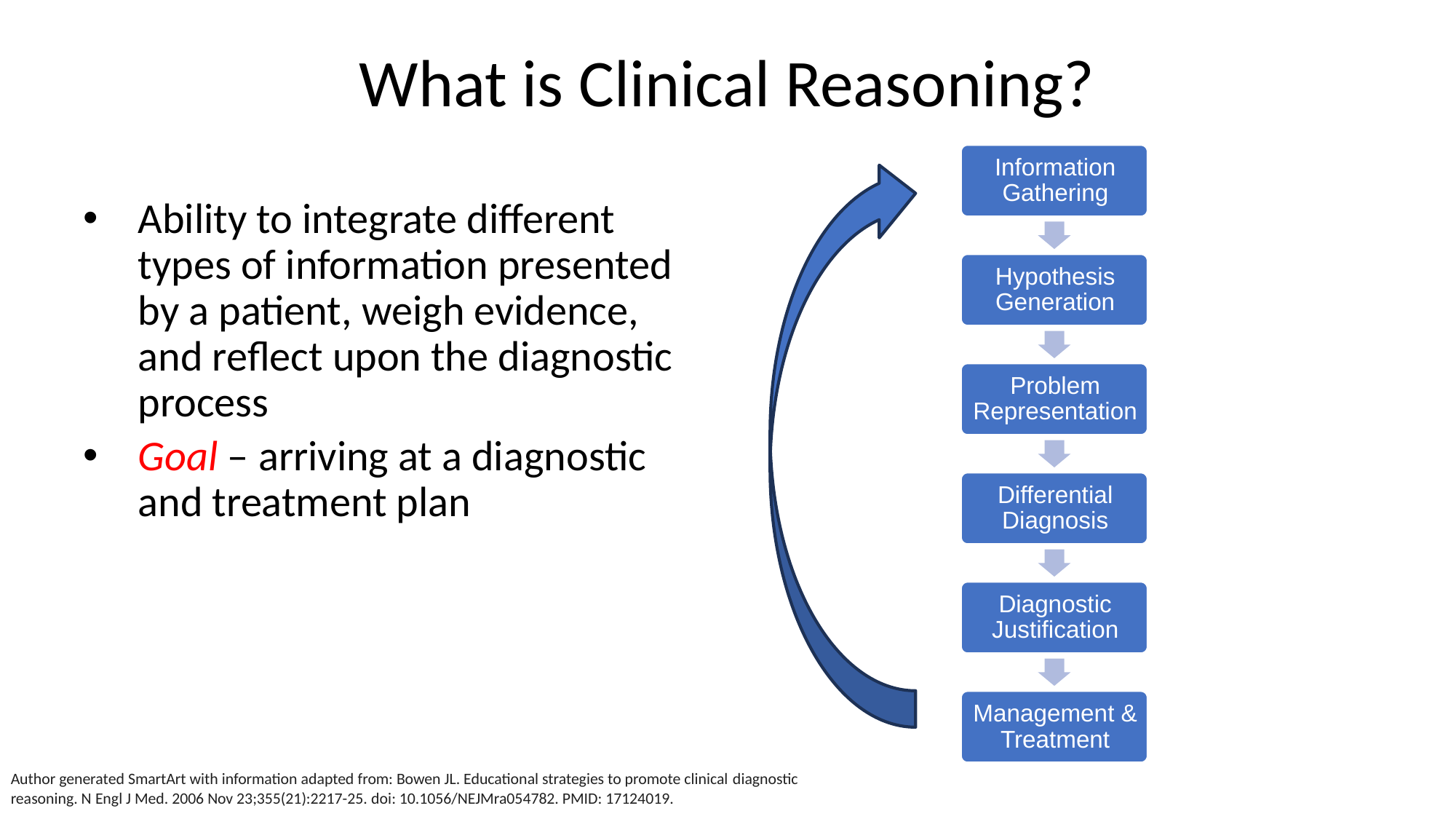

# What is Clinical Reasoning?
Ability to integrate different types of information presented by a patient, weigh evidence, and reflect upon the diagnostic process
Goal – arriving at a diagnostic and treatment plan
Author generated SmartArt with information adapted from: Bowen JL. Educational strategies to promote clinical diagnostic reasoning. N Engl J Med. 2006 Nov 23;355(21):2217-25. doi: 10.1056/NEJMra054782. PMID: 17124019.​

## Slide 8
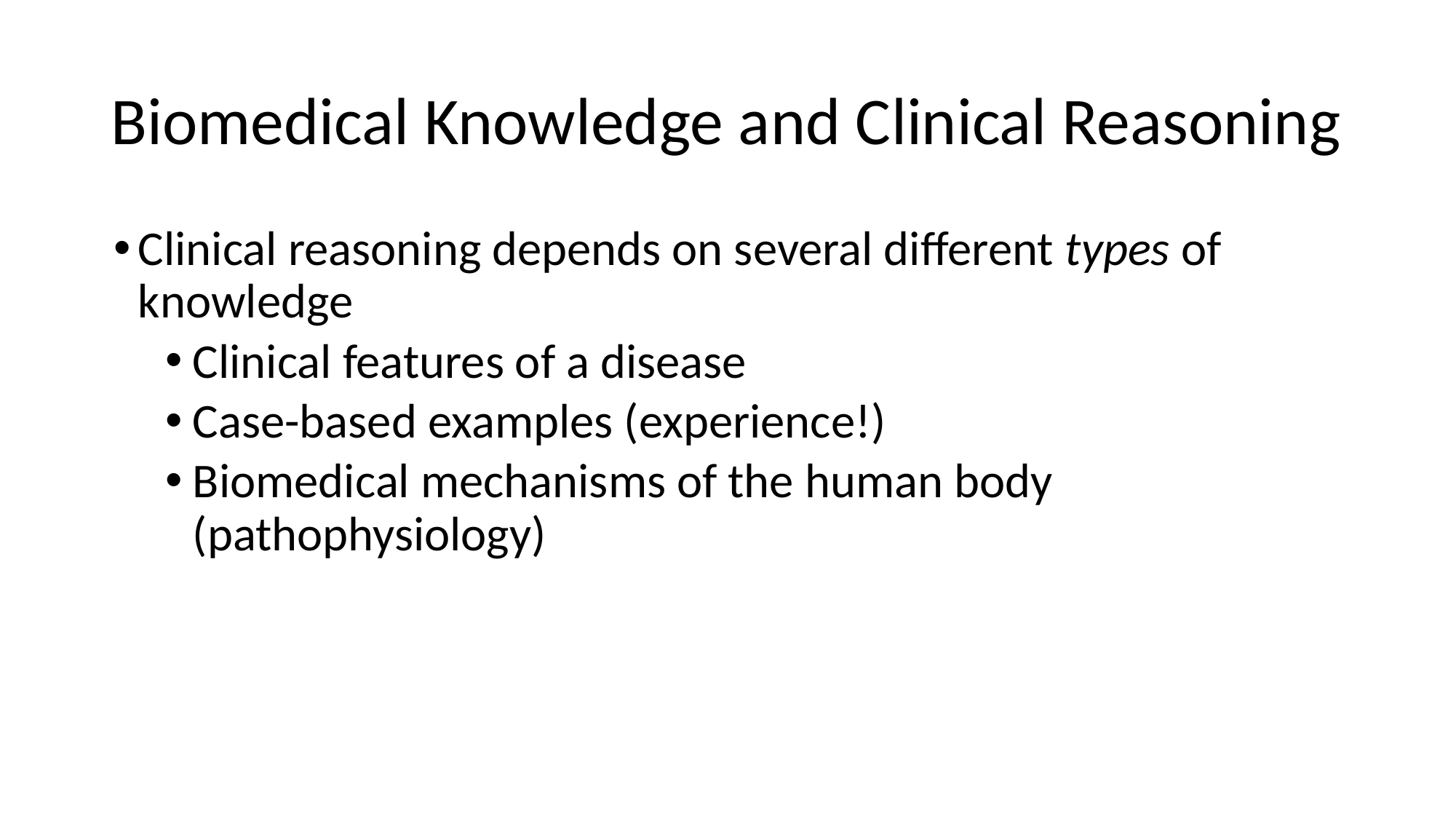

# Biomedical Knowledge and Clinical Reasoning
Clinical reasoning depends on several different types of knowledge
Clinical features of a disease
Case-based examples (experience!)
Biomedical mechanisms of the human body (pathophysiology)

## Slide 9
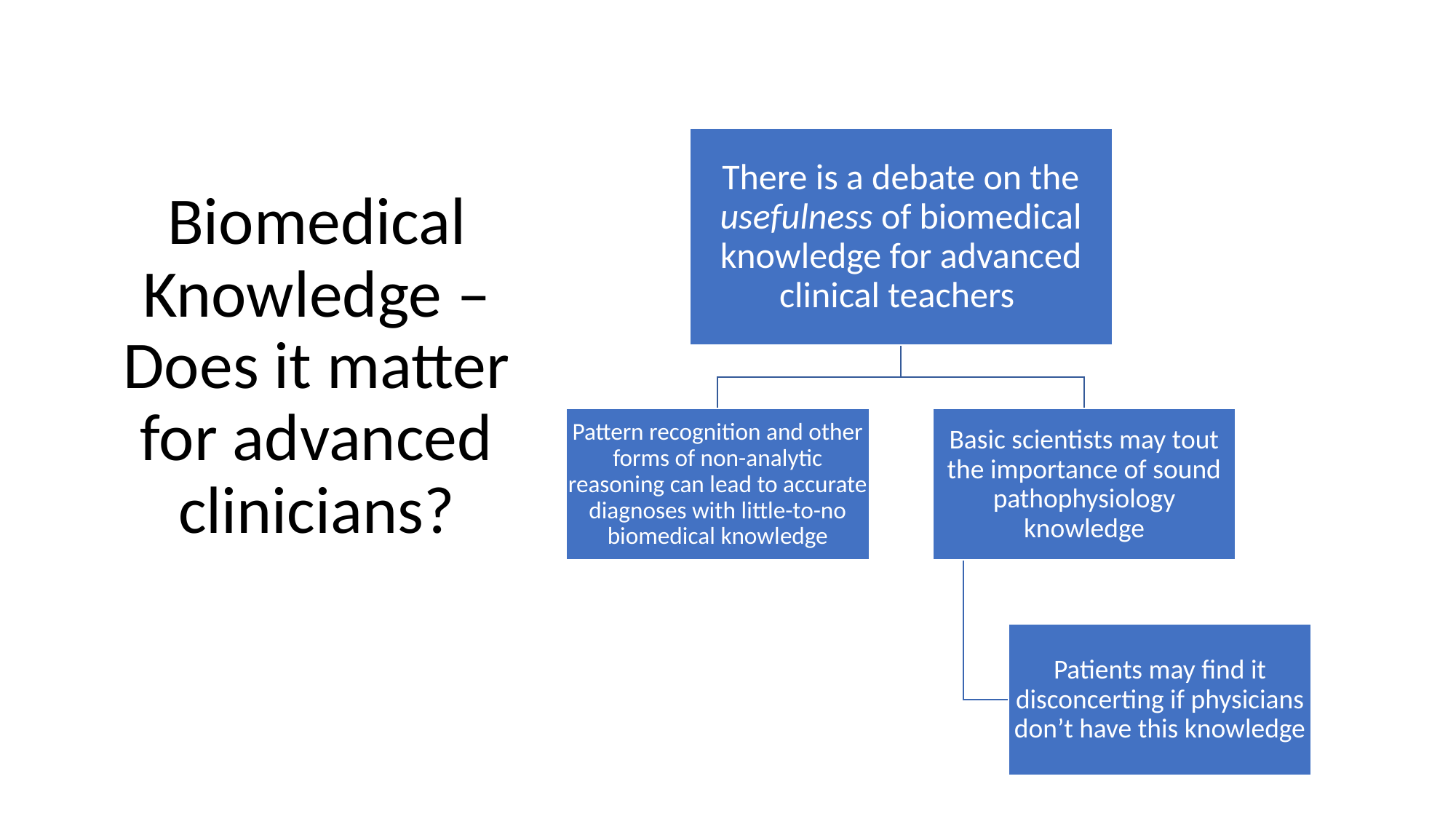

# Biomedical Knowledge – Does it matter for advanced clinicians?
There is a debate on the usefulness of biomedical knowledge for advanced clinical teachers
Pattern recognition and other forms of non-analytic reasoning can lead to accurate diagnoses with little-to-no biomedical knowledge
Basic scientists may tout the importance of sound pathophysiology knowledge
Patients may find it disconcerting if physicians don’t have this knowledge

## Slide 10
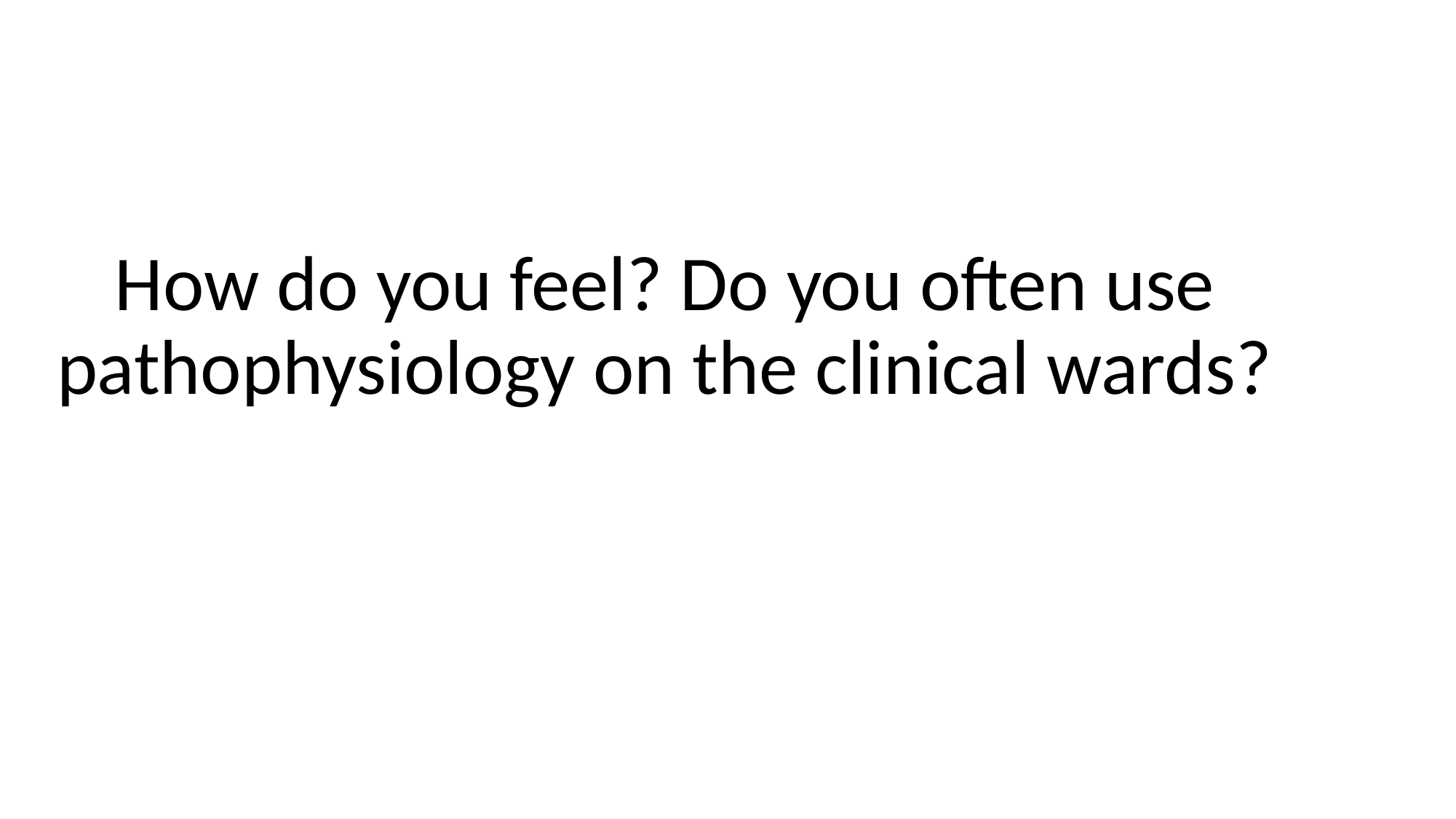

How do you feel? Do you often use pathophysiology on the clinical wards?

## Slide 11
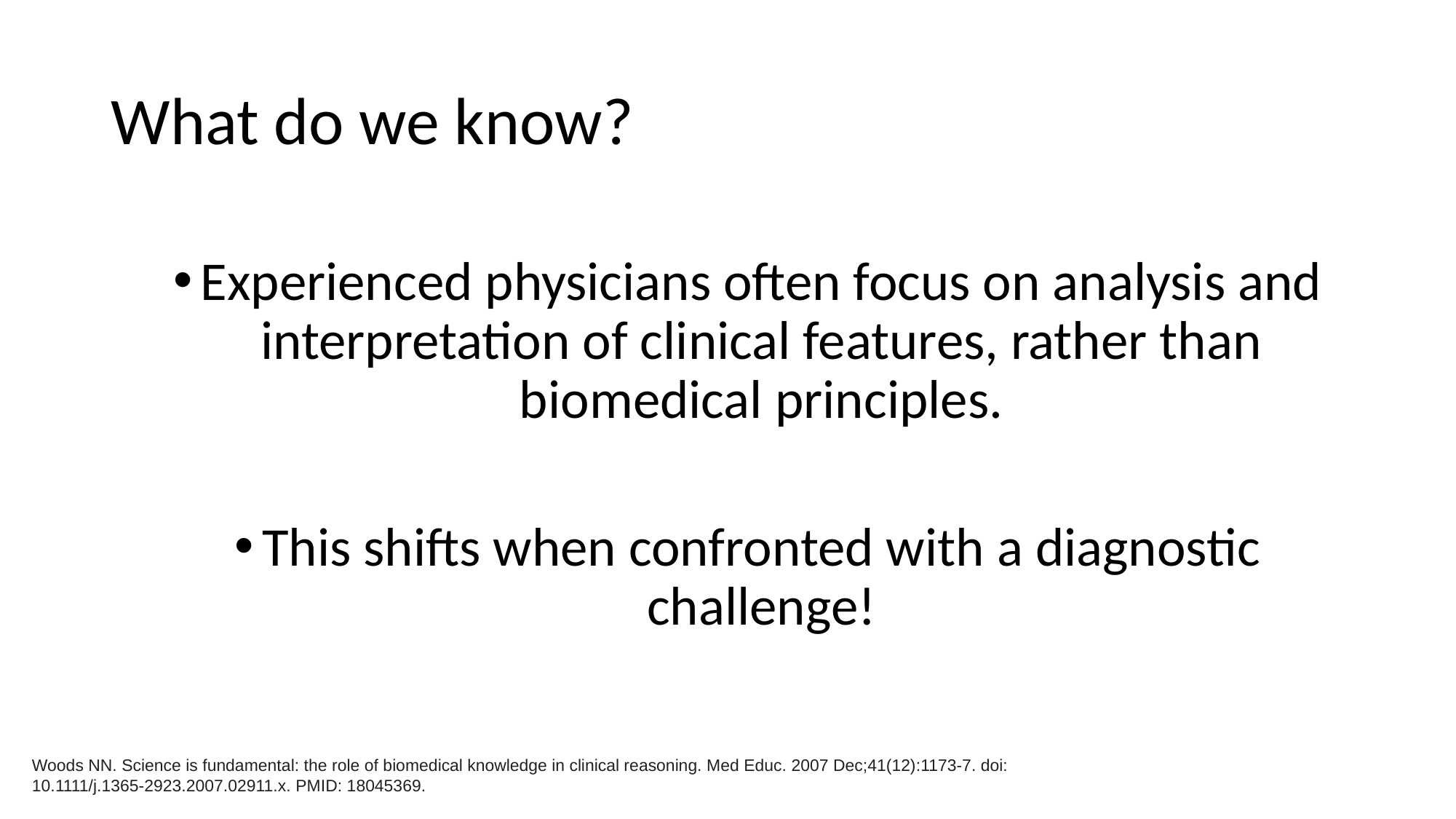

# What do we know?
Experienced physicians often focus on analysis and interpretation of clinical features, rather than biomedical principles.
This shifts when confronted with a diagnostic challenge!
Woods NN. Science is fundamental: the role of biomedical knowledge in clinical reasoning. Med Educ. 2007 Dec;41(12):1173-7. doi: 10.1111/j.1365-2923.2007.02911.x. PMID: 18045369.

## Slide 12
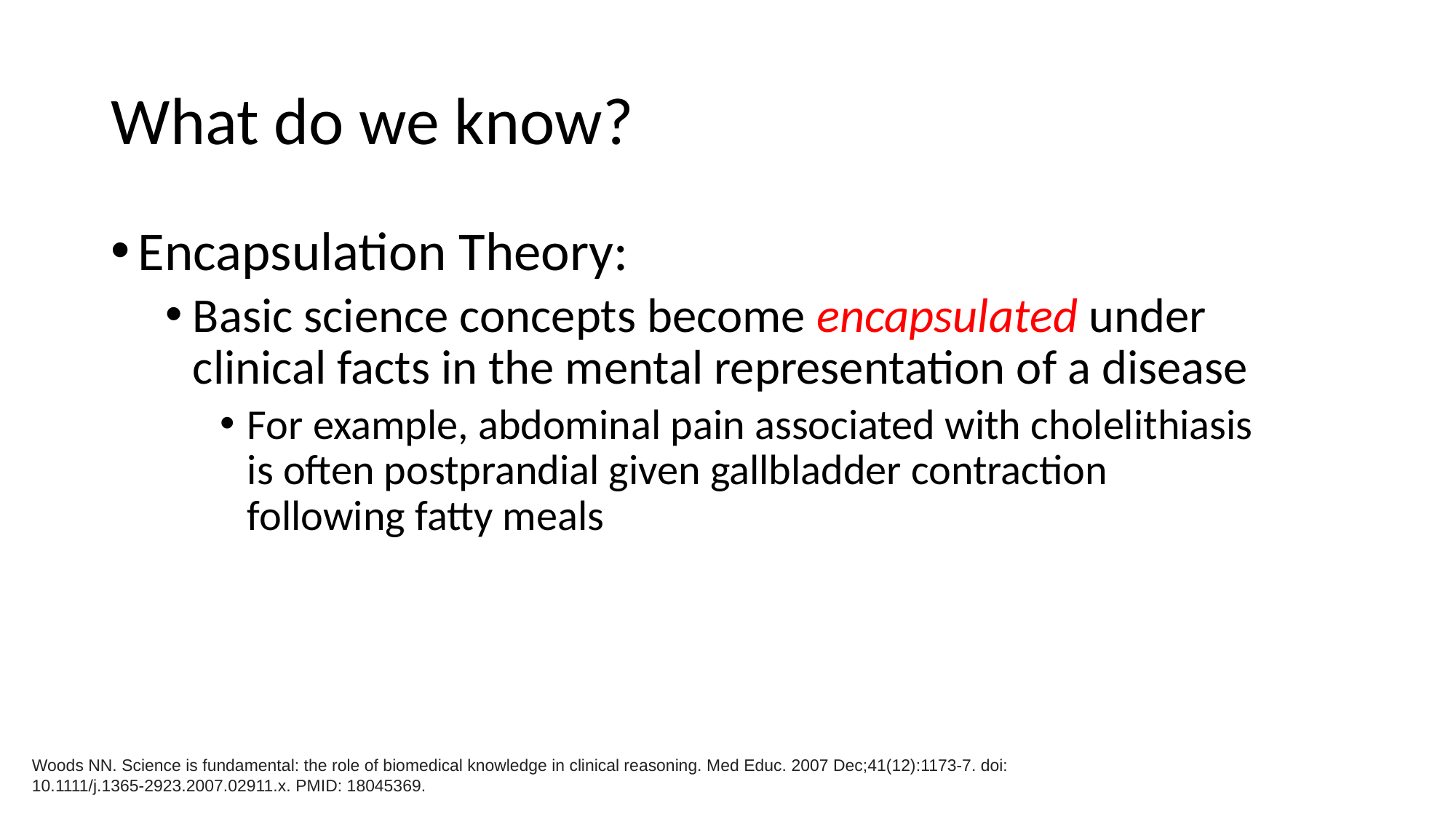

# What do we know?
Encapsulation Theory:
Basic science concepts become encapsulated under clinical facts in the mental representation of a disease
For example, abdominal pain associated with cholelithiasis is often postprandial given gallbladder contraction following fatty meals
Woods NN. Science is fundamental: the role of biomedical knowledge in clinical reasoning. Med Educ. 2007 Dec;41(12):1173-7. doi: 10.1111/j.1365-2923.2007.02911.x. PMID: 18045369.

## Slide 13
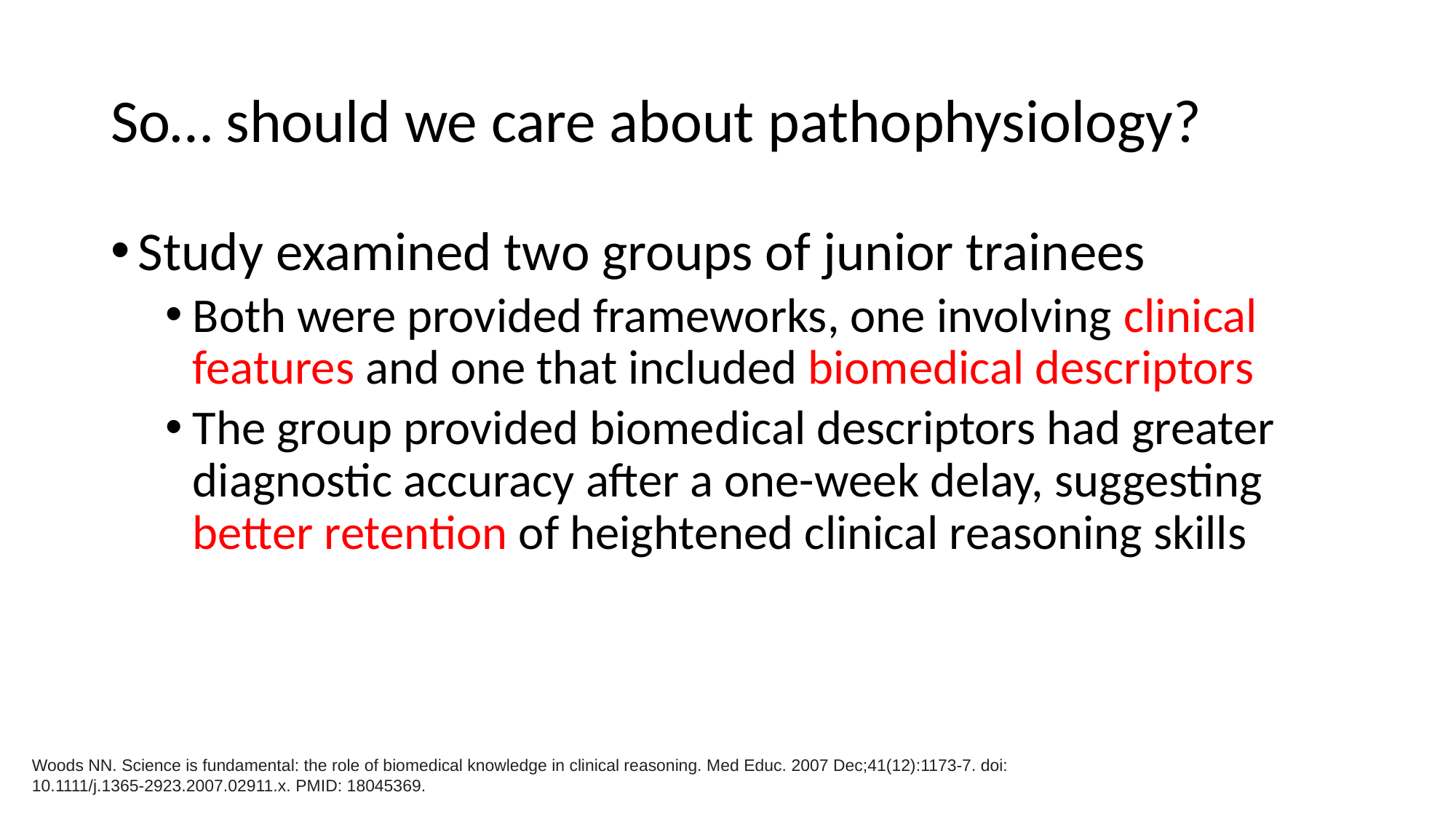

# So… should we care about pathophysiology?
Study examined two groups of junior trainees
Both were provided frameworks, one involving clinical features and one that included biomedical descriptors
The group provided biomedical descriptors had greater diagnostic accuracy after a one-week delay, suggesting better retention of heightened clinical reasoning skills
Woods NN. Science is fundamental: the role of biomedical knowledge in clinical reasoning. Med Educ. 2007 Dec;41(12):1173-7. doi: 10.1111/j.1365-2923.2007.02911.x. PMID: 18045369.

## Slide 14
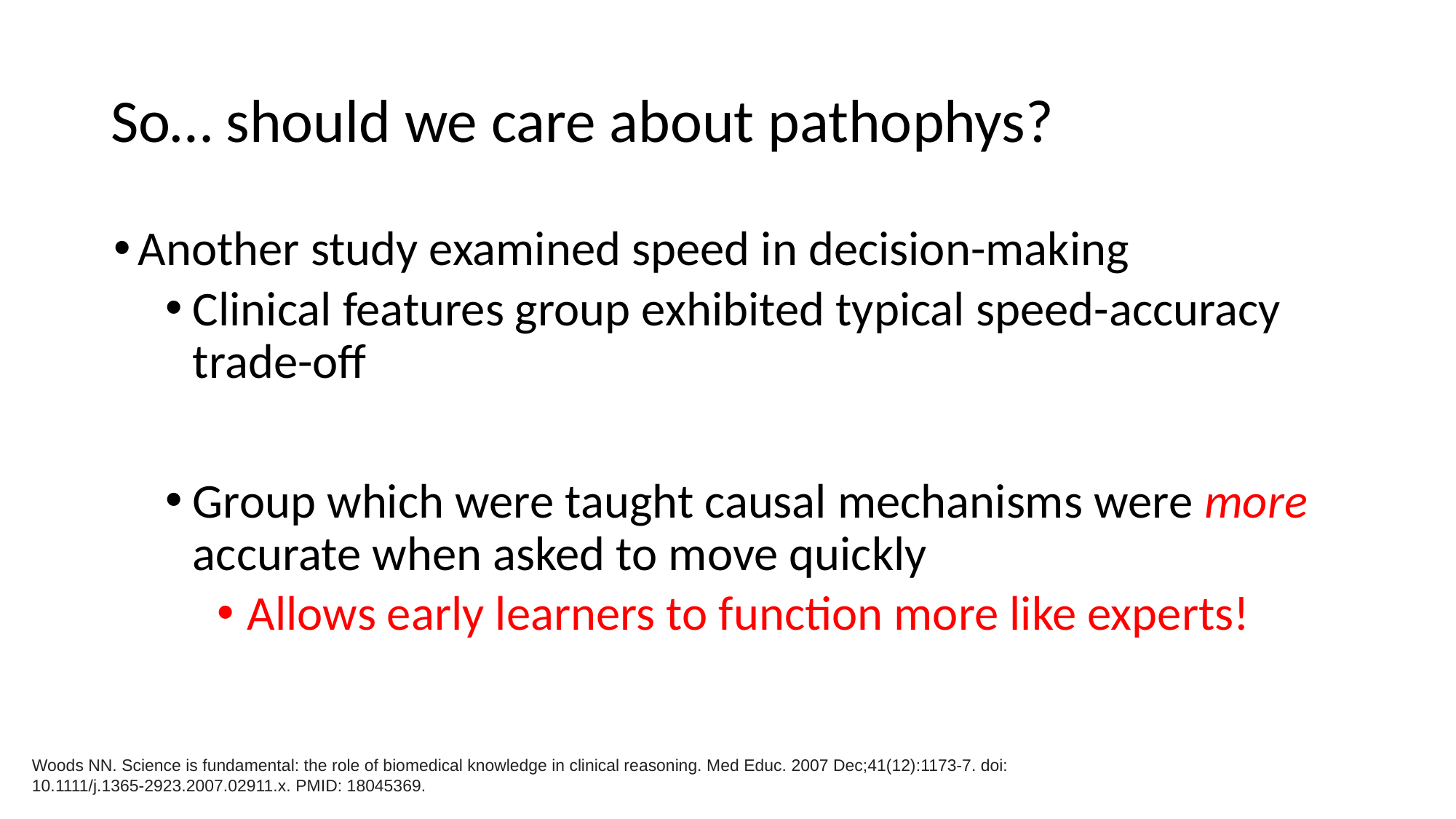

# So… should we care about pathophys?
Another study examined speed in decision-making
Clinical features group exhibited typical speed-accuracy trade-off
Group which were taught causal mechanisms were more accurate when asked to move quickly
Allows early learners to function more like experts!
Woods NN. Science is fundamental: the role of biomedical knowledge in clinical reasoning. Med Educ. 2007 Dec;41(12):1173-7. doi: 10.1111/j.1365-2923.2007.02911.x. PMID: 18045369.

## Slide 15
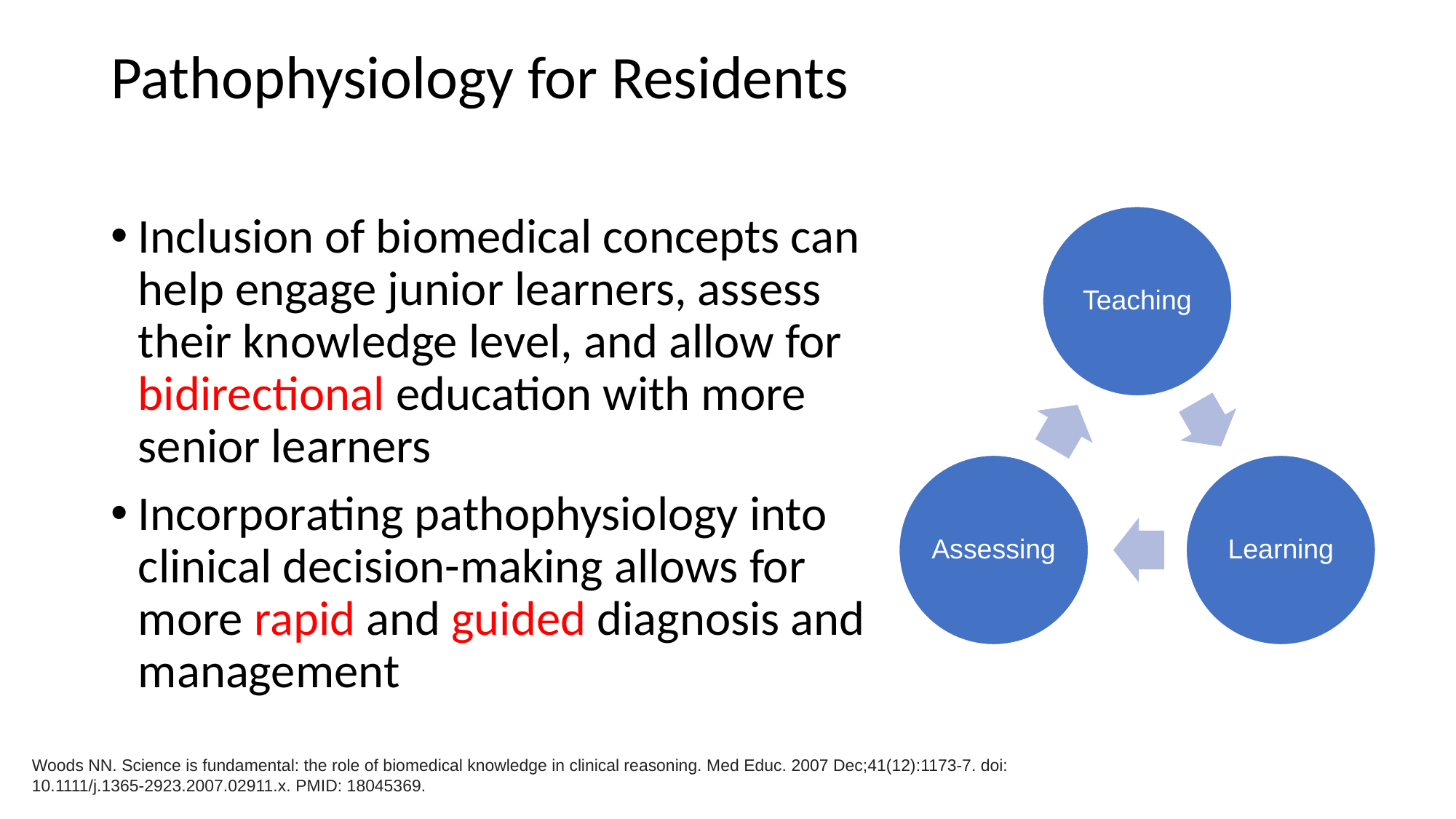

# Pathophysiology for Residents
Inclusion of biomedical concepts can help engage junior learners, assess their knowledge level, and allow for bidirectional education with more senior learners
Incorporating pathophysiology into clinical decision-making allows for more rapid and guided diagnosis and management
Woods NN. Science is fundamental: the role of biomedical knowledge in clinical reasoning. Med Educ. 2007 Dec;41(12):1173-7. doi: 10.1111/j.1365-2923.2007.02911.x. PMID: 18045369.

## Slide 16
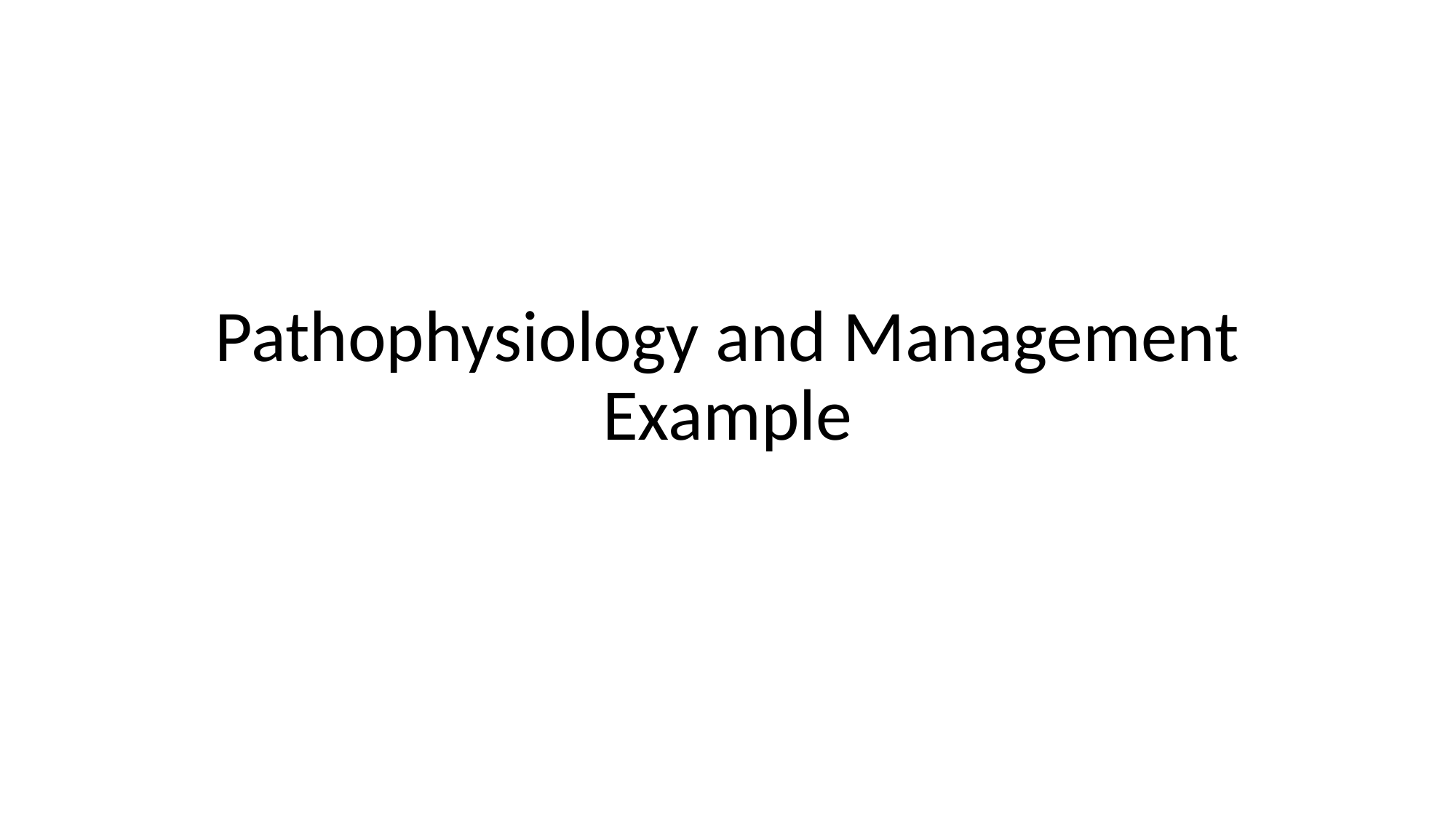

# Pathophysiology and Management Example

## Slide 17
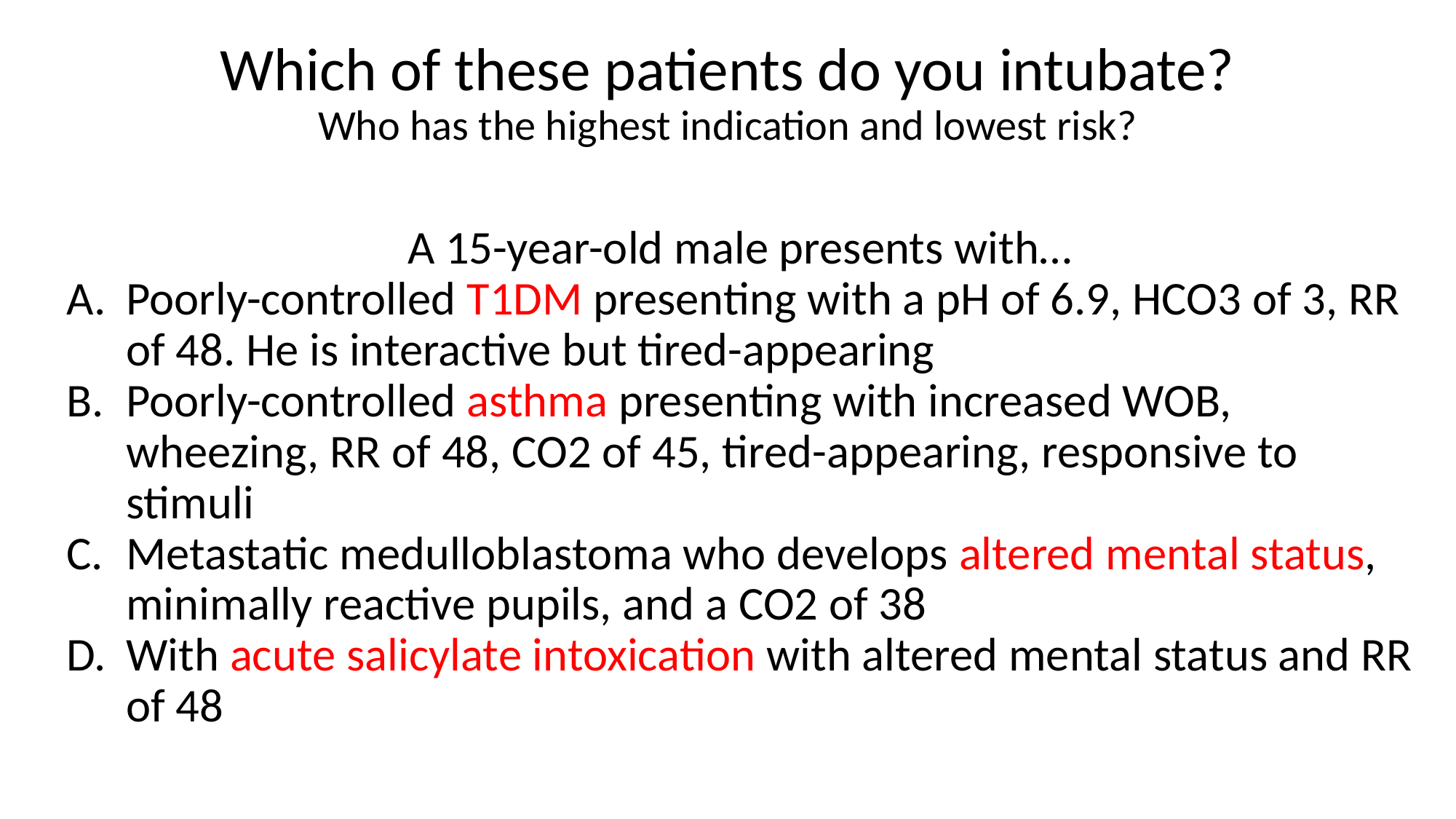

# Which of these patients do you intubate? Who has the highest indication and lowest risk?
A 15-year-old male presents with…
Poorly-controlled T1DM presenting with a pH of 6.9, HCO3 of 3, RR of 48. He is interactive but tired-appearing
Poorly-controlled asthma presenting with increased WOB, wheezing, RR of 48, CO2 of 45, tired-appearing, responsive to stimuli
Metastatic medulloblastoma who develops altered mental status, minimally reactive pupils, and a CO2 of 38
With acute salicylate intoxication with altered mental status and RR of 48

## Slide 18
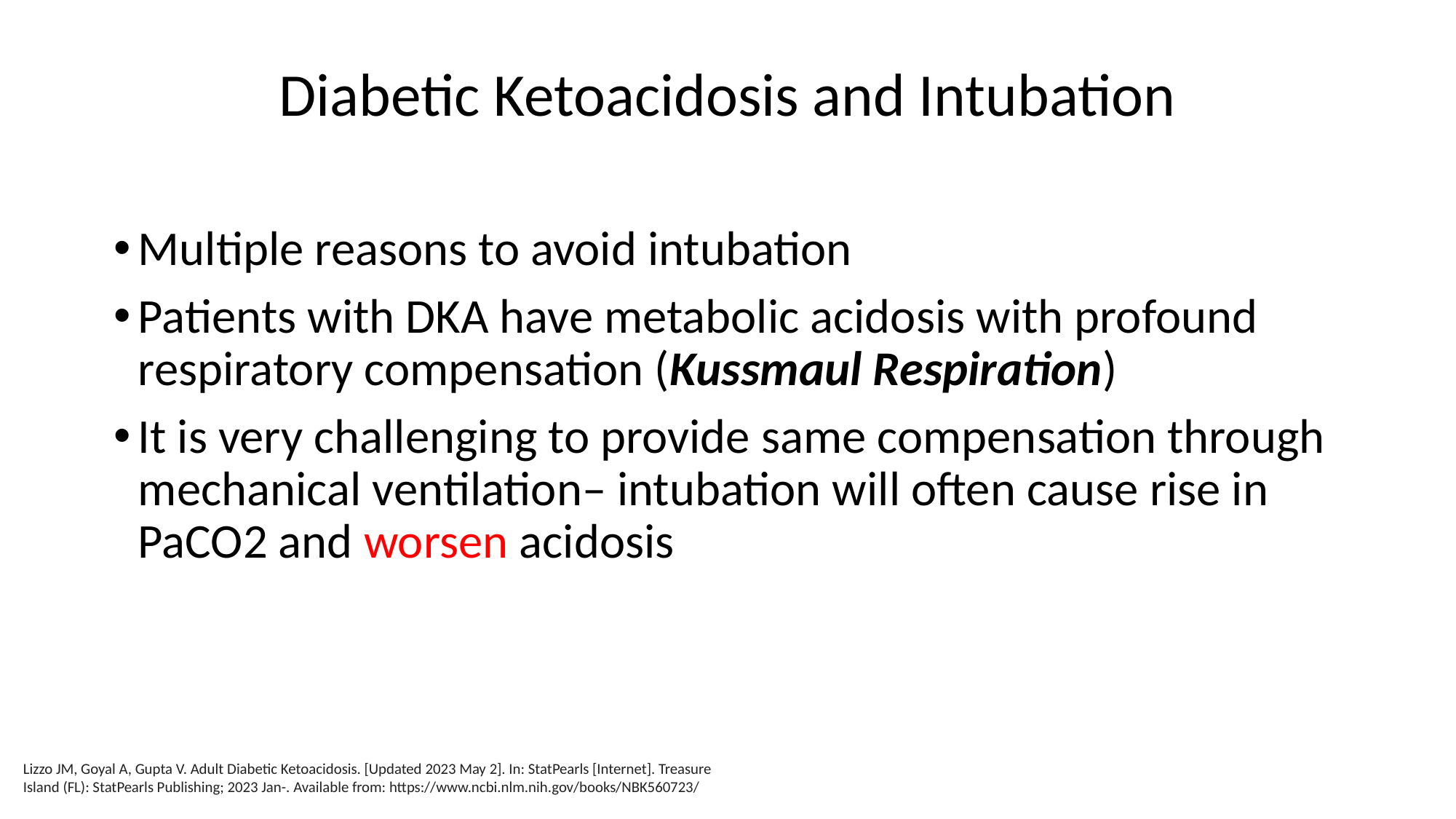

# Diabetic Ketoacidosis and Intubation
Multiple reasons to avoid intubation
Patients with DKA have metabolic acidosis with profound respiratory compensation (Kussmaul Respiration)
It is very challenging to provide same compensation through mechanical ventilation– intubation will often cause rise in PaCO2 and worsen acidosis
Lizzo JM, Goyal A, Gupta V. Adult Diabetic Ketoacidosis. [Updated 2023 May 2]. In: StatPearls [Internet]. Treasure Island (FL): StatPearls Publishing; 2023 Jan-. Available from: https://www.ncbi.nlm.nih.gov/books/NBK560723/

## Slide 19
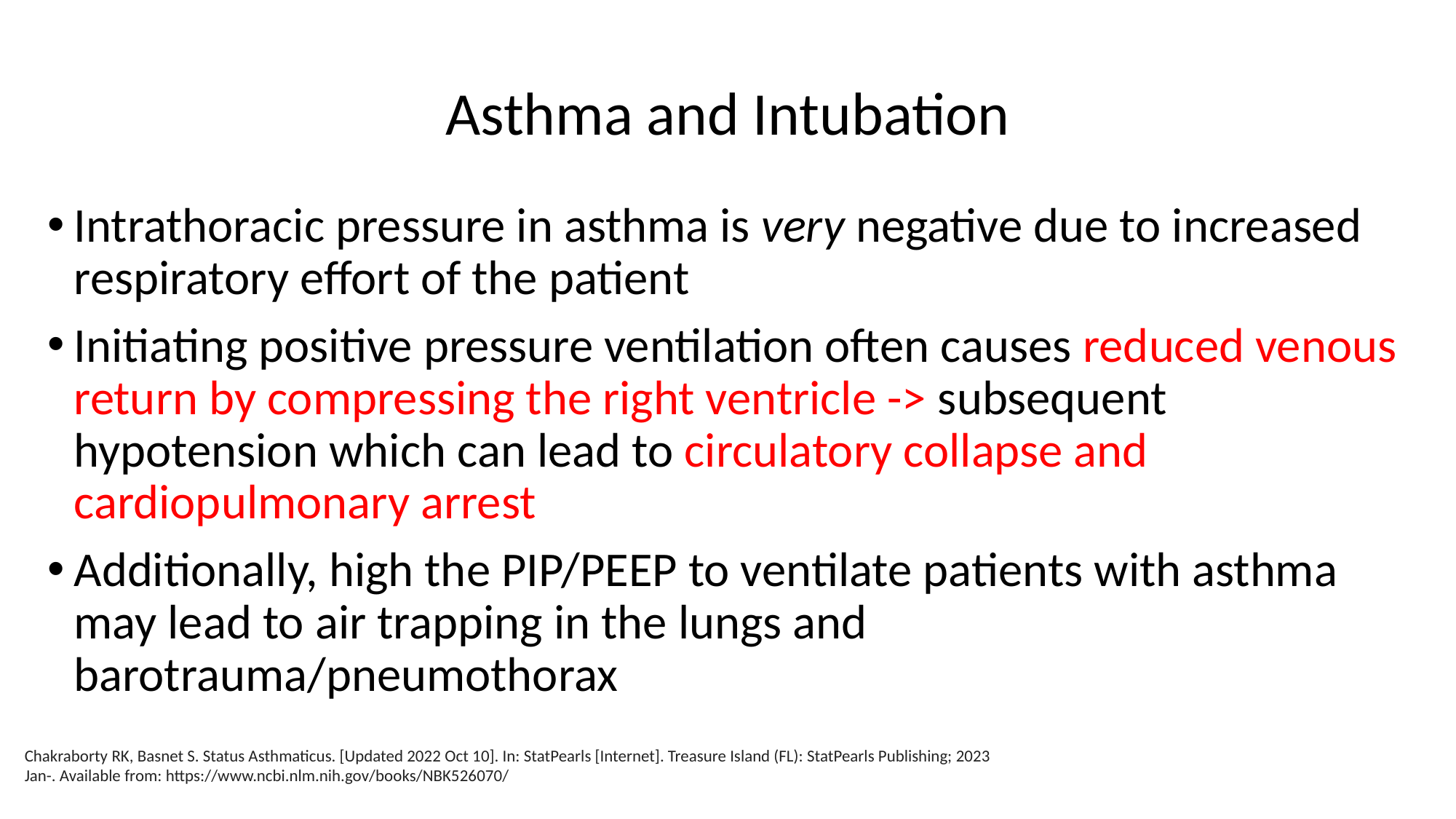

# Asthma and Intubation
Intrathoracic pressure in asthma is very negative due to increased respiratory effort of the patient
Initiating positive pressure ventilation often causes reduced venous return by compressing the right ventricle -> subsequent hypotension which can lead to circulatory collapse and cardiopulmonary arrest
Additionally, high the PIP/PEEP to ventilate patients with asthma may lead to air trapping in the lungs and barotrauma/pneumothorax
Chakraborty RK, Basnet S. Status Asthmaticus. [Updated 2022 Oct 10]. In: StatPearls [Internet]. Treasure Island (FL): StatPearls Publishing; 2023 Jan-. Available from: https://www.ncbi.nlm.nih.gov/books/NBK526070/

## Slide 20
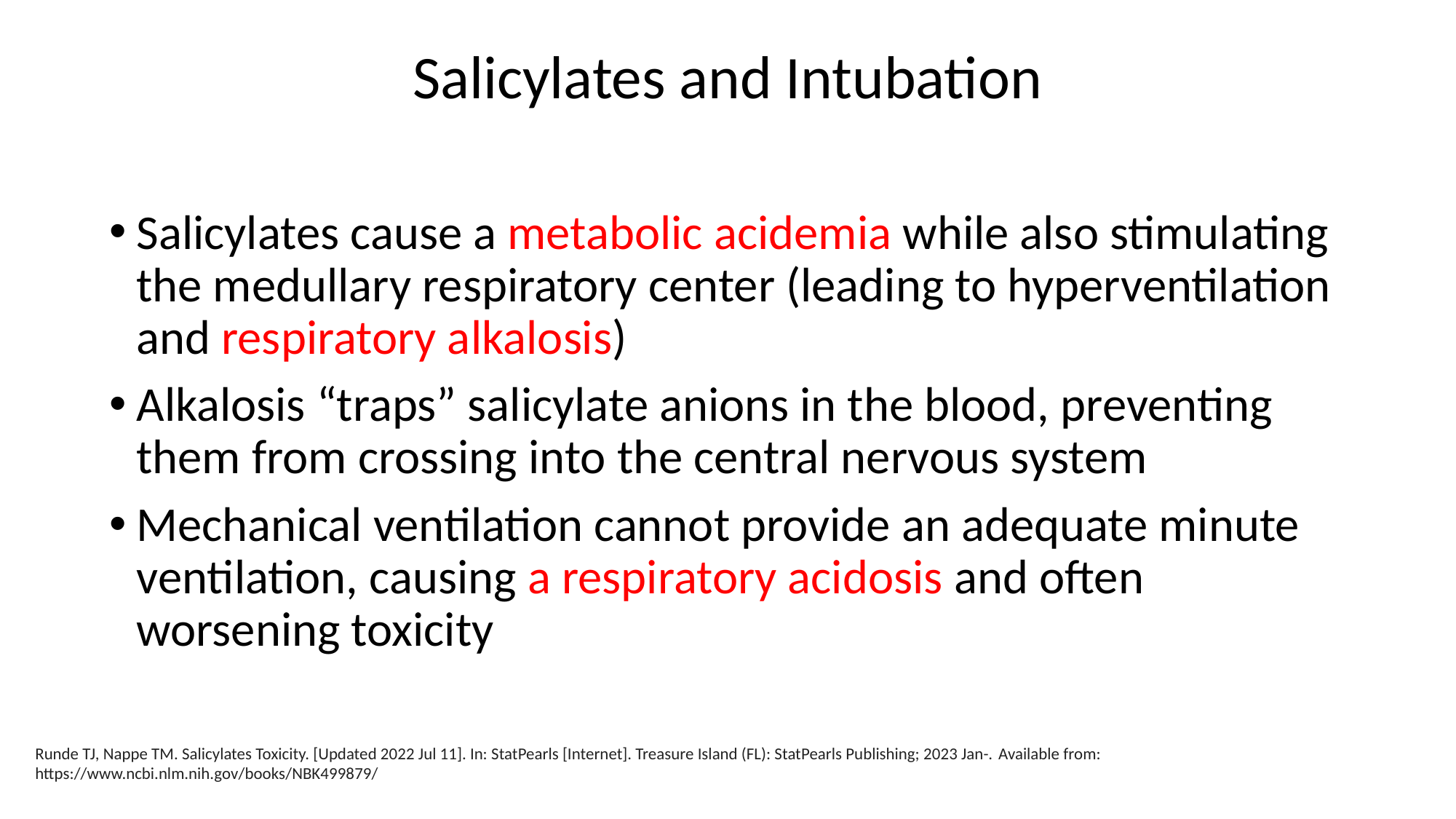

# Salicylates and Intubation
Salicylates cause a metabolic acidemia while also stimulating the medullary respiratory center (leading to hyperventilation and respiratory alkalosis)
Alkalosis “traps” salicylate anions in the blood, preventing them from crossing into the central nervous system
Mechanical ventilation cannot provide an adequate minute ventilation, causing a respiratory acidosis and often worsening toxicity
Runde TJ, Nappe TM. Salicylates Toxicity. [Updated 2022 Jul 11]. In: StatPearls [Internet]. Treasure Island (FL): StatPearls Publishing; 2023 Jan-. Available from: https://www.ncbi.nlm.nih.gov/books/NBK499879/

## Slide 21
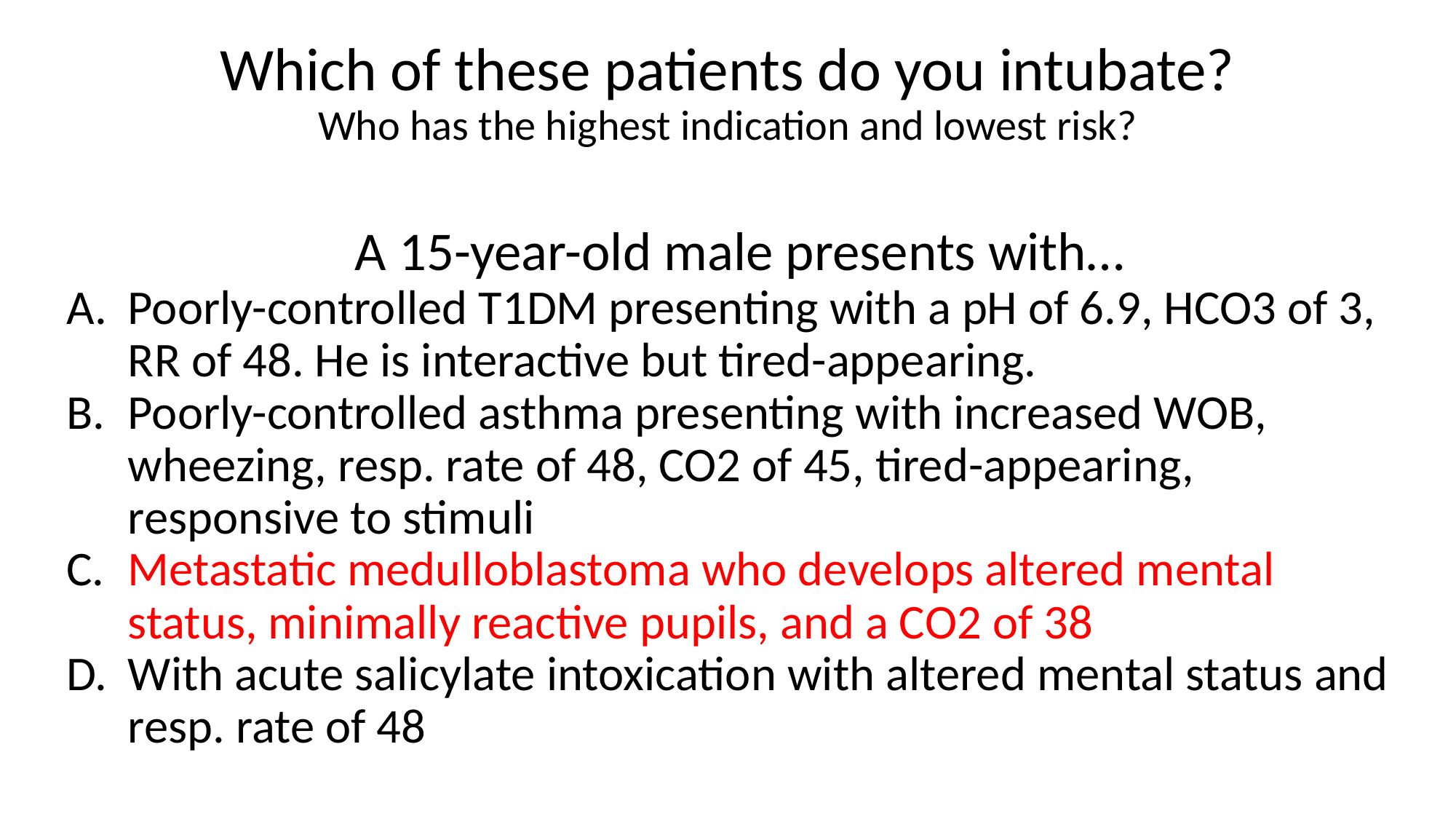

# Which of these patients do you intubate? Who has the highest indication and lowest risk?
A 15-year-old male presents with…
Poorly-controlled T1DM presenting with a pH of 6.9, HCO3 of 3, RR of 48. He is interactive but tired-appearing.
Poorly-controlled asthma presenting with increased WOB, wheezing, resp. rate of 48, CO2 of 45, tired-appearing, responsive to stimuli
Metastatic medulloblastoma who develops altered mental status, minimally reactive pupils, and a CO2 of 38
With acute salicylate intoxication with altered mental status and resp. rate of 48

## Slide 22
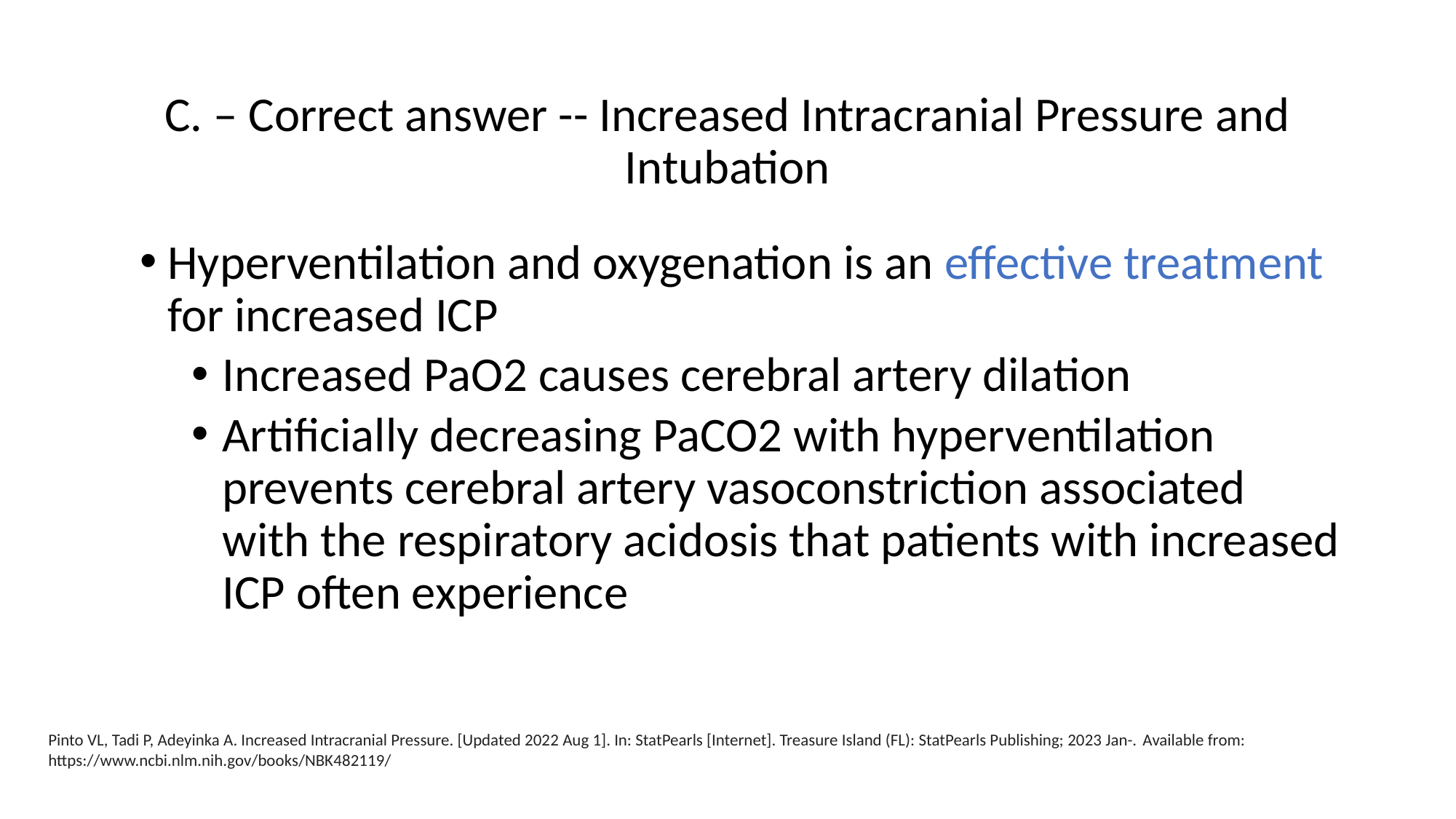

# C. – Correct answer -- Increased Intracranial Pressure and Intubation
Hyperventilation and oxygenation is an effective treatment for increased ICP
Increased PaO2 causes cerebral artery dilation
Artificially decreasing PaCO2 with hyperventilation prevents cerebral artery vasoconstriction associated with the respiratory acidosis that patients with increased ICP often experience
Pinto VL, Tadi P, Adeyinka A. Increased Intracranial Pressure. [Updated 2022 Aug 1]. In: StatPearls [Internet]. Treasure Island (FL): StatPearls Publishing; 2023 Jan-. Available from: https://www.ncbi.nlm.nih.gov/books/NBK482119/

## Slide 23
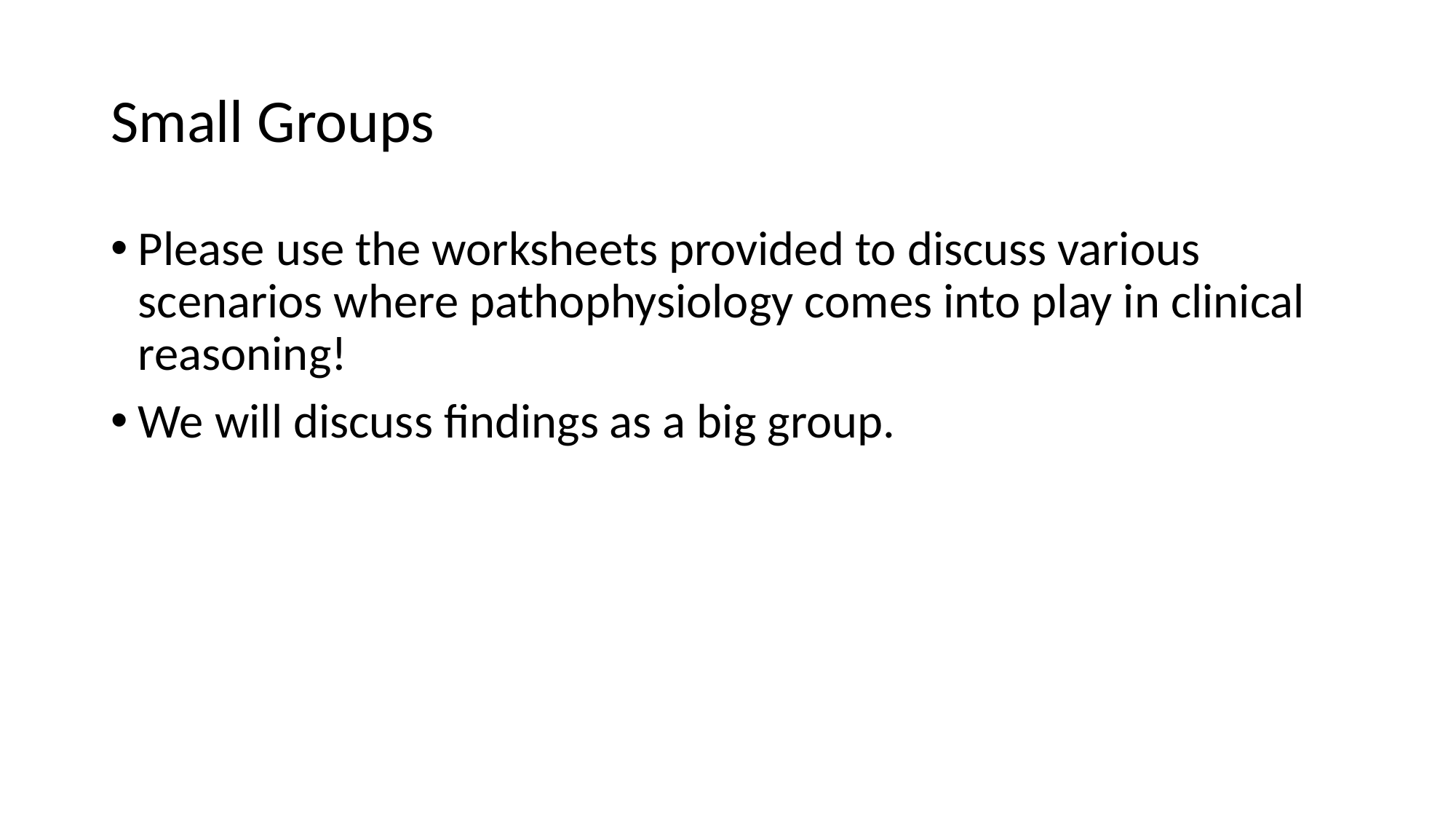

# Small Groups
Please use the worksheets provided to discuss various scenarios where pathophysiology comes into play in clinical reasoning!
We will discuss findings as a big group.

## Slide 24
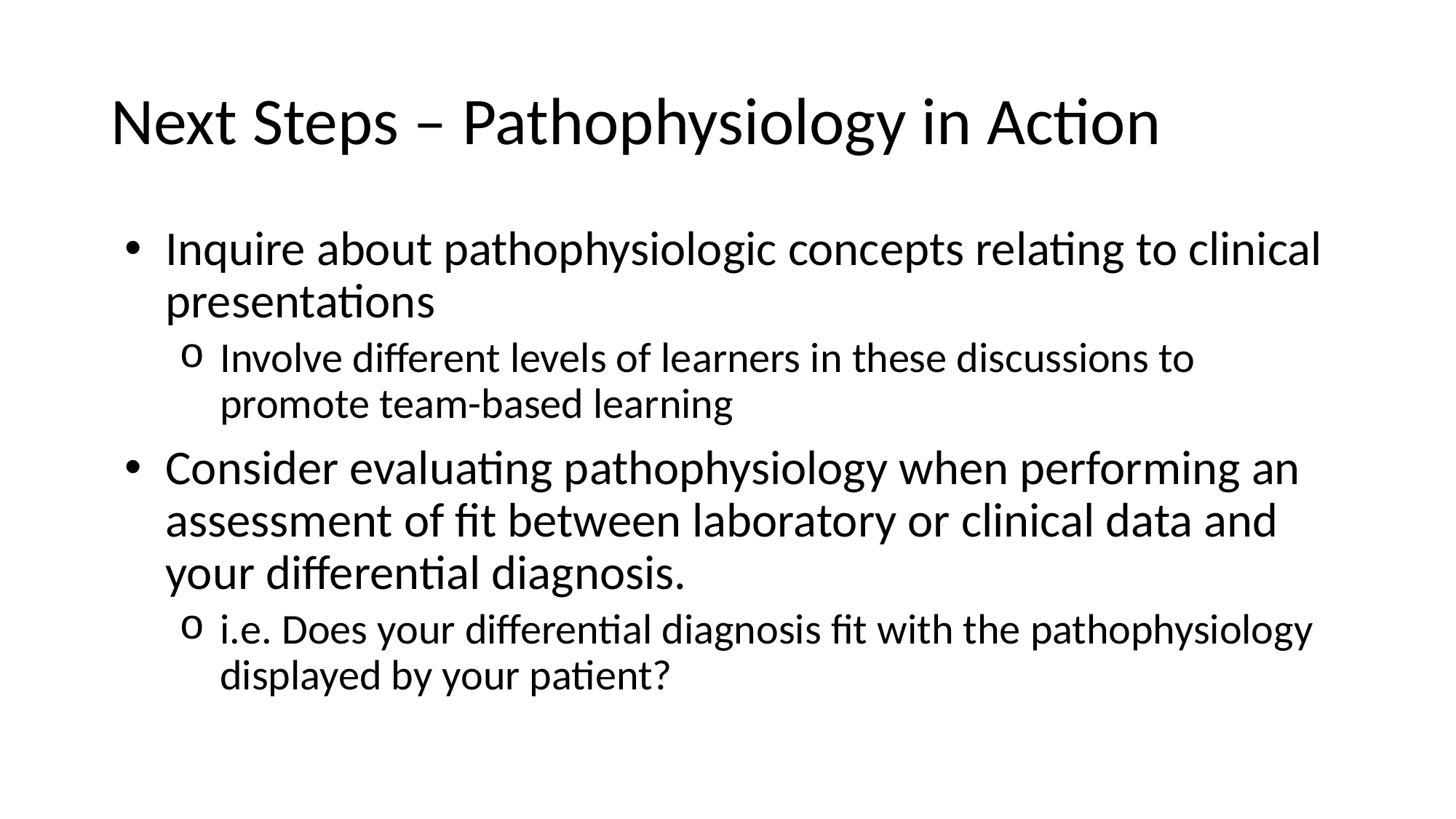

# Next Steps – Pathophysiology in Action
Inquire about pathophysiologic concepts relating to clinical presentations
Involve different levels of learners in these discussions to promote team-based learning
Consider evaluating pathophysiology when performing an assessment of fit between laboratory or clinical data and your differential diagnosis.
i.e. Does your differential diagnosis fit with the pathophysiology displayed by your patient?

## Slide 25
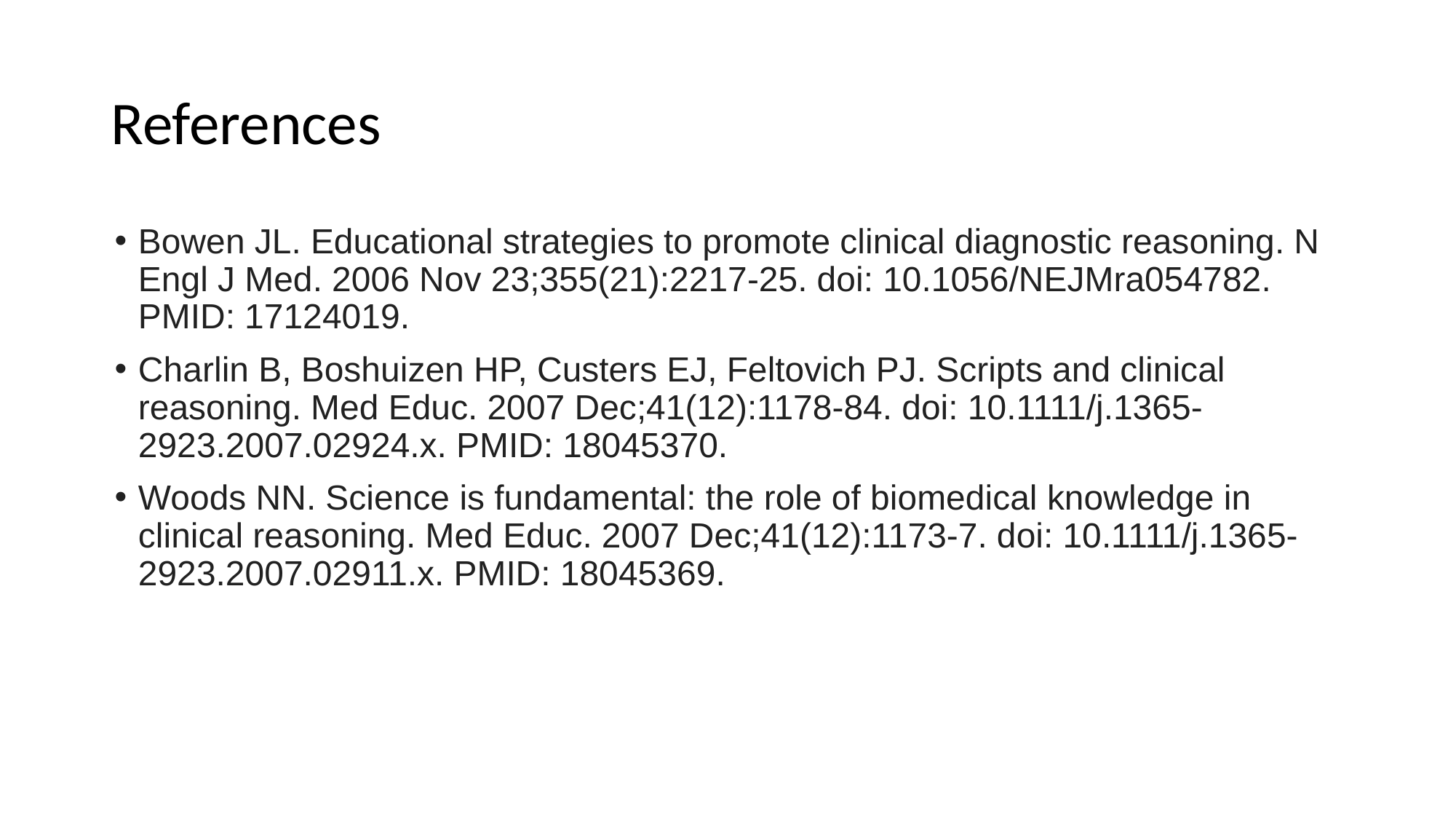

# References
Bowen JL. Educational strategies to promote clinical diagnostic reasoning. N Engl J Med. 2006 Nov 23;355(21):2217-25. doi: 10.1056/NEJMra054782. PMID: 17124019.
Charlin B, Boshuizen HP, Custers EJ, Feltovich PJ. Scripts and clinical reasoning. Med Educ. 2007 Dec;41(12):1178-84. doi: 10.1111/j.1365-2923.2007.02924.x. PMID: 18045370.
Woods NN. Science is fundamental: the role of biomedical knowledge in clinical reasoning. Med Educ. 2007 Dec;41(12):1173-7. doi: 10.1111/j.1365-2923.2007.02911.x. PMID: 18045369.
