# Supplementary material for: A Pilot Longitudinal Clinical Reasoning Curriculum for Pediatric Residents
Source: MedEdPORTAL. 2024 Sep 25;20:11447. doi: 10.15766/mep_2374-8265.11447 (PMC11422513; doi:10.15766/mep_2374-8265.11447)
Supplement: Supplementary file 1 — Preimplementation Survey.docxCurriculum Goals, Objectives, and Timeline.docxSession 1 - Illness Scripts.pptxSession 1 - Small-Group Facilitator Guide.docxSession 2 - Illness Scripts 2.pptxSession 2 - Small-Group Facilitator Guide.docxSession 3 - Script Concordance.pptxSession 3 - Small-Group Facilitator Guide.docxSession 3 - Small-Group Handout.docxSession 4 - Pathophysiology.pptxSession 4 - Small-Group Facilitator Guide.docxSession 4 - Small-Group Handout.docxSession 5 - Review Game.pptxPostimplementation Survey.docx [file mep_2374-8265.11447-s001.zip › M. Session 5 - Review Game.pptx]

## Slide 1
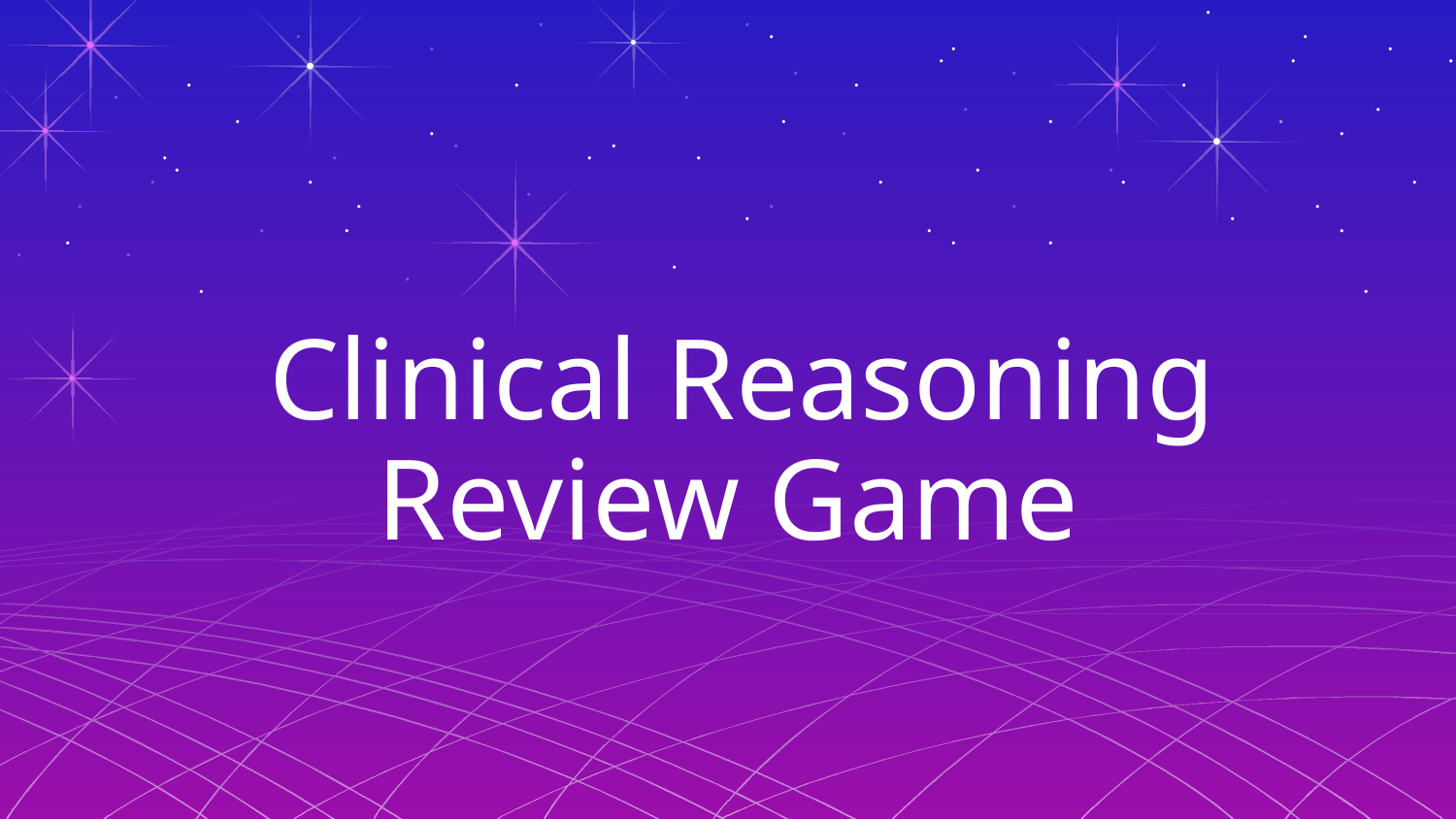

# Clinical Reasoning Review Game

## Slide 2
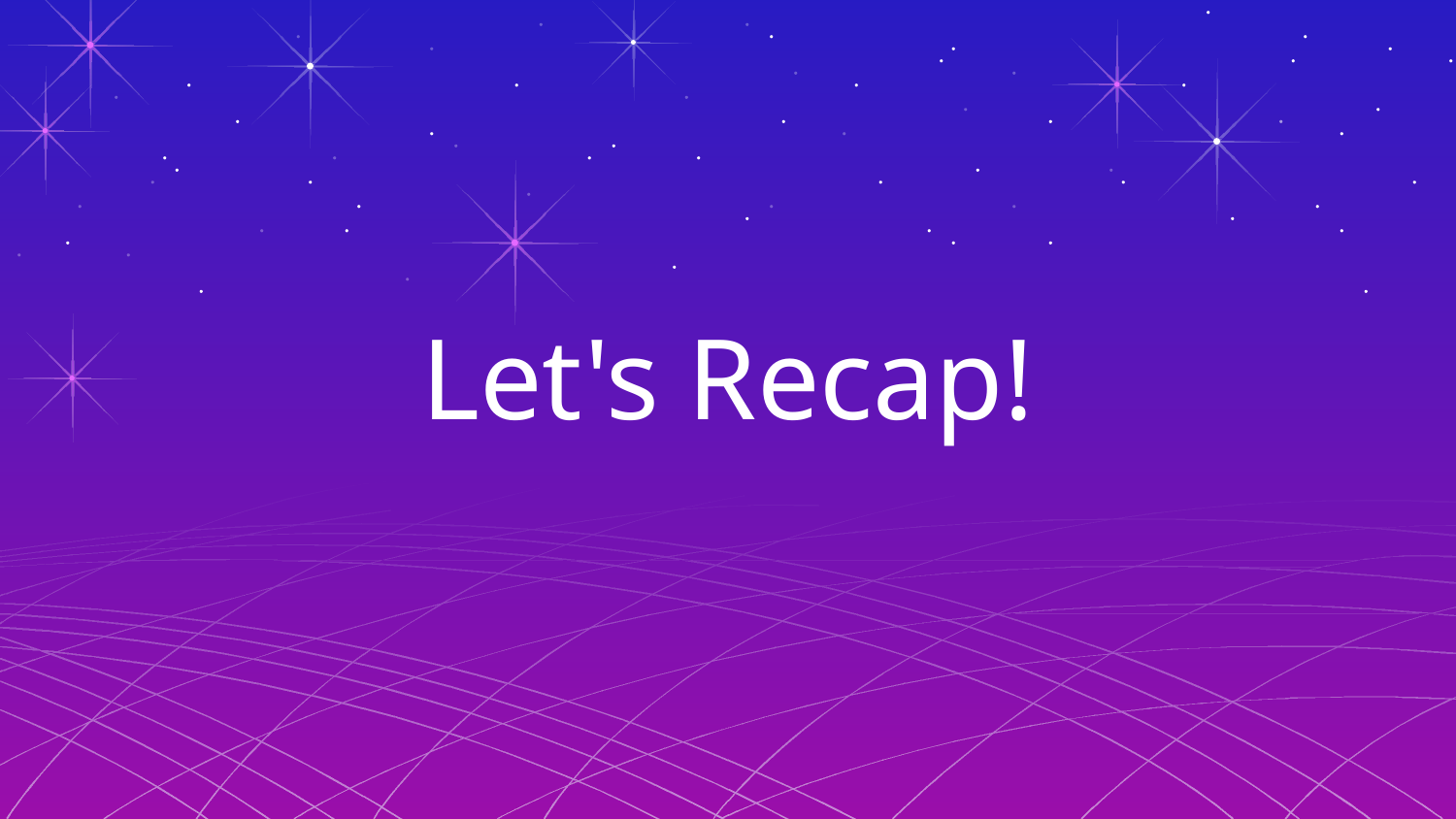

# Let's Recap!

## Slide 3
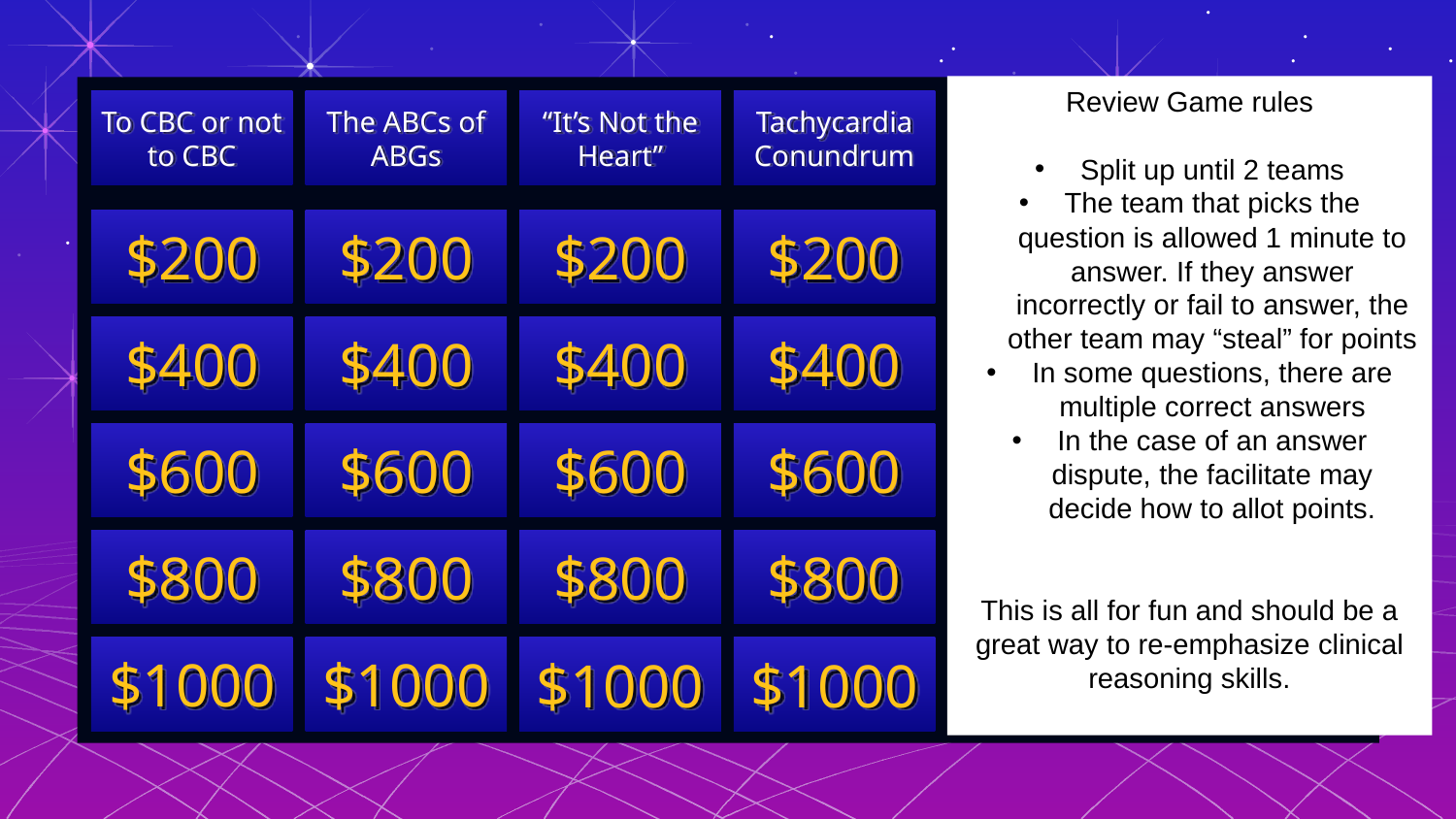

Review Game rules
Split up until 2 teams
The team that picks the question is allowed 1 minute to answer. If they answer incorrectly or fail to answer, the other team may “steal” for points
In some questions, there are multiple correct answers
In the case of an answer dispute, the facilitate may decide how to allot points.
This is all for fun and should be a great way to re-emphasize clinical reasoning skills.
To CBC or not to CBC
The ABCs of ABGs
“It’s Not the Heart”
Tachycardia Conundrum
$200
$200
$200
$200
$400
$400
$400
$400
$600
$600
$600
$600
$800
$800
$800
$800
$1000
$1000
$1000
$1000

## Slide 4
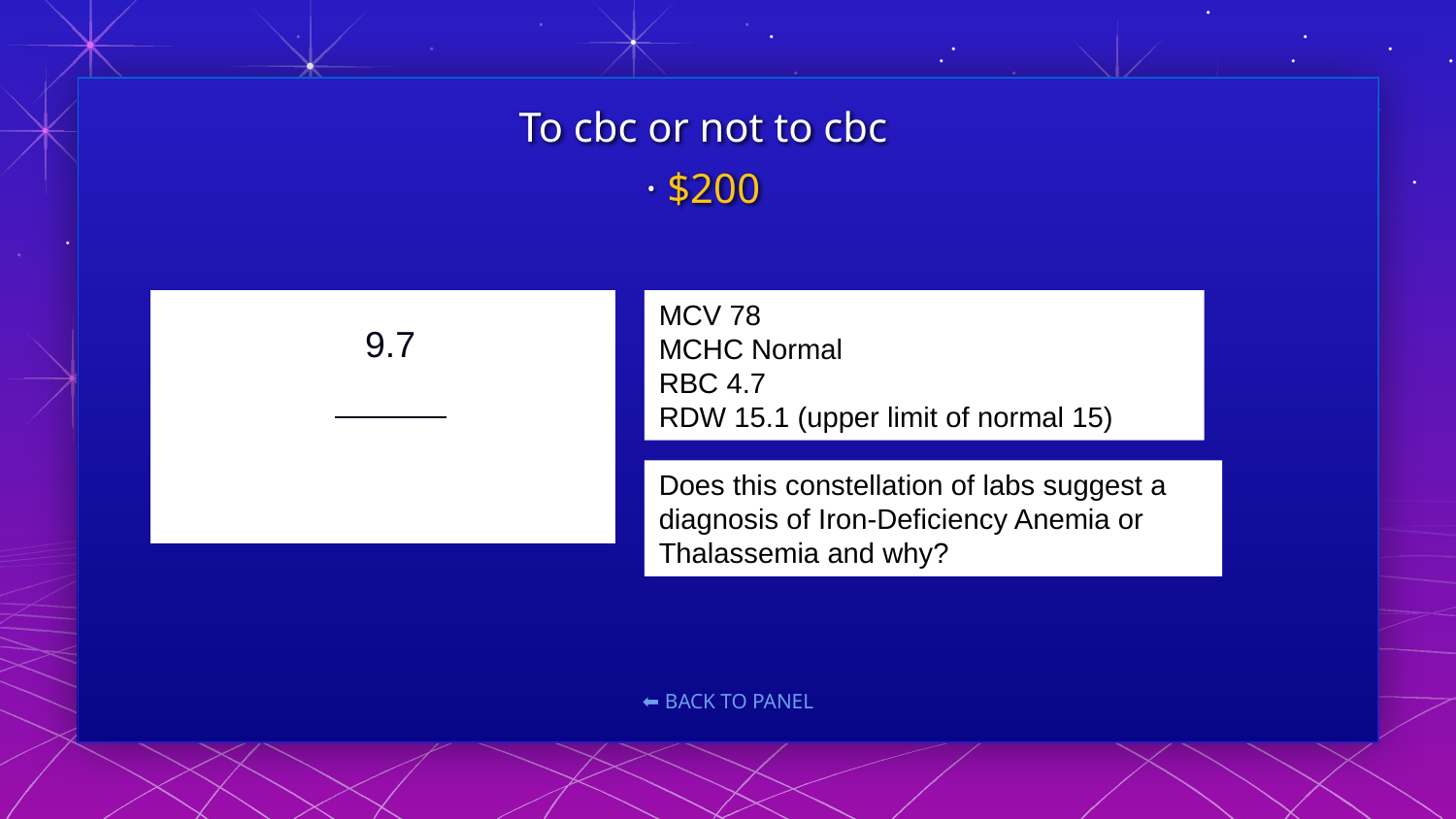

To cbc or not to cbc
· $200
#
| | | 9.7 | | |
| --- | --- | --- | --- | --- |
| | | | | |
MCV 78
MCHC Normal
RBC 4.7
RDW 15.1 (upper limit of normal 15)
Does this constellation of labs suggest a diagnosis of Iron-Deficiency Anemia or Thalassemia and why?

## Slide 5
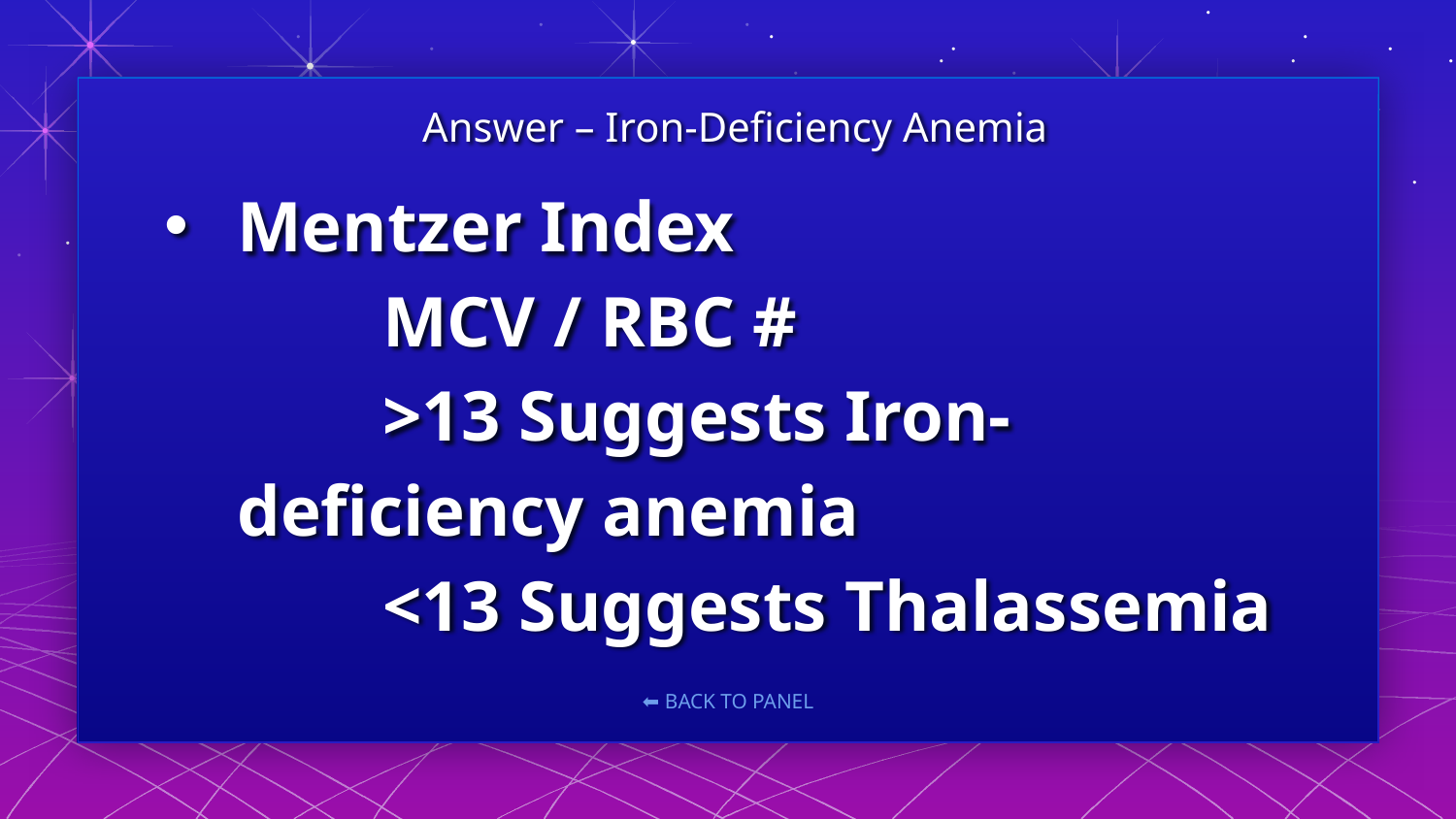

Answer – Iron-Deficiency Anemia
# Mentzer Index MCV / RBC # >13 Suggests Iron-deficiency anemia <13 Suggests Thalassemia

## Slide 6
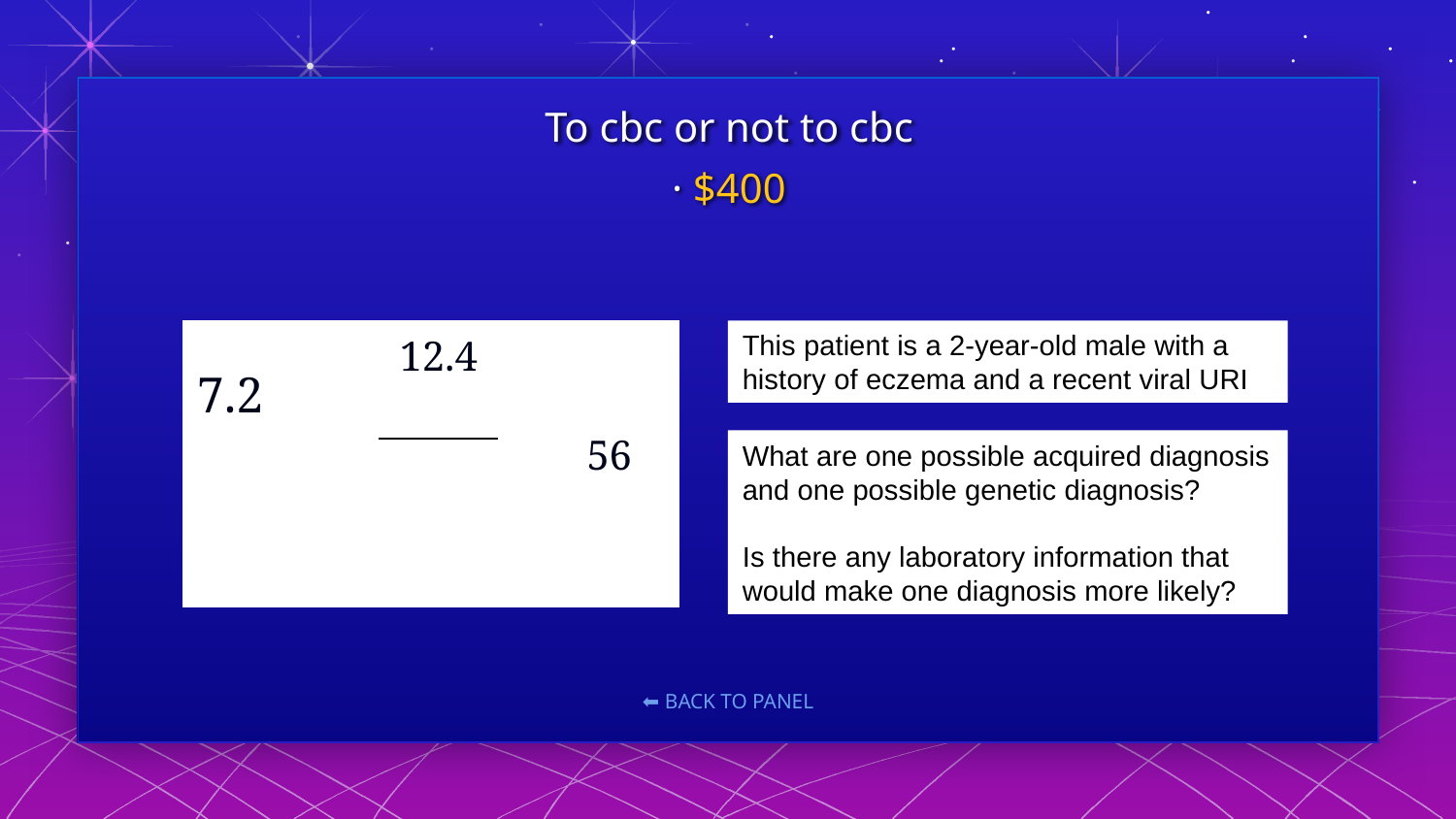

To cbc or not to cbc
· $400
| 7.2 | | 12.4 | | 56 |
| --- | --- | --- | --- | --- |
| | | | | |
This patient is a 2-year-old male with a history of eczema and a recent viral URI
What are one possible acquired diagnosis and one possible genetic diagnosis?
Is there any laboratory information that would make one diagnosis more likely?

## Slide 7
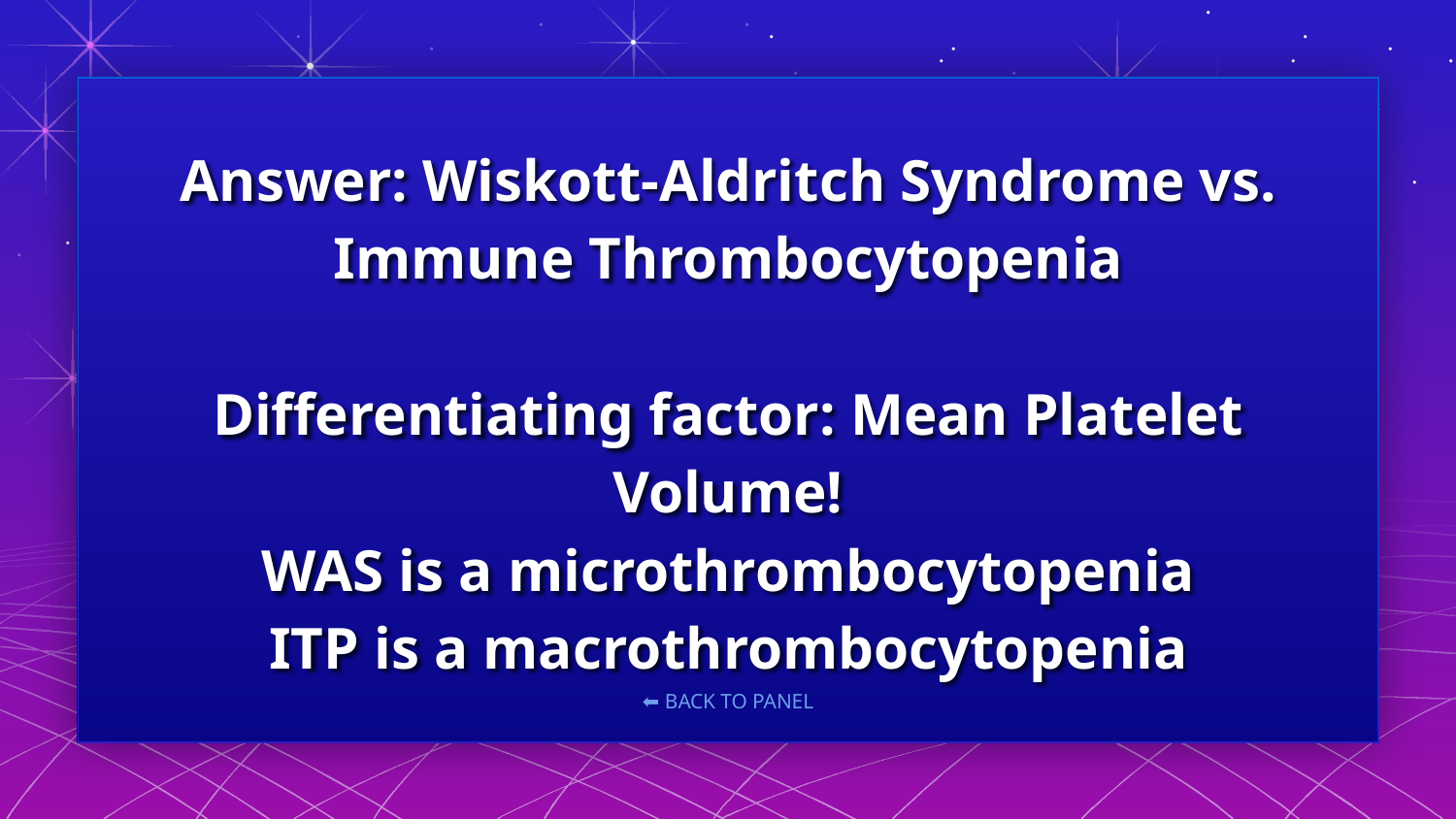

# Answer: Wiskott-Aldritch Syndrome vs. Immune Thrombocytopenia Differentiating factor: Mean Platelet Volume! WAS is a microthrombocytopenia ITP is a macrothrombocytopenia

## Slide 8
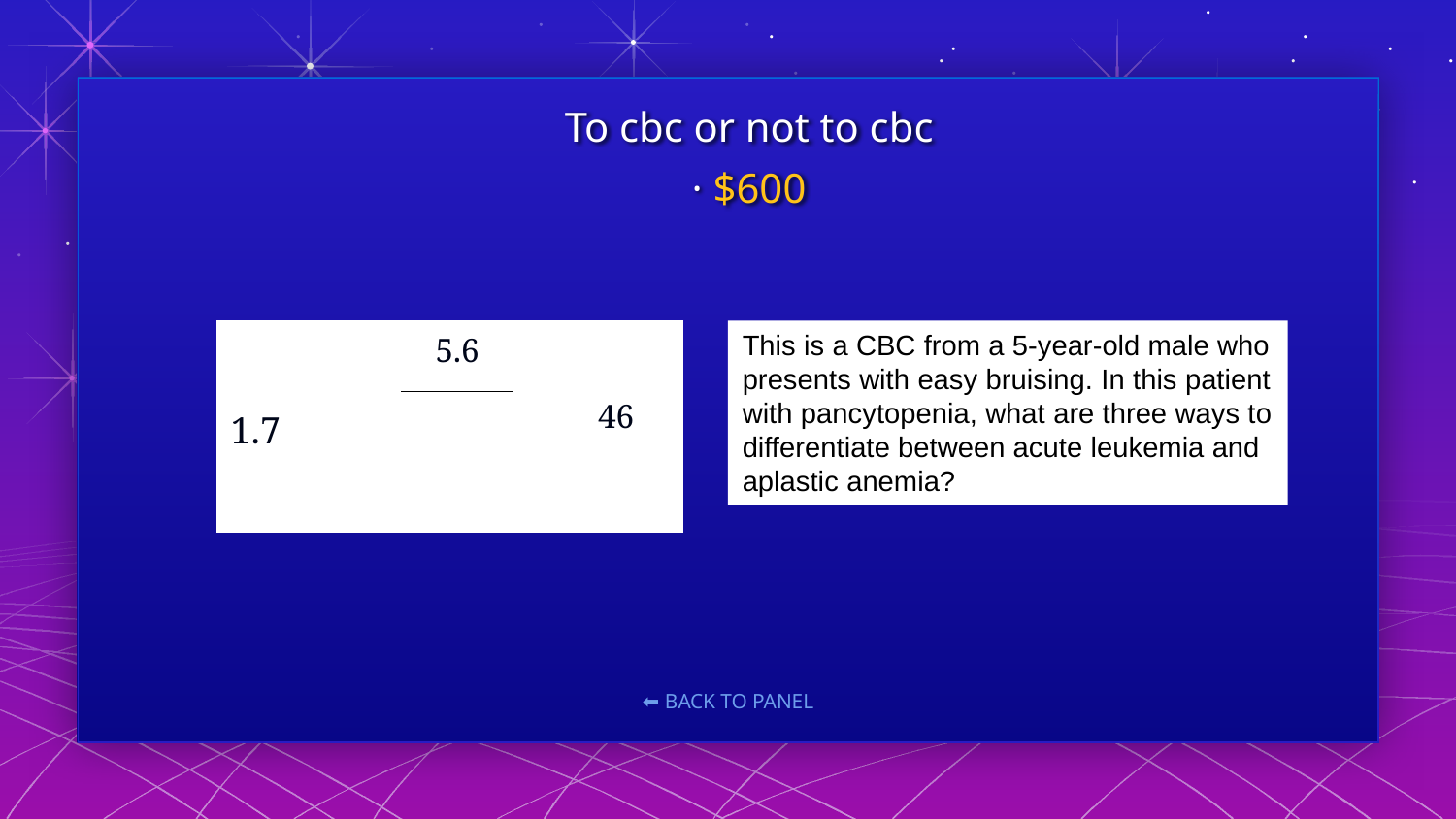

To cbc or not to cbc
· $600
| 1.7 | | 5.6 | | 46 |
| --- | --- | --- | --- | --- |
| | | | | |
This is a CBC from a 5-year-old male who presents with easy bruising. In this patient with pancytopenia, what are three ways to differentiate between acute leukemia and aplastic anemia?

## Slide 9
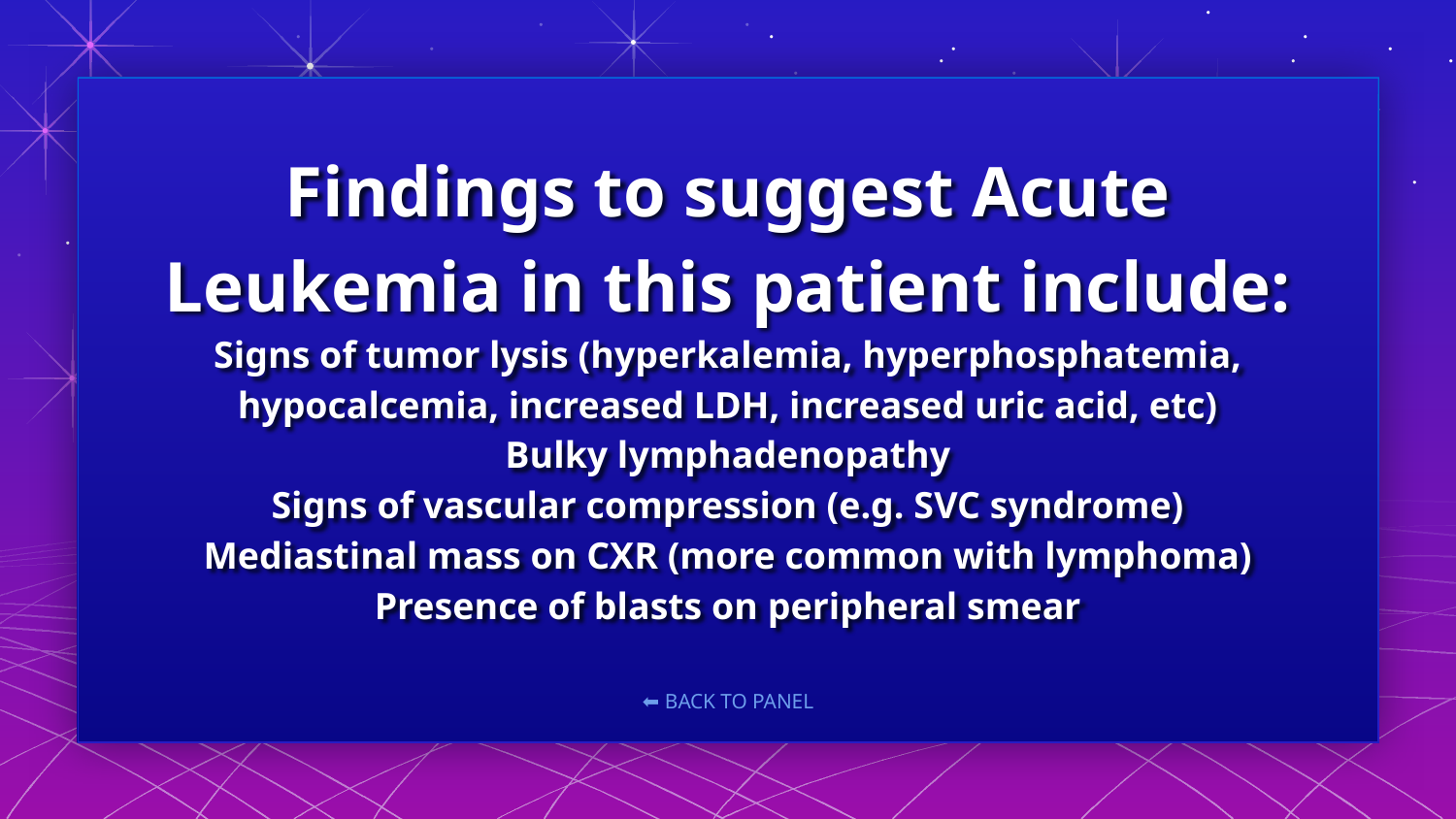

# Findings to suggest Acute Leukemia in this patient include: Signs of tumor lysis (hyperkalemia, hyperphosphatemia, hypocalcemia, increased LDH, increased uric acid, etc) Bulky lymphadenopathy Signs of vascular compression (e.g. SVC syndrome) Mediastinal mass on CXR (more common with lymphoma) Presence of blasts on peripheral smear

## Slide 10
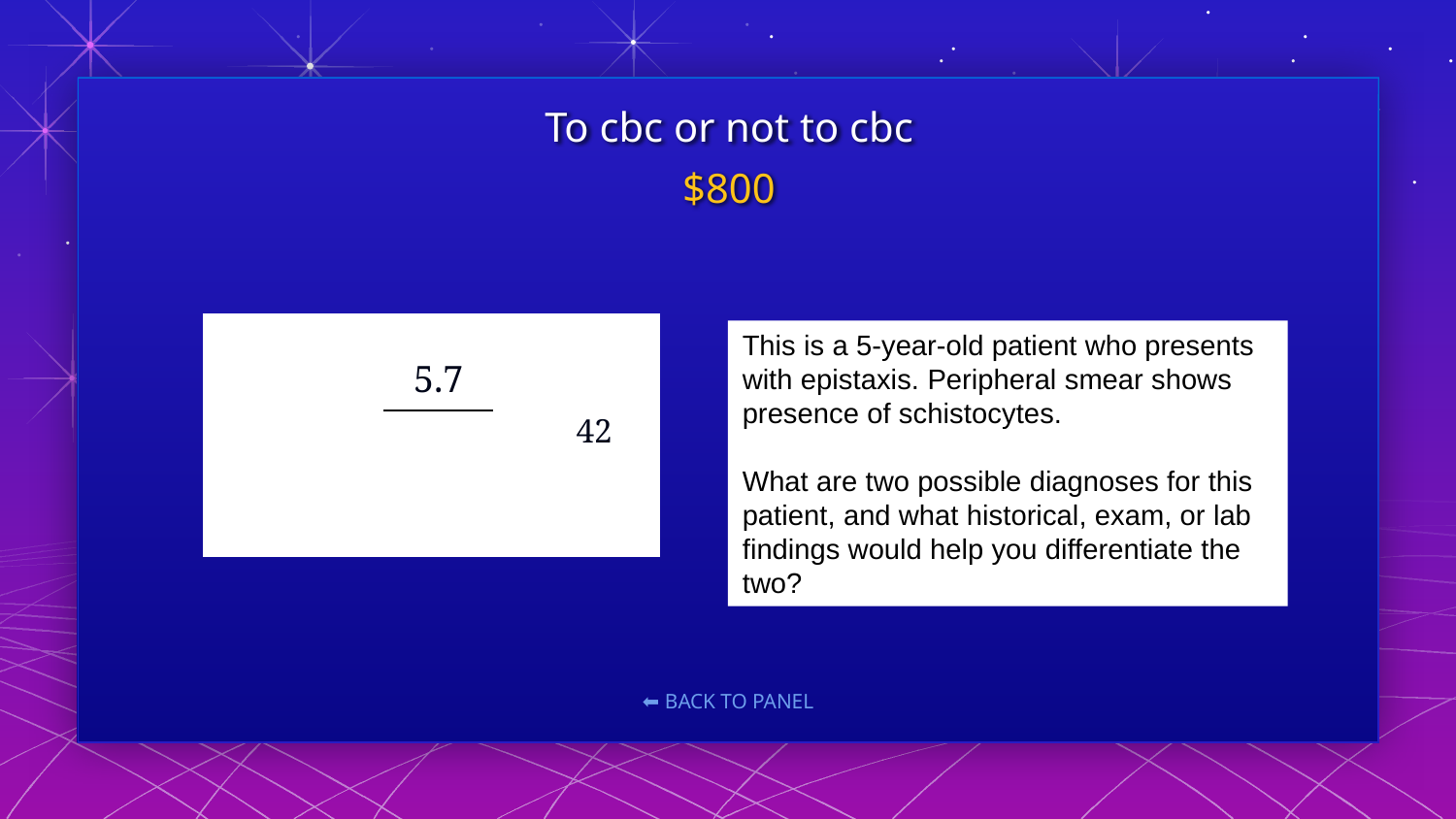

To cbc or not to cbc
$800
| | | 5.7 | | 42 |
| --- | --- | --- | --- | --- |
| | | | | |
This is a 5-year-old patient who presents with epistaxis. Peripheral smear shows presence of schistocytes.
What are two possible diagnoses for this patient, and what historical, exam, or lab findings would help you differentiate the two?

## Slide 11
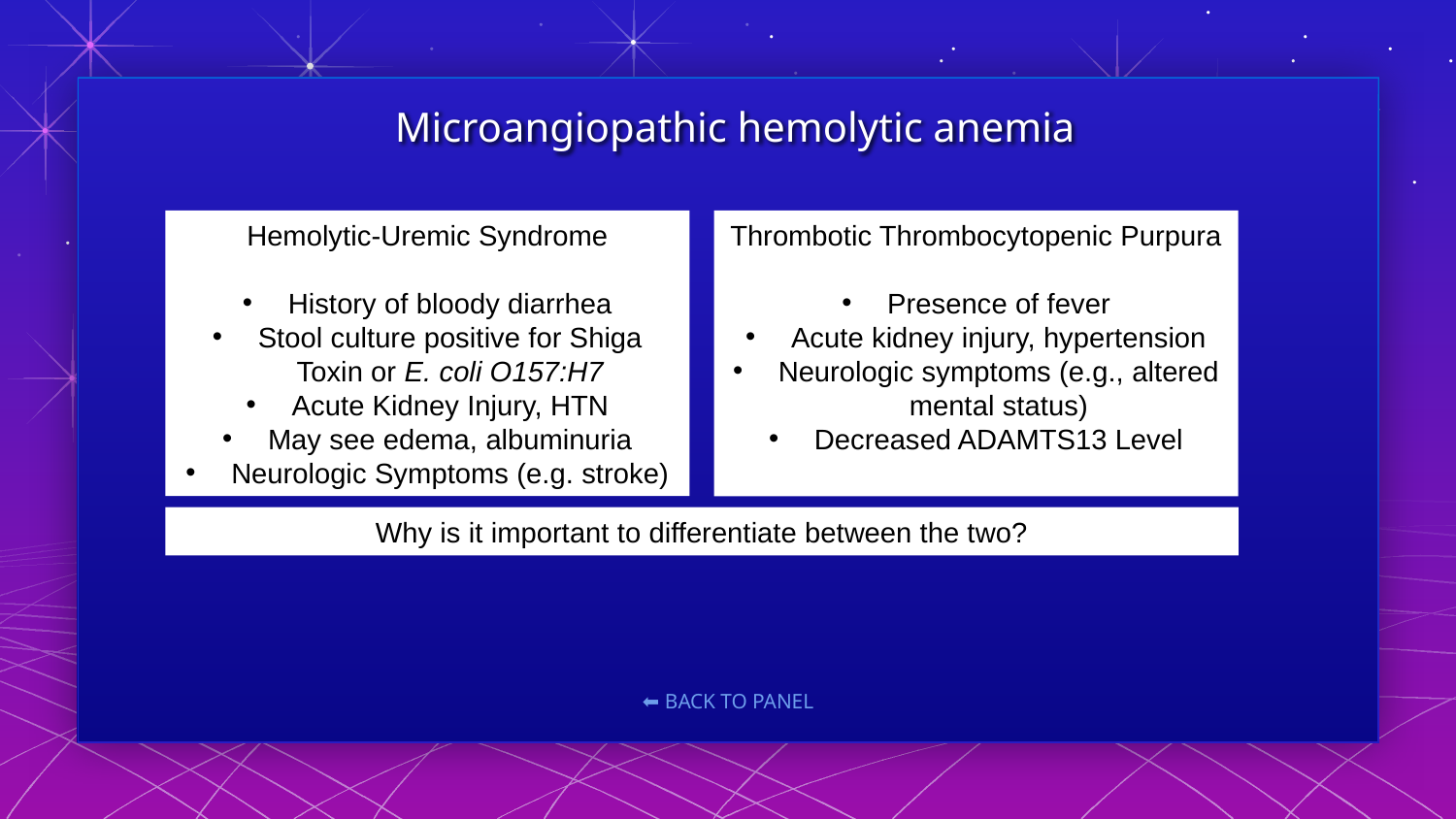

Microangiopathic hemolytic anemia
Hemolytic-Uremic Syndrome
History of bloody diarrhea
Stool culture positive for Shiga Toxin or E. coli O157:H7
Acute Kidney Injury, HTN
May see edema, albuminuria
Neurologic Symptoms (e.g. stroke)
Thrombotic Thrombocytopenic Purpura
Presence of fever
Acute kidney injury, hypertension
Neurologic symptoms (e.g., altered mental status)
Decreased ADAMTS13 Level
Why is it important to differentiate between the two?

## Slide 12
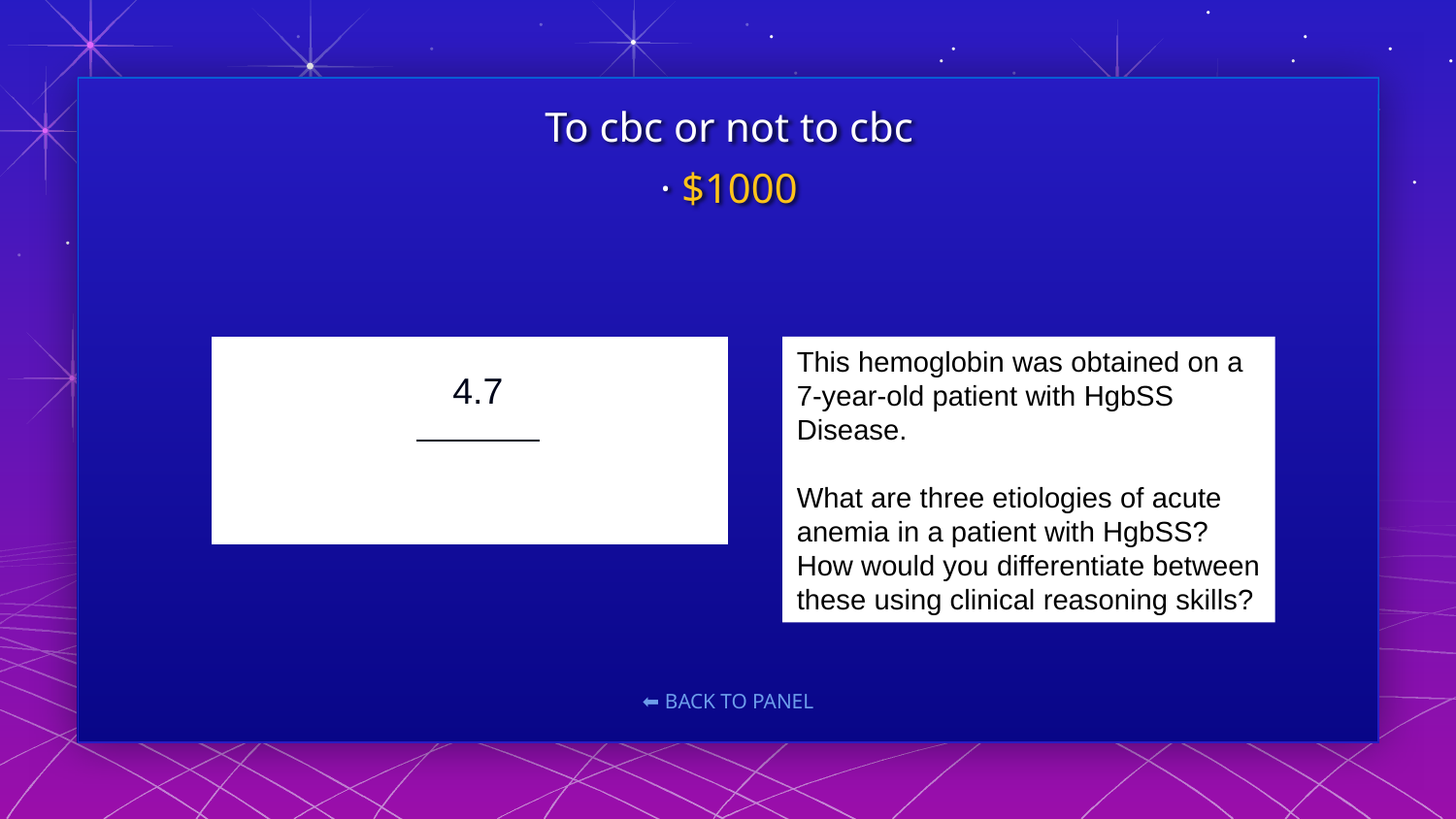

To cbc or not to cbc
· $1000
| | | 4.7 | | |
| --- | --- | --- | --- | --- |
| | | | | |
This hemoglobin was obtained on a 7-year-old patient with HgbSS Disease.
What are three etiologies of acute anemia in a patient with HgbSS? How would you differentiate between these using clinical reasoning skills?

## Slide 13
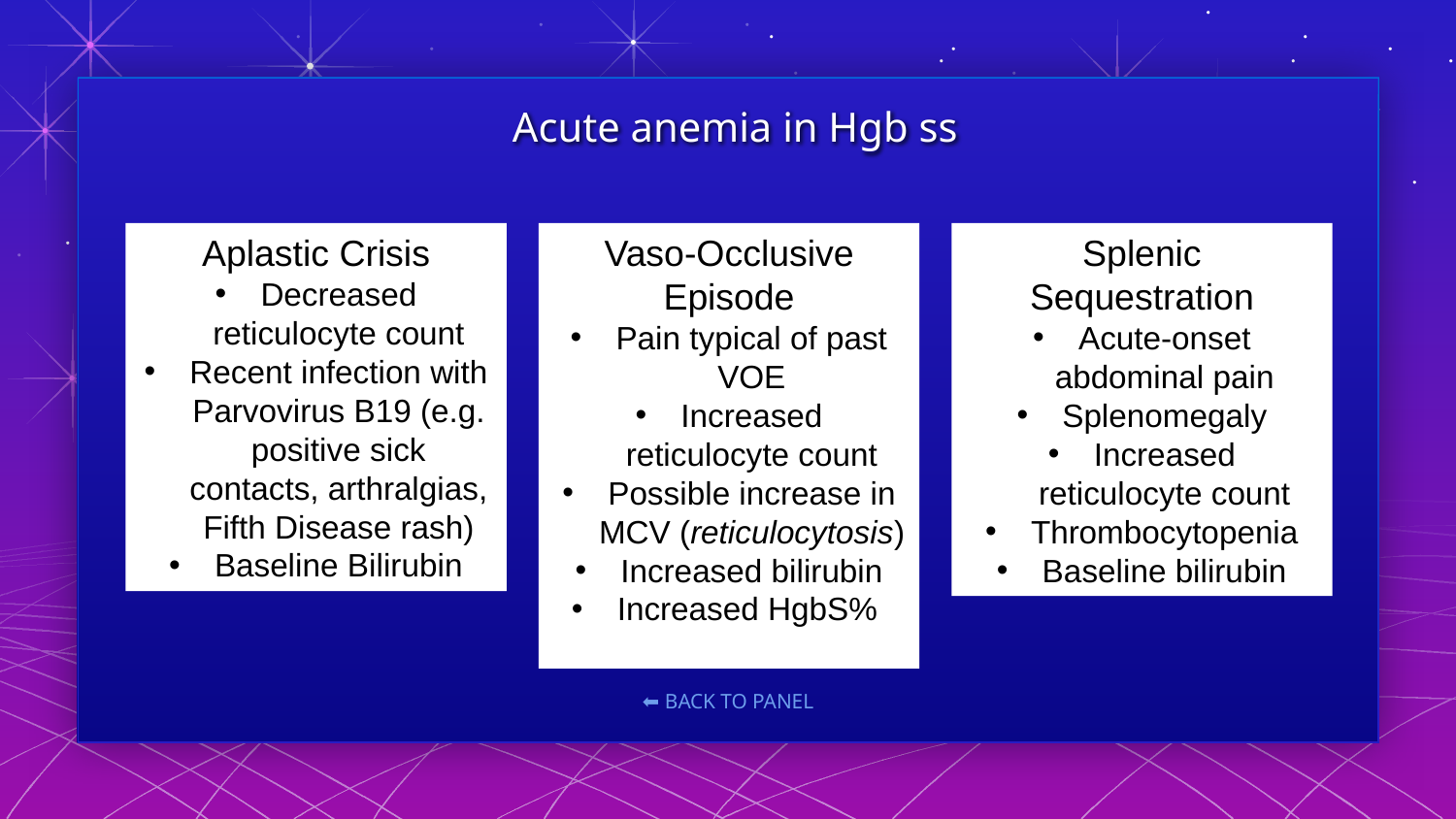

Acute anemia in Hgb ss
Aplastic Crisis
Decreased reticulocyte count
Recent infection with Parvovirus B19 (e.g. positive sick contacts, arthralgias, Fifth Disease rash)
Baseline Bilirubin
Vaso-Occlusive Episode
Pain typical of past VOE
Increased reticulocyte count
Possible increase in MCV (reticulocytosis)
Increased bilirubin
Increased HgbS%
Splenic Sequestration
Acute-onset abdominal pain
Splenomegaly
Increased reticulocyte count
Thrombocytopenia
Baseline bilirubin

## Slide 14
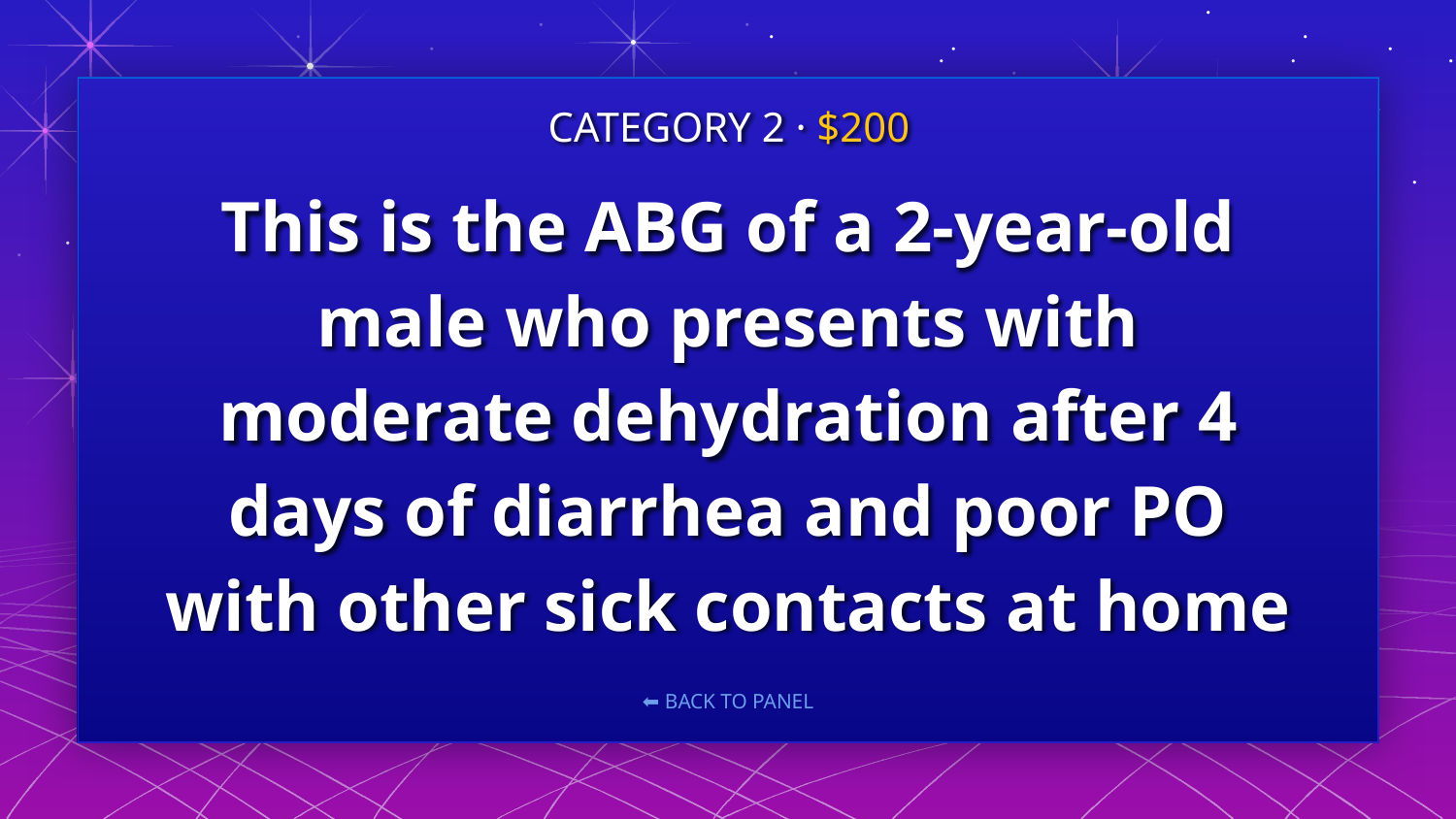

CATEGORY 2 · $200
# This is the ABG of a 2-year-old male who presents with moderate dehydration after 4 days of diarrhea and poor PO with other sick contacts at home

## Slide 15
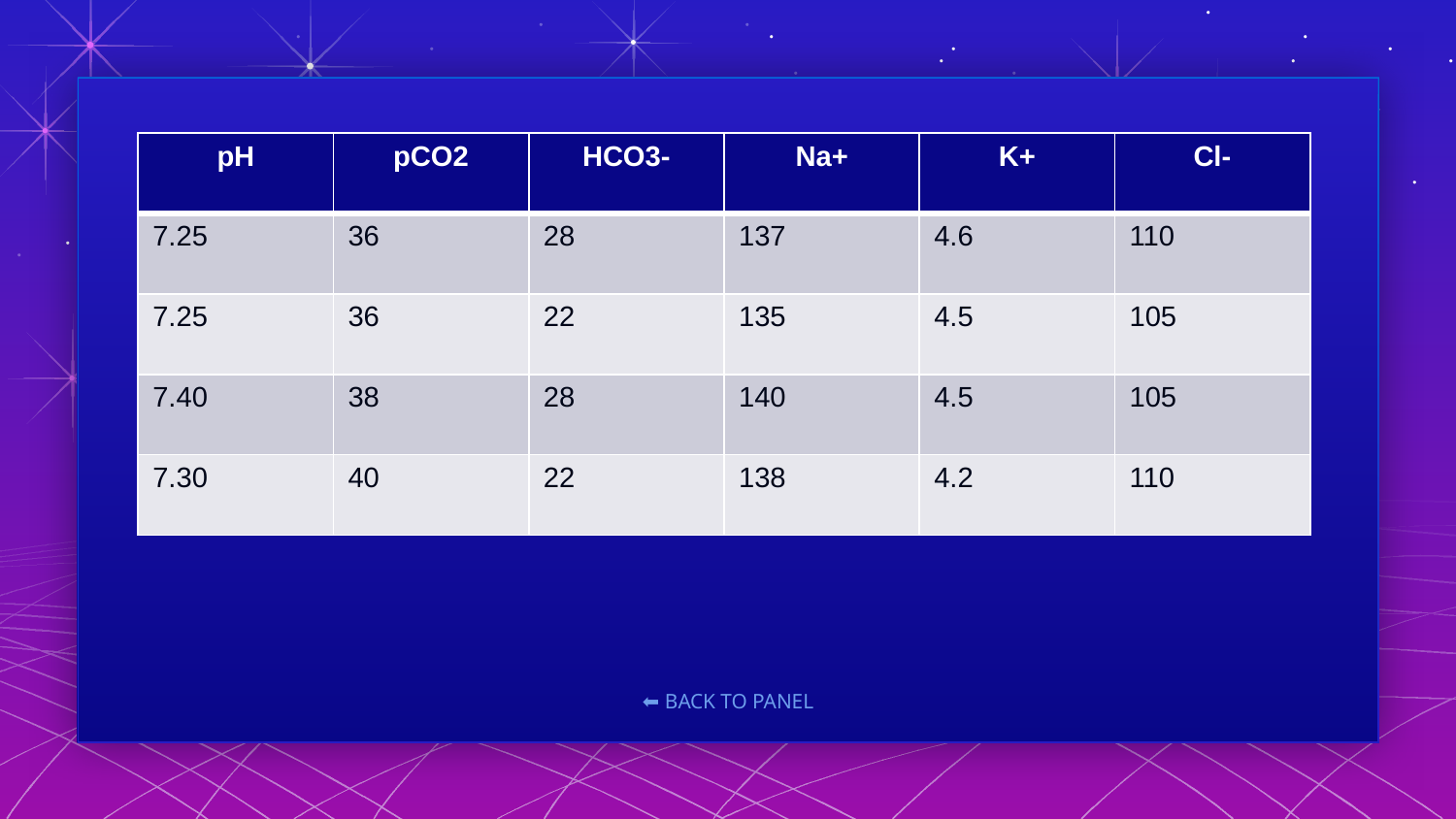

| pH | pCO2 | HCO3- | Na+ | K+ | Cl- |
| --- | --- | --- | --- | --- | --- |
| 7.25 | 36 | 28 | 137 | 4.6 | 110 |
| 7.25 | 36 | 22 | 135 | 4.5 | 105 |
| 7.40 | 38 | 28 | 140 | 4.5 | 105 |
| 7.30 | 40 | 22 | 138 | 4.2 | 110 |

## Slide 16
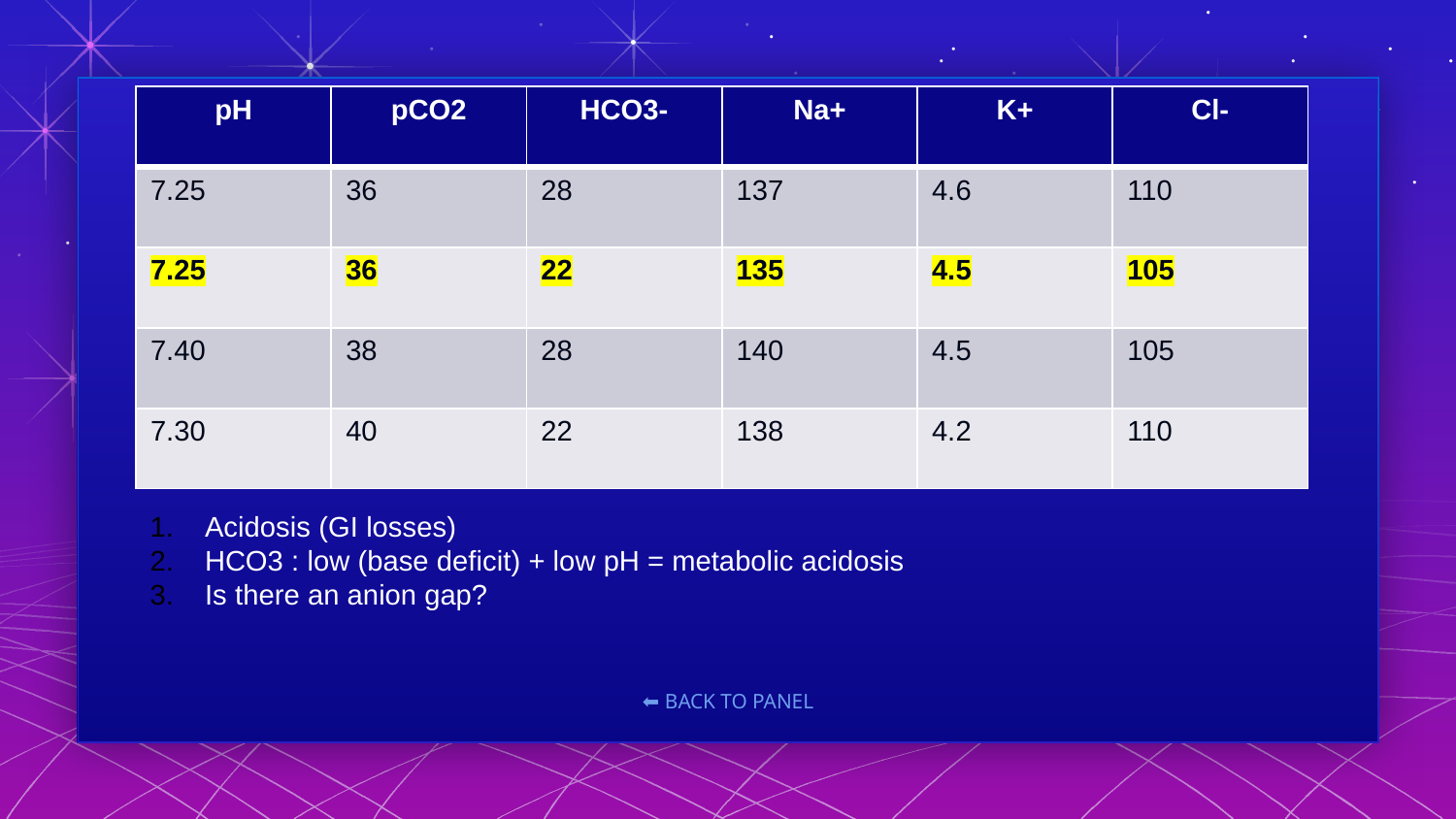

| pH | pCO2 | HCO3- | Na+ | K+ | Cl- |
| --- | --- | --- | --- | --- | --- |
| 7.25 | 36 | 28 | 137 | 4.6 | 110 |
| 7.25 | 36 | 22 | 135 | 4.5 | 105 |
| 7.40 | 38 | 28 | 140 | 4.5 | 105 |
| 7.30 | 40 | 22 | 138 | 4.2 | 110 |
Acidosis (GI losses)
HCO3 : low (base deficit) + low pH = metabolic acidosis
Is there an anion gap?

## Slide 17
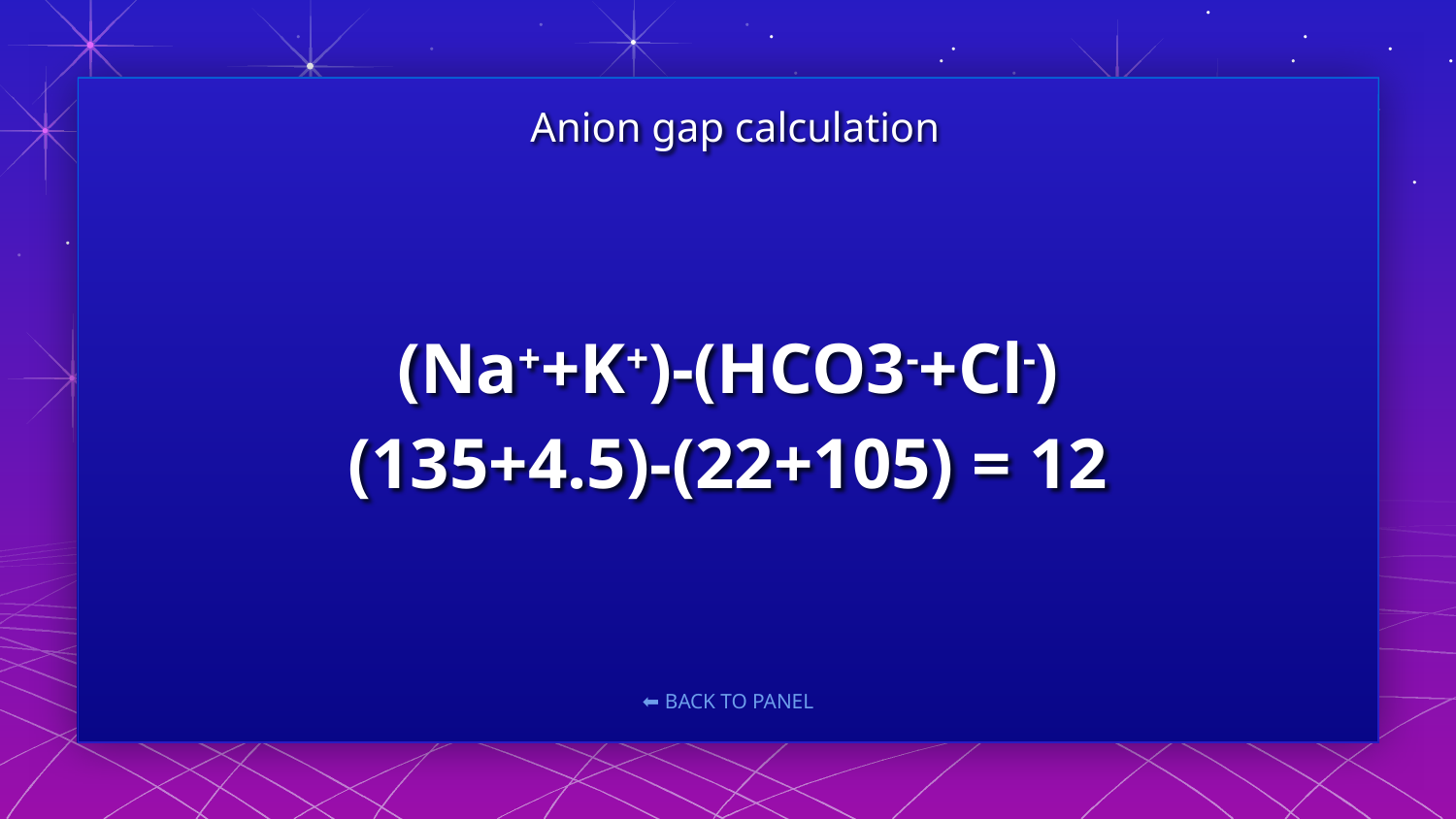

Anion gap calculation
# (Na++K+)-(HCO3-+Cl-) (135+4.5)-(22+105) = 12

## Slide 18
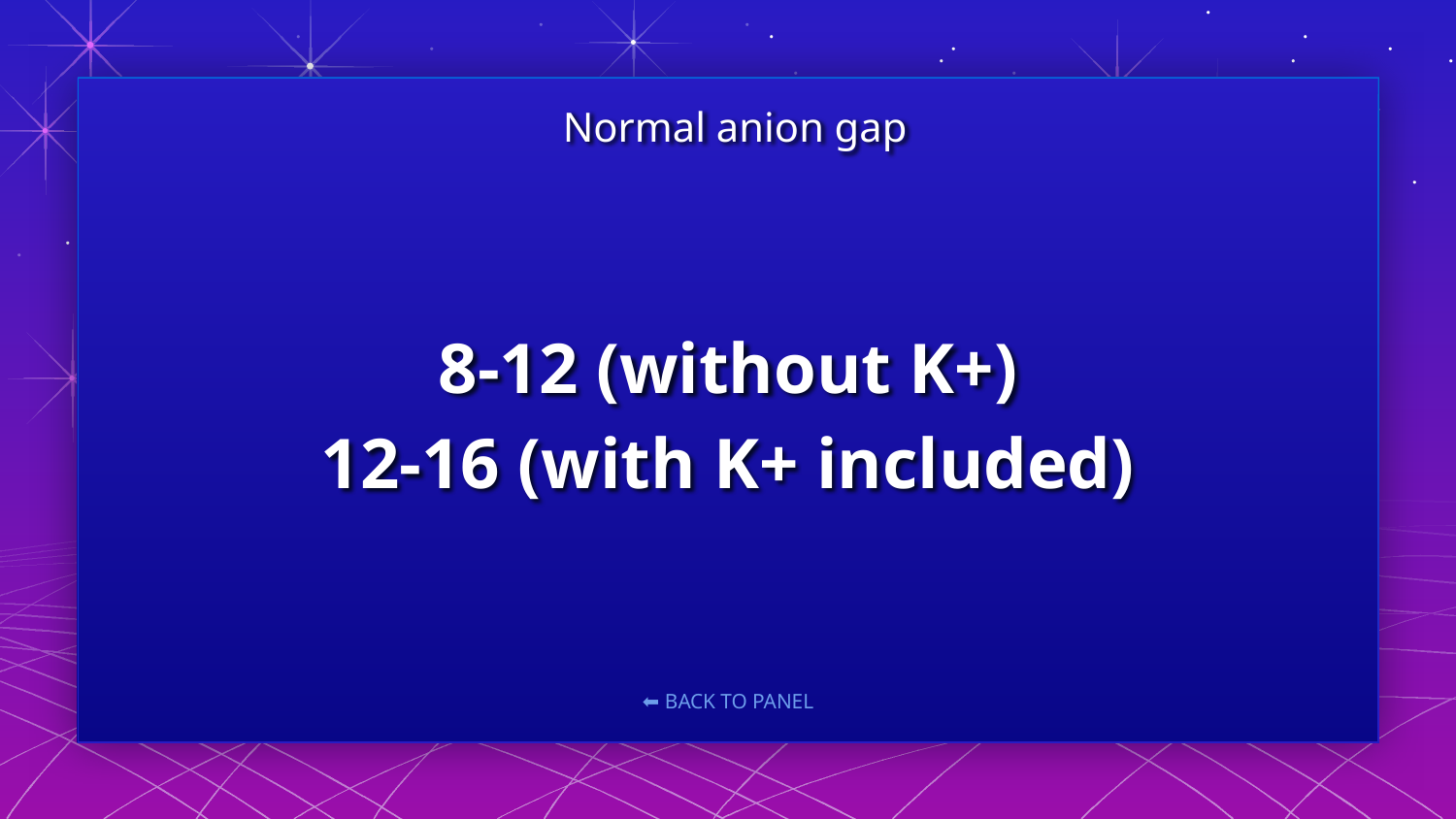

Normal anion gap
# 8-12 (without K+) 12-16 (with K+ included)

## Slide 19
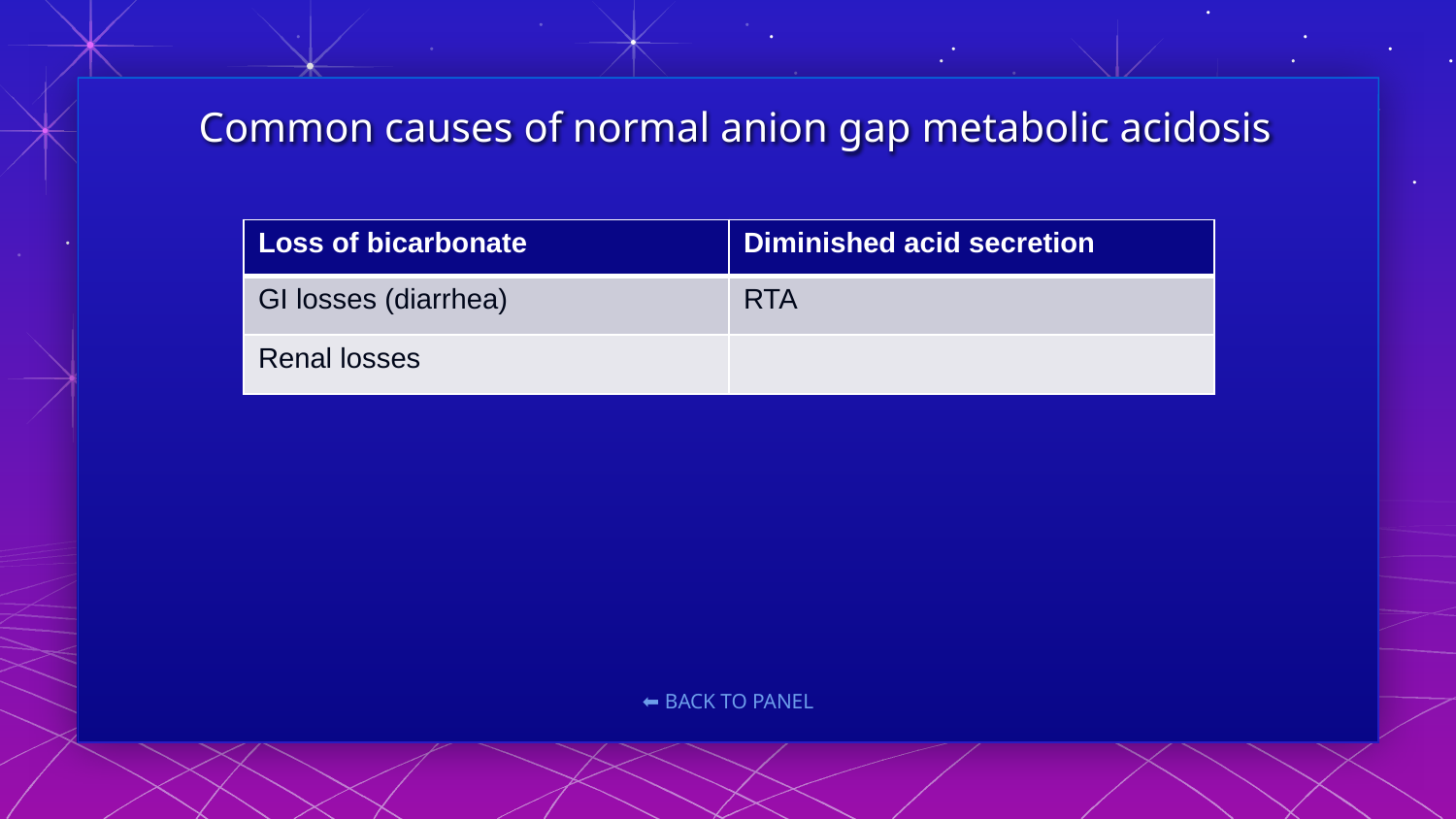

Common causes of normal anion gap metabolic acidosis
#
| Loss of bicarbonate | Diminished acid secretion |
| --- | --- |
| GI losses (diarrhea) | RTA |
| Renal losses | |

## Slide 20
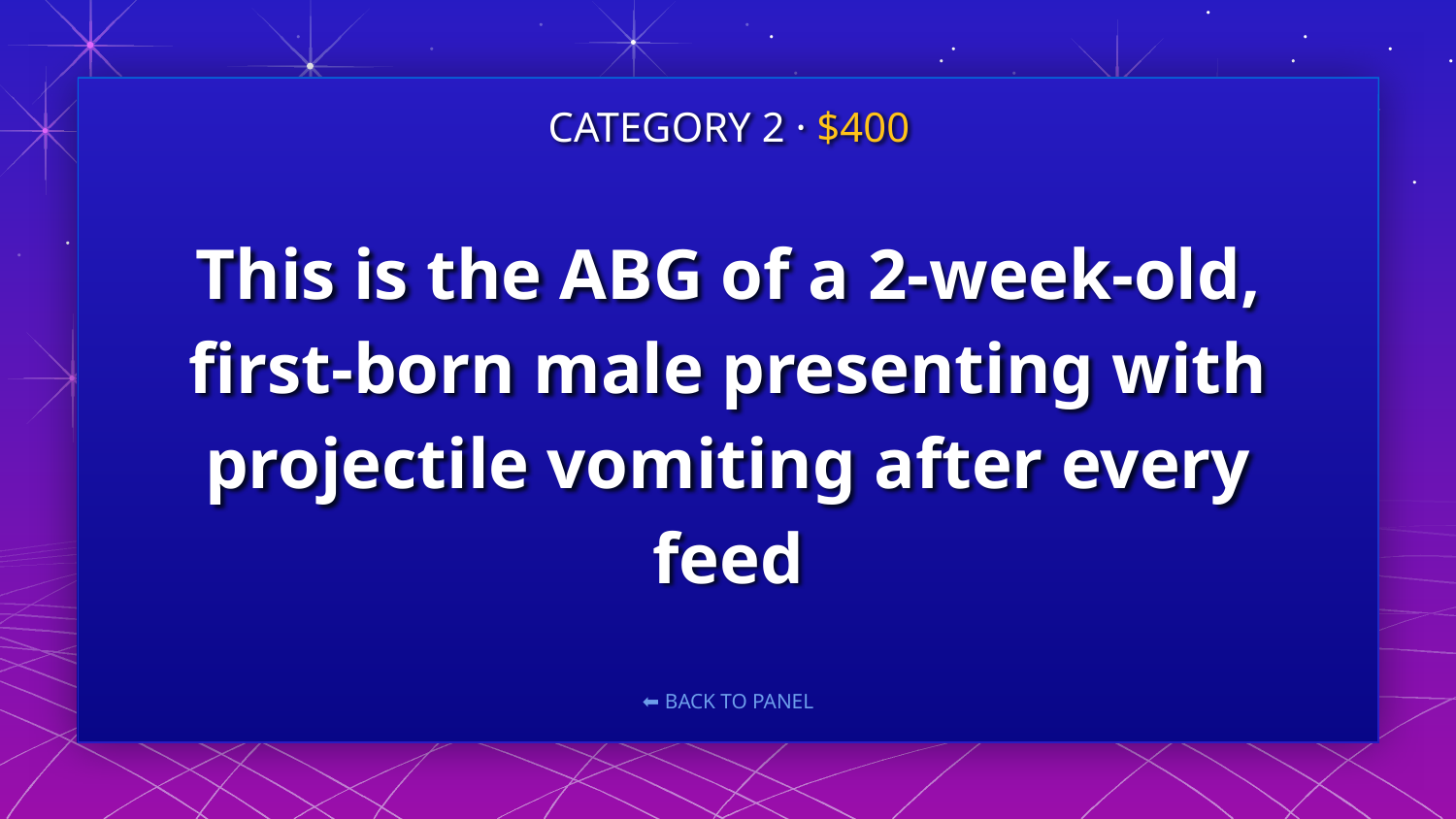

CATEGORY 2 · $400
# This is the ABG of a 2-week-old, first-born male presenting with projectile vomiting after every feed

## Slide 21
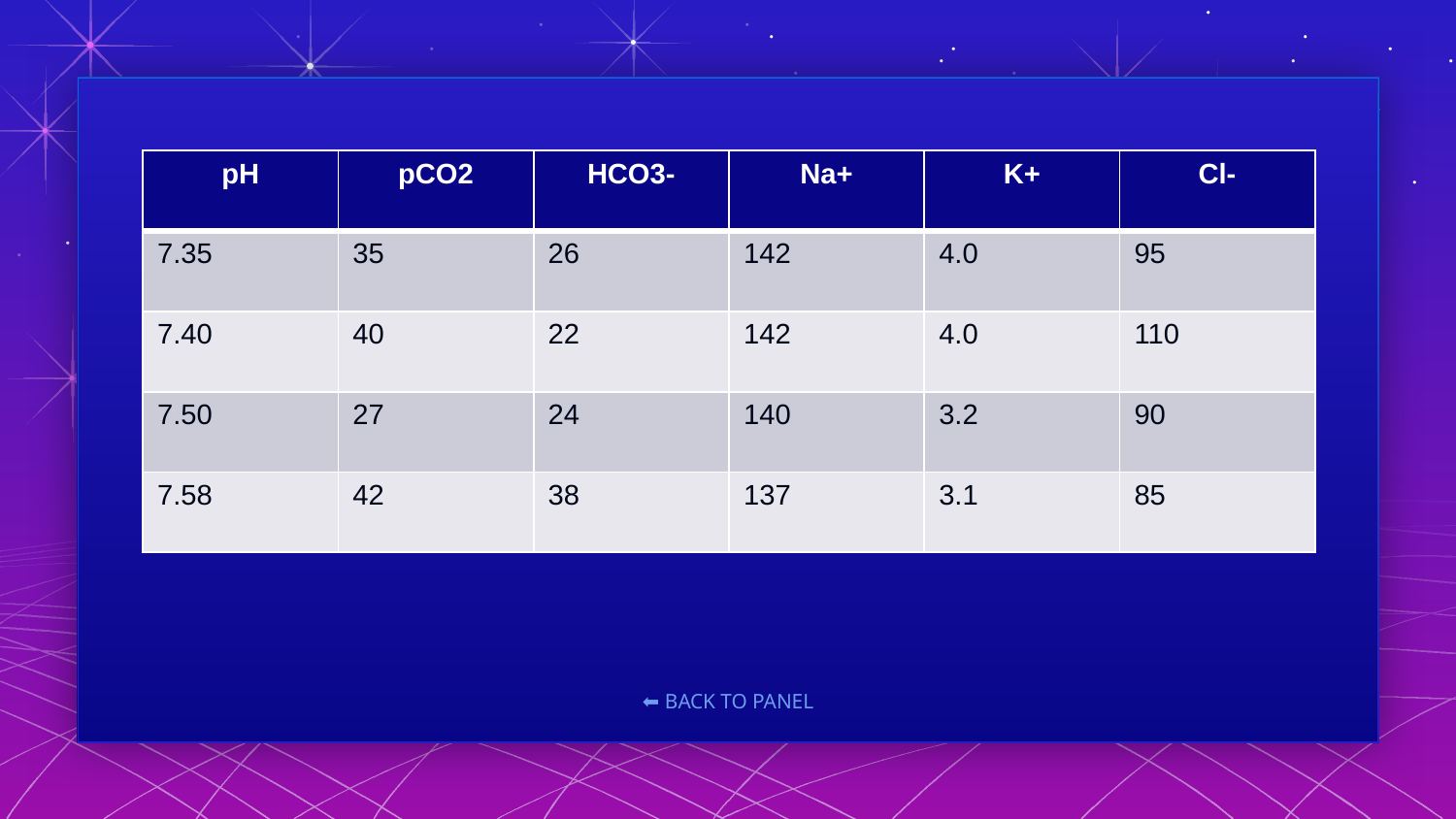

| pH | pCO2 | HCO3- | Na+ | K+ | Cl- |
| --- | --- | --- | --- | --- | --- |
| 7.35 | 35 | 26 | 142 | 4.0 | 95 |
| 7.40 | 40 | 22 | 142 | 4.0 | 110 |
| 7.50 | 27 | 24 | 140 | 3.2 | 90 |
| 7.58 | 42 | 38 | 137 | 3.1 | 85 |
#

## Slide 22
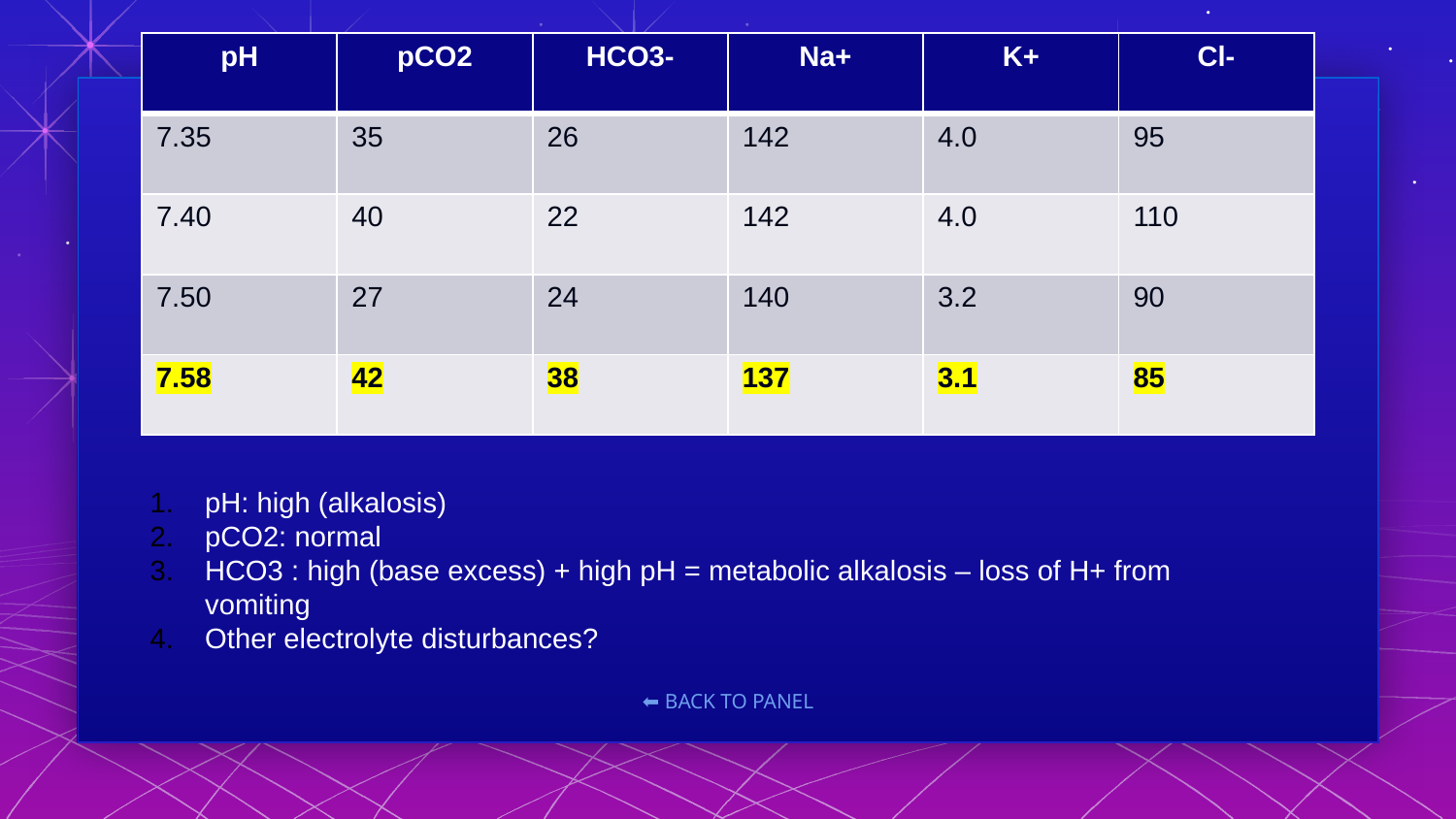

| pH | pCO2 | HCO3- | Na+ | K+ | Cl- |
| --- | --- | --- | --- | --- | --- |
| 7.35 | 35 | 26 | 142 | 4.0 | 95 |
| 7.40 | 40 | 22 | 142 | 4.0 | 110 |
| 7.50 | 27 | 24 | 140 | 3.2 | 90 |
| 7.58 | 42 | 38 | 137 | 3.1 | 85 |
pH: high (alkalosis)
pCO2: normal
HCO3 : high (base excess) + high pH = metabolic alkalosis – loss of H+ from vomiting
Other electrolyte disturbances?

## Slide 23
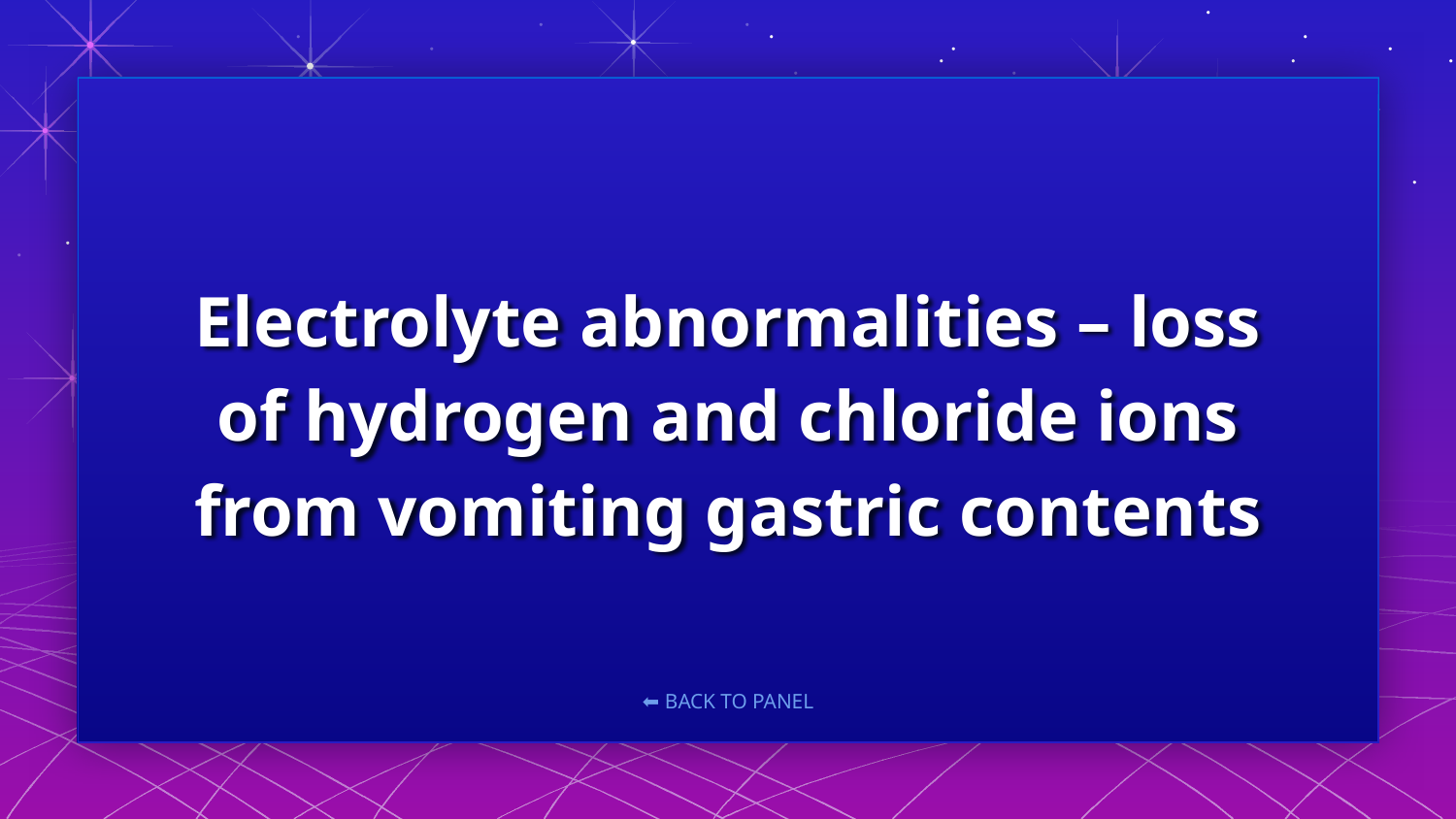

# Electrolyte abnormalities – loss of hydrogen and chloride ions from vomiting gastric contents

## Slide 24
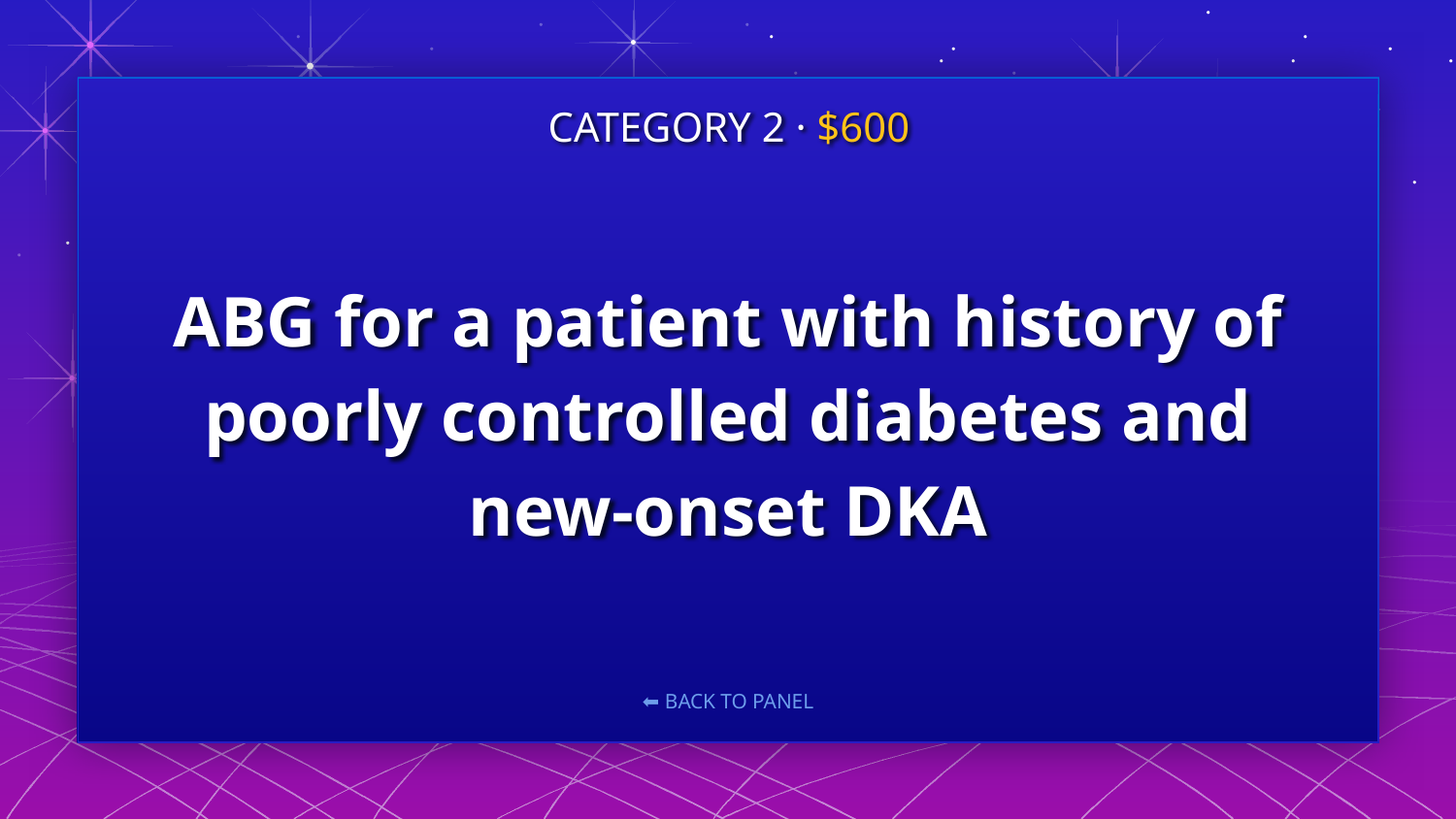

CATEGORY 2 · $600
# ABG for a patient with history of poorly controlled diabetes and new-onset DKA

## Slide 25
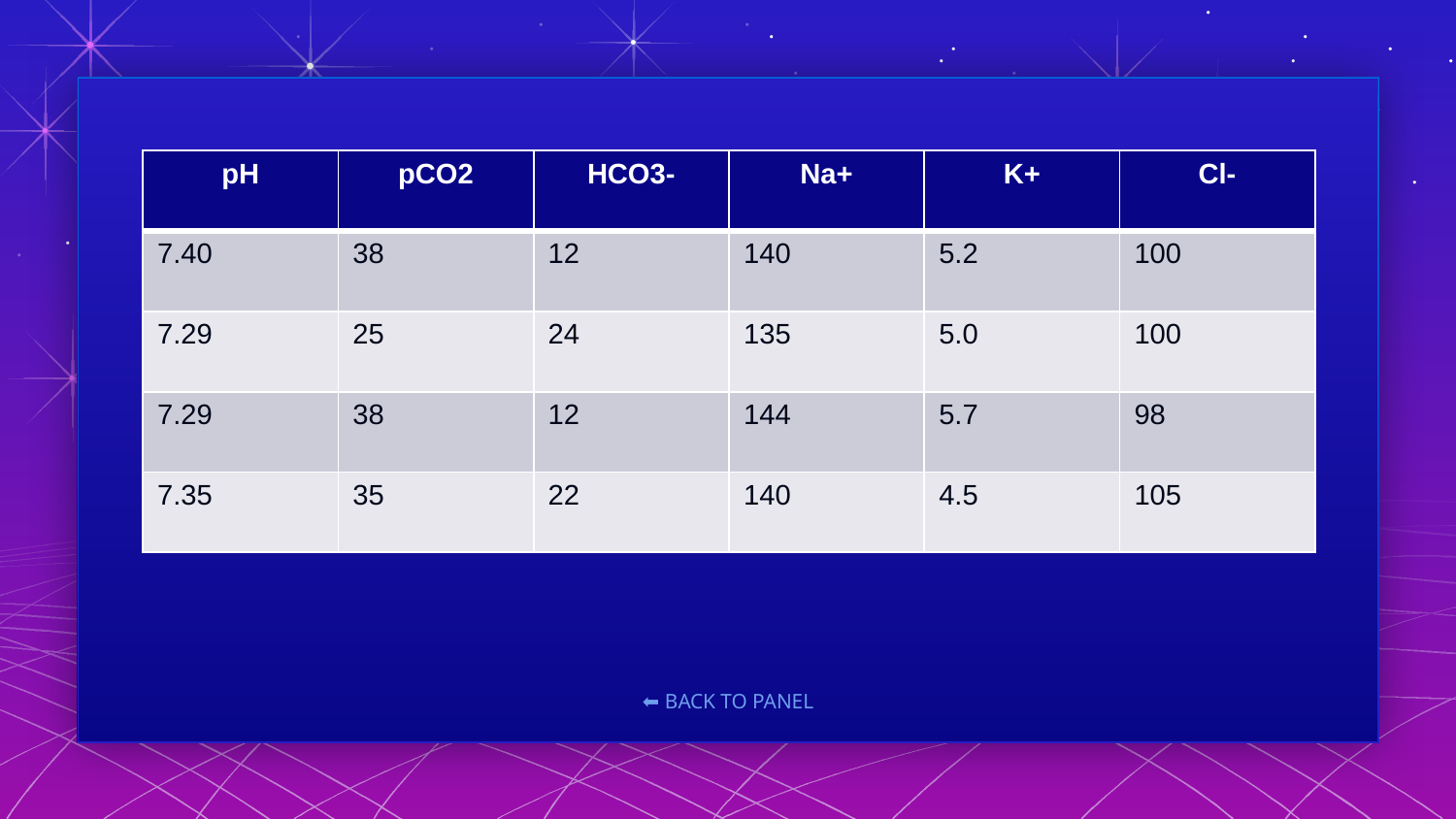

| pH | pCO2 | HCO3- | Na+ | K+ | Cl- |
| --- | --- | --- | --- | --- | --- |
| 7.40 | 38 | 12 | 140 | 5.2 | 100 |
| 7.29 | 25 | 24 | 135 | 5.0 | 100 |
| 7.29 | 38 | 12 | 144 | 5.7 | 98 |
| 7.35 | 35 | 22 | 140 | 4.5 | 105 |
#

## Slide 26
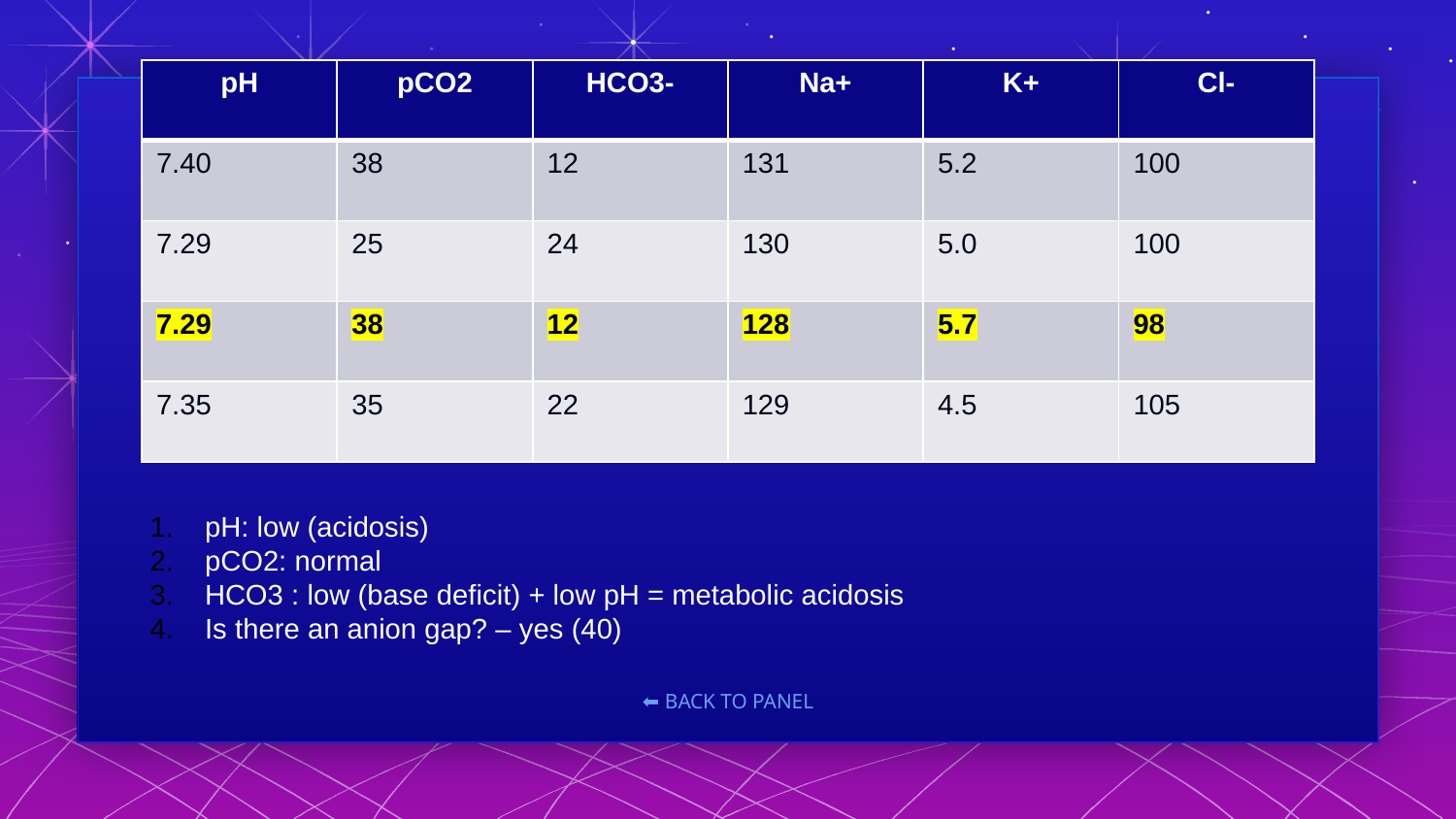

| pH | pCO2 | HCO3- | Na+ | K+ | Cl- |
| --- | --- | --- | --- | --- | --- |
| 7.40 | 38 | 12 | 131 | 5.2 | 100 |
| 7.29 | 25 | 24 | 130 | 5.0 | 100 |
| 7.29 | 38 | 12 | 128 | 5.7 | 98 |
| 7.35 | 35 | 22 | 129 | 4.5 | 105 |
pH: low (acidosis)
pCO2: normal
HCO3 : low (base deficit) + low pH = metabolic acidosis
Is there an anion gap? – yes (40)

## Slide 27
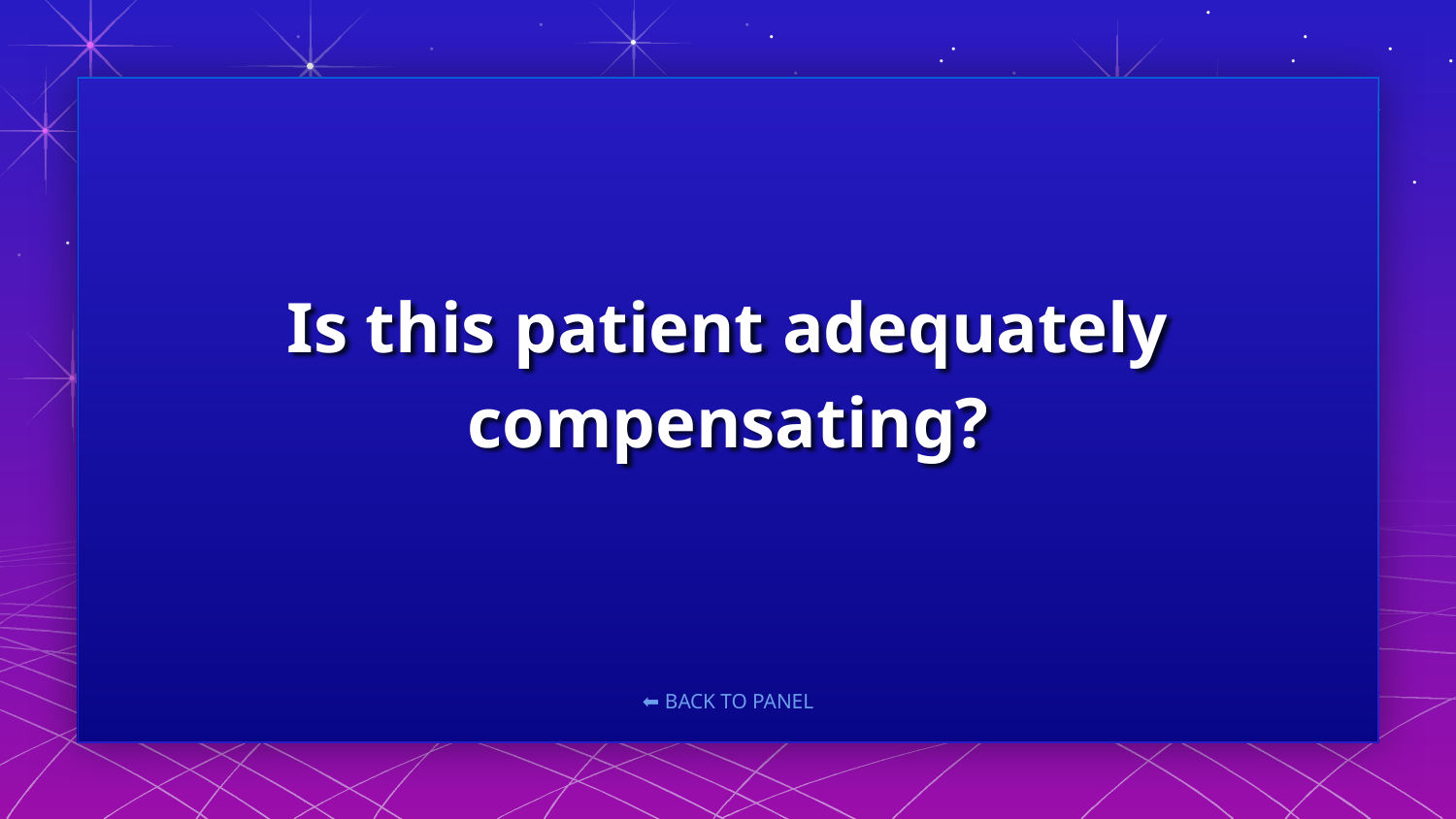

# Is this patient adequately compensating?

## Slide 28
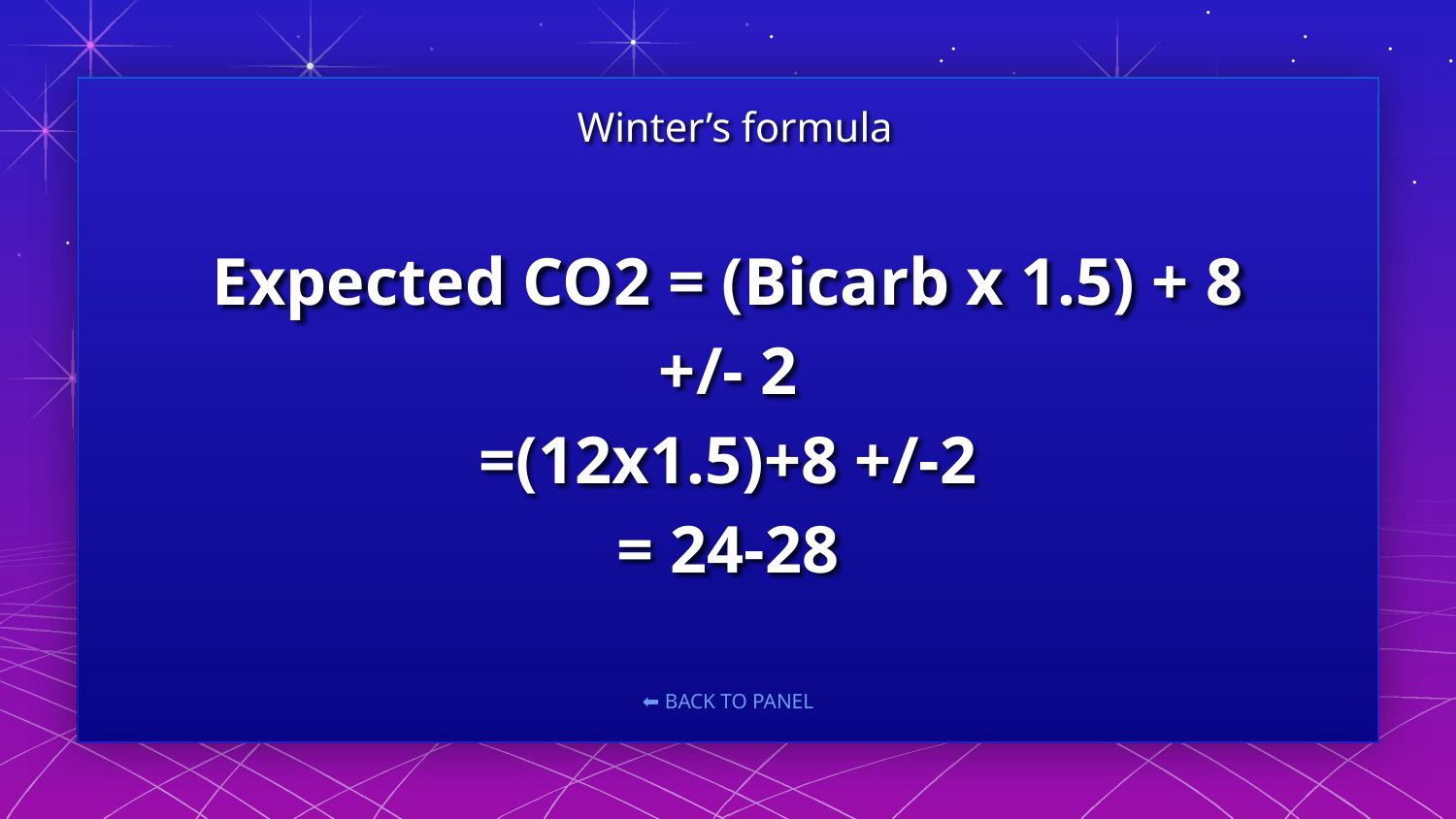

Winter’s formula
# Expected CO2 = (Bicarb x 1.5) + 8 +/- 2 =(12x1.5)+8 +/-2 = 24-28

## Slide 29
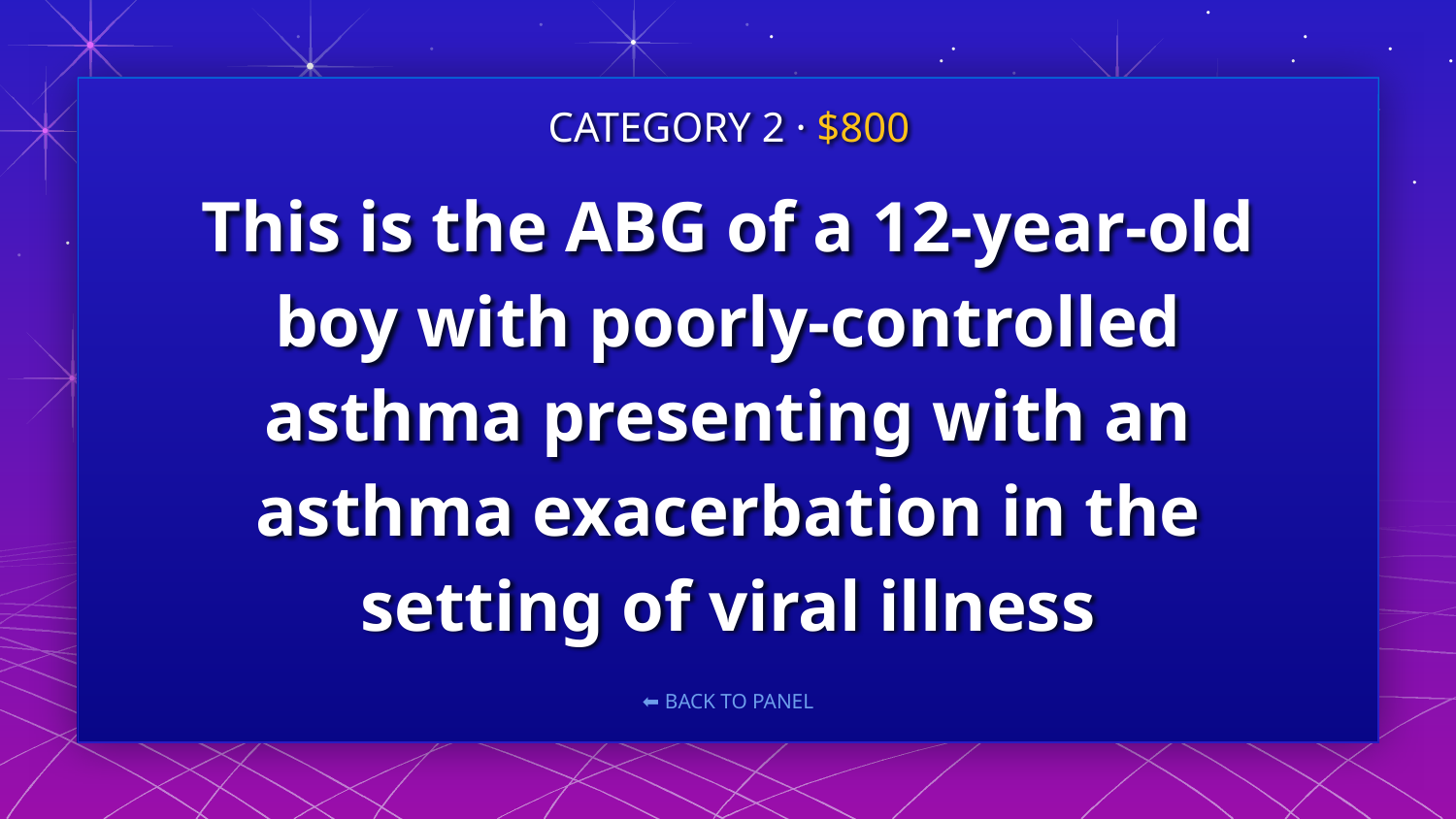

CATEGORY 2 · $800
# This is the ABG of a 12-year-old boy with poorly-controlled asthma presenting with an asthma exacerbation in the setting of viral illness

## Slide 30
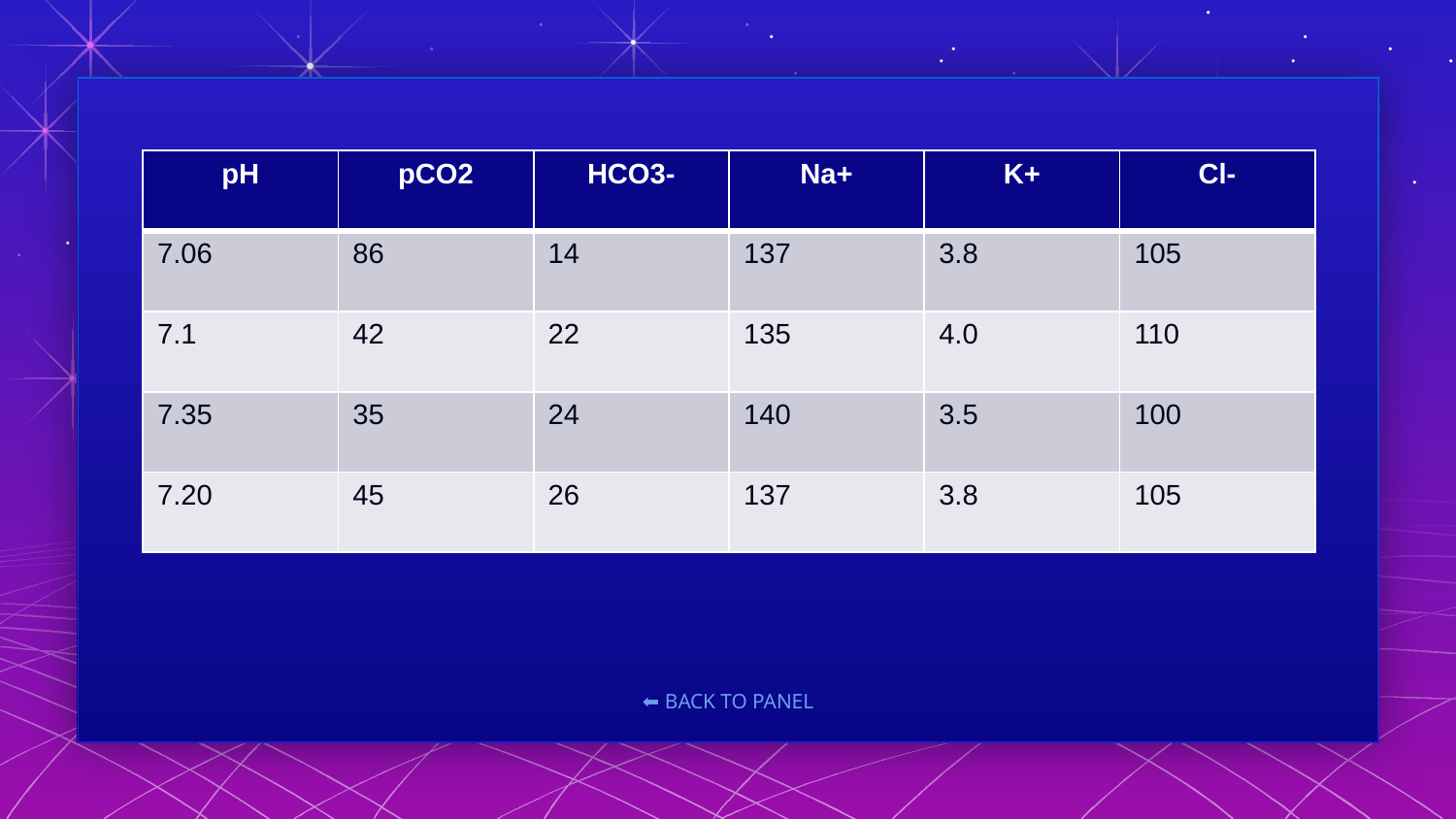

| pH | pCO2 | HCO3- | Na+ | K+ | Cl- |
| --- | --- | --- | --- | --- | --- |
| 7.06 | 86 | 14 | 137 | 3.8 | 105 |
| 7.1 | 42 | 22 | 135 | 4.0 | 110 |
| 7.35 | 35 | 24 | 140 | 3.5 | 100 |
| 7.20 | 45 | 26 | 137 | 3.8 | 105 |
#

## Slide 31
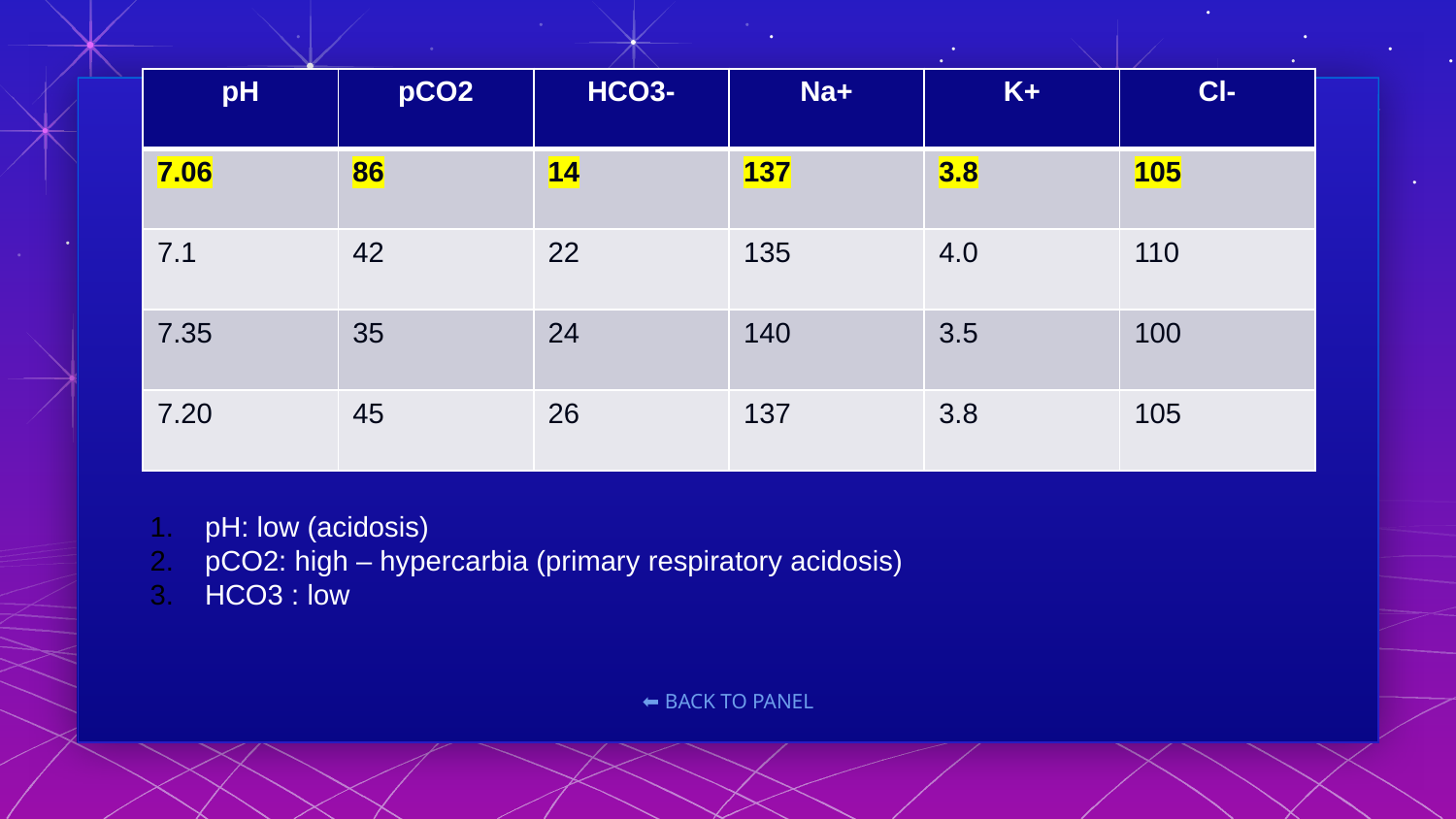

| pH | pCO2 | HCO3- | Na+ | K+ | Cl- |
| --- | --- | --- | --- | --- | --- |
| 7.06 | 86 | 14 | 137 | 3.8 | 105 |
| 7.1 | 42 | 22 | 135 | 4.0 | 110 |
| 7.35 | 35 | 24 | 140 | 3.5 | 100 |
| 7.20 | 45 | 26 | 137 | 3.8 | 105 |
pH: low (acidosis)
pCO2: high – hypercarbia (primary respiratory acidosis)
HCO3 : low

## Slide 32
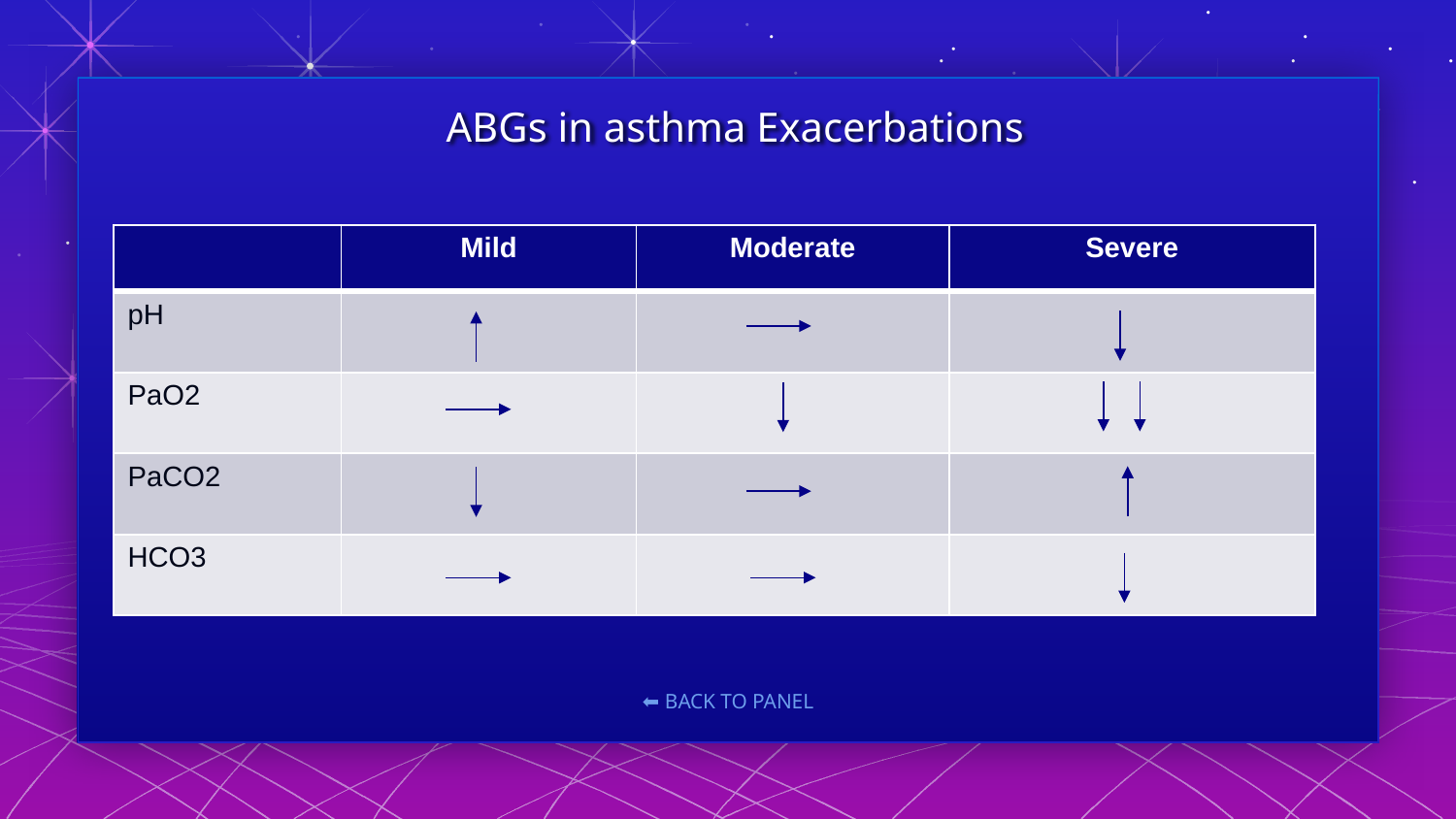

ABGs in asthma Exacerbations
| | Mild | Moderate | Severe |
| --- | --- | --- | --- |
| pH | | | |
| PaO2 | | | |
| PaCO2 | | | |
| HCO3 | | | |

## Slide 33
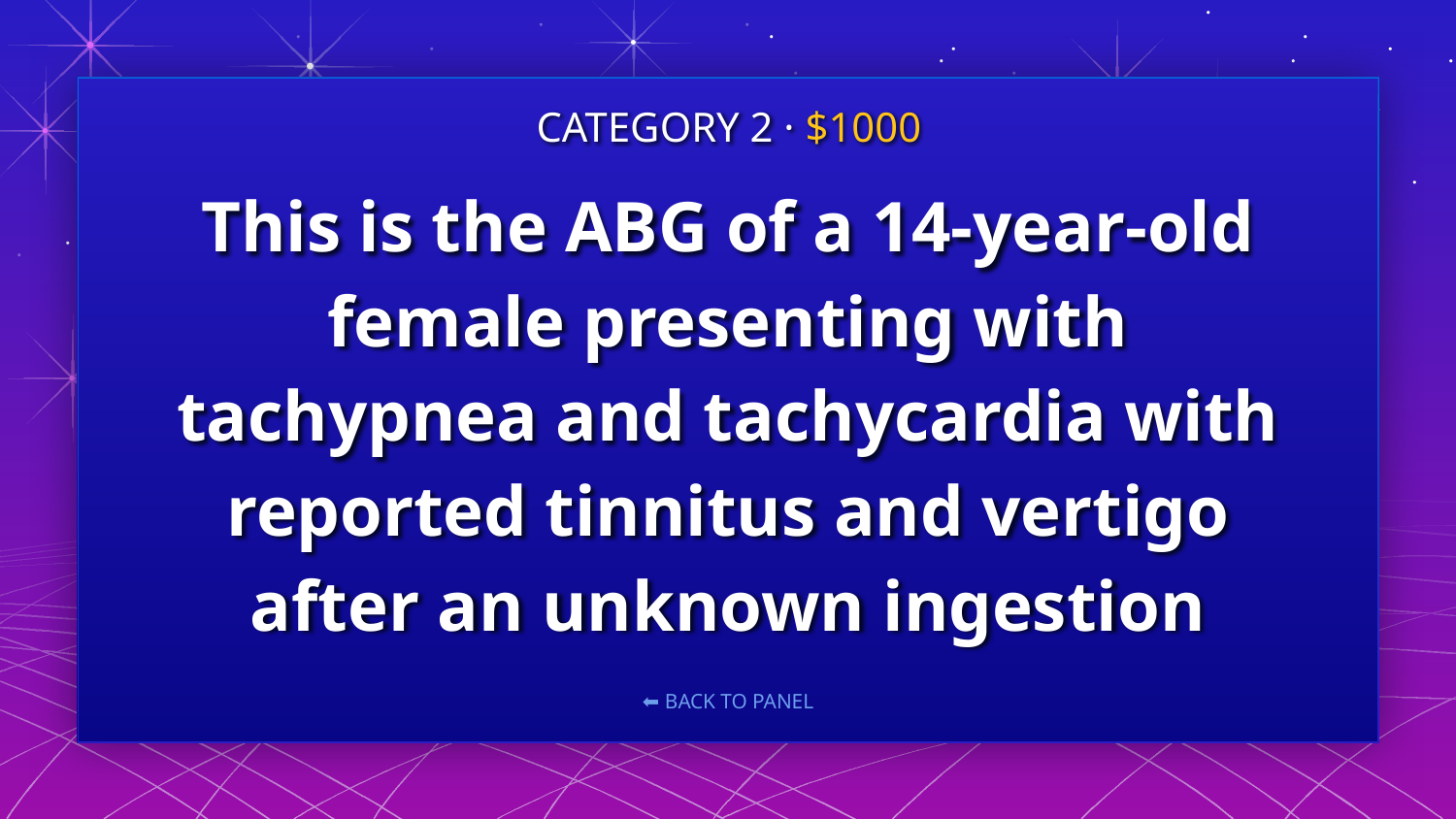

CATEGORY 2 · $1000
# This is the ABG of a 14-year-old female presenting with tachypnea and tachycardia with reported tinnitus and vertigo after an unknown ingestion

## Slide 34
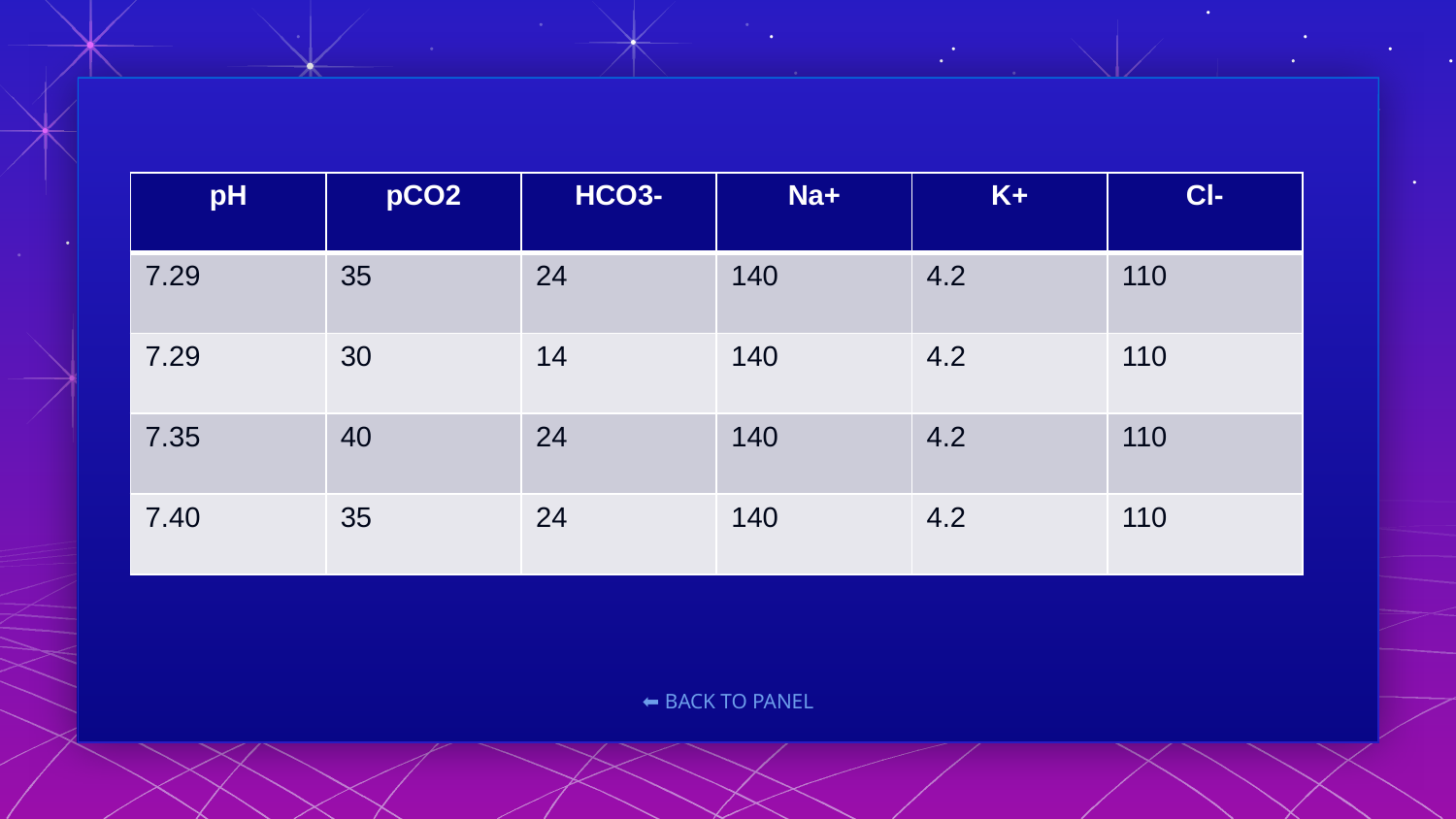

| pH | pCO2 | HCO3- | Na+ | K+ | Cl- |
| --- | --- | --- | --- | --- | --- |
| 7.29 | 35 | 24 | 140 | 4.2 | 110 |
| 7.29 | 30 | 14 | 140 | 4.2 | 110 |
| 7.35 | 40 | 24 | 140 | 4.2 | 110 |
| 7.40 | 35 | 24 | 140 | 4.2 | 110 |

## Slide 35
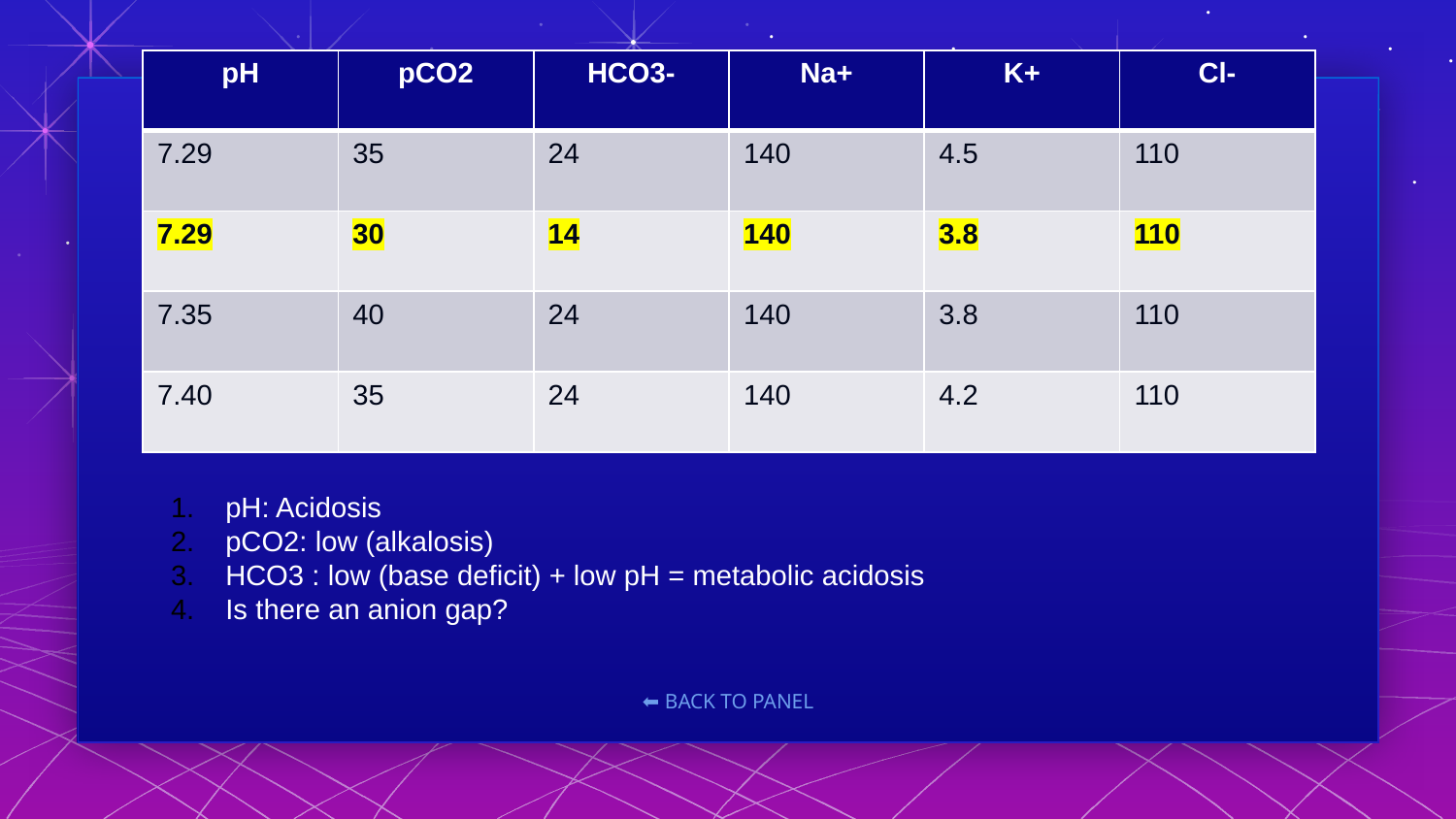

| pH | pCO2 | HCO3- | Na+ | K+ | Cl- |
| --- | --- | --- | --- | --- | --- |
| 7.29 | 35 | 24 | 140 | 4.5 | 110 |
| 7.29 | 30 | 14 | 140 | 3.8 | 110 |
| 7.35 | 40 | 24 | 140 | 3.8 | 110 |
| 7.40 | 35 | 24 | 140 | 4.2 | 110 |
pH: Acidosis
pCO2: low (alkalosis)
HCO3 : low (base deficit) + low pH = metabolic acidosis
Is there an anion gap?

## Slide 36
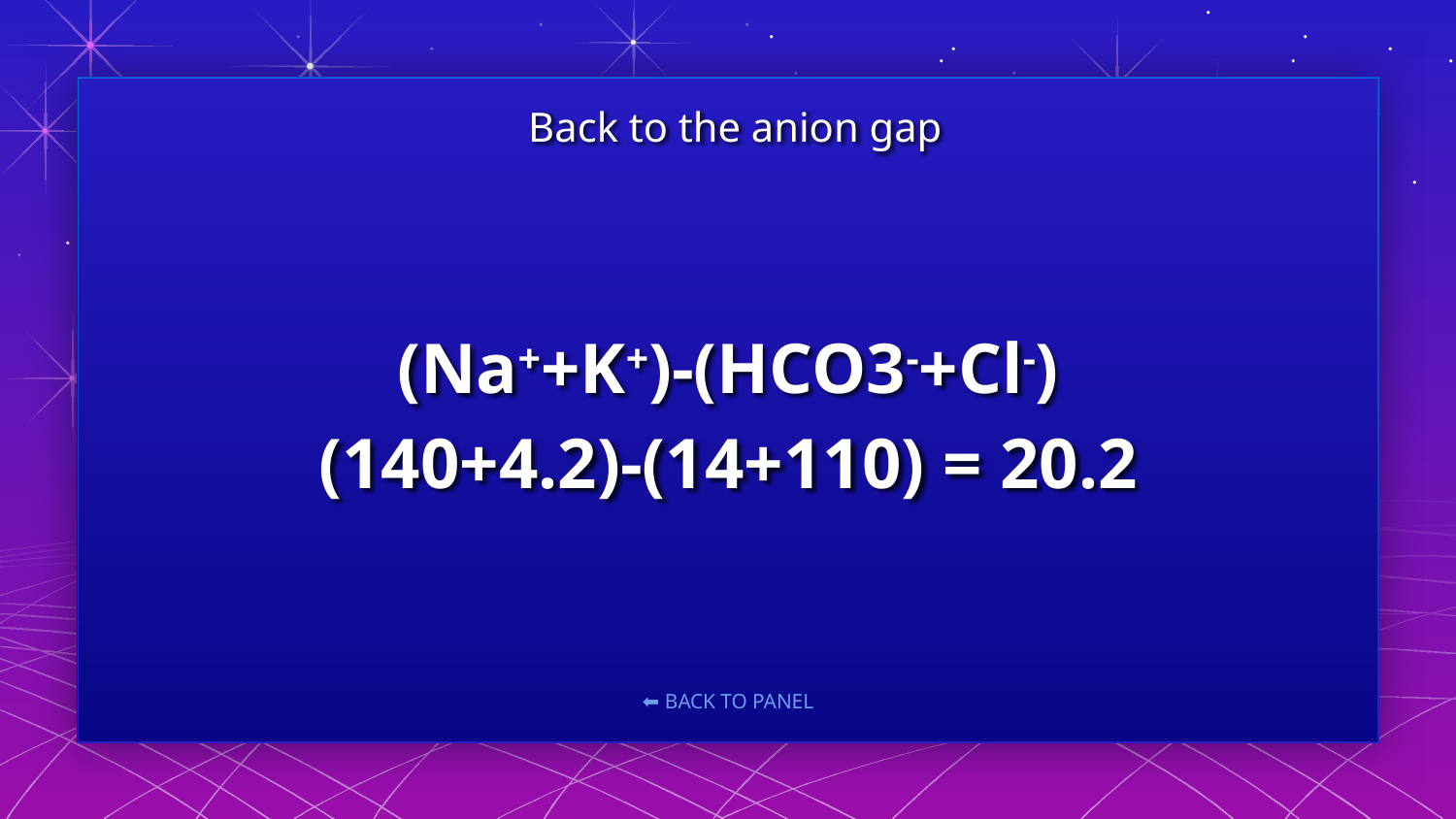

Back to the anion gap
# (Na++K+)-(HCO3-+Cl-) (140+4.2)-(14+110) = 20.2

## Slide 37
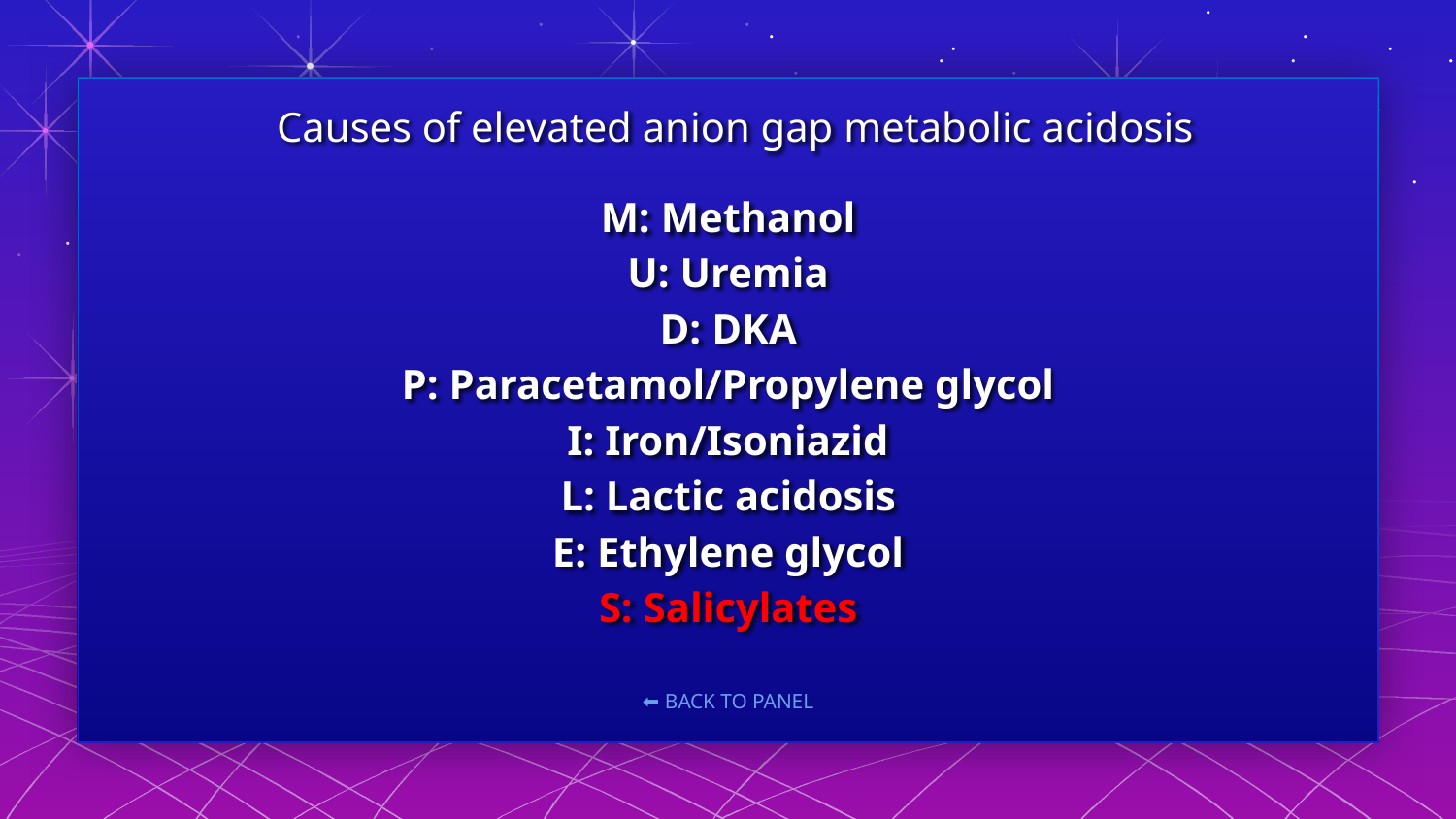

Causes of elevated anion gap metabolic acidosis
# M: Methanol U: Uremia D: DKA P: Paracetamol/Propylene glycol I: Iron/Isoniazid L: Lactic acidosis E: Ethylene glycol S: Salicylates

## Slide 38
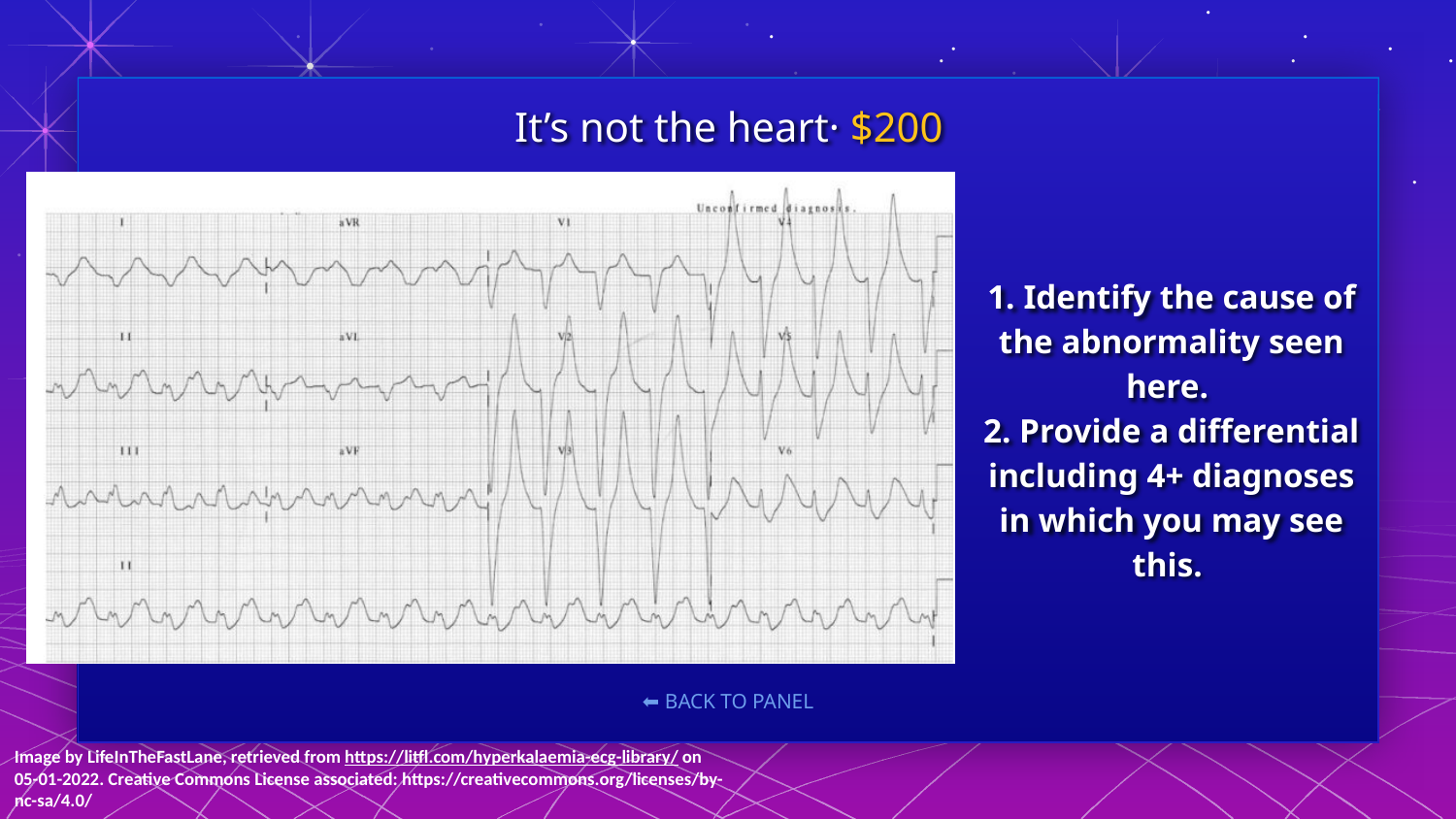

It’s not the heart· $200
# 1. Identify the cause of the abnormality seen here. 2. Provide a differential including 4+ diagnoses in which you may see this.
Image by LifeInTheFastLane, retrieved from https://litfl.com/hyperkalaemia-ecg-library/ on 05-01-2022. Creative Commons License associated: https://creativecommons.org/licenses/by-nc-sa/4.0/

## Slide 39
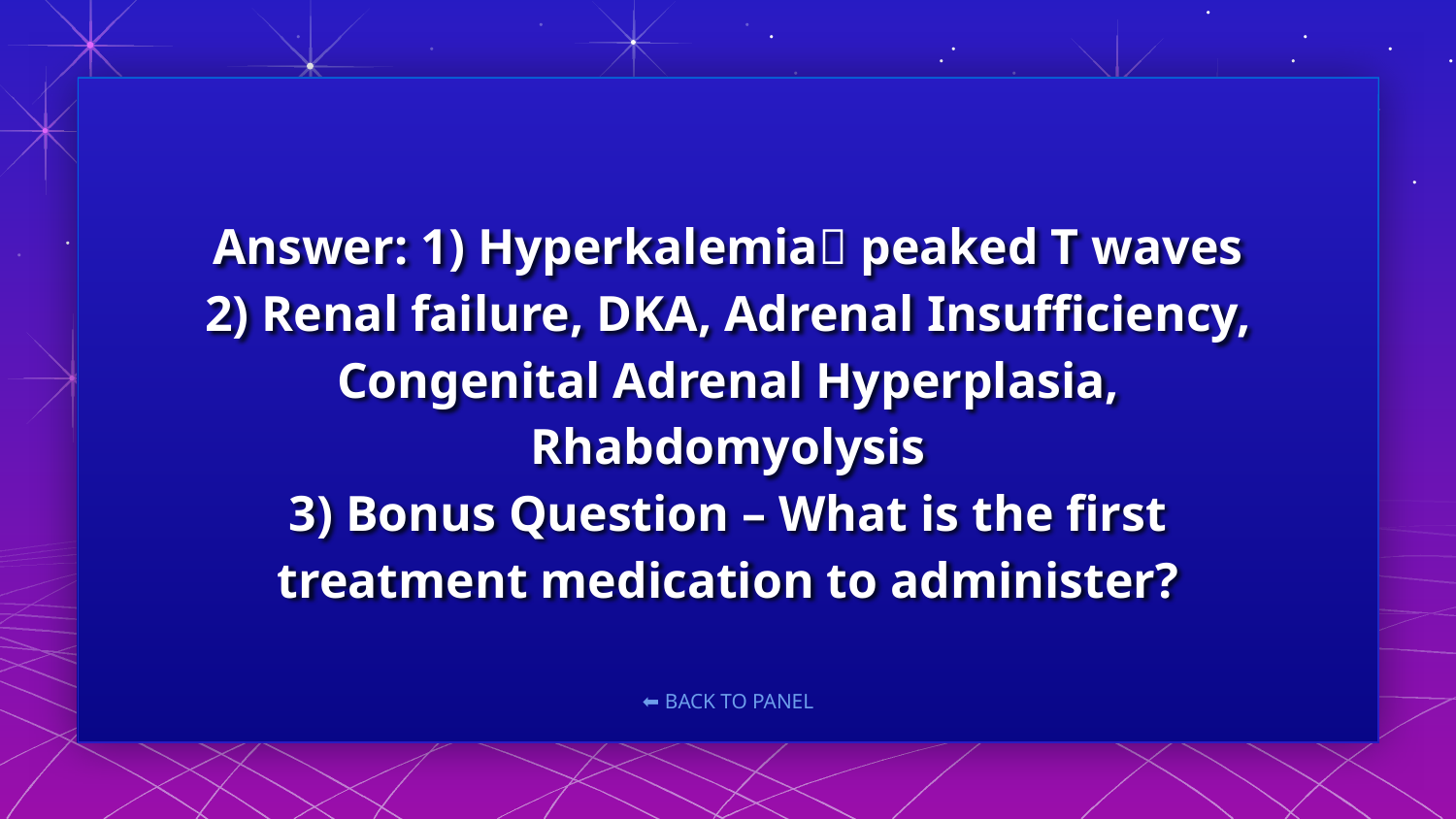

# Answer: 1) Hyperkalemia peaked T waves 2) Renal failure, DKA, Adrenal Insufficiency, Congenital Adrenal Hyperplasia, Rhabdomyolysis 3) Bonus Question – What is the first treatment medication to administer?

## Slide 40
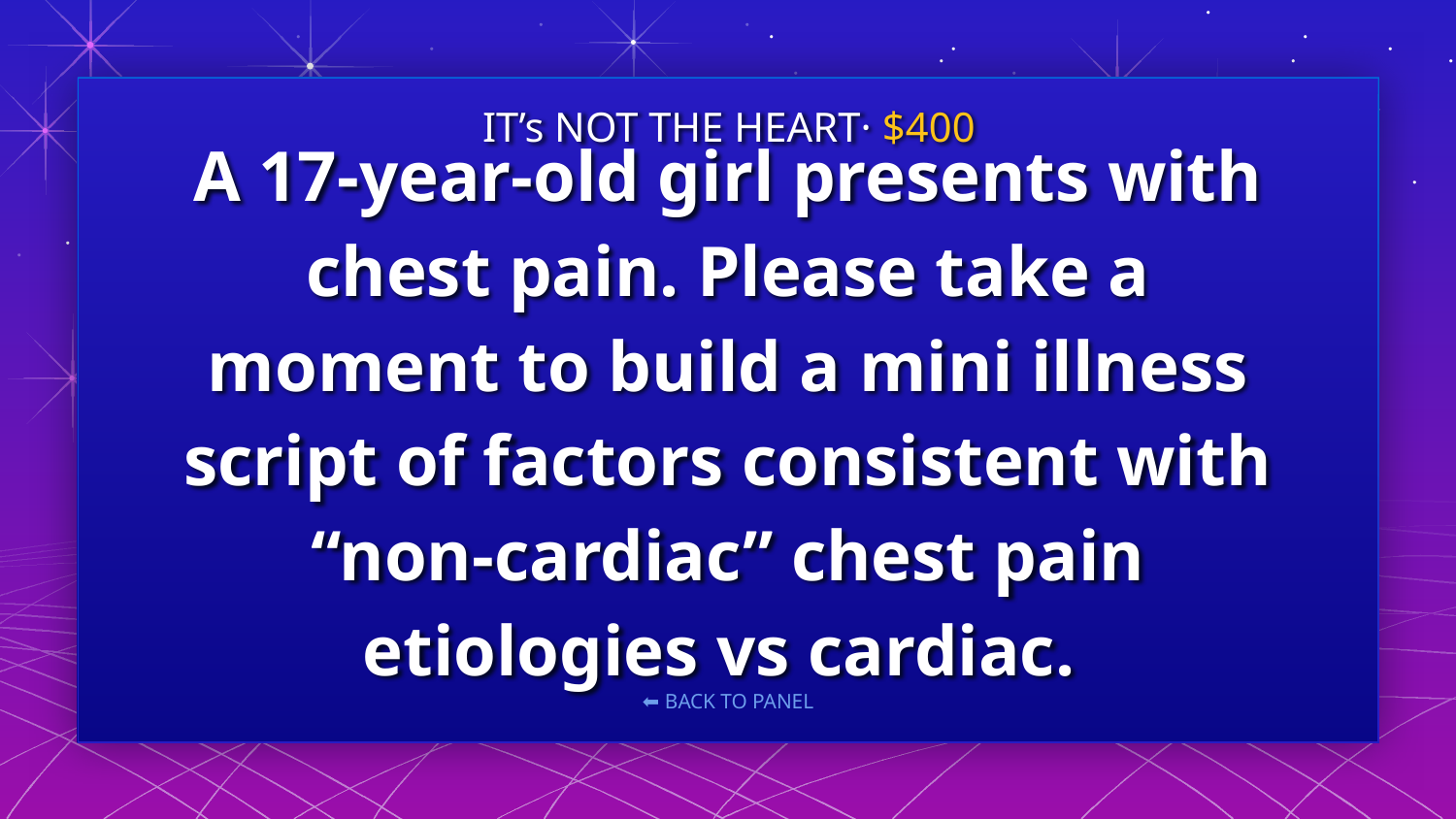

IT’s NOT THE HEART· $400
# A 17-year-old girl presents with chest pain. Please take a moment to build a mini illness script of factors consistent with “non-cardiac” chest pain etiologies vs cardiac.

## Slide 41
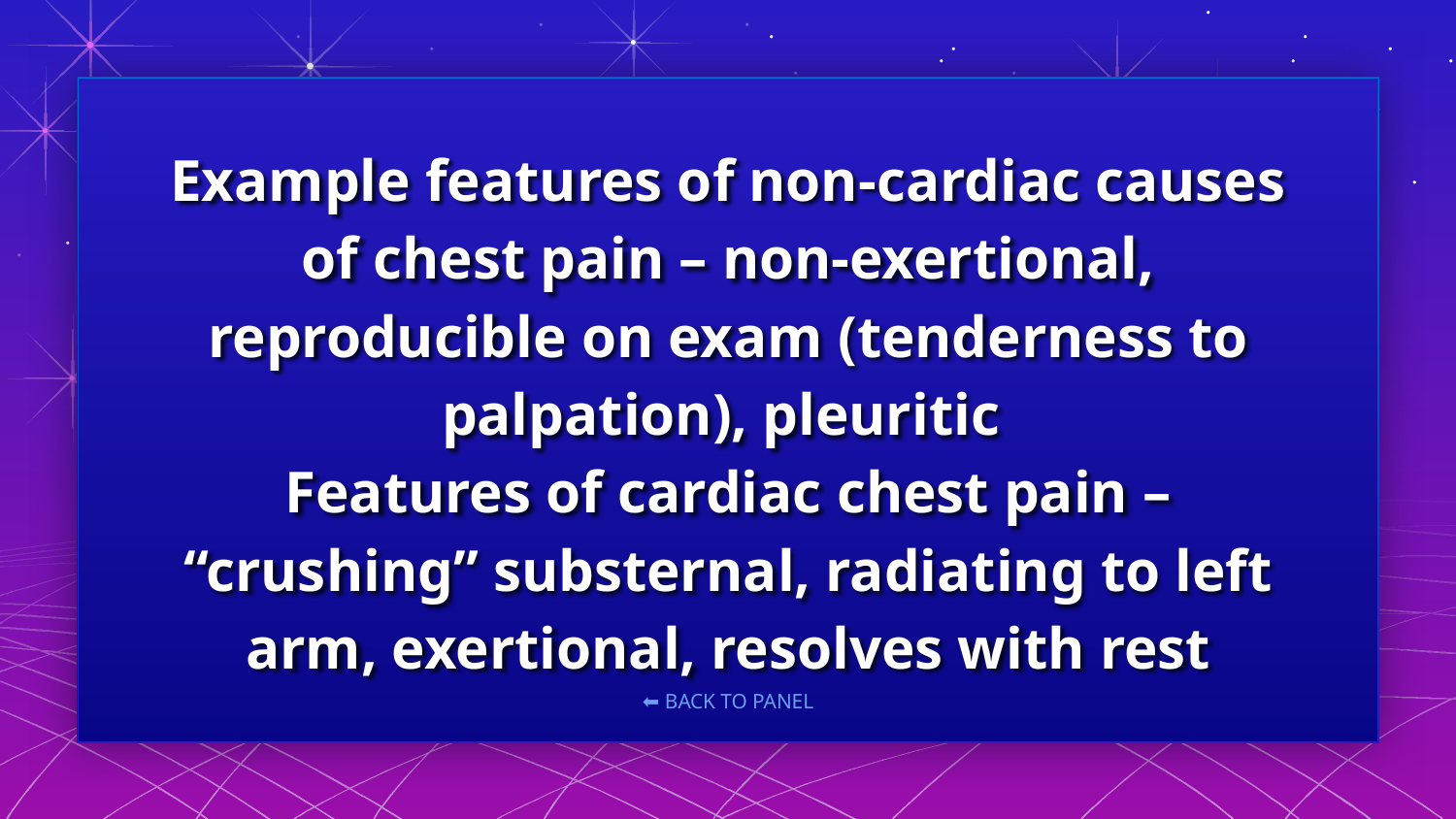

# Example features of non-cardiac causes of chest pain – non-exertional, reproducible on exam (tenderness to palpation), pleuritic Features of cardiac chest pain – “crushing” substernal, radiating to left arm, exertional, resolves with rest

## Slide 42
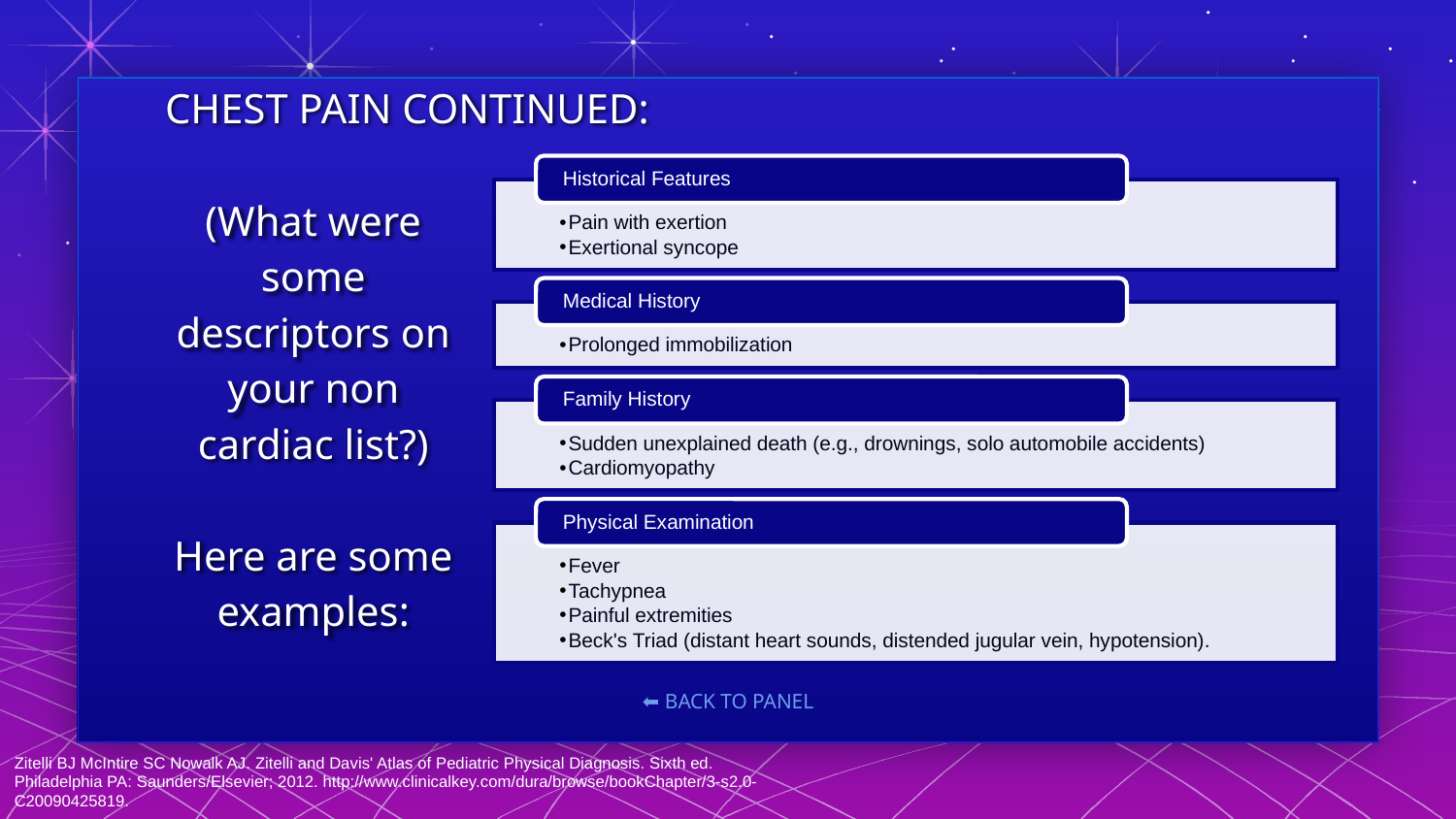

CHEST PAIN CONTINUED:
# (What were some descriptors on your non cardiac list?) Here are some examples:
Zitelli BJ McIntire SC Nowalk AJ. Zitelli and Davis' Atlas of Pediatric Physical Diagnosis. Sixth ed. Philadelphia PA: Saunders/Elsevier; 2012. http://www.clinicalkey.com/dura/browse/bookChapter/3-s2.0-C20090425819.

## Slide 43
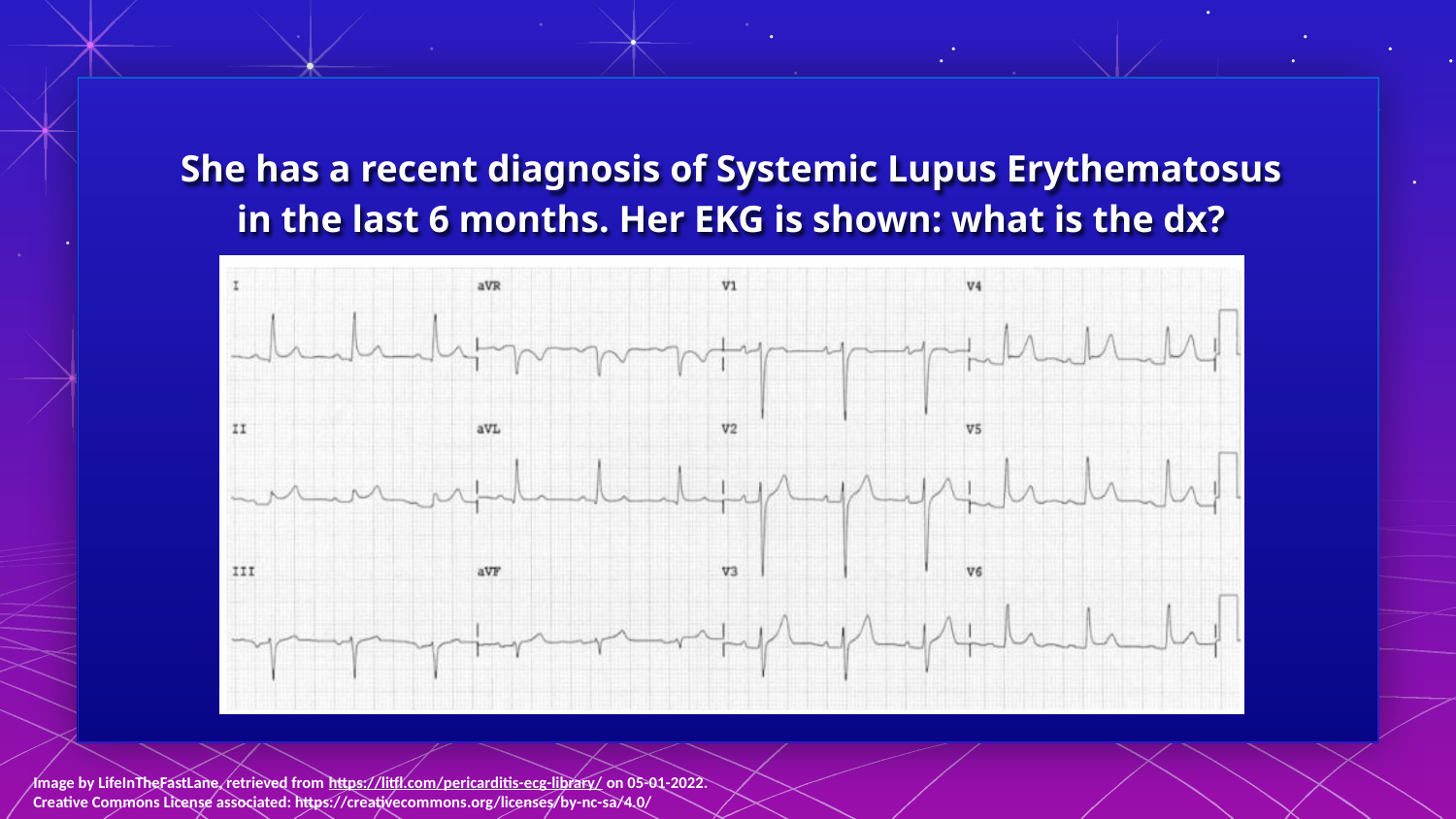

# She has a recent diagnosis of Systemic Lupus Erythematosus in the last 6 months. Her EKG is shown: what is the dx?
Image by LifeInTheFastLane, retrieved from https://litfl.com/pericarditis-ecg-library/ on 05-01-2022. Creative Commons License associated: https://creativecommons.org/licenses/by-nc-sa/4.0/

## Slide 44
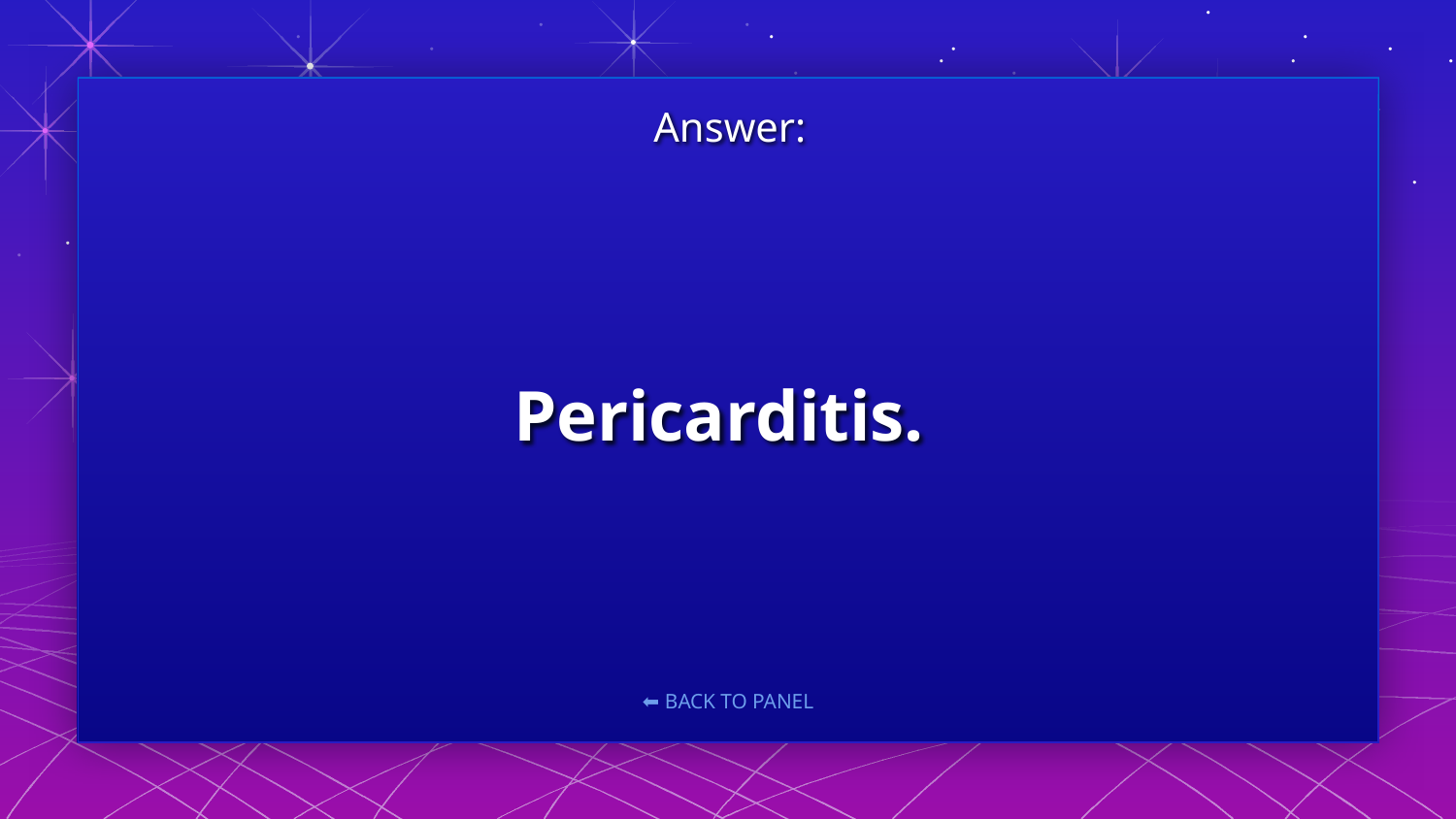

Answer:
# Pericarditis.

## Slide 45
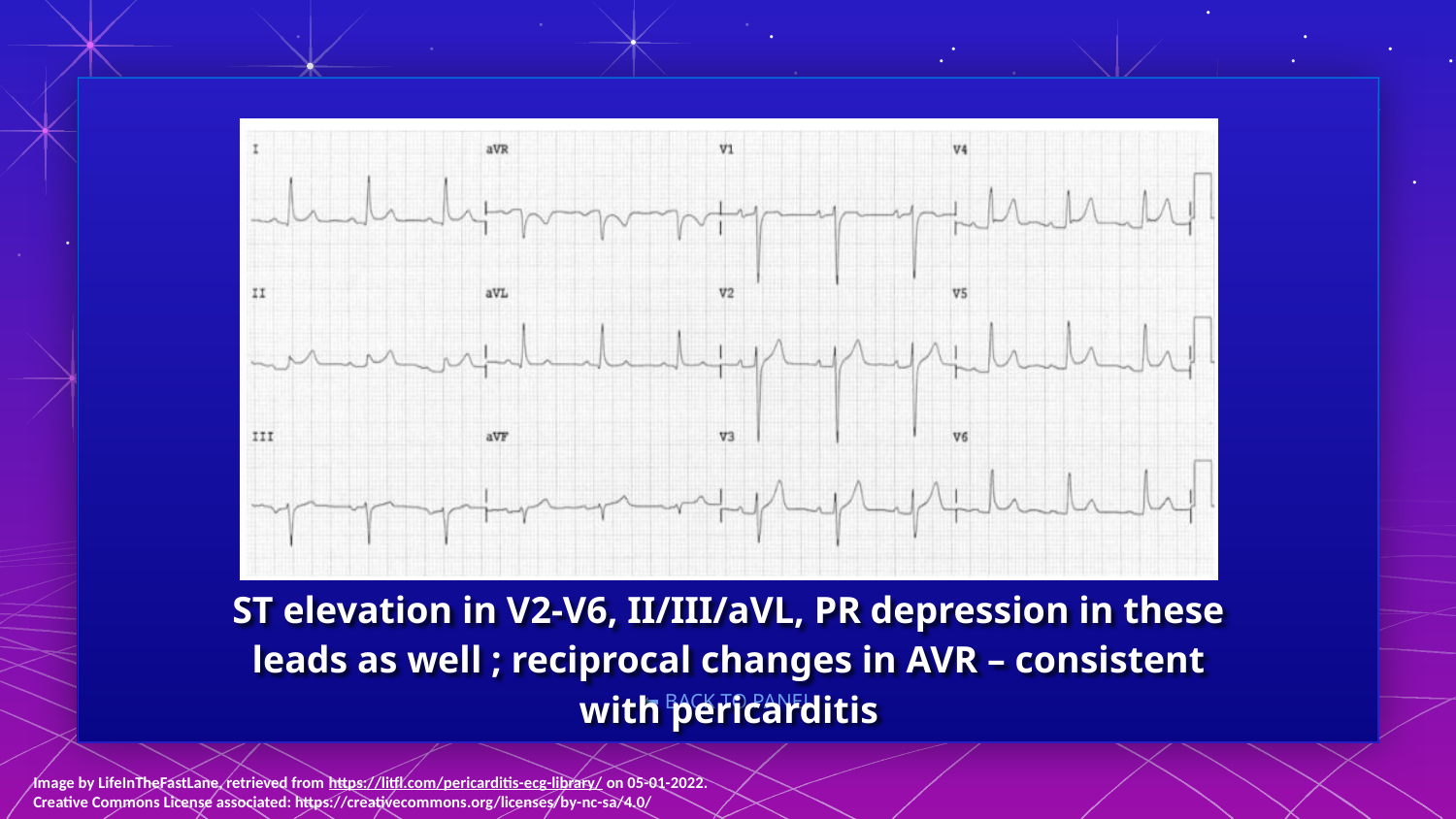

# ST elevation in V2-V6, II/III/aVL, PR depression in these leads as well ; reciprocal changes in AVR – consistent with pericarditis
Image by LifeInTheFastLane, retrieved from https://litfl.com/pericarditis-ecg-library/ on 05-01-2022. Creative Commons License associated: https://creativecommons.org/licenses/by-nc-sa/4.0/

## Slide 46
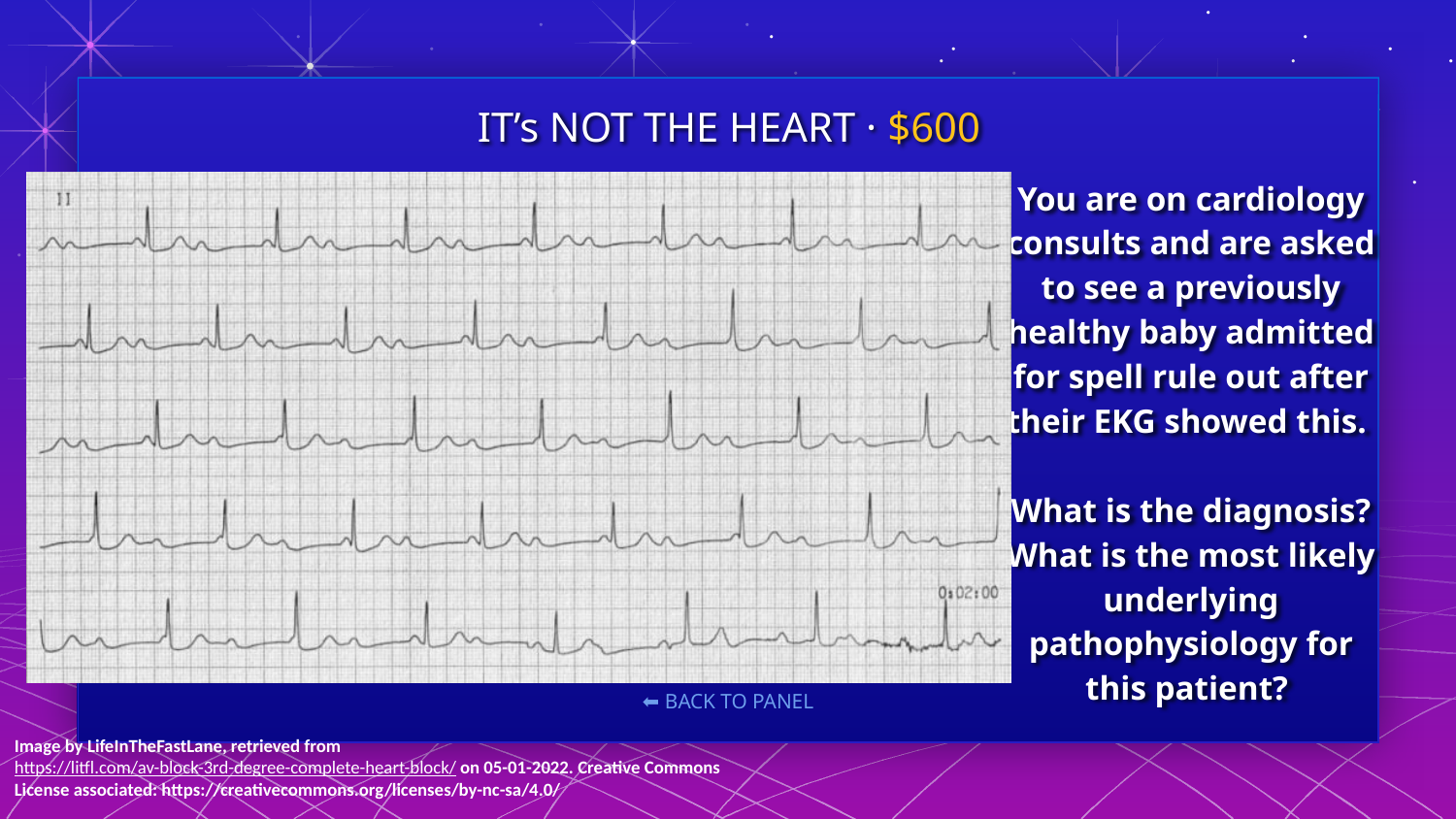

IT’s NOT THE HEART · $600
# You are on cardiology consults and are asked to see a previously healthy baby admitted for spell rule out after their EKG showed this. What is the diagnosis? What is the most likely underlying pathophysiology for this patient?
Image by LifeInTheFastLane, retrieved from https://litfl.com/av-block-3rd-degree-complete-heart-block/ on 05-01-2022. Creative Commons License associated: https://creativecommons.org/licenses/by-nc-sa/4.0/

## Slide 47
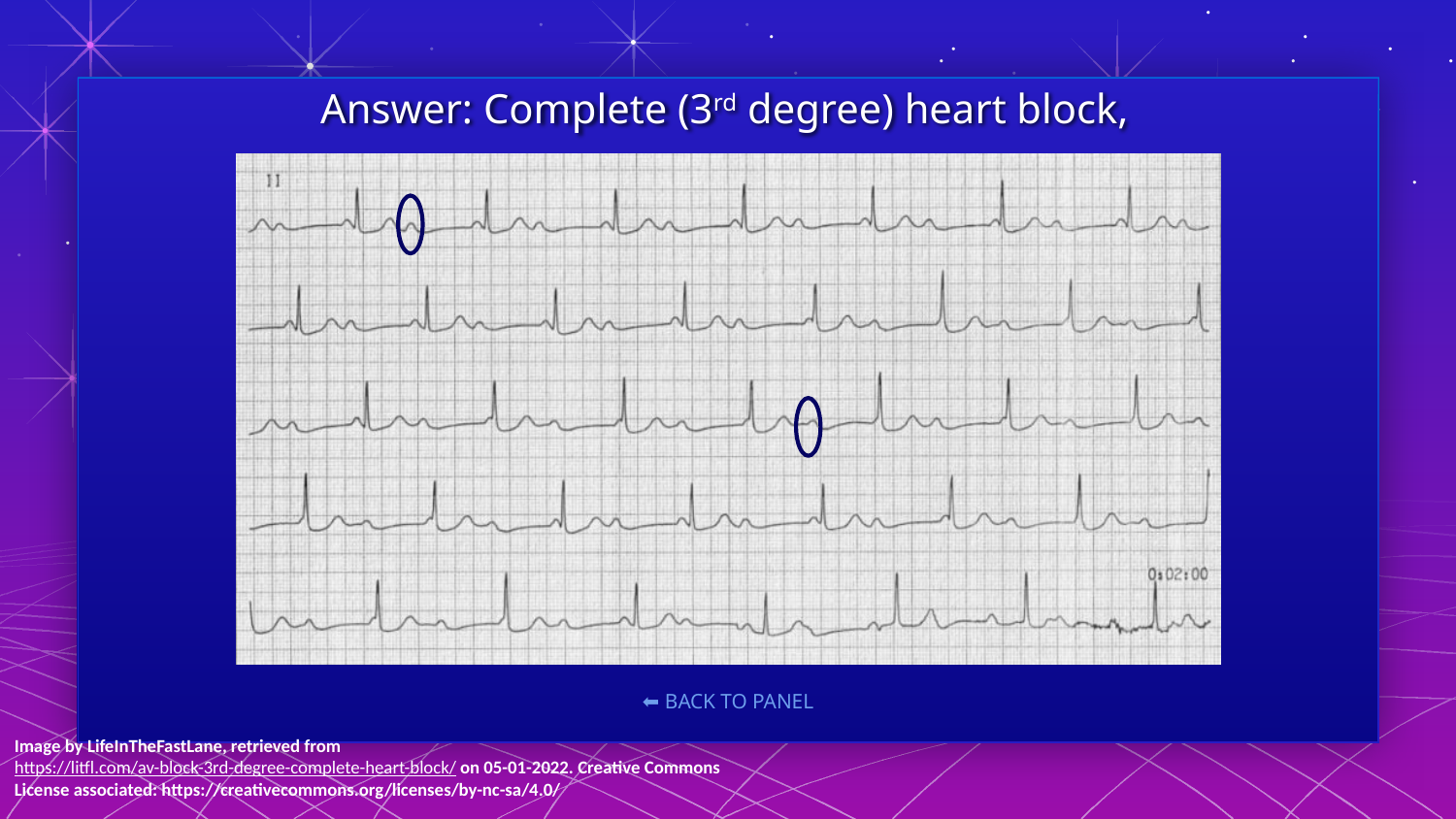

Answer: Complete (3rd degree) heart block,
#
Image by LifeInTheFastLane, retrieved from https://litfl.com/av-block-3rd-degree-complete-heart-block/ on 05-01-2022. Creative Commons License associated: https://creativecommons.org/licenses/by-nc-sa/4.0/

## Slide 48
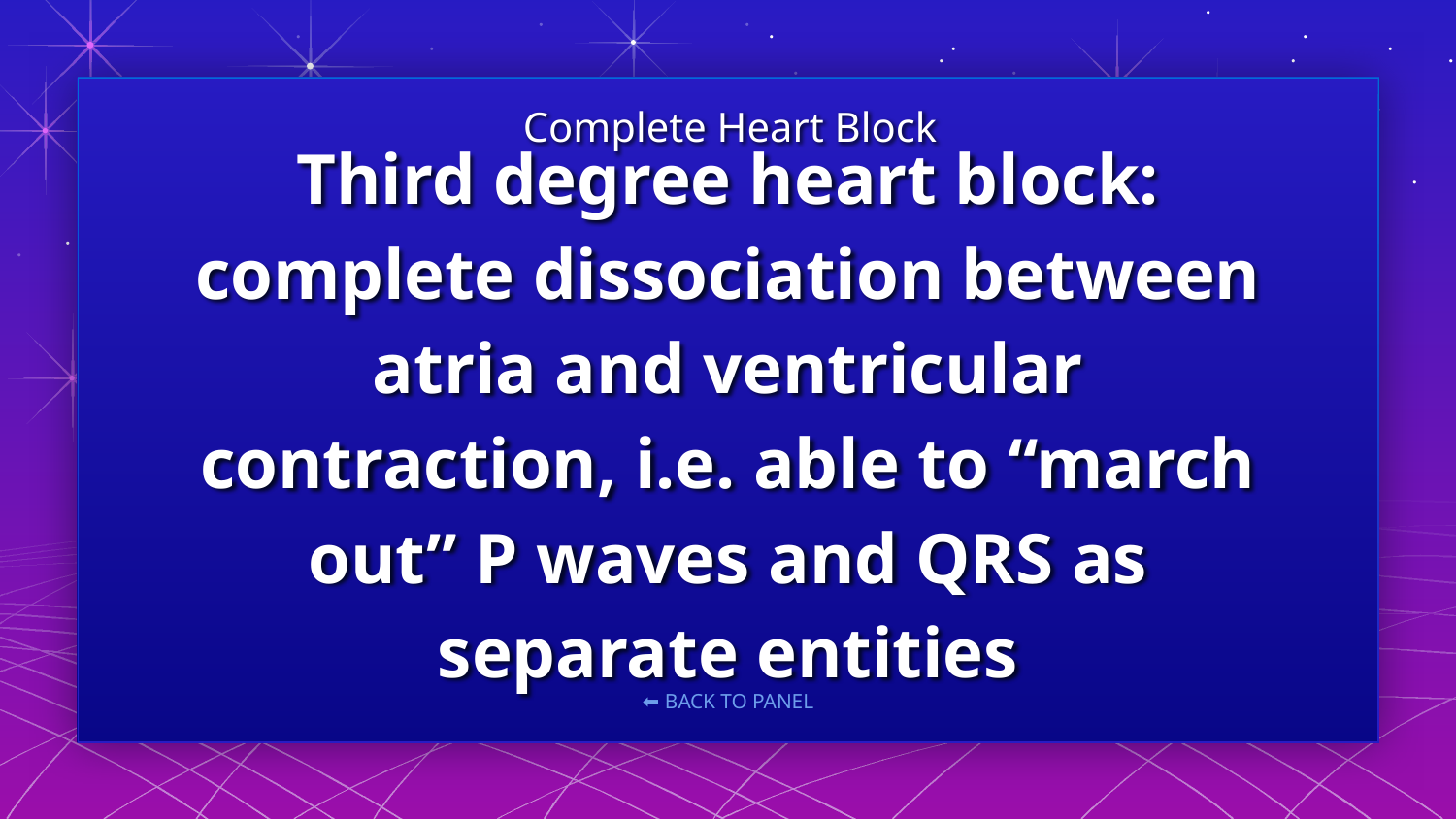

Complete Heart Block
# Third degree heart block: complete dissociation between atria and ventricular contraction, i.e. able to “march out” P waves and QRS as separate entities

## Slide 49
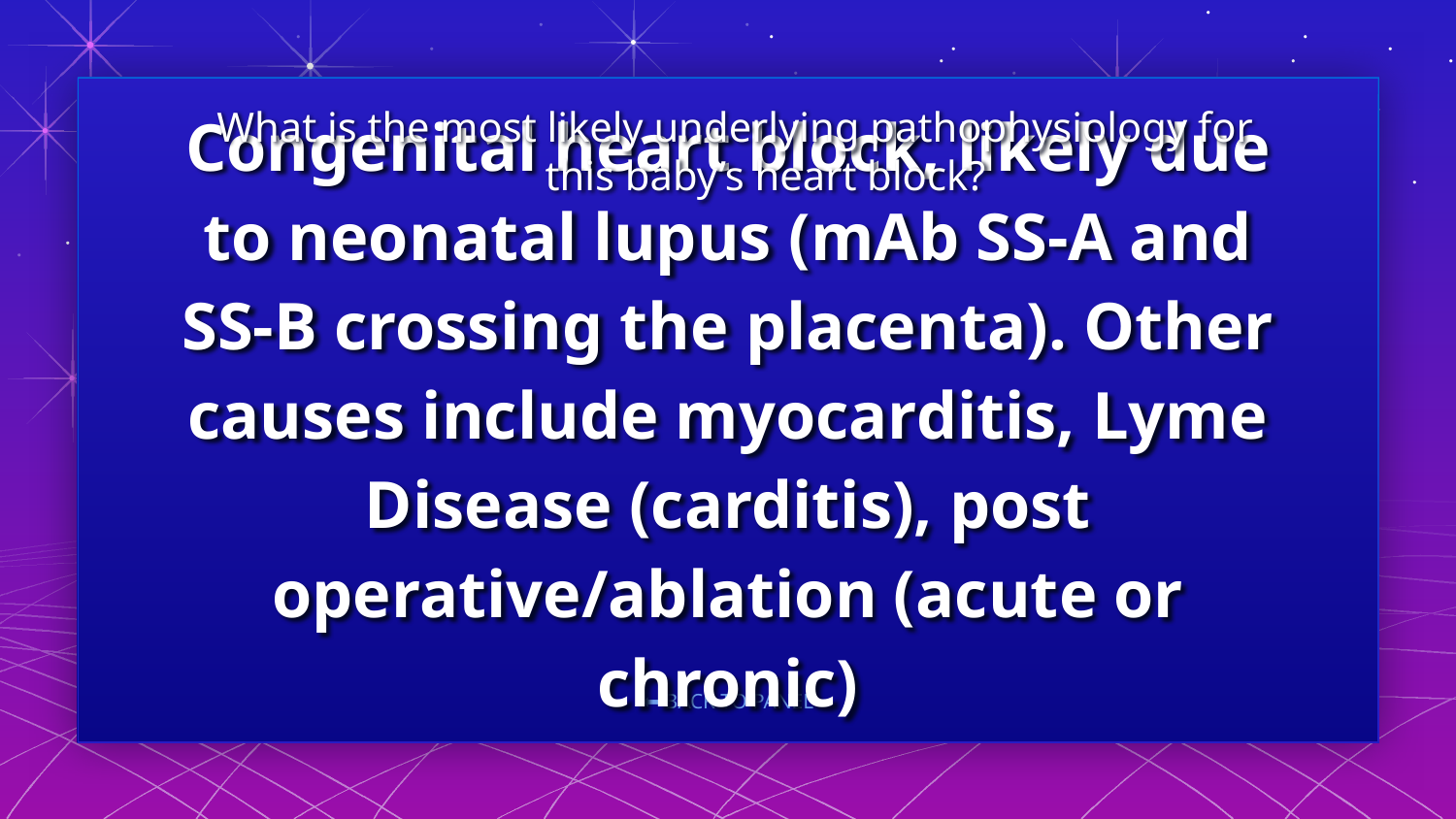

What is the most likely underlying pathophysiology for this baby’s heart block?
# Congenital heart block, likely due to neonatal lupus (mAb SS-A and SS-B crossing the placenta). Other causes include myocarditis, Lyme Disease (carditis), post operative/ablation (acute or chronic)

## Slide 50
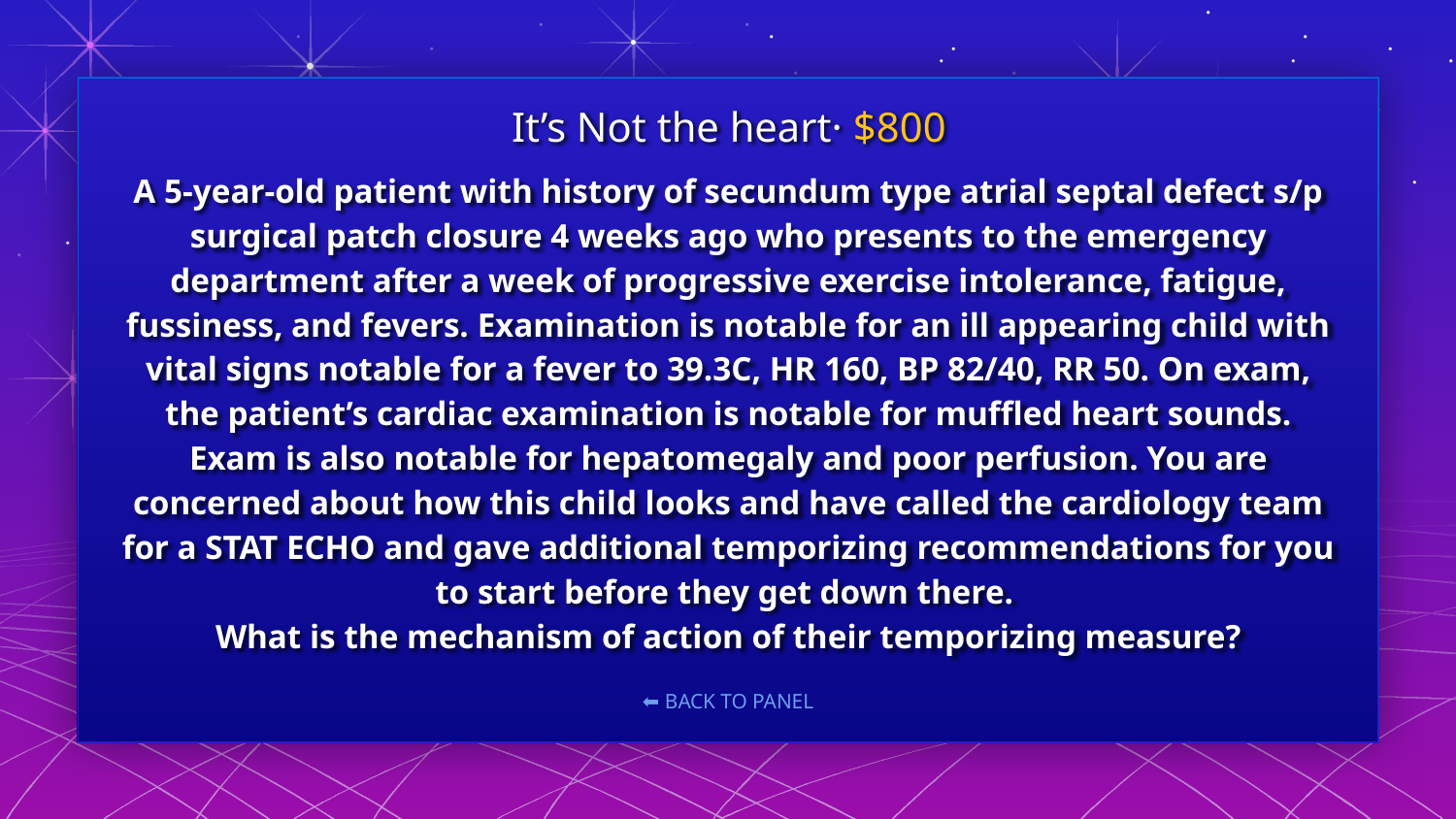

It’s Not the heart· $800
# A 5-year-old patient with history of secundum type atrial septal defect s/p surgical patch closure 4 weeks ago who presents to the emergency department after a week of progressive exercise intolerance, fatigue, fussiness, and fevers. Examination is notable for an ill appearing child with vital signs notable for a fever to 39.3C, HR 160, BP 82/40, RR 50. On exam, the patient’s cardiac examination is notable for muffled heart sounds. Exam is also notable for hepatomegaly and poor perfusion. You are concerned about how this child looks and have called the cardiology team for a STAT ECHO and gave additional temporizing recommendations for you to start before they get down there. What is the mechanism of action of their temporizing measure?

## Slide 51
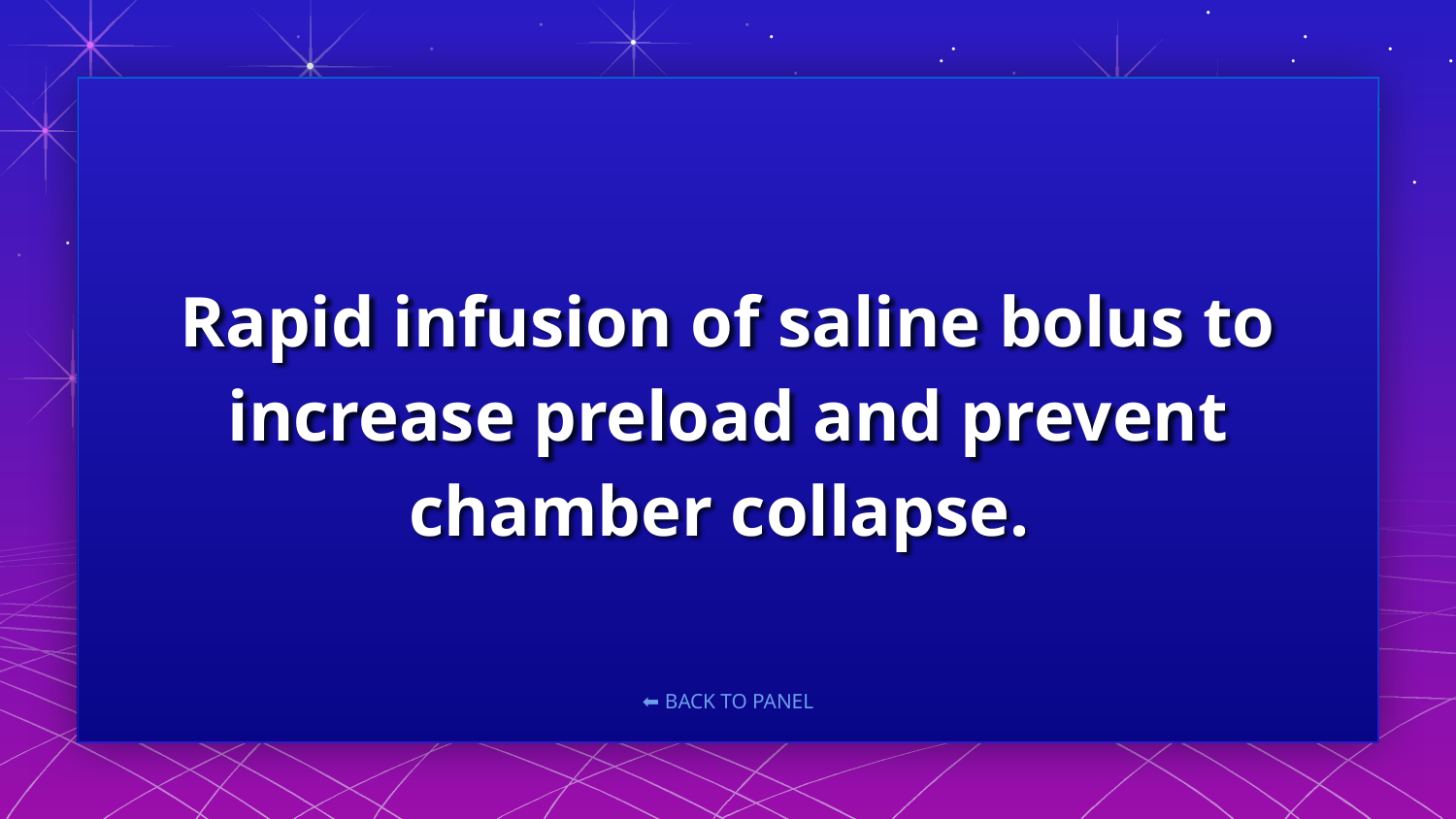

# Rapid infusion of saline bolus to increase preload and prevent chamber collapse.

## Slide 52
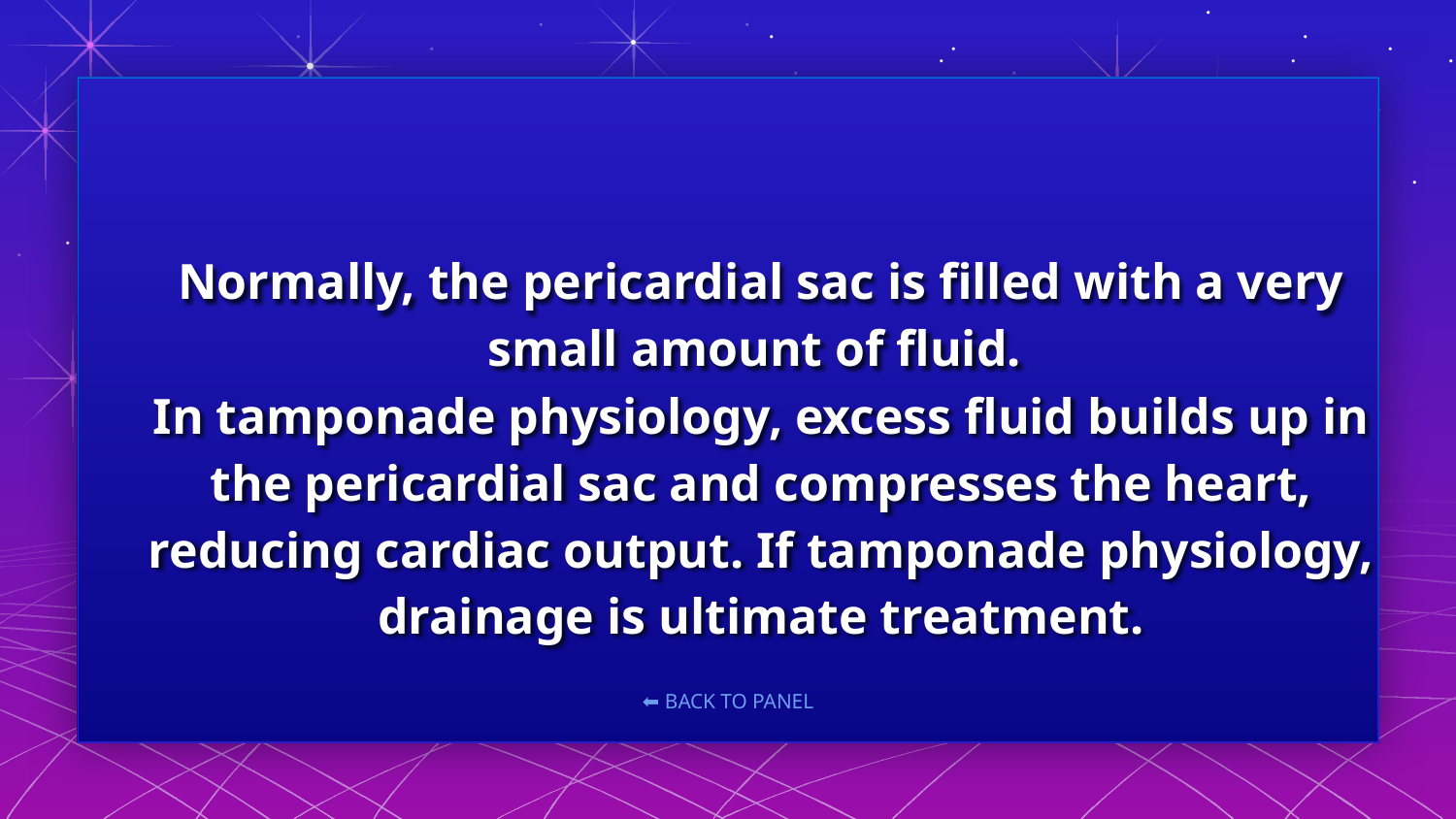

# Normally, the pericardial sac is filled with a very small amount of fluid. In tamponade physiology, excess fluid builds up in the pericardial sac and compresses the heart, reducing cardiac output. If tamponade physiology, drainage is ultimate treatment.

## Slide 53
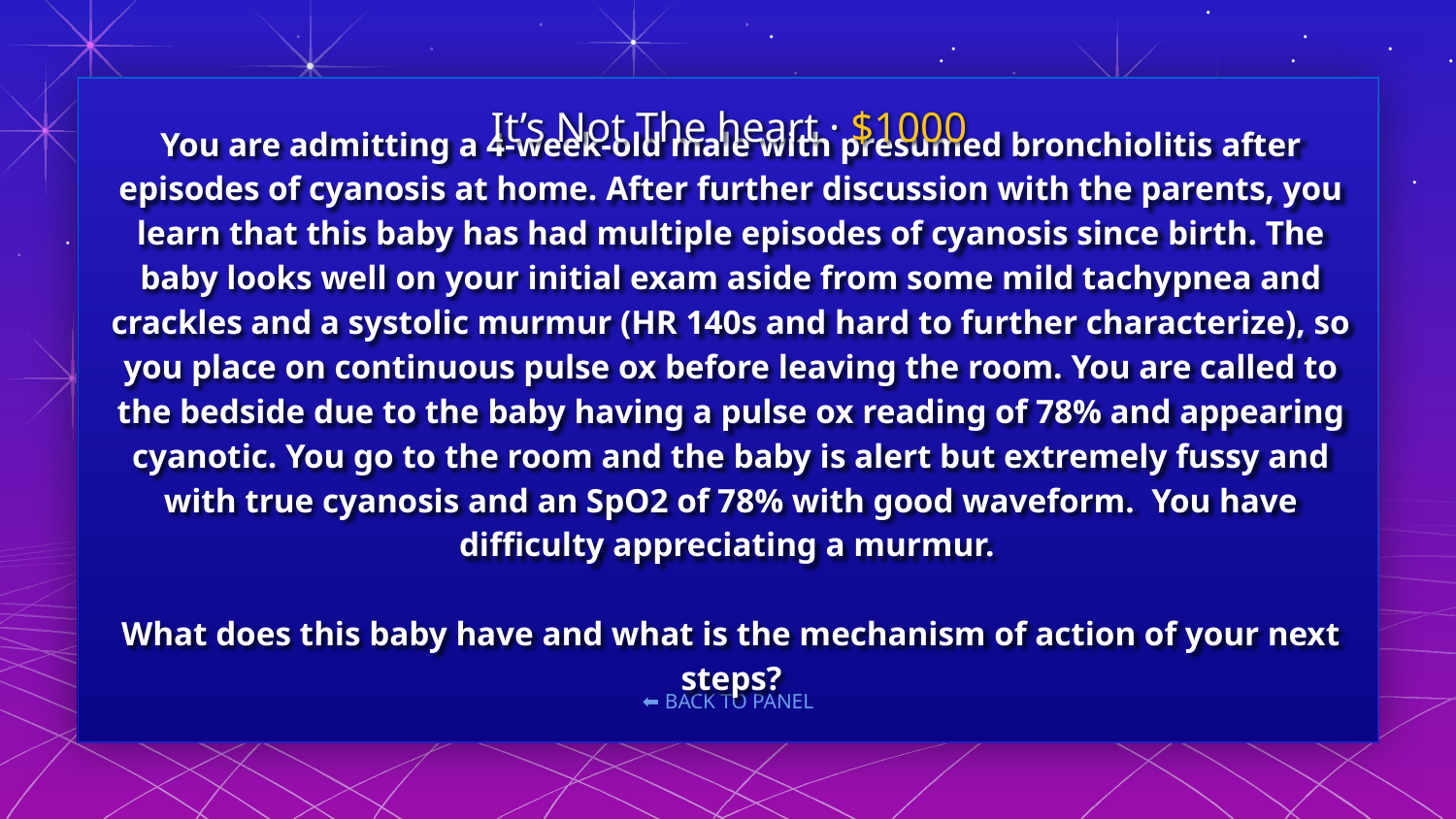

It’s Not The heart · $1000
# You are admitting a 4-week-old male with presumed bronchiolitis after episodes of cyanosis at home. After further discussion with the parents, you learn that this baby has had multiple episodes of cyanosis since birth. The baby looks well on your initial exam aside from some mild tachypnea and crackles and a systolic murmur (HR 140s and hard to further characterize), so you place on continuous pulse ox before leaving the room. You are called to the bedside due to the baby having a pulse ox reading of 78% and appearing cyanotic. You go to the room and the baby is alert but extremely fussy and with true cyanosis and an SpO2 of 78% with good waveform. You have difficulty appreciating a murmur. What does this baby have and what is the mechanism of action of your next steps?

## Slide 54
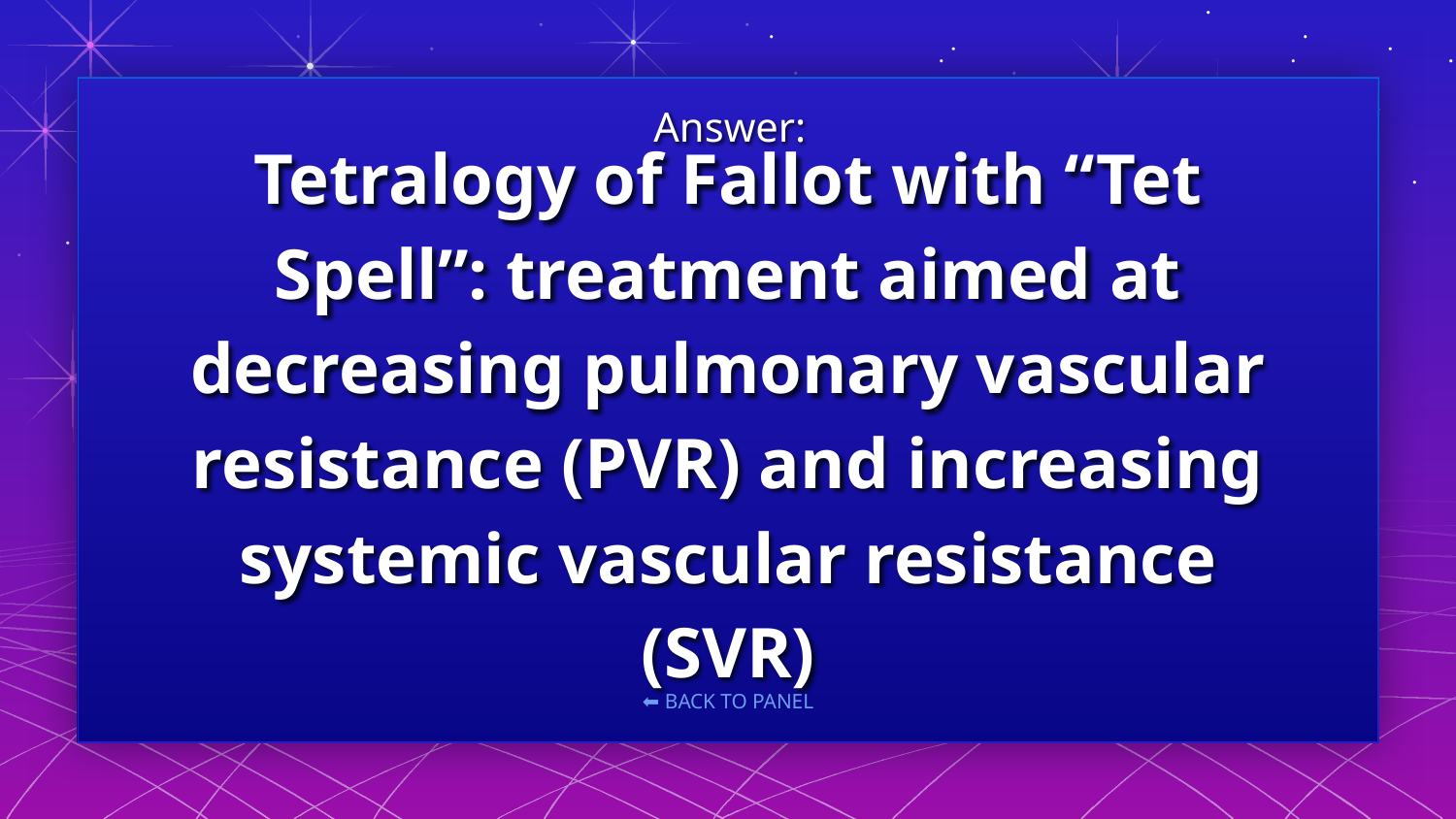

Answer:
# Tetralogy of Fallot with “Tet Spell”: treatment aimed at decreasing pulmonary vascular resistance (PVR) and increasing systemic vascular resistance (SVR)

## Slide 55
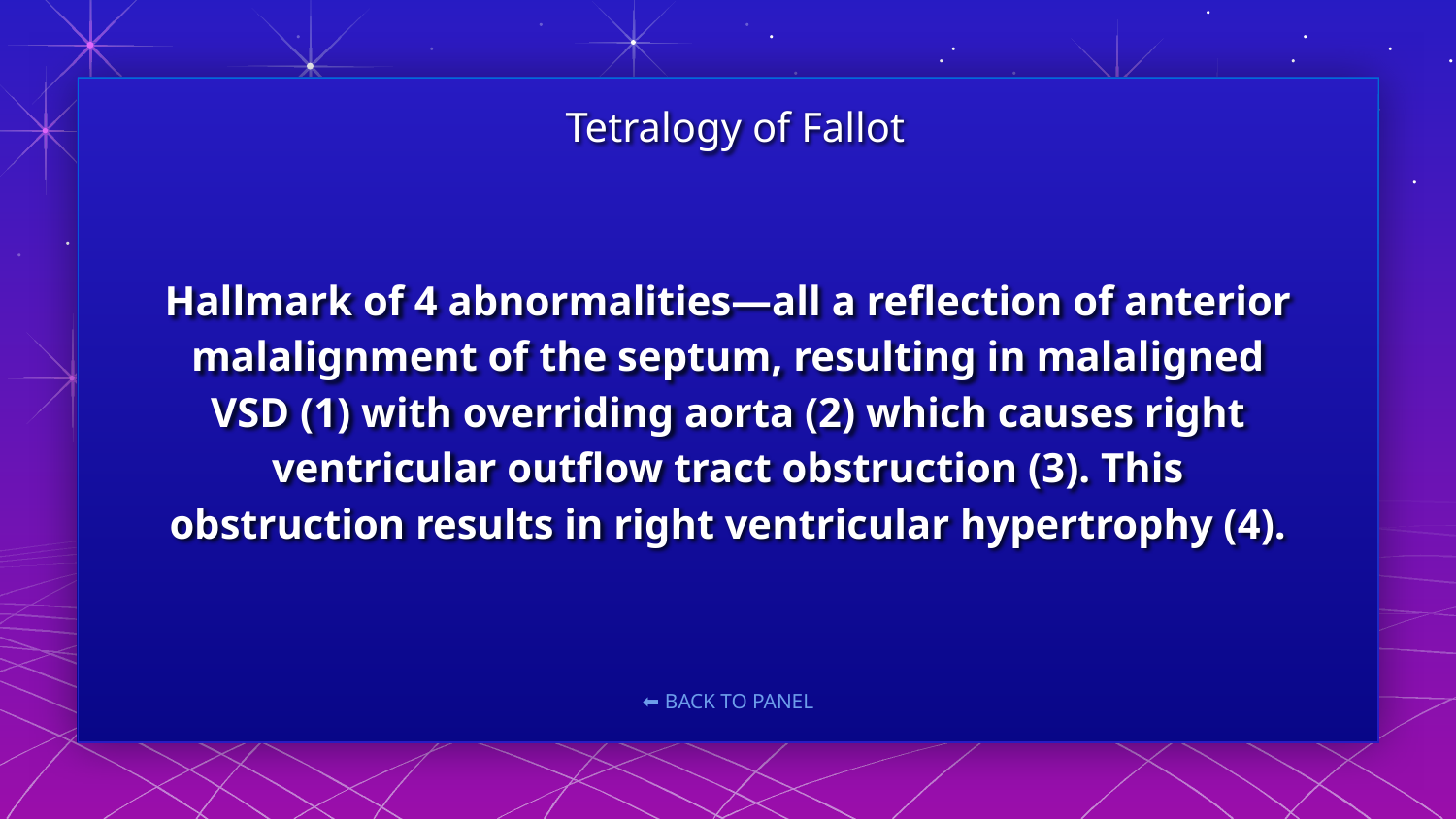

Tetralogy of Fallot
# Hallmark of 4 abnormalities—all a reflection of anterior malalignment of the septum, resulting in malaligned VSD (1) with overriding aorta (2) which causes right ventricular outflow tract obstruction (3). This obstruction results in right ventricular hypertrophy (4).

## Slide 56
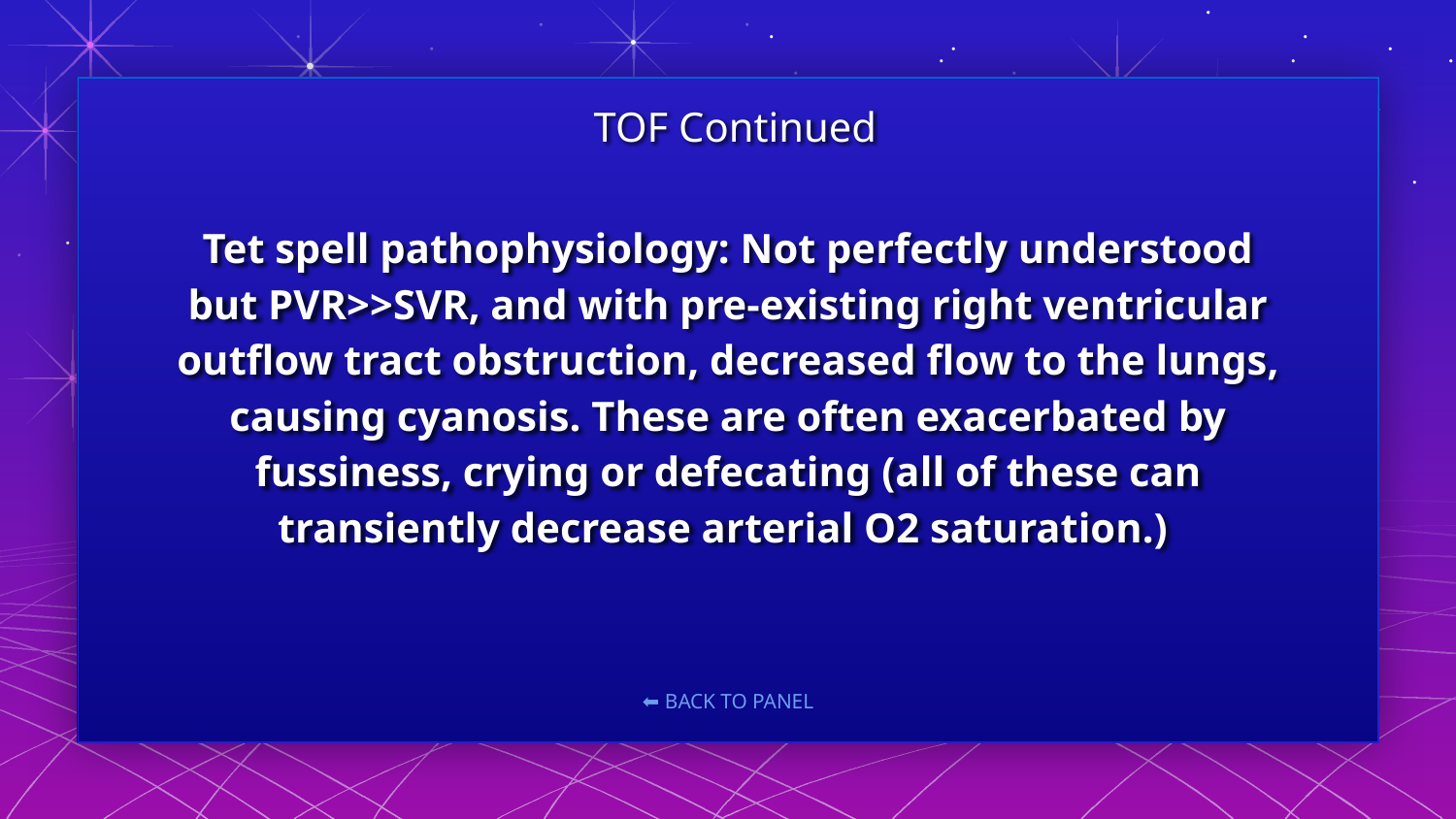

TOF Continued
# Tet spell pathophysiology: Not perfectly understood but PVR>>SVR, and with pre-existing right ventricular outflow tract obstruction, decreased flow to the lungs, causing cyanosis. These are often exacerbated by fussiness, crying or defecating (all of these can transiently decrease arterial O2 saturation.)

## Slide 57
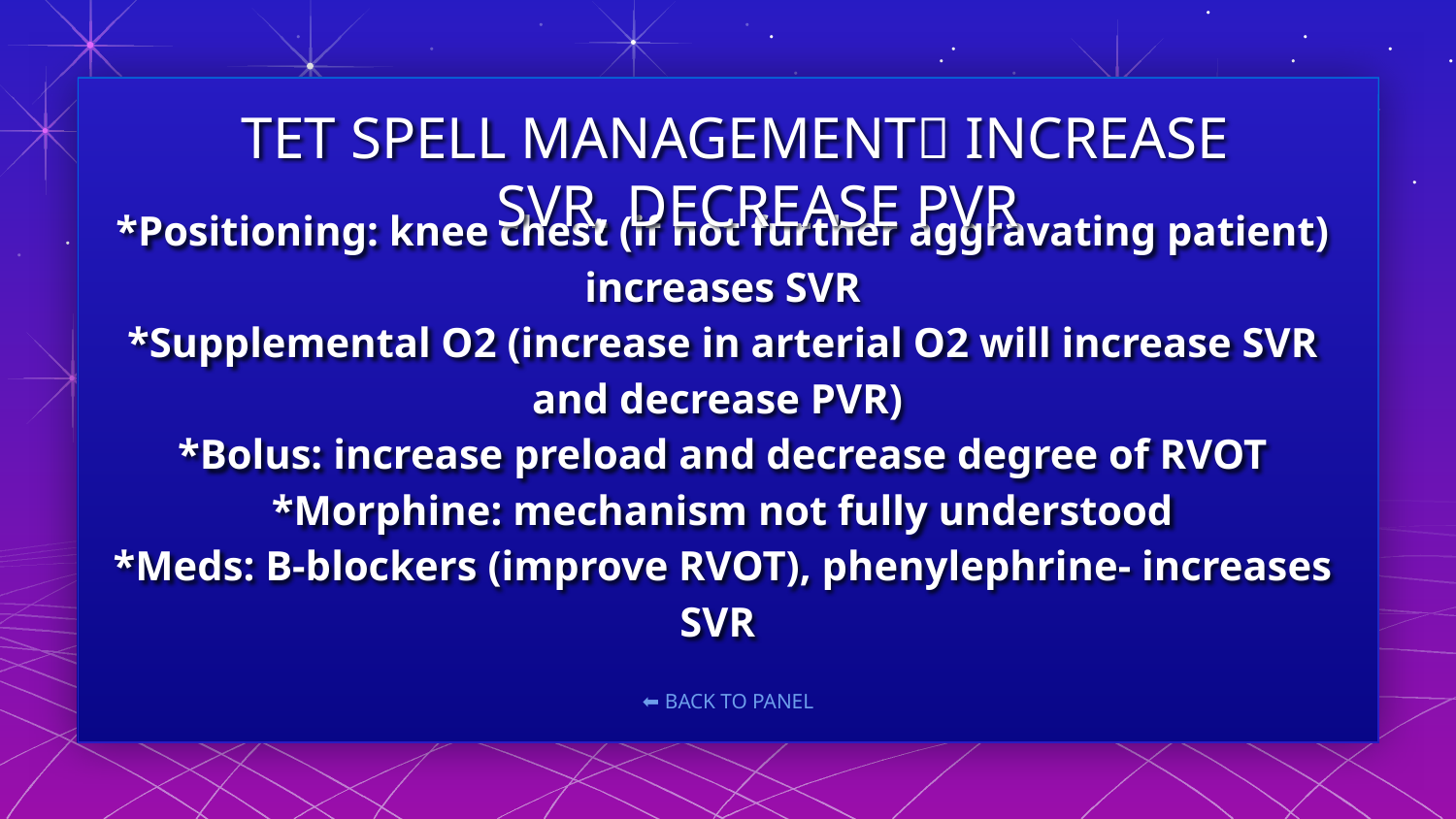

TET SPELL MANAGEMENT INCREASE SVR, DECREASE PVR
# *Positioning: knee chest (if not further aggravating patient) increases SVR *Supplemental O2 (increase in arterial O2 will increase SVR and decrease PVR) *Bolus: increase preload and decrease degree of RVOT *Morphine: mechanism not fully understood *Meds: B-blockers (improve RVOT), phenylephrine- increases SVR

## Slide 58
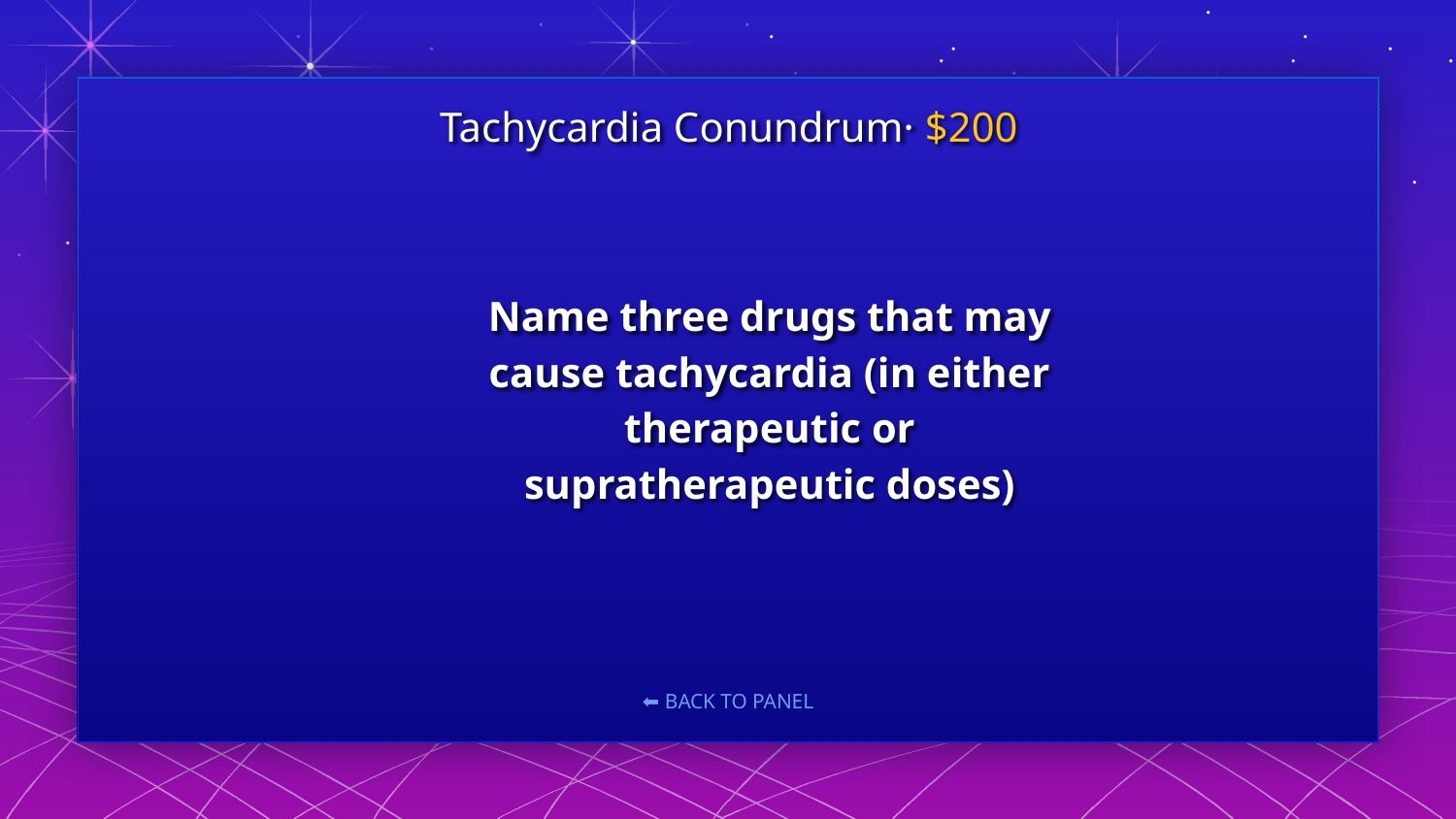

Tachycardia Conundrum· $200
# Name three drugs that may cause tachycardia (in either therapeutic or supratherapeutic doses)

## Slide 59
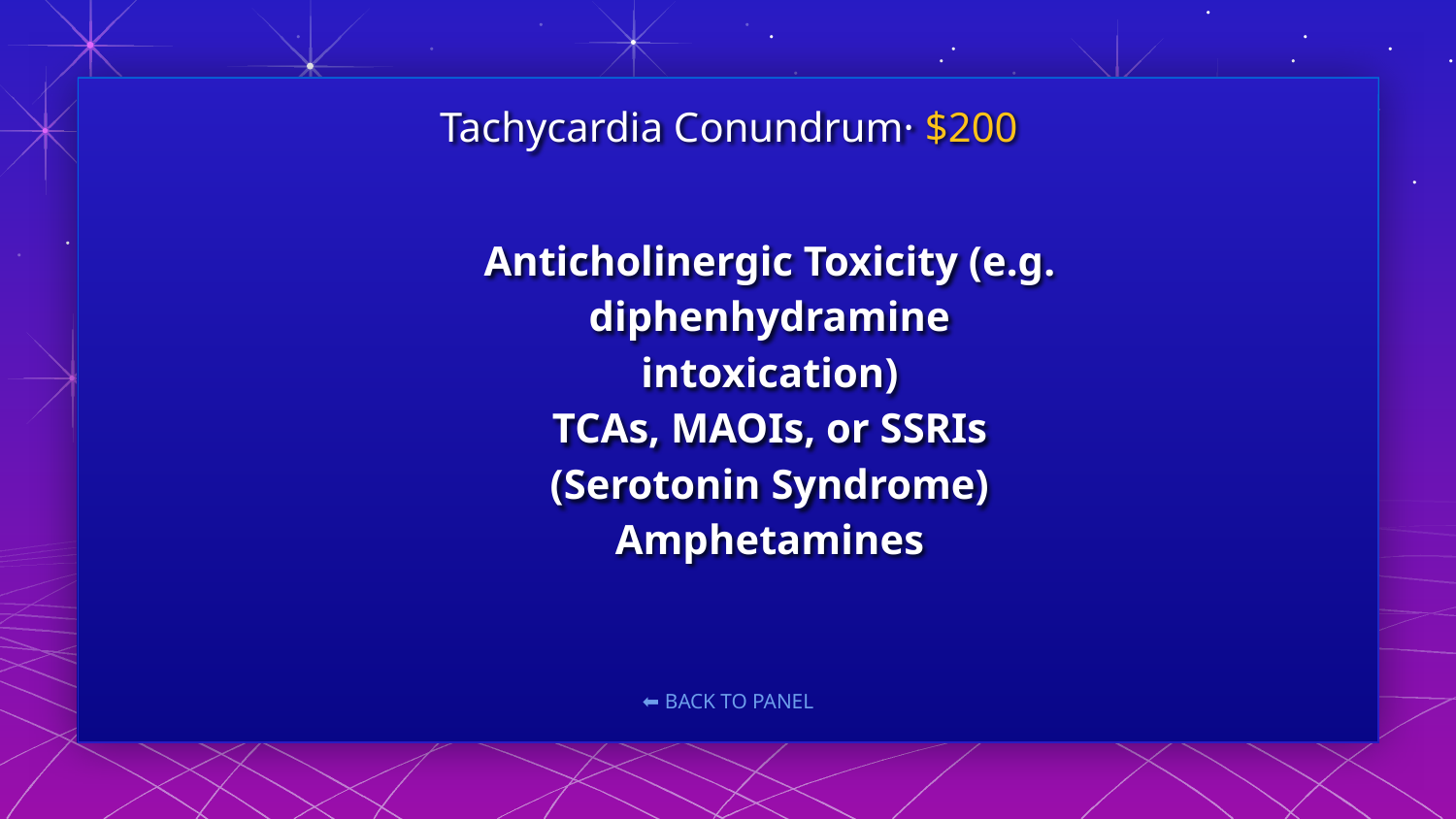

Tachycardia Conundrum· $200
# Anticholinergic Toxicity (e.g. diphenhydramine intoxication) TCAs, MAOIs, or SSRIs (Serotonin Syndrome) Amphetamines

## Slide 60
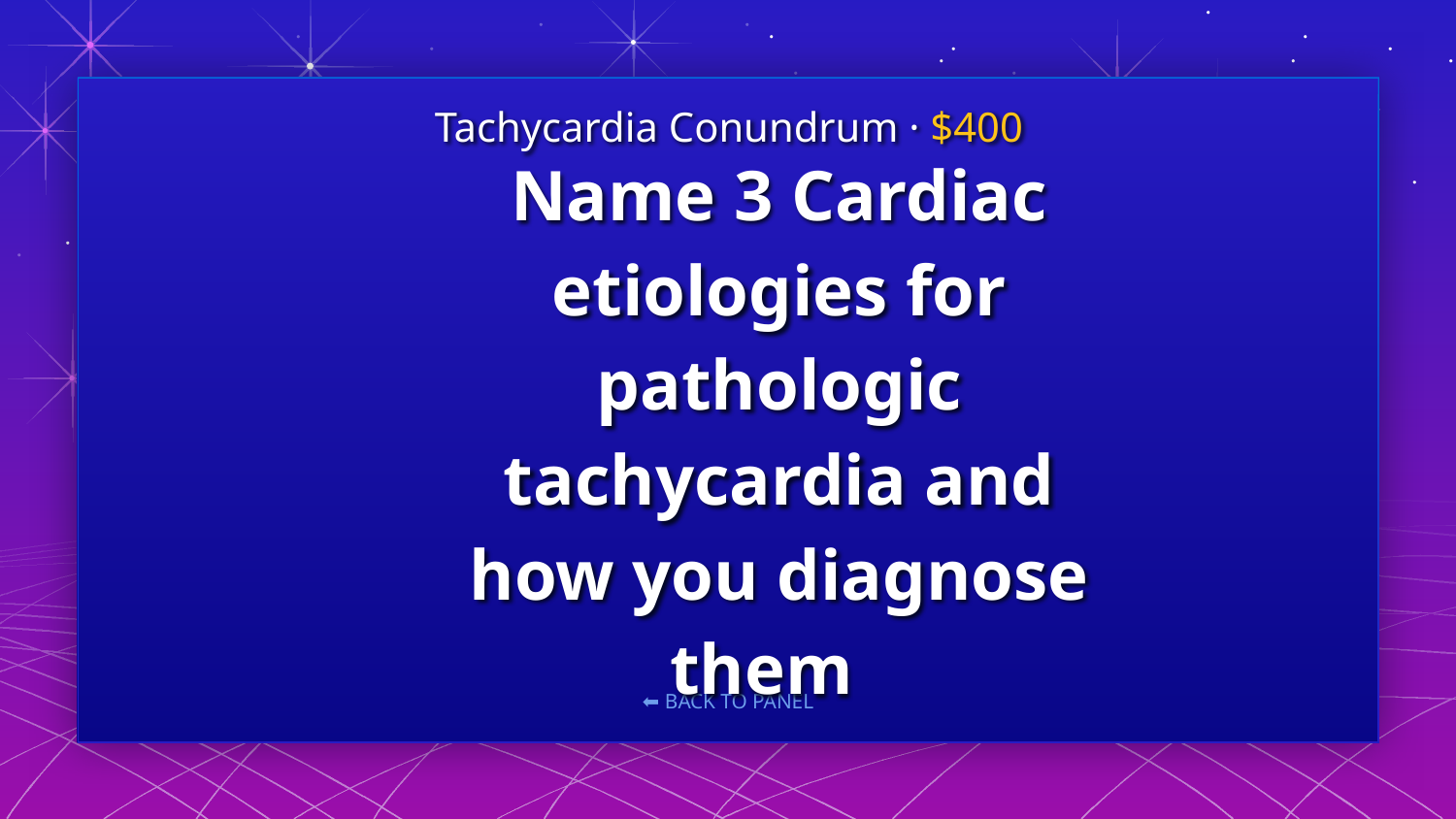

Tachycardia Conundrum · $400
# Name 3 Cardiac etiologies for pathologic tachycardia and how you diagnose them

## Slide 61
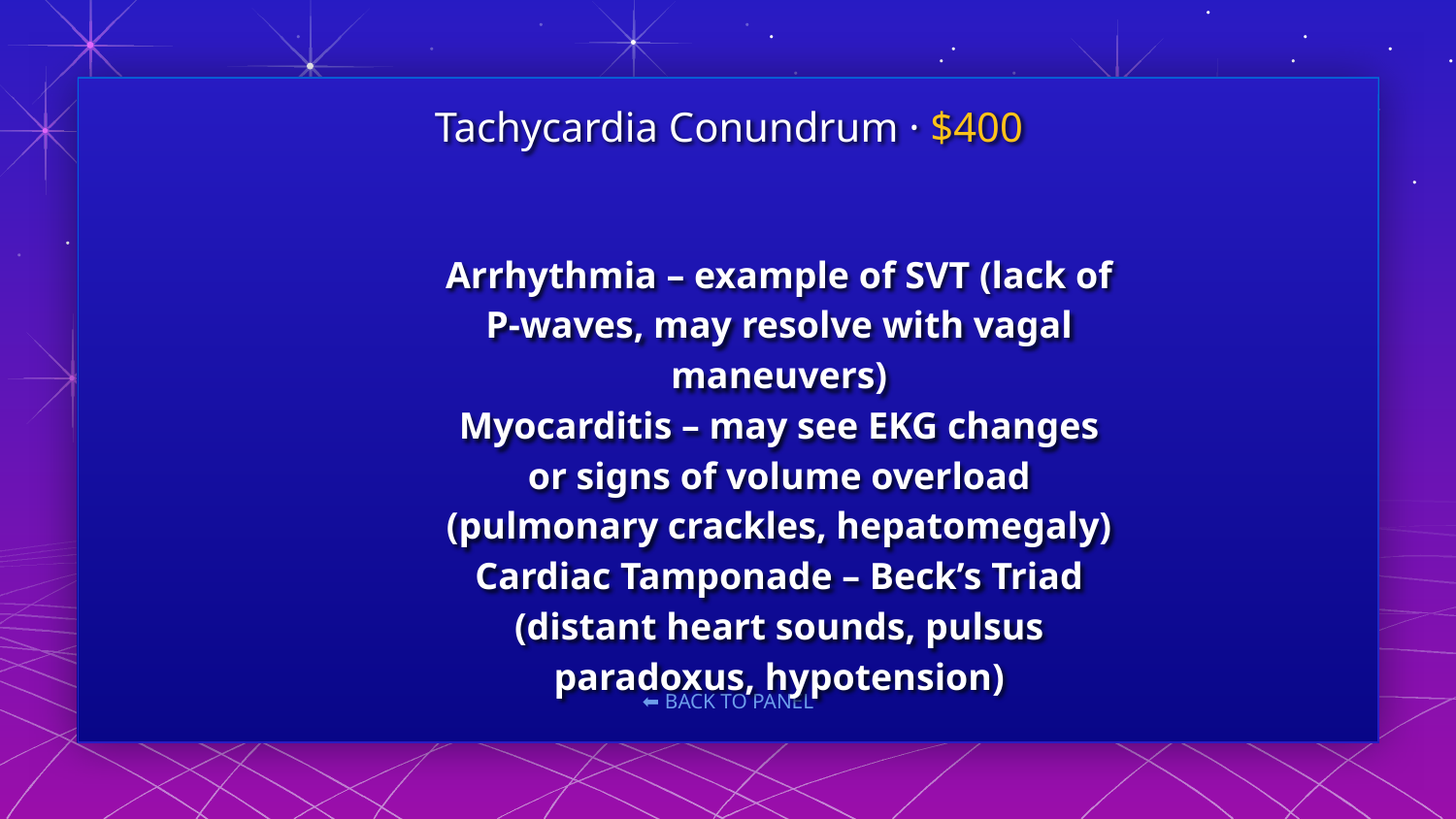

Tachycardia Conundrum · $400
# Arrhythmia – example of SVT (lack of P-waves, may resolve with vagal maneuvers) Myocarditis – may see EKG changes or signs of volume overload (pulmonary crackles, hepatomegaly) Cardiac Tamponade – Beck’s Triad (distant heart sounds, pulsus paradoxus, hypotension)

## Slide 62
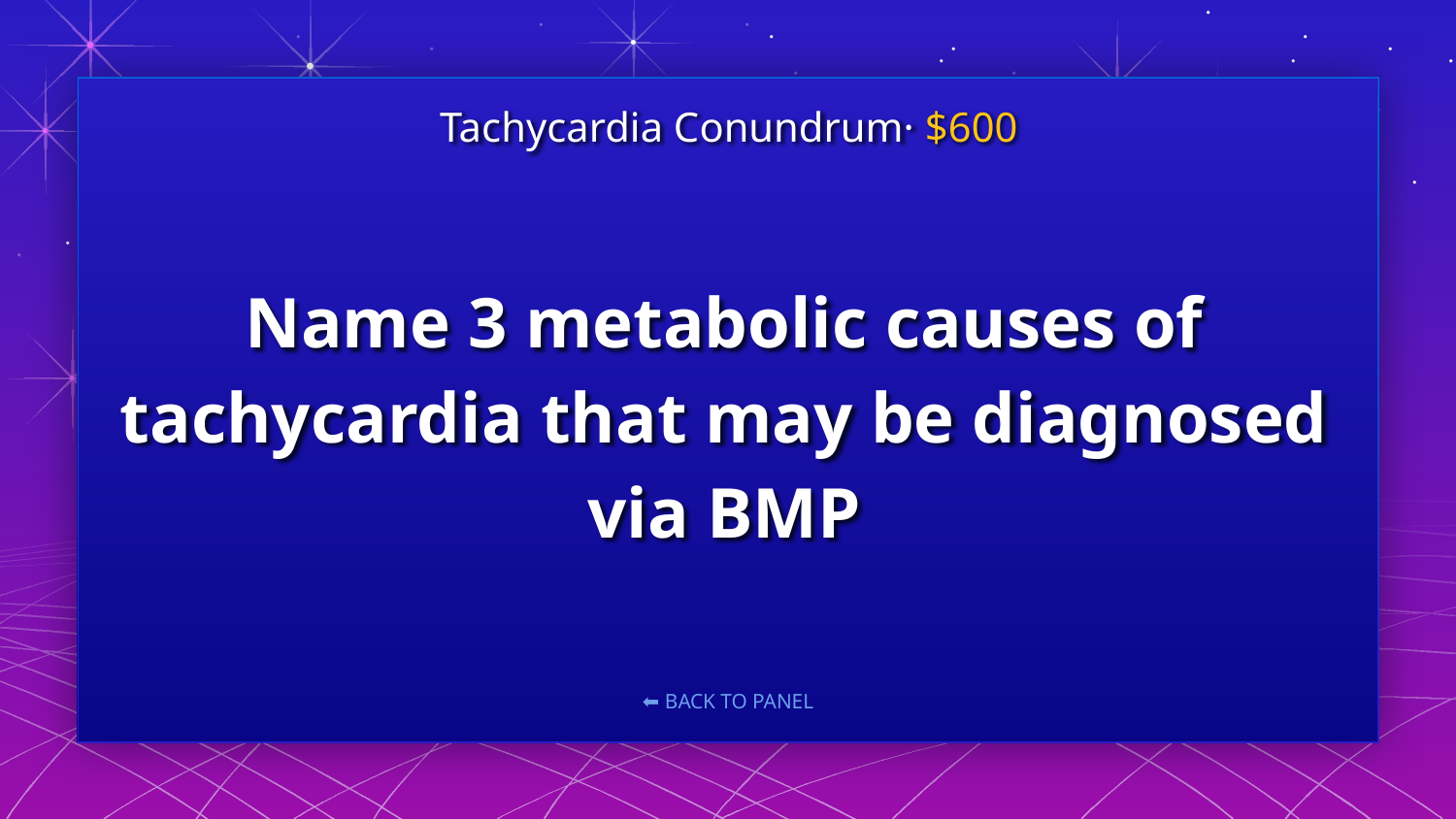

Tachycardia Conundrum· $600
# Name 3 metabolic causes of tachycardia that may be diagnosed via BMP

## Slide 63
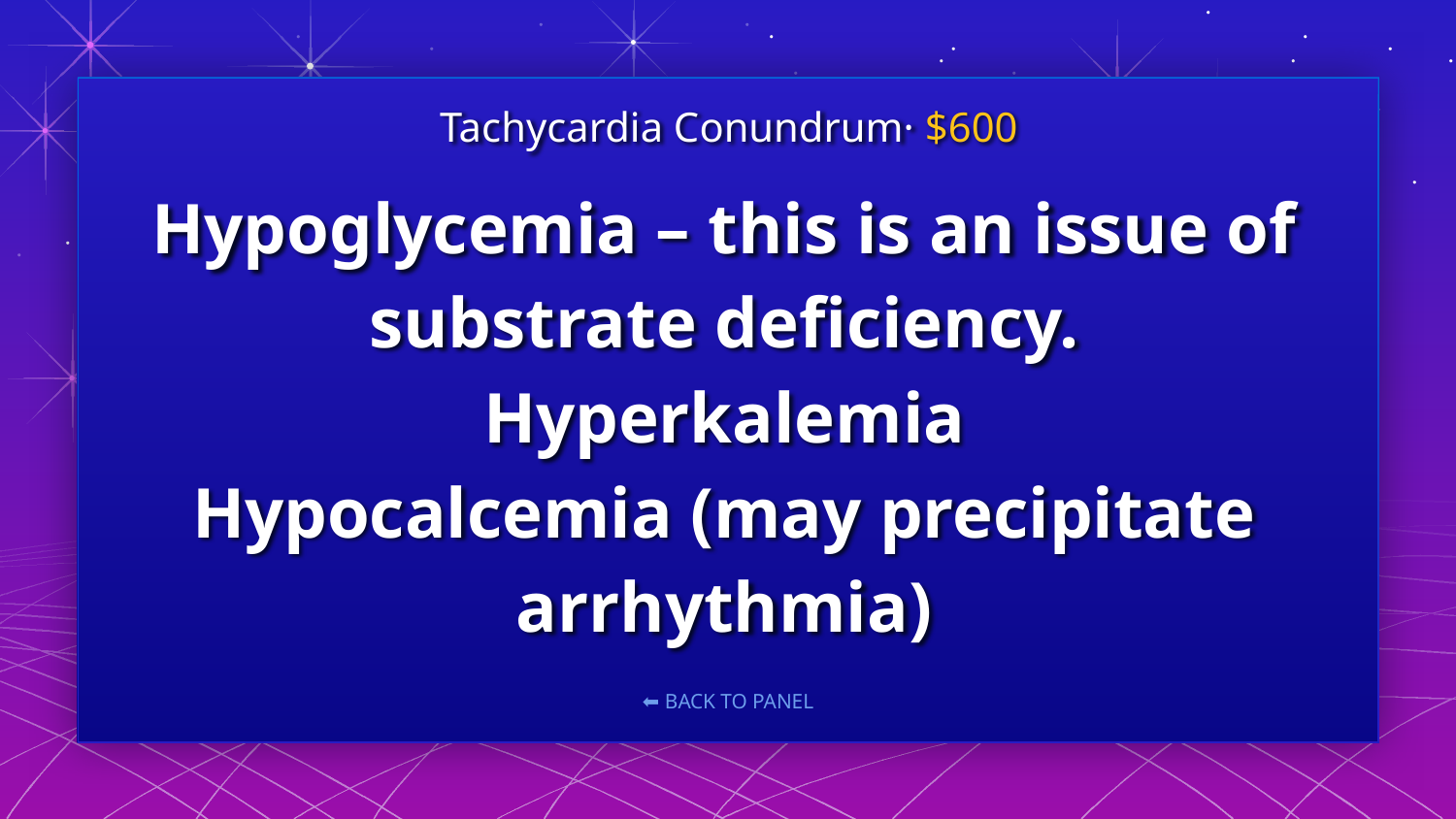

Tachycardia Conundrum· $600
# Hypoglycemia – this is an issue of substrate deficiency. Hyperkalemia Hypocalcemia (may precipitate arrhythmia)

## Slide 64
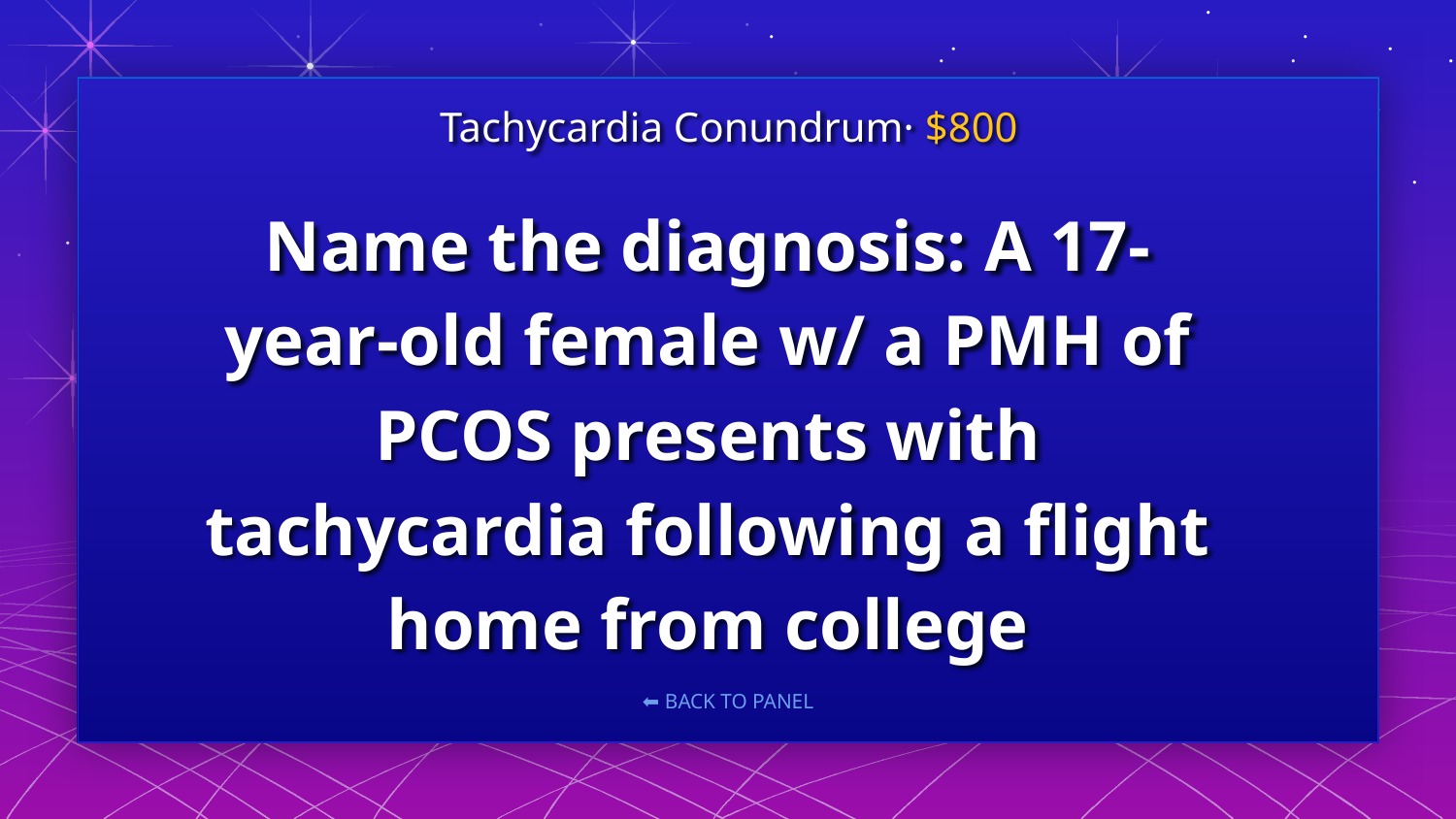

Tachycardia Conundrum· $800
# Name the diagnosis: A 17-year-old female w/ a PMH of PCOS presents with tachycardia following a flight home from college

## Slide 65
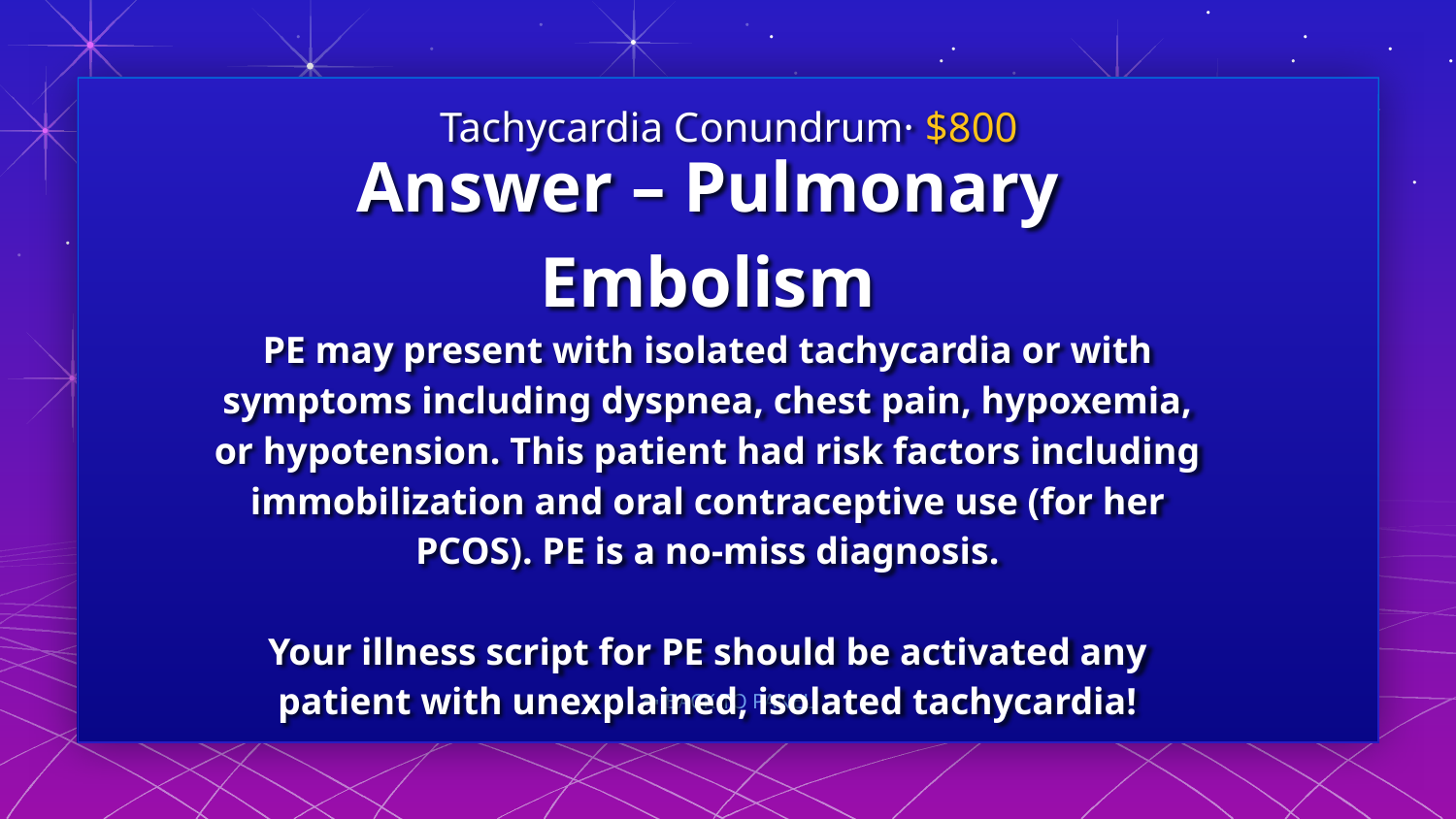

Tachycardia Conundrum· $800
# Answer – Pulmonary Embolism PE may present with isolated tachycardia or with symptoms including dyspnea, chest pain, hypoxemia, or hypotension. This patient had risk factors including immobilization and oral contraceptive use (for her PCOS). PE is a no-miss diagnosis. Your illness script for PE should be activated any patient with unexplained, isolated tachycardia!

## Slide 66
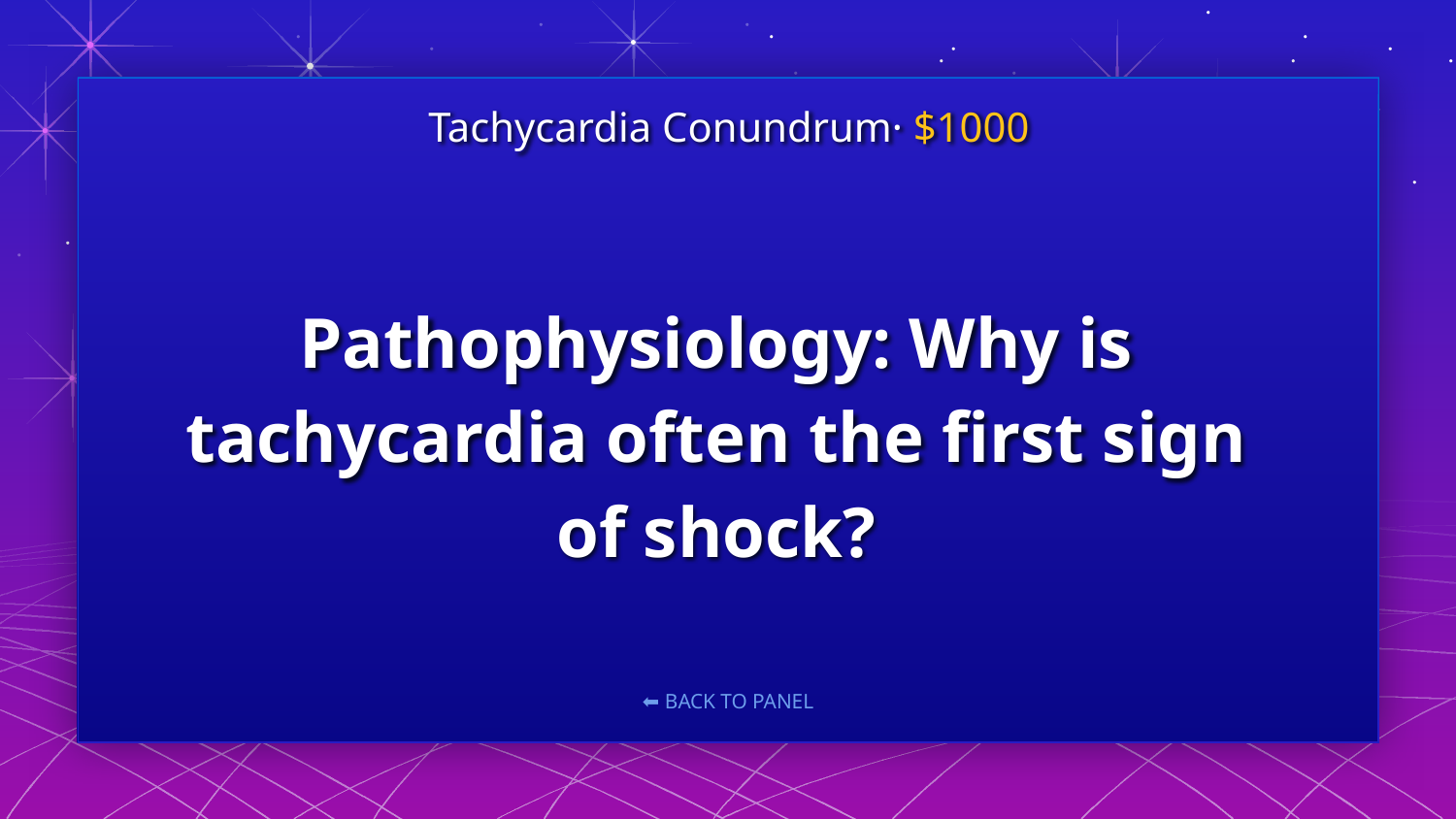

Tachycardia Conundrum· $1000
# Pathophysiology: Why is tachycardia often the first sign of shock?

## Slide 67
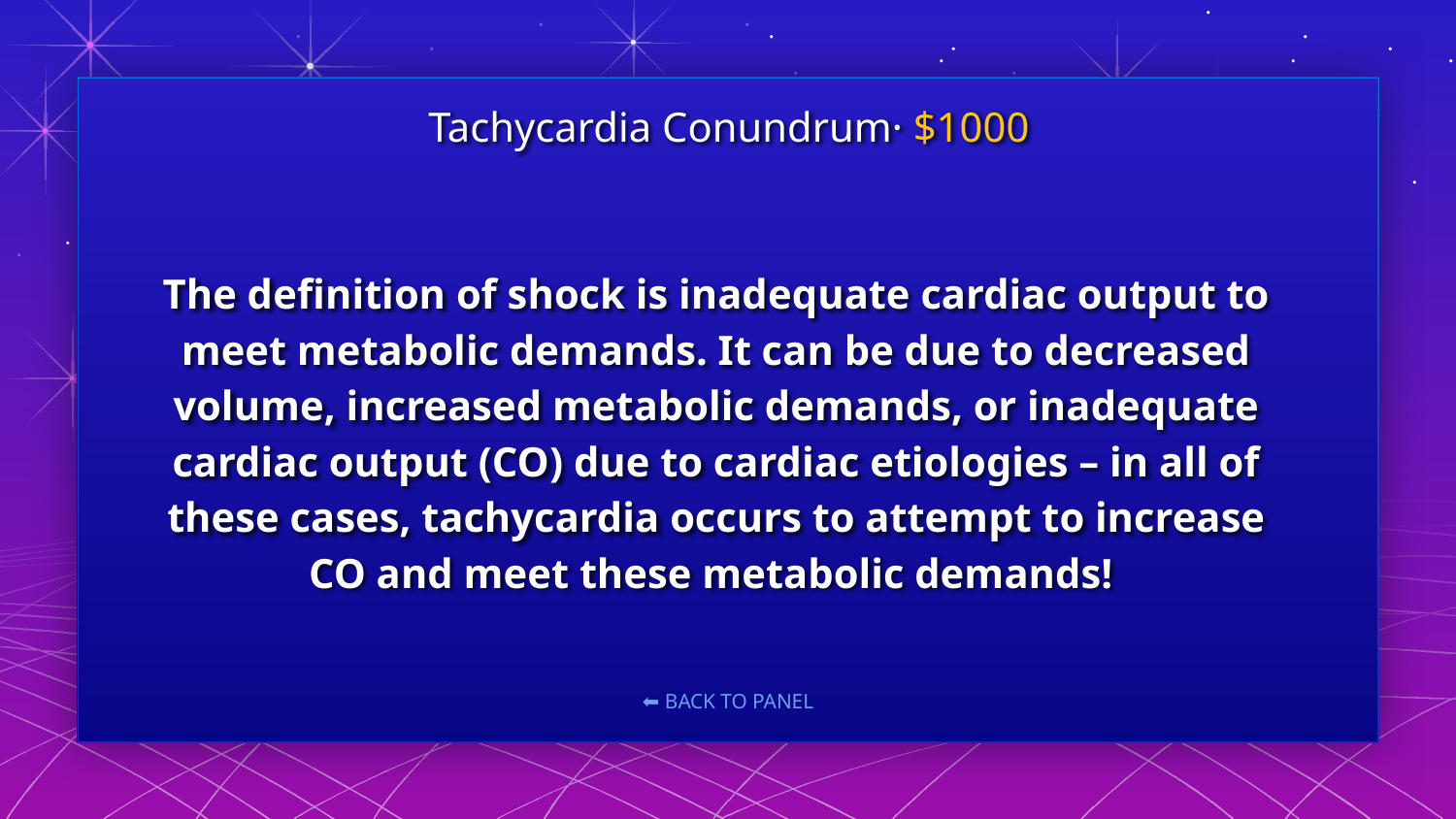

Tachycardia Conundrum· $1000
# The definition of shock is inadequate cardiac output to meet metabolic demands. It can be due to decreased volume, increased metabolic demands, or inadequate cardiac output (CO) due to cardiac etiologies – in all of these cases, tachycardia occurs to attempt to increase CO and meet these metabolic demands!

## Slide 68
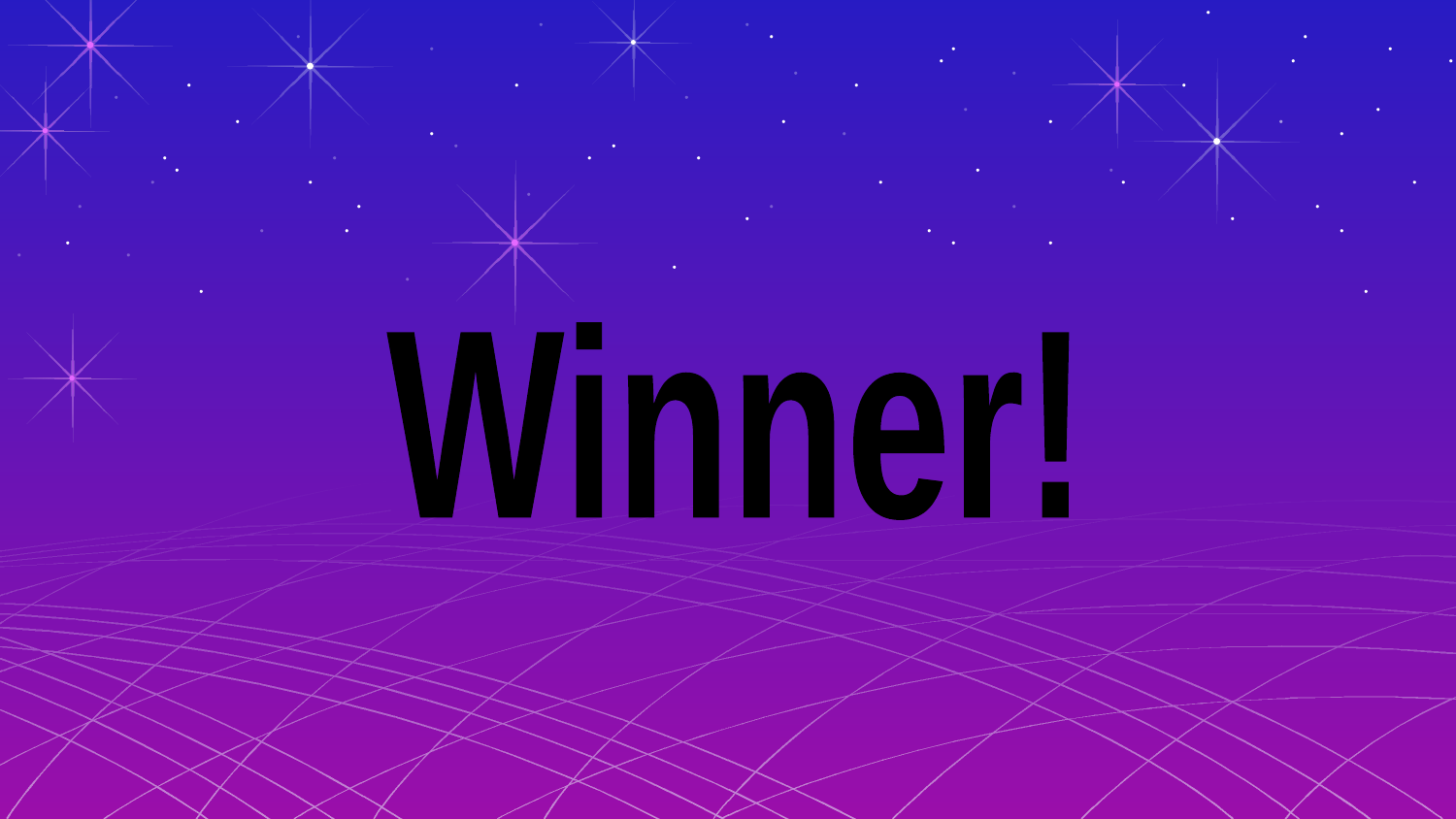

Winner!

## Slide 69
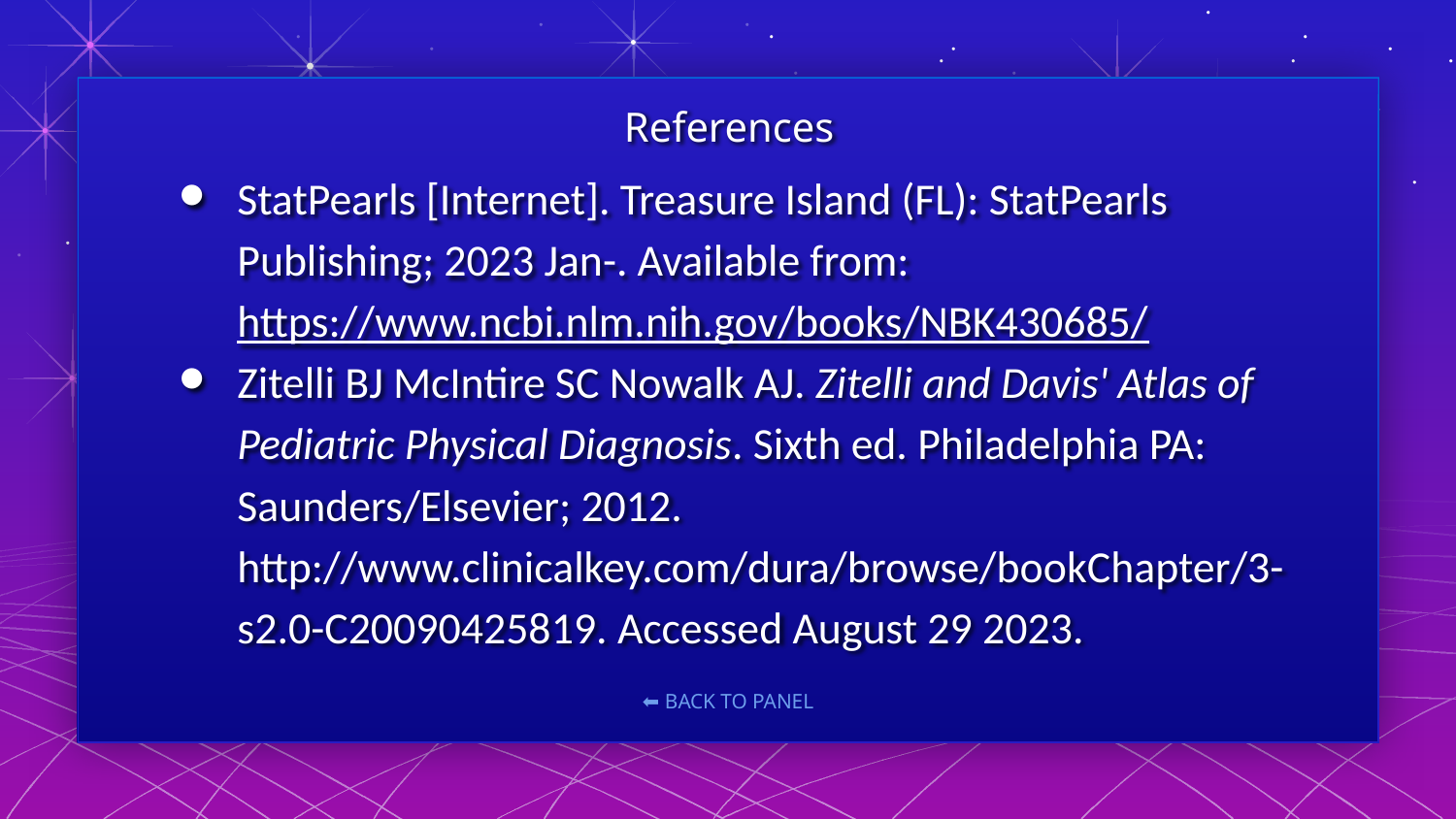

References
# StatPearls [Internet]. Treasure Island (FL): StatPearls Publishing; 2023 Jan-. Available from: https://www.ncbi.nlm.nih.gov/books/NBK430685/
Zitelli BJ McIntire SC Nowalk AJ. Zitelli and Davis' Atlas of Pediatric Physical Diagnosis. Sixth ed. Philadelphia PA: Saunders/Elsevier; 2012. http://www.clinicalkey.com/dura/browse/bookChapter/3-s2.0-C20090425819. Accessed August 29 2023.
