# Supplementary material for: A Pilot Longitudinal Clinical Reasoning Curriculum for Pediatric Residents
Source: MedEdPORTAL. 2024 Sep 25;20:11447. doi: 10.15766/mep_2374-8265.11447 (PMC11422513; doi:10.15766/mep_2374-8265.11447)
Supplement: Supplementary file 1 — Preimplementation Survey.docxCurriculum Goals, Objectives, and Timeline.docxSession 1 - Illness Scripts.pptxSession 1 - Small-Group Facilitator Guide.docxSession 2 - Illness Scripts 2.pptxSession 2 - Small-Group Facilitator Guide.docxSession 3 - Script Concordance.pptxSession 3 - Small-Group Facilitator Guide.docxSession 3 - Small-Group Handout.docxSession 4 - Pathophysiology.pptxSession 4 - Small-Group Facilitator Guide.docxSession 4 - Small-Group Handout.docxSession 5 - Review Game.pptxPostimplementation Survey.docx [file mep_2374-8265.11447-s001.zip › B. Curriculum Goals, Objectives, and Timeline.docx]

**Pediatric Advanced Clinical Reasoning Curriculum**

Curriculum Design, and Learning Objectives, and Session Outlines

Clinical reasoning encompasses skills highlighted in many of the Accreditation Council for Graduate Medical Education Milestones (subcompetencies) and the American Board of Pediatrics Enstrustable Professional Activities (EPAs). Content of the curriculum was mapped to milestones and EPAs that we felt most specifically focus on clinical reasoning, including:

- ACMGE Milestone: Medical knowledge 2 (MK2)
- ACGME Milestone: Patient care 4 (PC4)
- ABP EPA 4: Manage patients with acute, common diagnoses in an ambulatory, emergency, or inpatient setting.
- ABP EPA 11: Manage information from a variety of sources for both learning and application to patient care.

The following table outlines the overall curricular learning objectives, specific session topics and session learning objectives, and which subcompetencies or EPAs were highlighted.

| **Overall curricular learning objectives**   1. Identify common clinical reasoning skills in educational conferences and simulated encounters. 2. Apply common clinical reasoning skills in educational conferences. 3. Demonstrate clinical reasoning skills (e.g., using illness scripts, script concordance, and pathophysiology in clinical decision-making) in various simulated clinical environments. 4. Appraise clinical reasoning skills used in everyday clinical environments and in educational conferences. | | |
| --- | --- | --- |
| **Session Number and Topic** | **Sessions Learning Objectives**  **By the end of each individual session, participants will be able to:** | **Highlighted Subcompetencies and EPAs** |
| Session 1 - Illness Scripts Part 1  This session highlights clinical reasoning as a competency and illness scripts (Appendix C). Participants will use their own illness scripts to perform hypothesis-driven history-taking to make efficient, accurate diagnoses (Appendix D). | 1. Define the term “illness script.” 2. Implement the use of illness scripts to perform guided history-taking to reach a diagnostic hypothesis. 3. Weigh the risks and benefits of using illness scripts in both common and uncommon clinical presentations. | **MK 2, EPA 4** |
| Session 2 - Illness Scripts Part 2  This session expands on the concepts presented in session #1 to further illustrate the nuances of illness script theory (Appendix E). Participants will work as teams to build illness scripts for common and uncommon diagnoses (Appendix F). | 1. Define the terms “semantic qualifier” and “distinguishing feature” as they relate to the construction and use of illness scripts. 2. Implement reviewed clinical reasoning skills to build illness scripts for common and uncommon pediatric diagnoses. 3. Evaluate distinguishing features in common pediatric clinical presentations. 4. Identify differences in illness script structure/composition between common and uncommon diagnoses. | **MK 2, PC 4, EPA 4** |
| Session 3 - Script Concordance  This session highlights the use of script concordance in clinical reasoning (Appendix G). Participants will use skills gained in sessions #1 and #2 to perform script concordance in evaluating common pediatric presenting symptoms (Appendices H, I. | 1. Define the term “script concordance.” 2. Execute the skill of script concordance in the diagnostic process of common pediatric clinical presentations. | **PC 4, EPA 11** |
| Session 4 – Pathophysiology  This session shows pathophysiology use in clinical decision-making and the assessment and discussion of learner clinical reasoning (Appendix J). Participants will use pathophysiologic concepts to evaluate common clinical scenarios (Appendices K, L) | 1. Describe the use of pathophysiology in everyday clinical practice. 2. Relate common pathophysiologic processes to the diagnostic process of common pediatric clinical scenarios. | **MK 2, EPA 11** |
| Session 5 – Game Review  This session acts as a global review of the curriculum and uses gamification to promote adult learning (Appendix M). | 1. Execute common clinical reasoning skills to solve questions based on common pediatric clinical scenarios. | **All** |

The first four sessions of the curriculum are comprised of a didactic portion, where facilitators will use PowerPoint materials to review clinical reasoning concepts, followed by small group activities, where participants will practice the highlighted skills in a simulated environment. The following table outlines suggested flow and timing for each session.

| **Session** | **Suggested Time** |
| --- | --- |
| 1. **Illness Scripts Part 1**   **Didactic Material (Appendix C)**   - Curriculum overview (Slides 1-5) - Overview of clinical reasoning (Slides 6-14) - Illness Scripts – Definition, uses, benefits, and disadvantages (Slides 15-24)   **Small Group Activities**   - Explanation of activities (Slides 25-29) - Activity 1 **(Facilitator Guide Appendix D)** - Debrief Activity 1 - Activity 2 - Debrief session | **Didactic Material**  20 Minutes   - 2 minutes - 8 minutes - 10 minutes   **Small Group Activities**   - 1 Minute - 15 minutes - 4 minutes - 15 minutes - 5 minutes |
| **2. Illness Scripts Part 2**  **Didactic Material (Appendix E)**   - Review of curriculum and introduction (Slides 1-5) - Review definition and use of illness scripts (Slides 6-10) - Define and discuss distinguishing features (Slides 11-12) - Define and discuss semantic qualifiers (Slides 13) - Small group explanation (Slide 14-16)   **Small Group Activities**   - Activity 1 **(Facilitator Guide Appendix F)** - Debrief activity 1 - Activity 2 - Debrief activity 2, conclude session | **Didactic Material**  20 Minutes   - 4 minutes - 6 minutes - 4 minutes - 4 minutes - 2 minutes   **Small Group Activities**   - 10 minutes - 8 minutes - 10 minutes - 12 minutes |
| **3. Script Concordance**  **Didactic Material (Appendix G)**   - Review curriculum and learning objectives (Slides 1-8) - Review illness scripts (Slides 9-12) - Define and provide examples of script concordance (Slides 13-17)   **Small Group Activities**   - Explanation of activities (Slide 18-19) - Small group activities **(Facilitator Guide Appendix H, Small Group Handout Appendix I)** - Debrief session | **Didactic Material**  20 minutes   - 3 minutes - 8 minutes - 9 minutes   **Small Group Activities**   - 1 minute - 29 minutes - 10 minutes |
| **4. Pathophysiology**  **Didactic Material (Appendix J)**   - Review of previous sessions (Slides 1-5) - Pathophysiology and clinical reasoning (Slides 6-15) - Examples of pathophysiology in clinical context (Slides 16-22)   **Small Group Activities**   - Explanation of activities (Slide 23-24) - Small group activities **(Facilitator Guide Appendix K, Small Group Handout Appendix L)** - Debrief session | **Didactic Material**  20 minutes   - 3 minutes - 10 minutes - 7 minutes   **Small Group Activities**   - 1 minute - 39 minutes - 10 minutes |
| **5. Review**  **Didactic Material (Appendix M)**   - Review curriculum (Slides 1-2) - Quiz Review Game | **Didactic Material**   - 10 minutes - 50 minutes |
