# Supplementary material for: A Pilot Longitudinal Clinical Reasoning Curriculum for Pediatric Residents
Source: MedEdPORTAL. 2024 Sep 25;20:11447. doi: 10.15766/mep_2374-8265.11447 (PMC11422513; doi:10.15766/mep_2374-8265.11447)
Supplement: Supplementary file 1 — Preimplementation Survey.docxCurriculum Goals, Objectives, and Timeline.docxSession 1 - Illness Scripts.pptxSession 1 - Small-Group Facilitator Guide.docxSession 2 - Illness Scripts 2.pptxSession 2 - Small-Group Facilitator Guide.docxSession 3 - Script Concordance.pptxSession 3 - Small-Group Facilitator Guide.docxSession 3 - Small-Group Handout.docxSession 4 - Pathophysiology.pptxSession 4 - Small-Group Facilitator Guide.docxSession 4 - Small-Group Handout.docxSession 5 - Review Game.pptxPostimplementation Survey.docx [file mep_2374-8265.11447-s001.zip › C. Session 1 - Illness Scripts.pptx]

## Slide 1
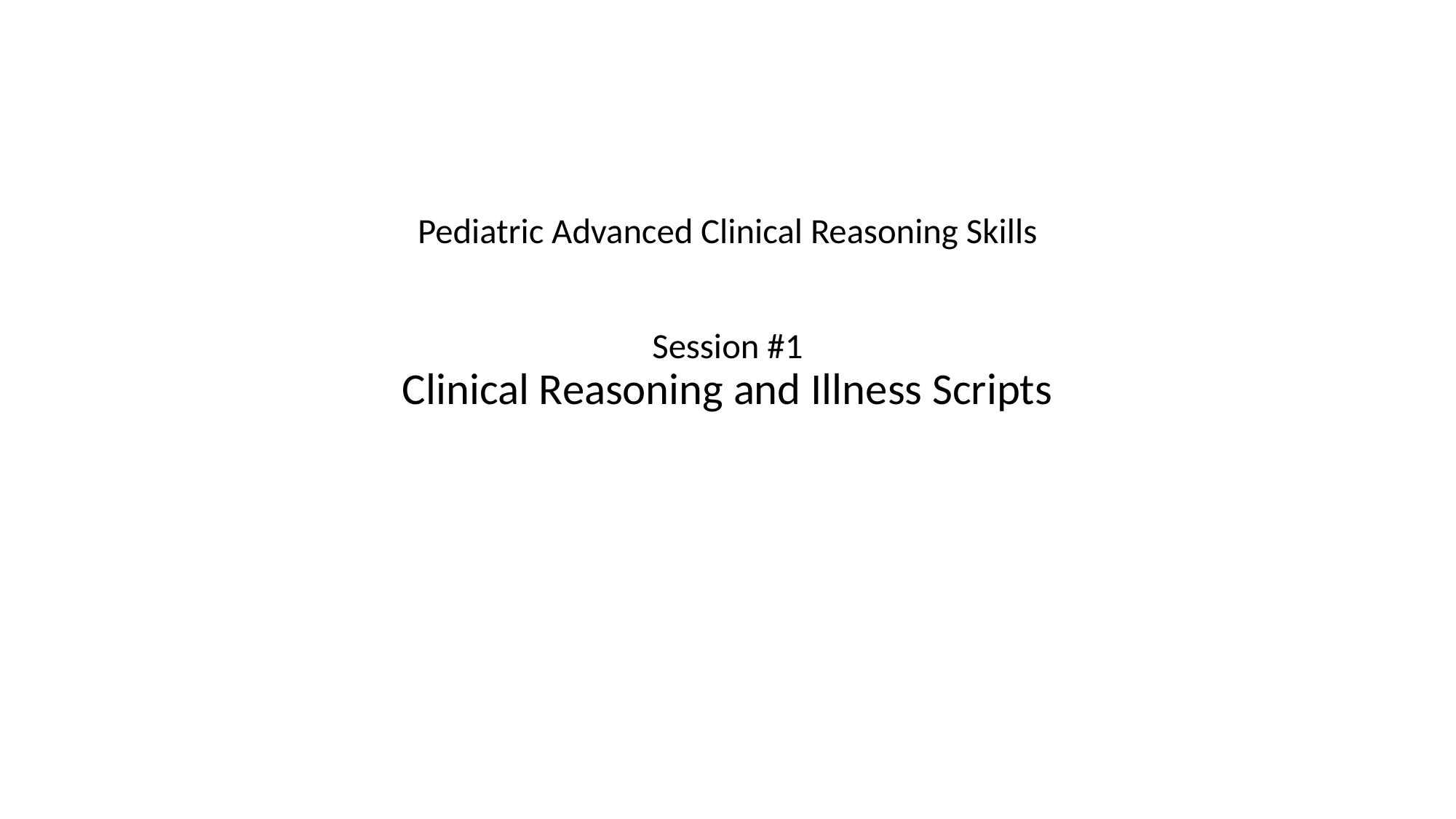

# Pediatric Advanced Clinical Reasoning Skills Session #1 Clinical Reasoning and Illness Scripts

## Slide 2
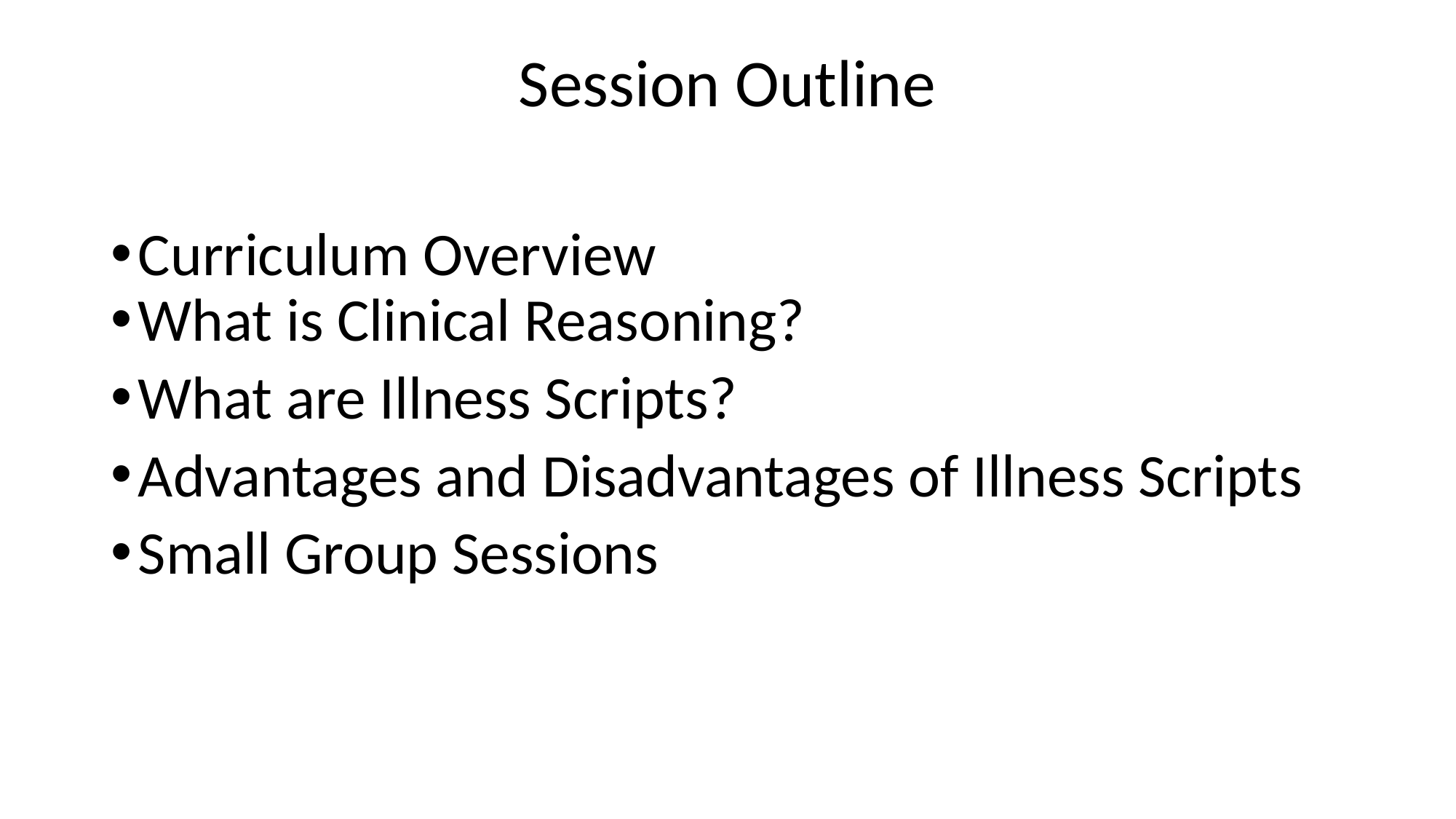

# Session Outline
Curriculum Overview
What is Clinical Reasoning?
What are Illness Scripts?
Advantages and Disadvantages of Illness Scripts
Small Group Sessions

## Slide 3
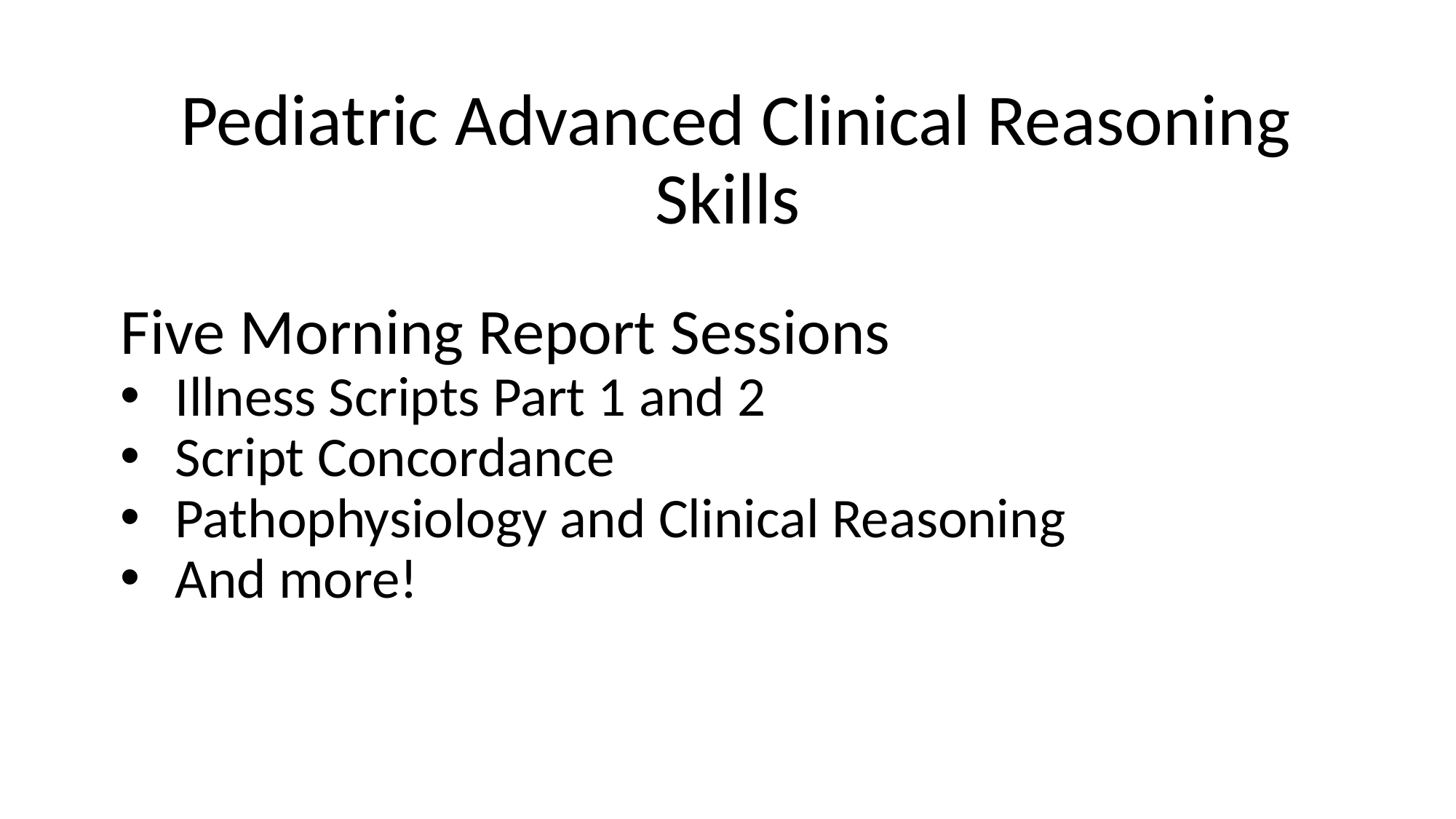

# Pediatric Advanced Clinical Reasoning Skills
Five Morning Report Sessions
Illness Scripts Part 1 and 2
Script Concordance
Pathophysiology and Clinical Reasoning
And more!

## Slide 4
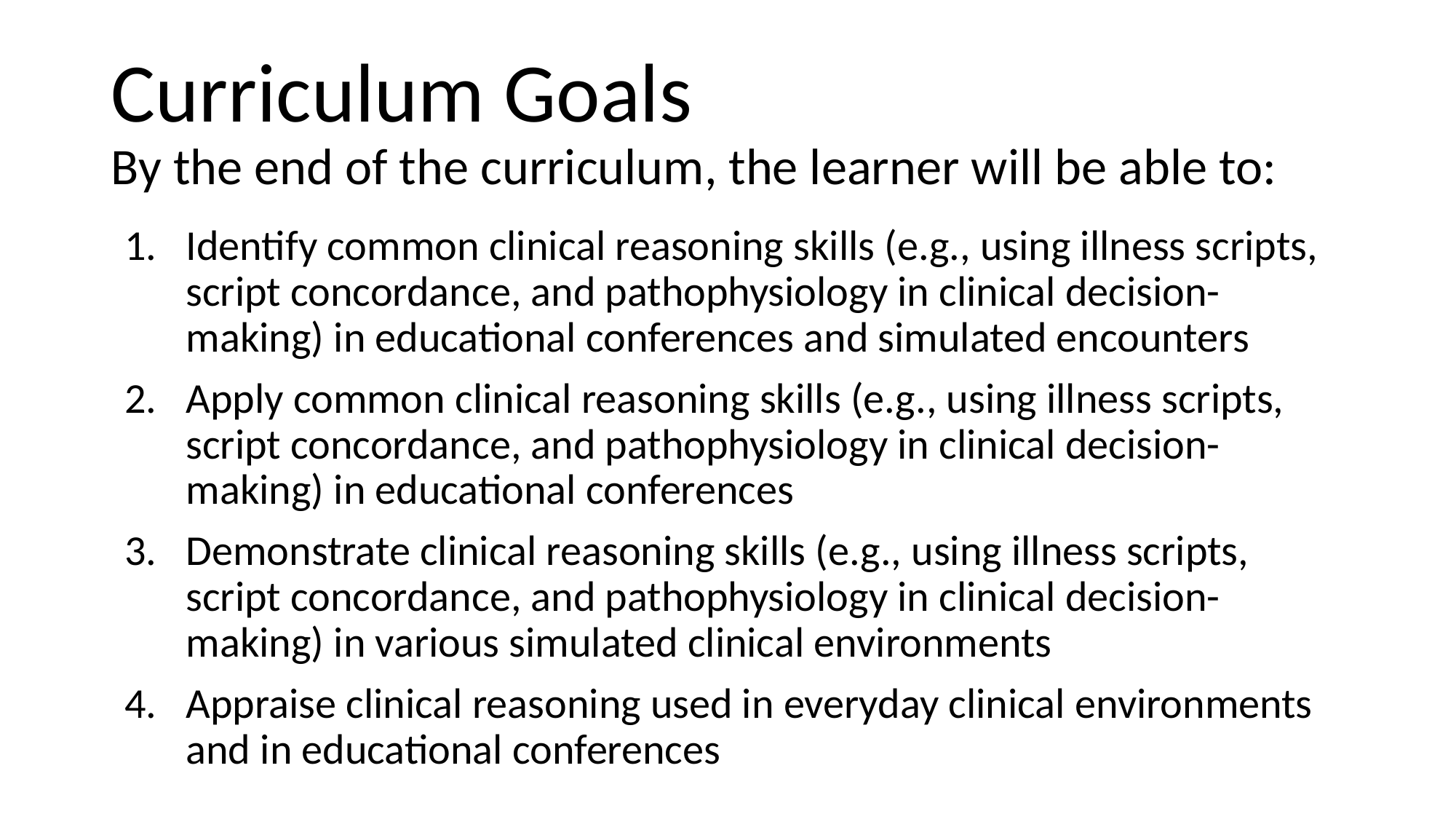

# Curriculum Goals By the end of the curriculum, the learner will be able to:
Identify common clinical reasoning skills (e.g., using illness scripts, script concordance, and pathophysiology in clinical decision-making) in educational conferences and simulated encounters
Apply common clinical reasoning skills (e.g., using illness scripts, script concordance, and pathophysiology in clinical decision-making) in educational conferences
Demonstrate clinical reasoning skills (e.g., using illness scripts, script concordance, and pathophysiology in clinical decision-making) in various simulated clinical environments
Appraise clinical reasoning used in everyday clinical environments and in educational conferences

## Slide 5
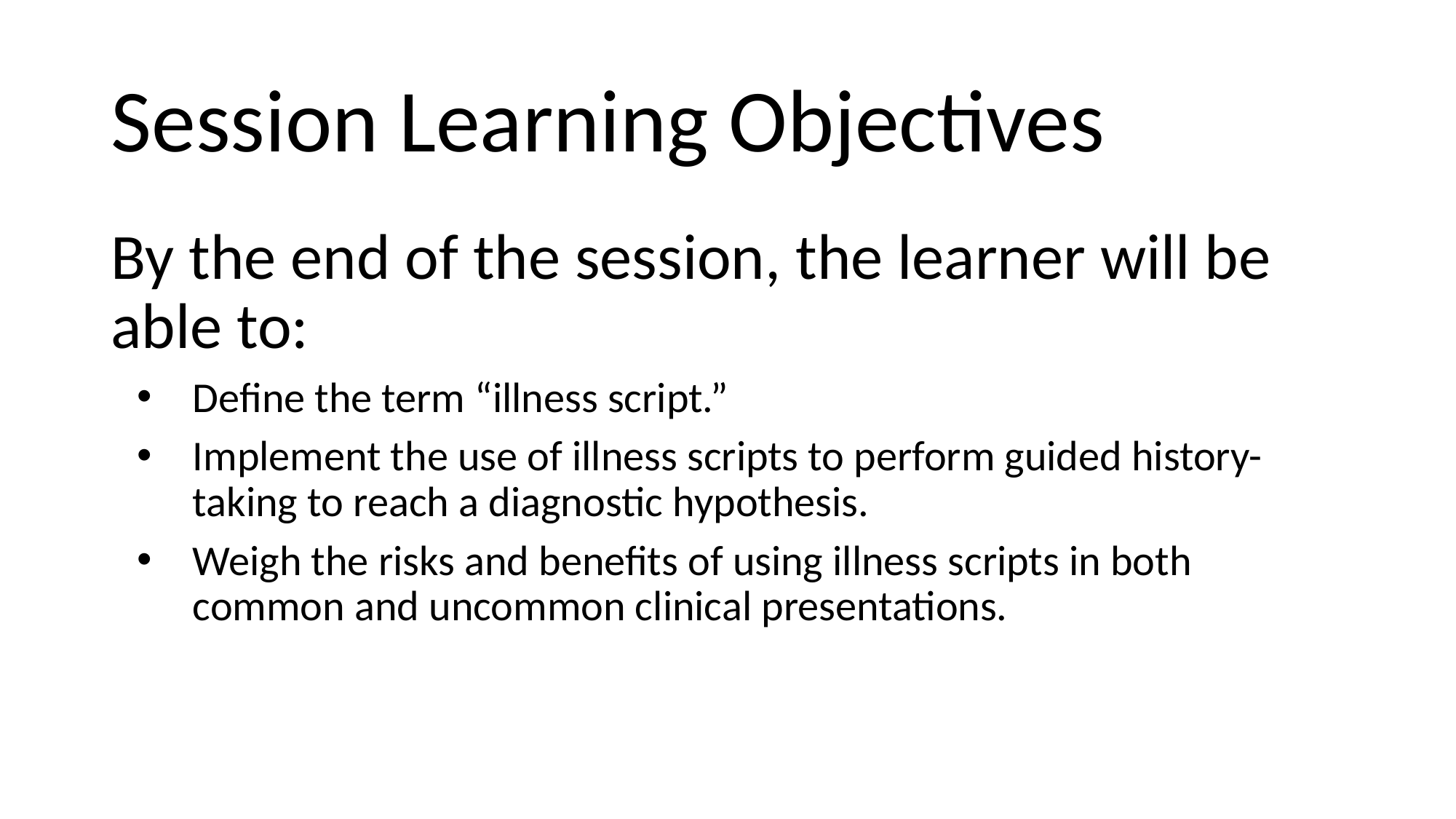

# Session Learning Objectives
By the end of the session, the learner will be able to:
Define the term “illness script.”
Implement the use of illness scripts to perform guided history-taking to reach a diagnostic hypothesis.
Weigh the risks and benefits of using illness scripts in both common and uncommon clinical presentations.

## Slide 6
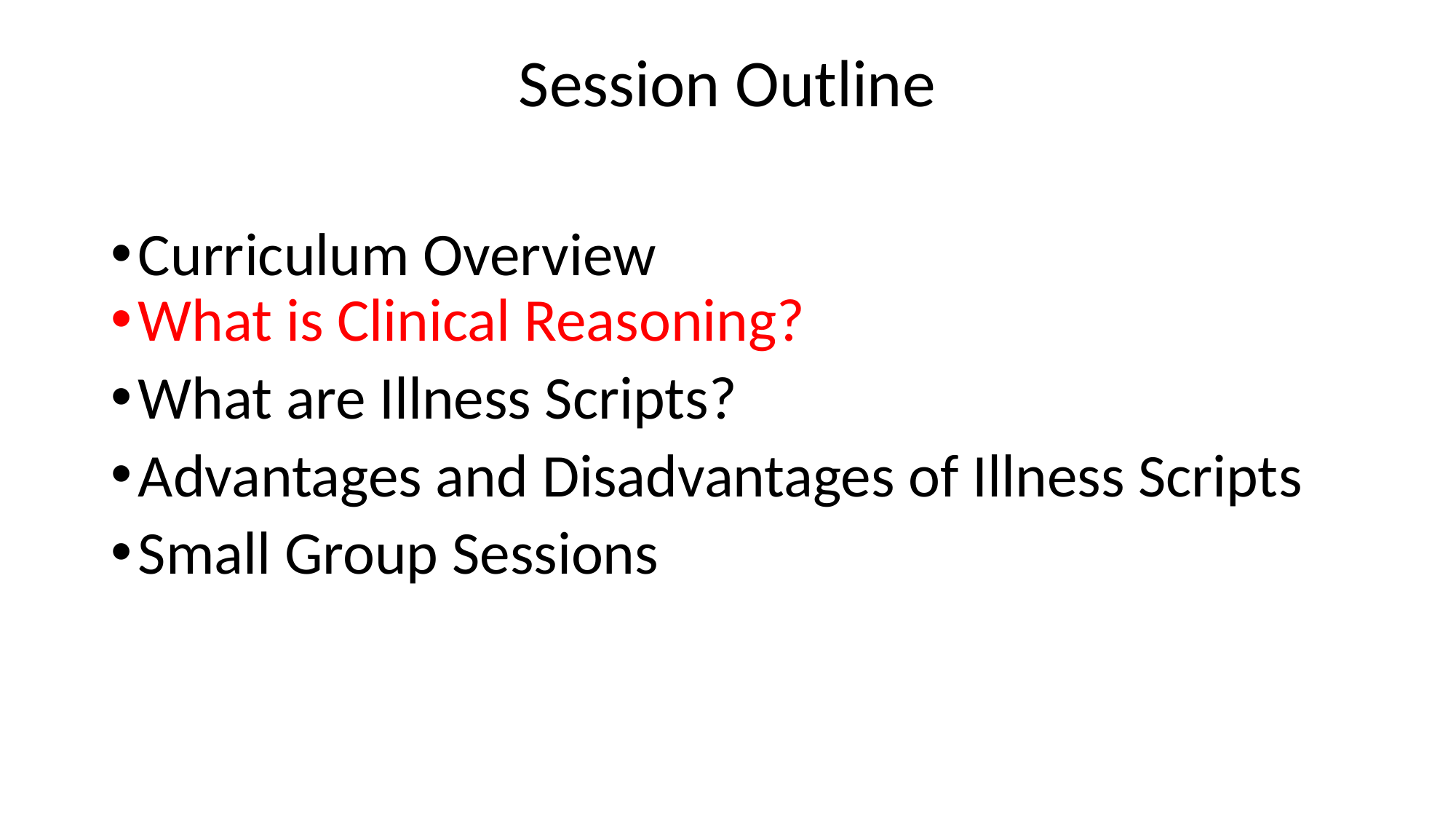

# Session Outline
Curriculum Overview
What is Clinical Reasoning?
What are Illness Scripts?
Advantages and Disadvantages of Illness Scripts
Small Group Sessions

## Slide 7
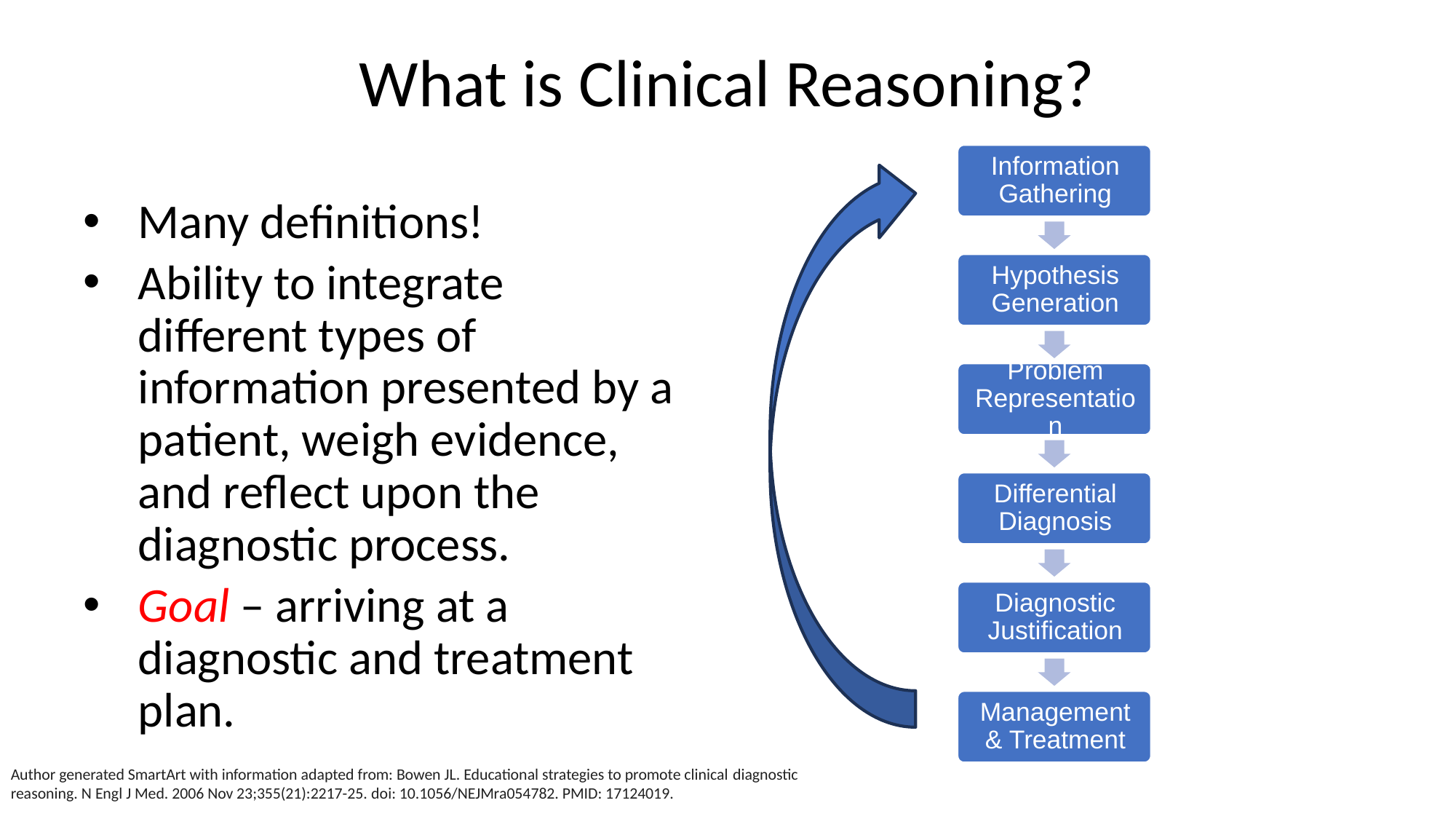

# What is Clinical Reasoning?
Many definitions!
Ability to integrate different types of information presented by a patient, weigh evidence, and reflect upon the diagnostic process.
Goal – arriving at a diagnostic and treatment plan.
Author generated SmartArt with information adapted from: Bowen JL. Educational strategies to promote clinical diagnostic reasoning. N Engl J Med. 2006 Nov 23;355(21):2217-25. doi: 10.1056/NEJMra054782. PMID: 17124019.​

## Slide 8
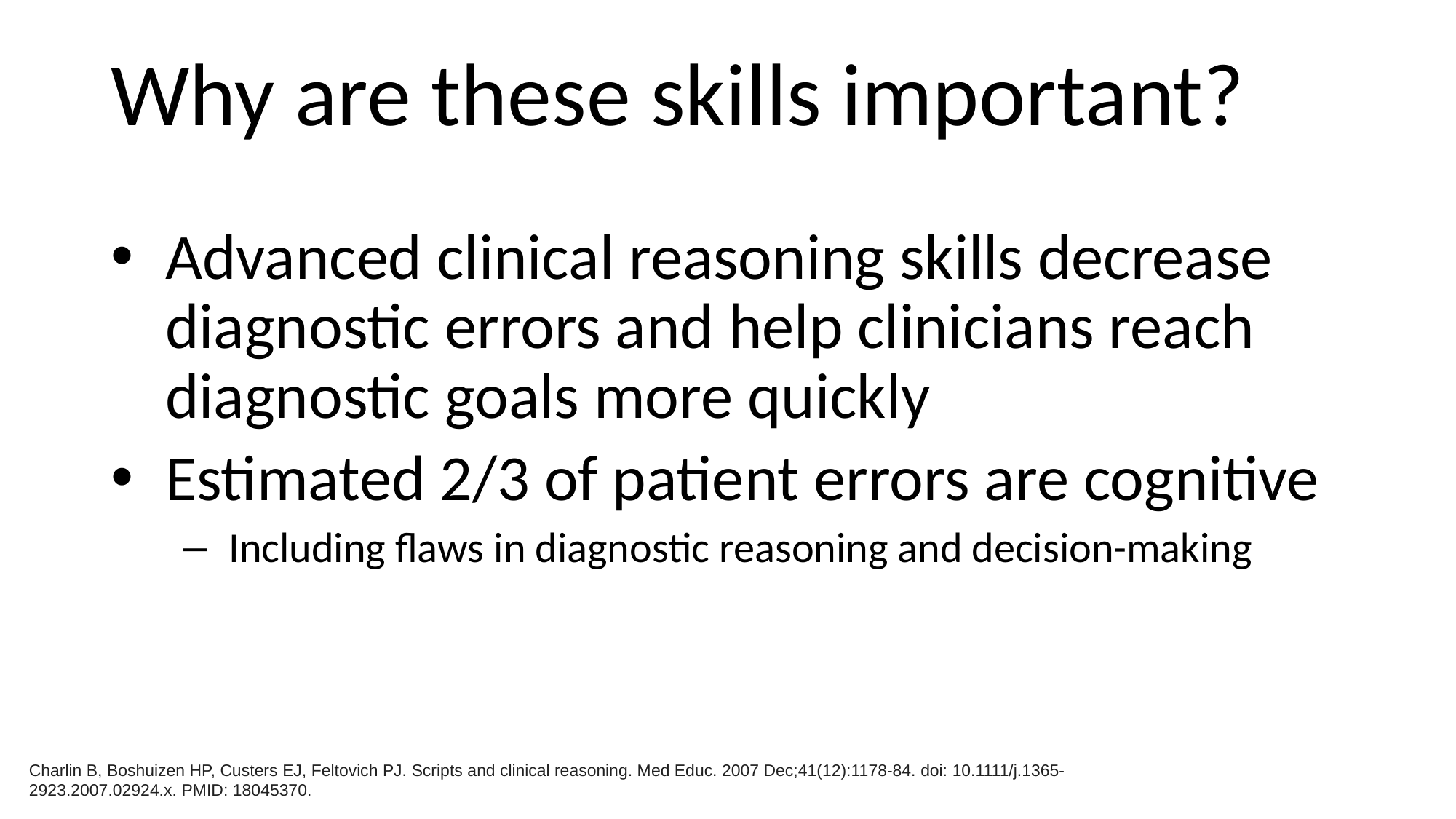

# Why are these skills important?
Advanced clinical reasoning skills decrease diagnostic errors and help clinicians reach diagnostic goals more quickly
Estimated 2/3 of patient errors are cognitive
Including flaws in diagnostic reasoning and decision-making
Charlin B, Boshuizen HP, Custers EJ, Feltovich PJ. Scripts and clinical reasoning. Med Educ. 2007 Dec;41(12):1178-84. doi: 10.1111/j.1365-2923.2007.02924.x. PMID: 18045370.

## Slide 9
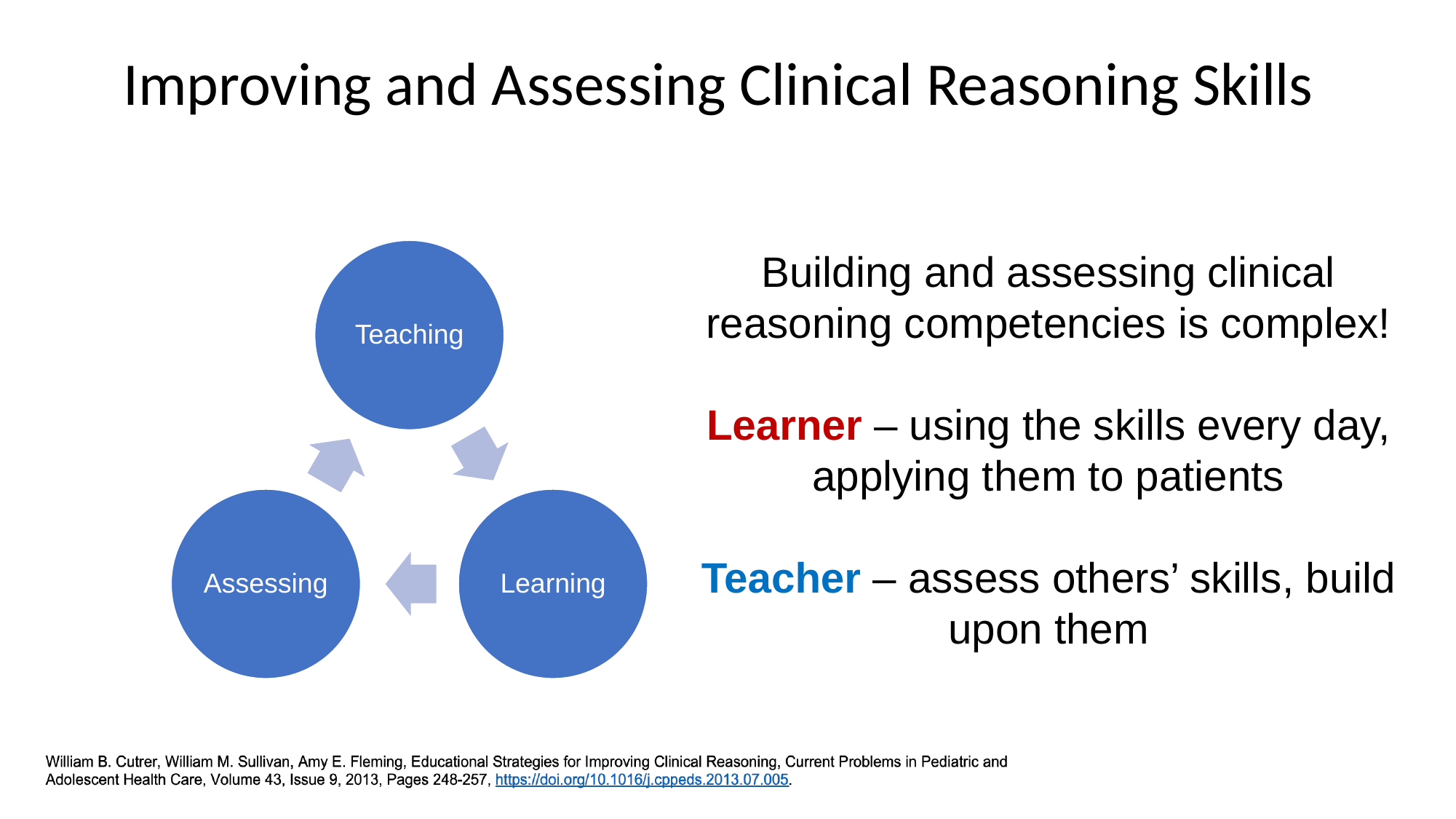

# Improving and Assessing Clinical Reasoning Skills
Building and assessing clinical reasoning competencies is complex!
Learner – using the skills every day, applying them to patients
Teacher – assess others’ skills, build upon them

## Slide 10
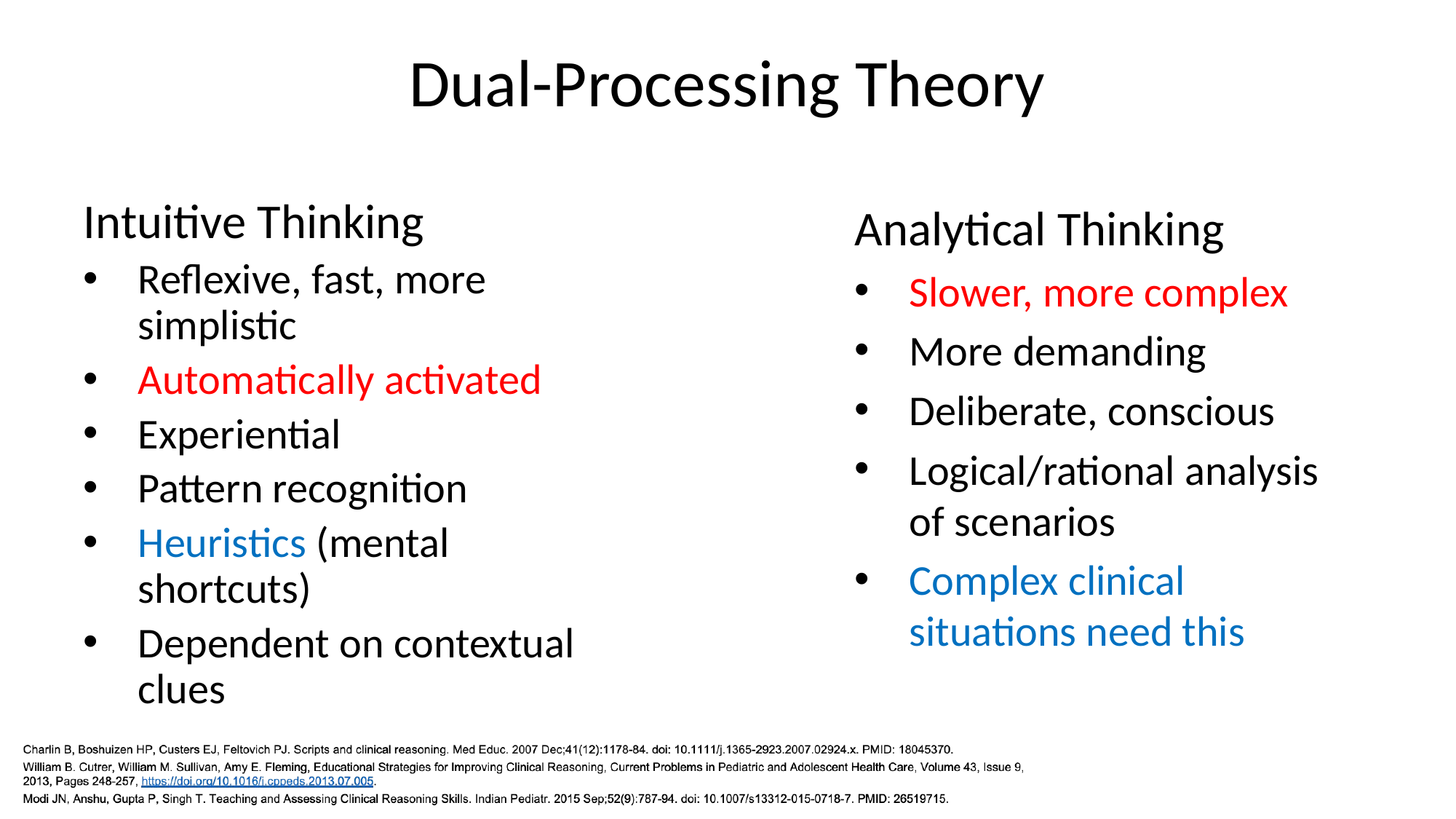

# Dual-Processing Theory
Intuitive Thinking
Reflexive, fast, more simplistic
Automatically activated
Experiential
Pattern recognition
Heuristics (mental shortcuts)
Dependent on contextual clues
Analytical Thinking
Slower, more complex
More demanding
Deliberate, conscious
Logical/rational analysis of scenarios
Complex clinical situations need this

## Slide 11
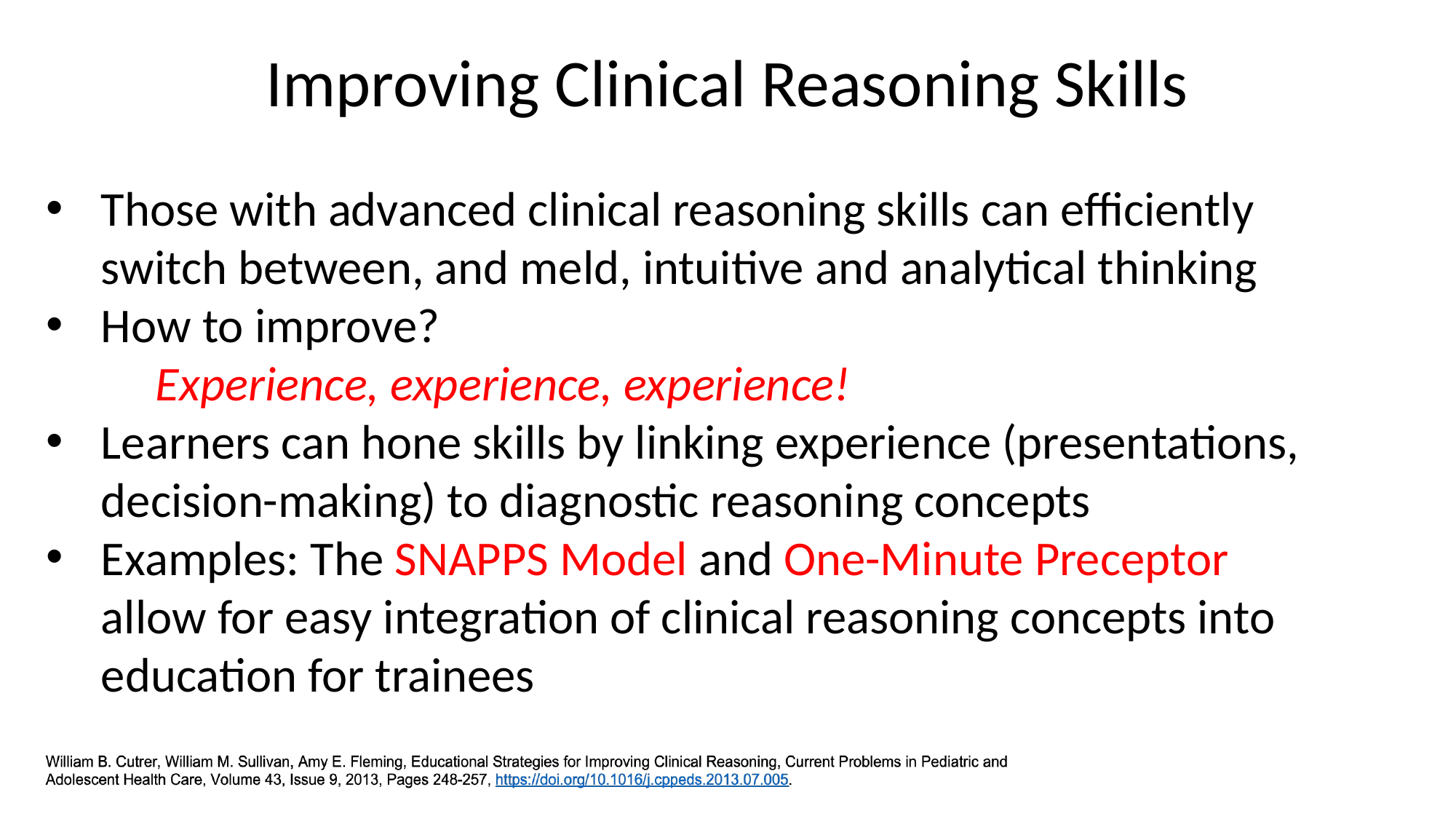

# Improving Clinical Reasoning Skills
Those with advanced clinical reasoning skills can efficiently switch between, and meld, intuitive and analytical thinking
How to improve?
	Experience, experience, experience!
Learners can hone skills by linking experience (presentations, decision-making) to diagnostic reasoning concepts
Examples: The SNAPPS Model and One-Minute Preceptor allow for easy integration of clinical reasoning concepts into education for trainees

## Slide 12
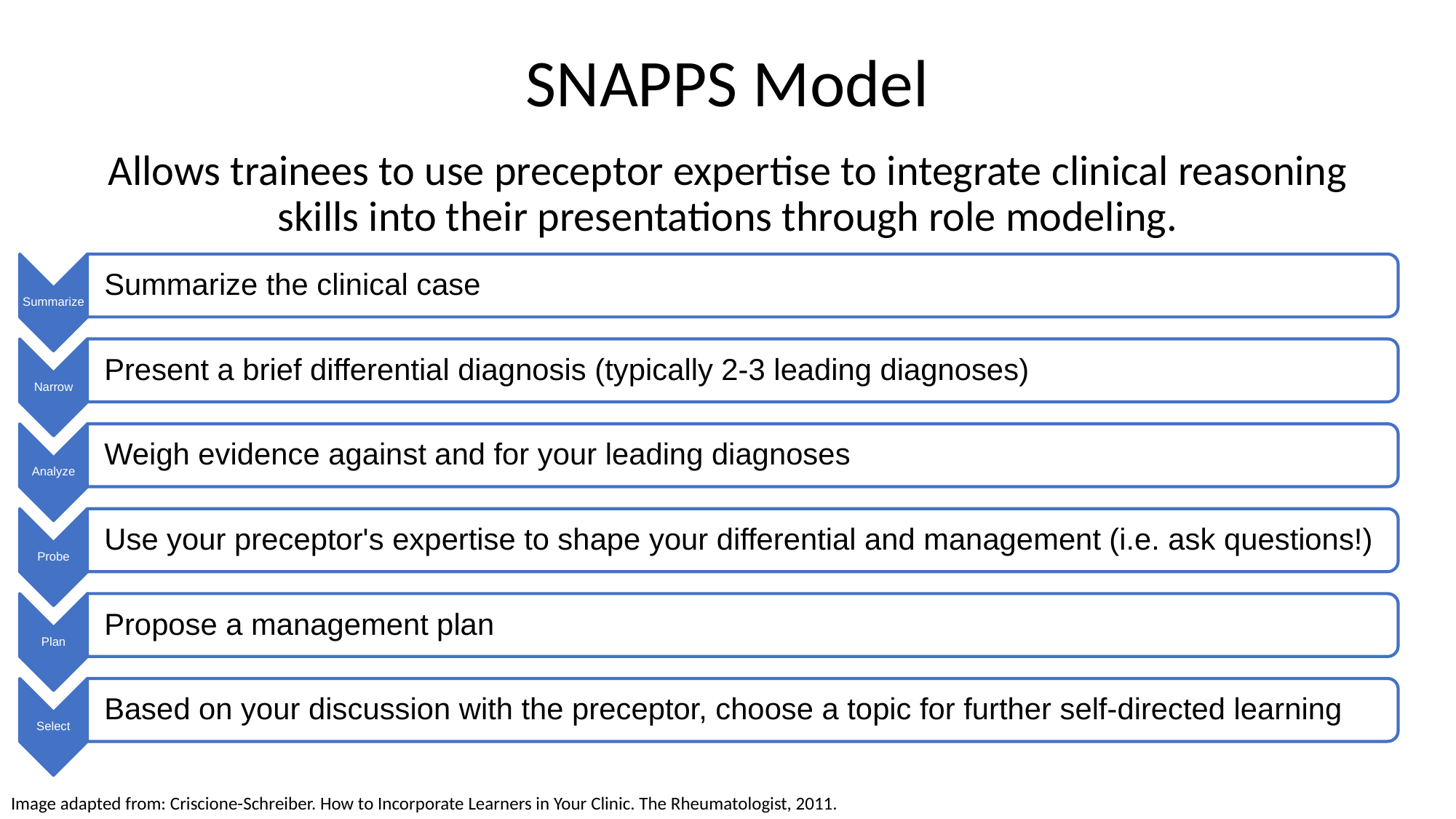

# SNAPPS Model
Allows trainees to use preceptor expertise to integrate clinical reasoning skills into their presentations through role modeling.
Image adapted from: Criscione-Schreiber. How to Incorporate Learners in Your Clinic. The Rheumatologist, 2011.

## Slide 13
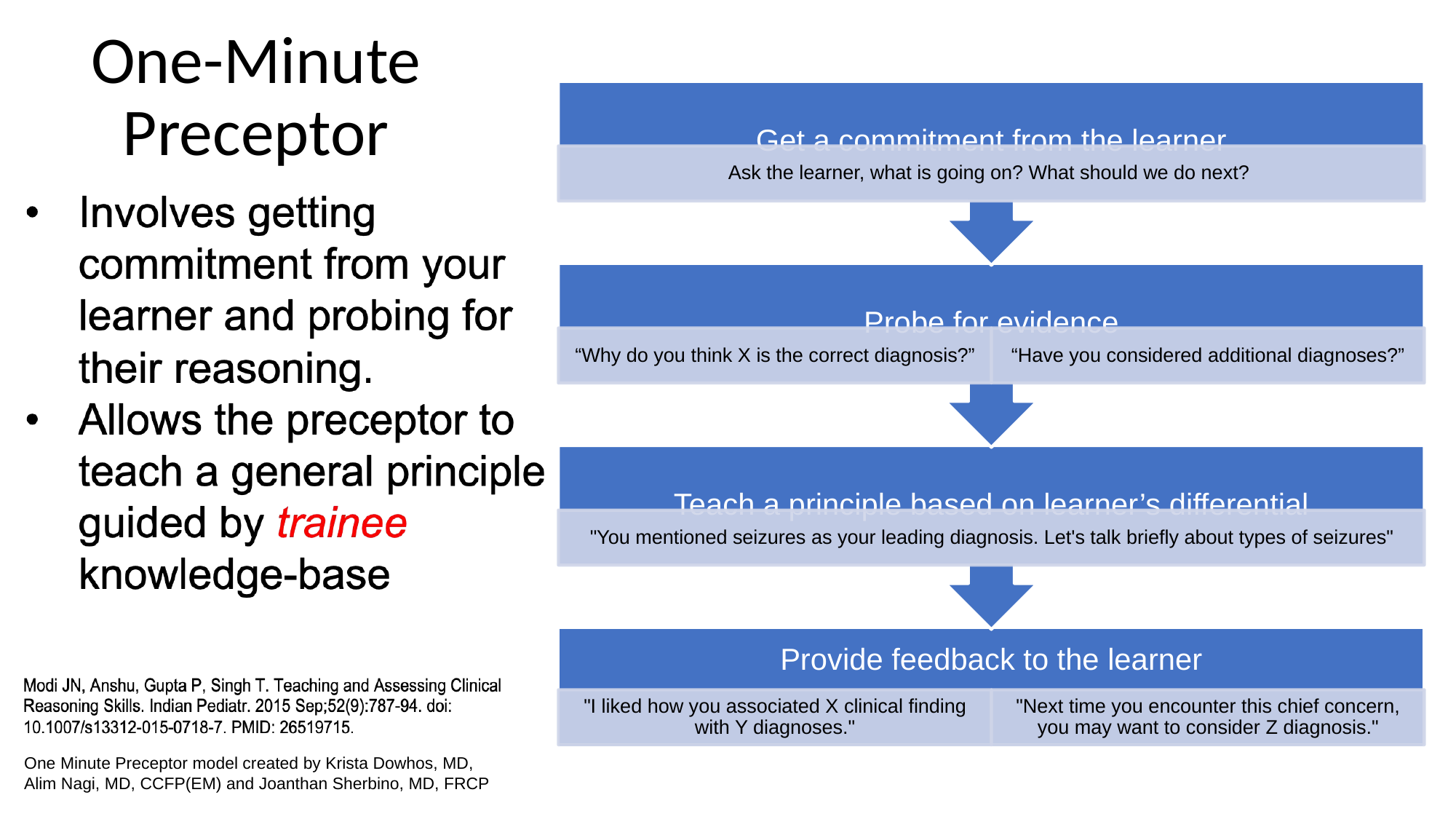

# One-Minute Preceptor
One Minute Preceptor model created by Krista Dowhos, MD, Alim Nagi, MD, CCFP(EM) and Joanthan Sherbino, MD, FRCP

## Slide 14
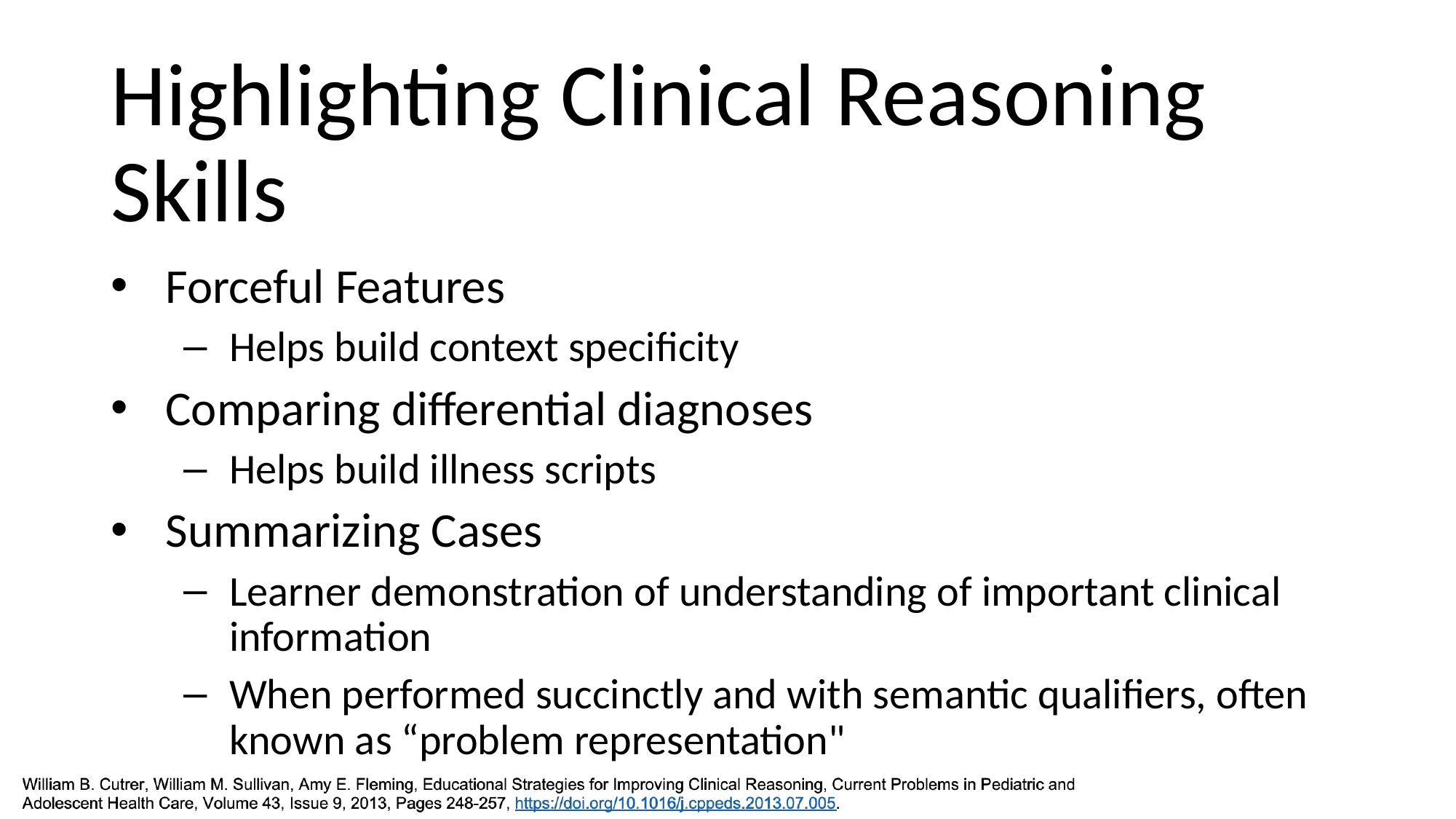

# Highlighting Clinical Reasoning Skills
Forceful Features
Helps build context specificity
Comparing differential diagnoses
Helps build illness scripts
Summarizing Cases
Learner demonstration of understanding of important clinical information
When performed succinctly and with semantic qualifiers, often known as “problem representation"

## Slide 15
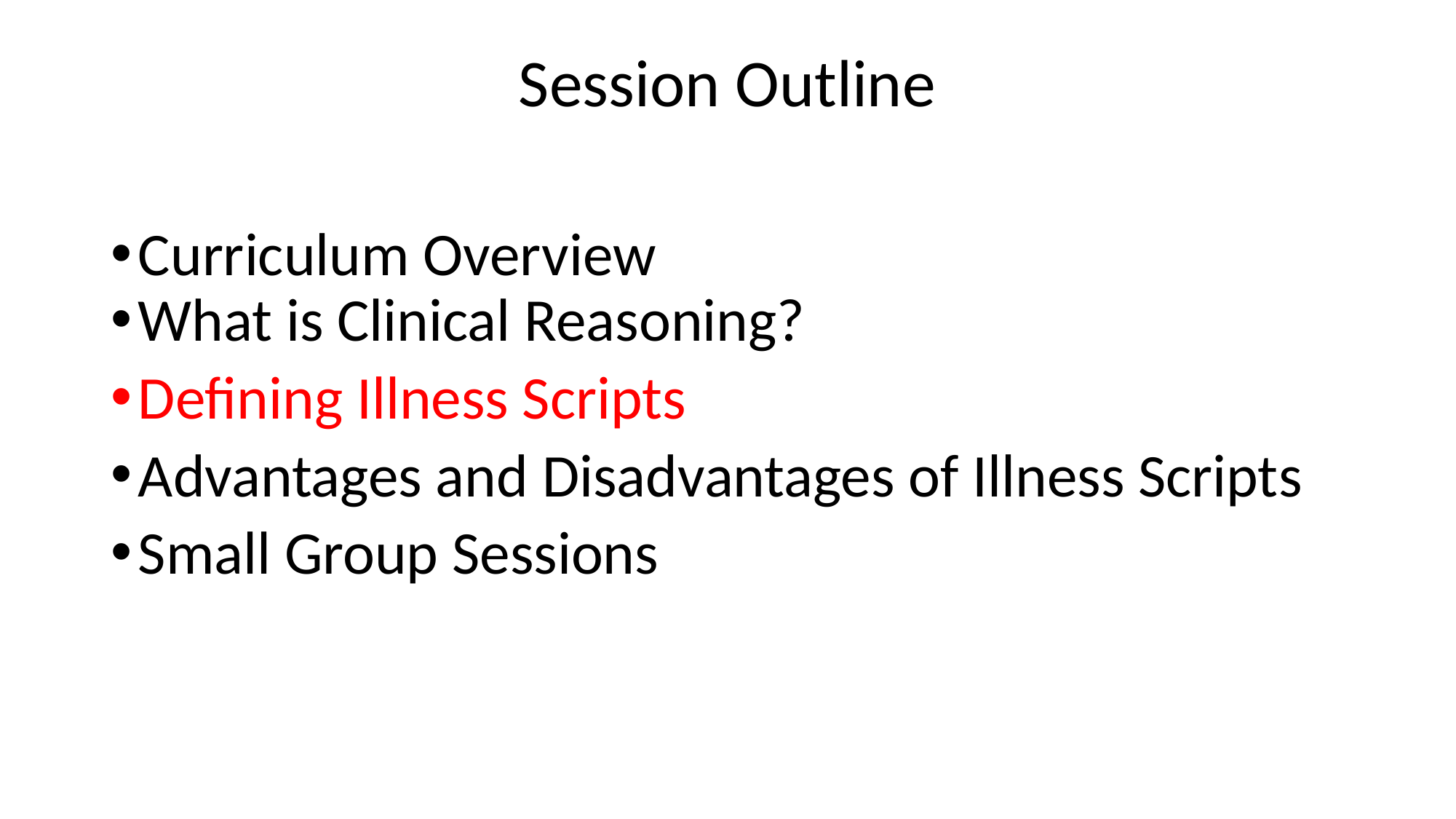

# Session Outline
Curriculum Overview
What is Clinical Reasoning?
Defining Illness Scripts
Advantages and Disadvantages of Illness Scripts
Small Group Sessions

## Slide 16
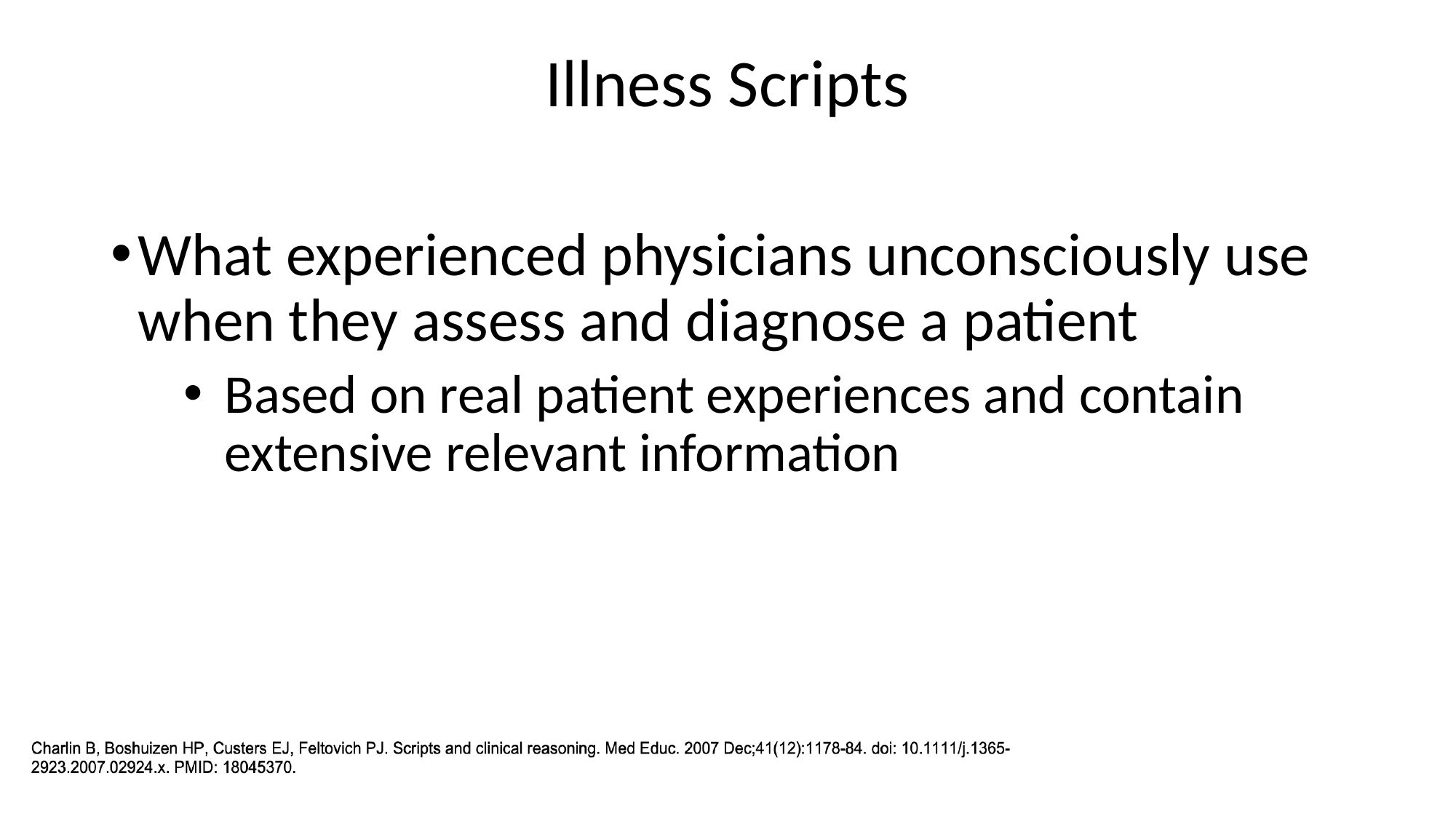

# Illness Scripts
What experienced physicians unconsciously use when they assess and diagnose a patient
Based on real patient experiences and contain extensive relevant information

## Slide 17
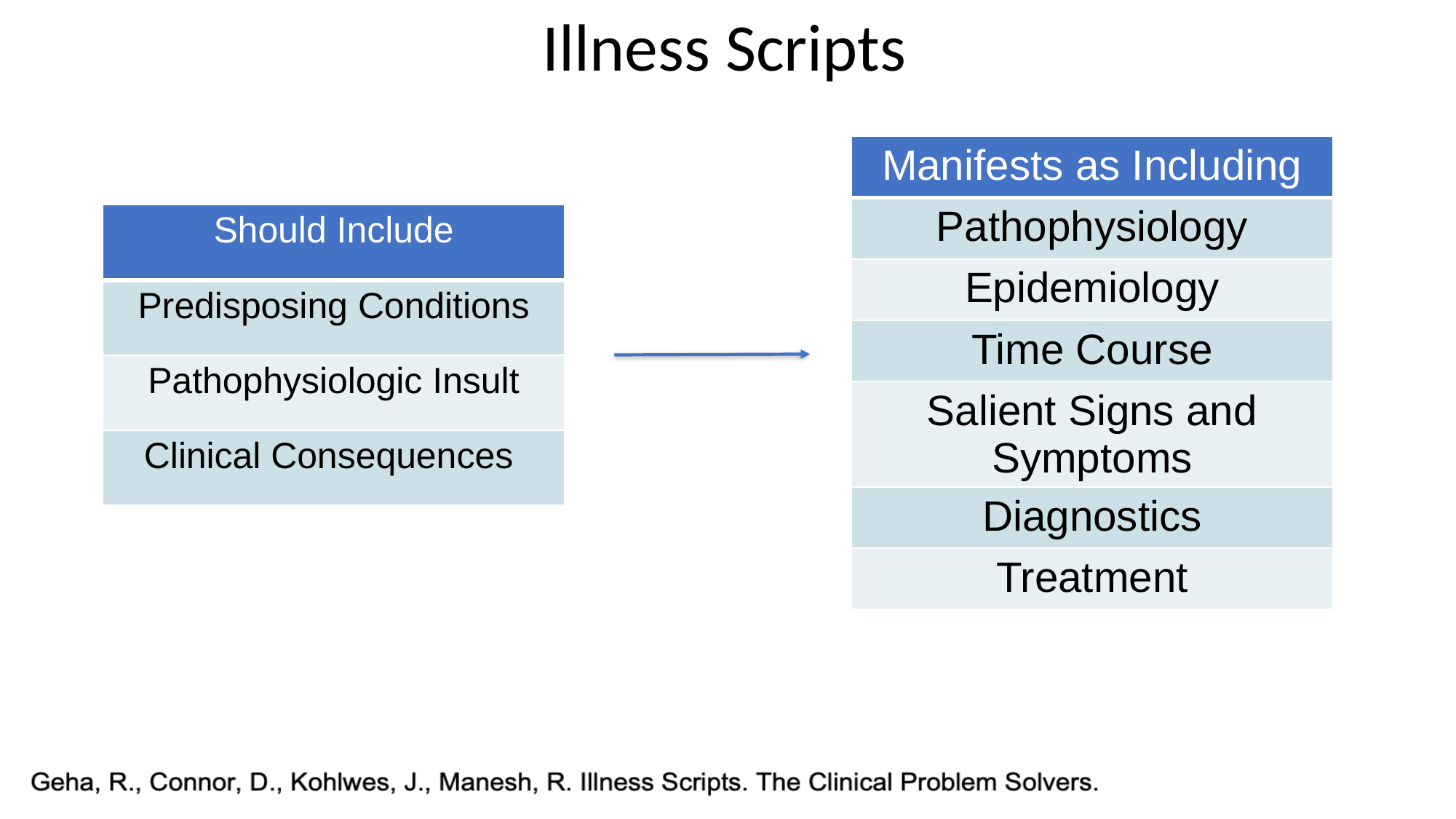

# Illness Scripts
| Manifests as Including |
| --- |
| Pathophysiology |
| Epidemiology |
| Time Course |
| Salient Signs and Symptoms |
| Diagnostics |
| Treatment |
| Should Include |
| --- |
| Predisposing Conditions |
| Pathophysiologic Insult |
| Clinical Consequences |

## Slide 18
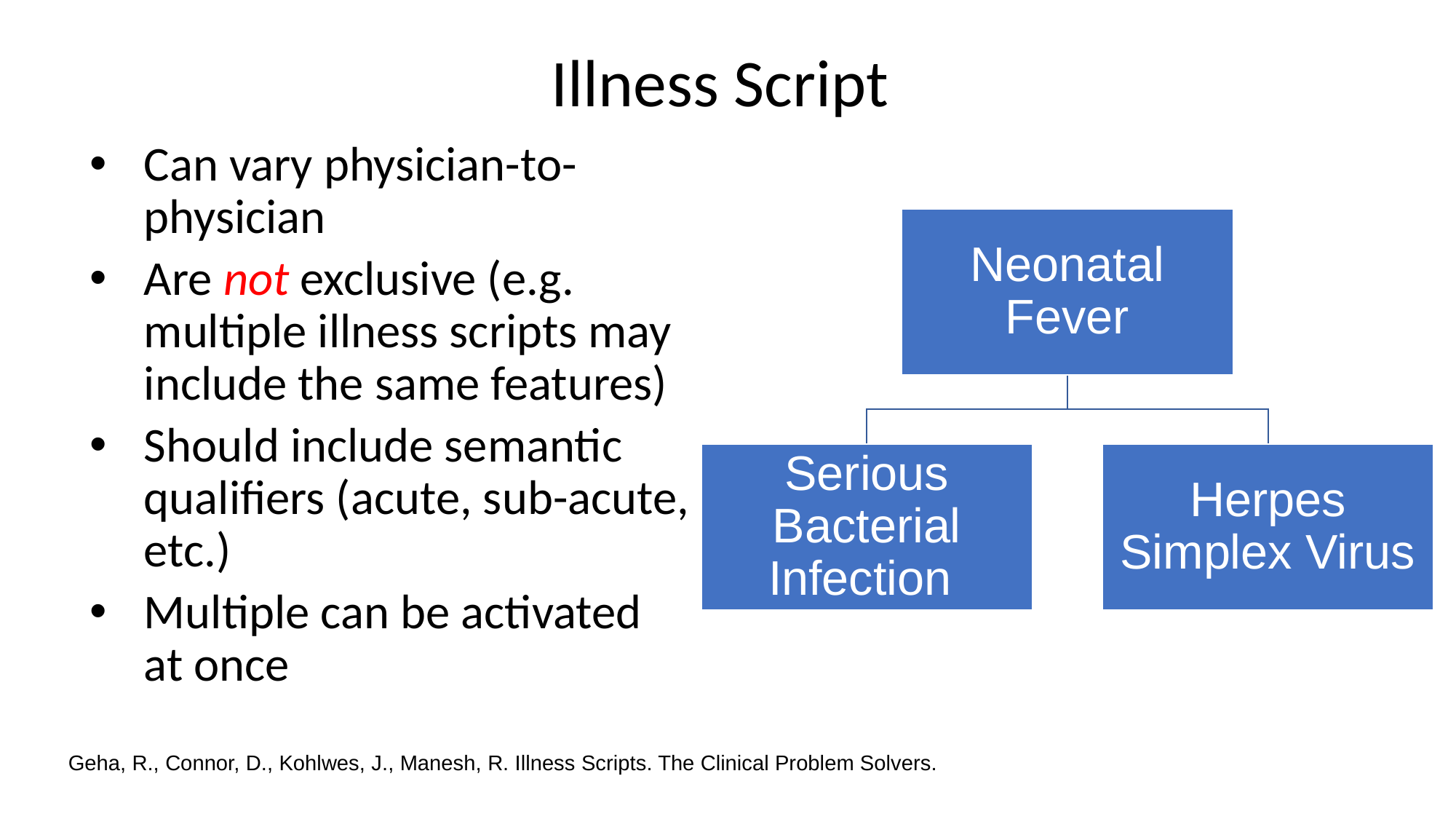

# Illness Script
Can vary physician-to-physician
Are not exclusive (e.g. multiple illness scripts may include the same features)
Should include semantic qualifiers (acute, sub-acute, etc.)
Multiple can be activated at once
Neonatal Fever
Serious Bacterial Infection
Herpes Simplex Virus
Geha, R., Connor, D., Kohlwes, J., Manesh, R. Illness Scripts. The Clinical Problem Solvers.

## Slide 19
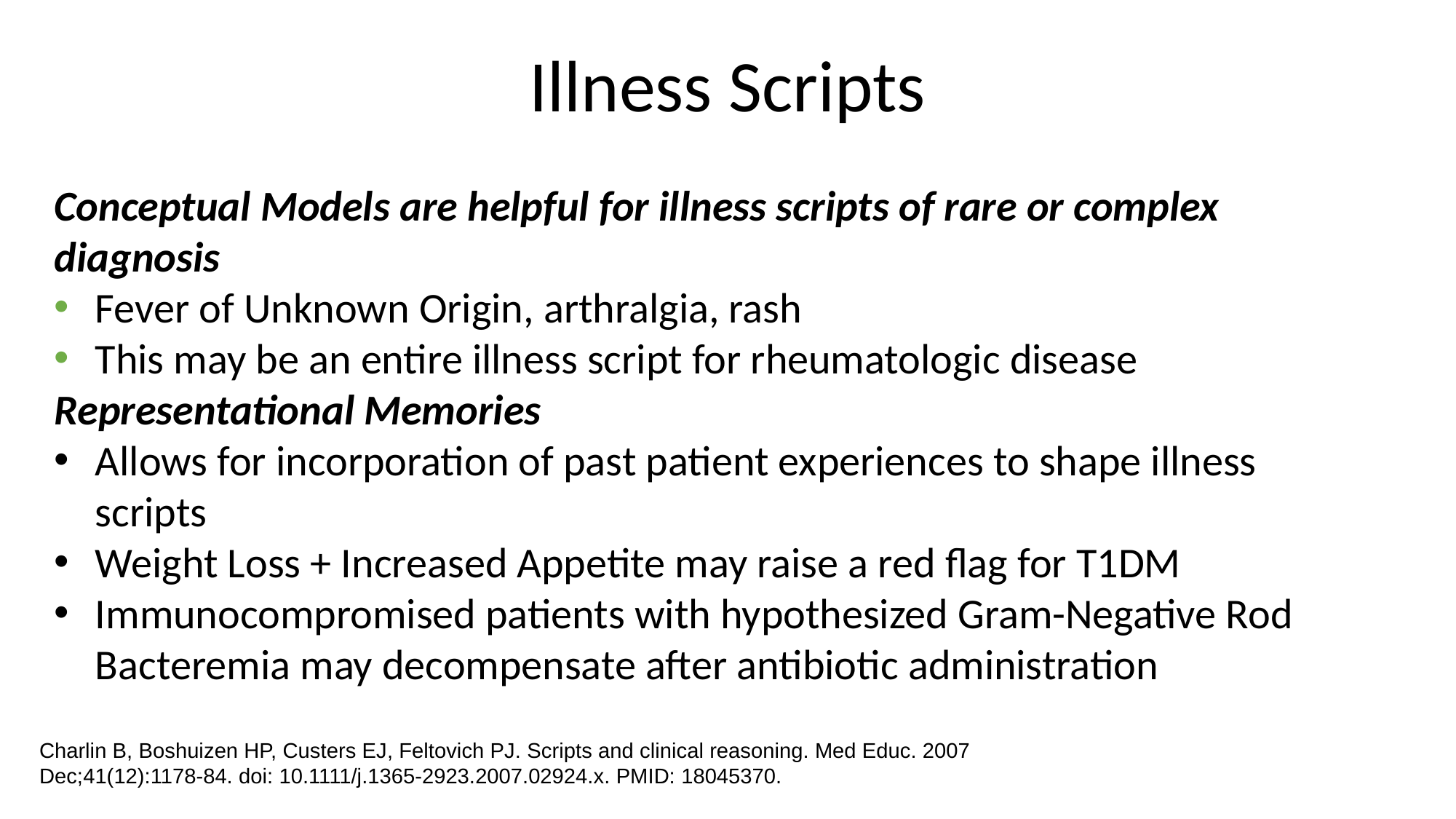

# Illness Scripts
Conceptual Models are helpful for illness scripts of rare or complex diagnosis
Fever of Unknown Origin, arthralgia, rash
This may be an entire illness script for rheumatologic disease
Representational Memories
Allows for incorporation of past patient experiences to shape illness scripts
Weight Loss + Increased Appetite may raise a red flag for T1DM
Immunocompromised patients with hypothesized Gram-Negative Rod Bacteremia may decompensate after antibiotic administration
Charlin B, Boshuizen HP, Custers EJ, Feltovich PJ. Scripts and clinical reasoning. Med Educ. 2007 Dec;41(12):1178-84. doi: 10.1111/j.1365-2923.2007.02924.x. PMID: 18045370.

## Slide 20
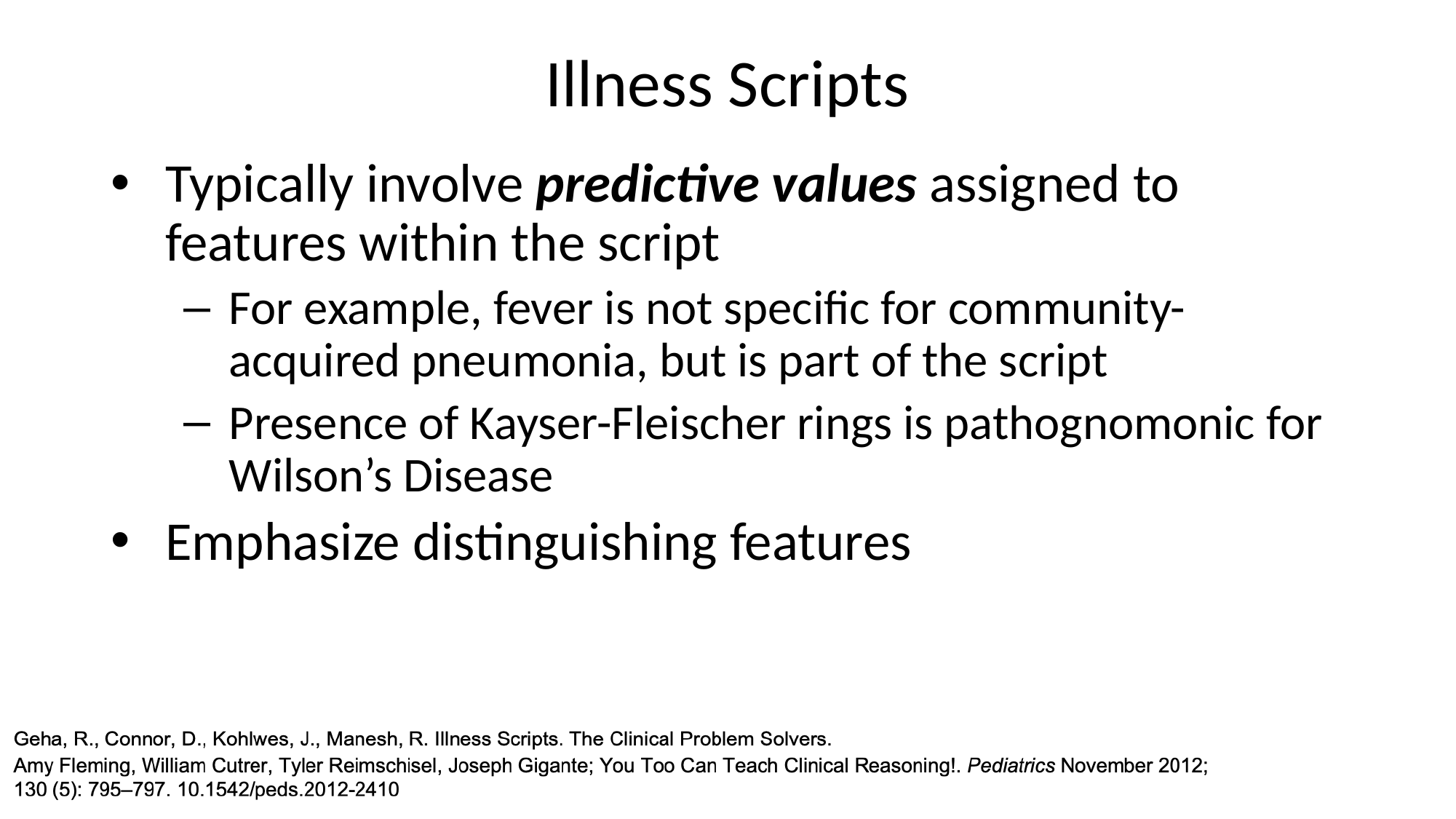

# Illness Scripts
Typically involve predictive values assigned to features within the script
For example, fever is not specific for community-acquired pneumonia, but is part of the script
Presence of Kayser-Fleischer rings is pathognomonic for Wilson’s Disease
Emphasize distinguishing features

## Slide 21
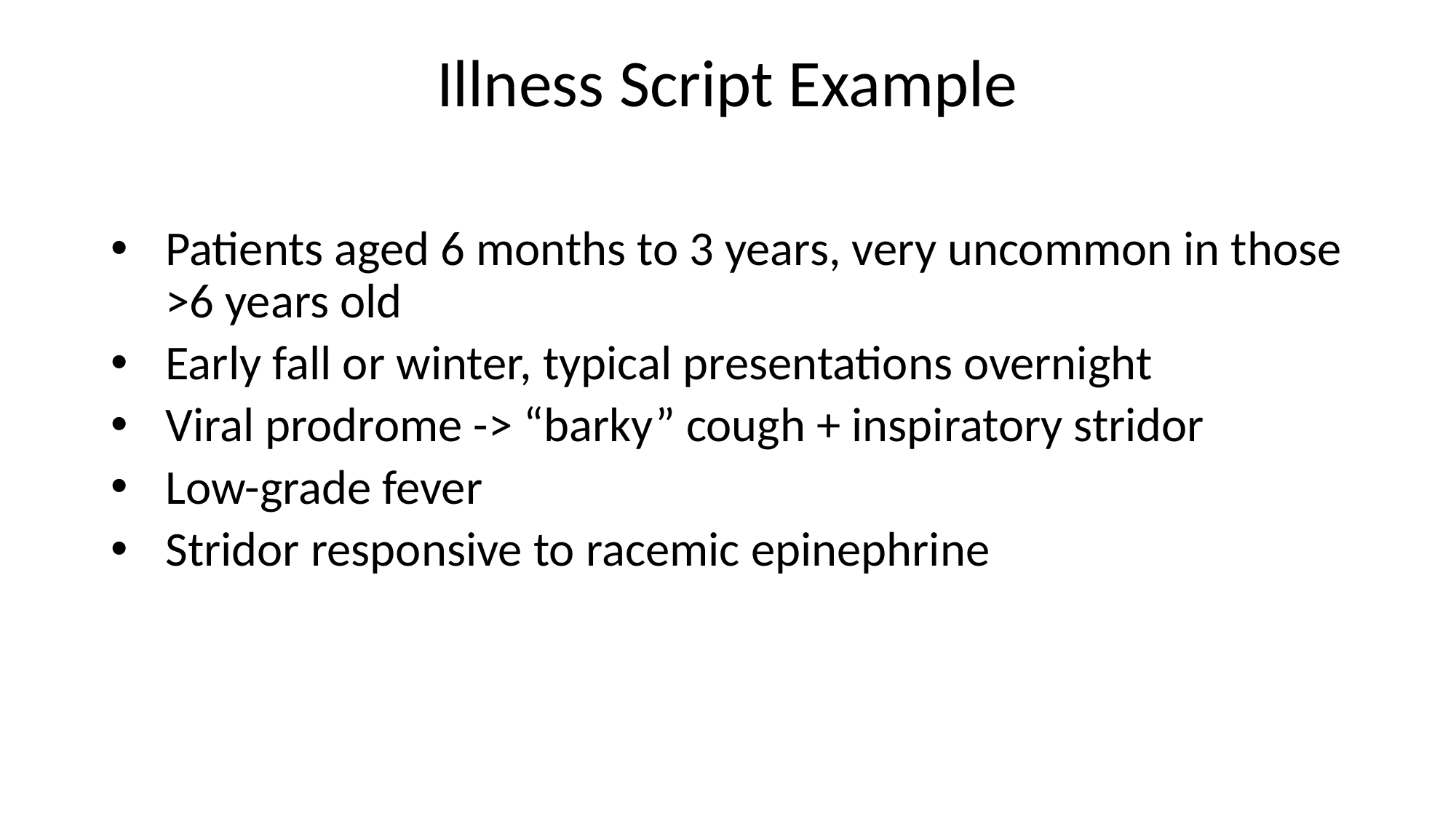

# Illness Script Example
Patients aged 6 months to 3 years, very uncommon in those >6 years old
Early fall or winter, typical presentations overnight
Viral prodrome -> “barky” cough + inspiratory stridor
Low-grade fever
Stridor responsive to racemic epinephrine

## Slide 22
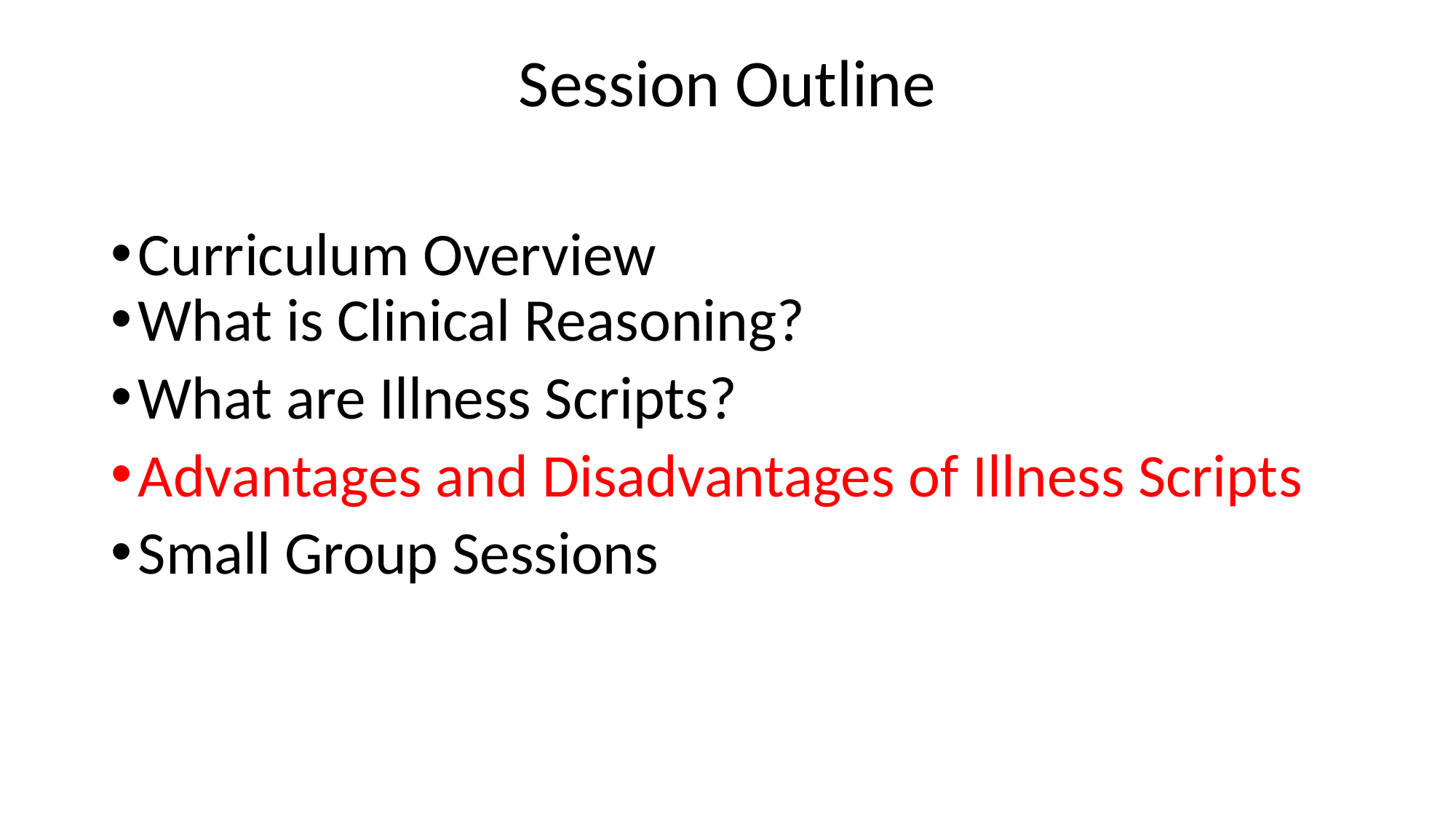

# Session Outline
Curriculum Overview
What is Clinical Reasoning?
What are Illness Scripts?
Advantages and Disadvantages of Illness Scripts
Small Group Sessions

## Slide 23
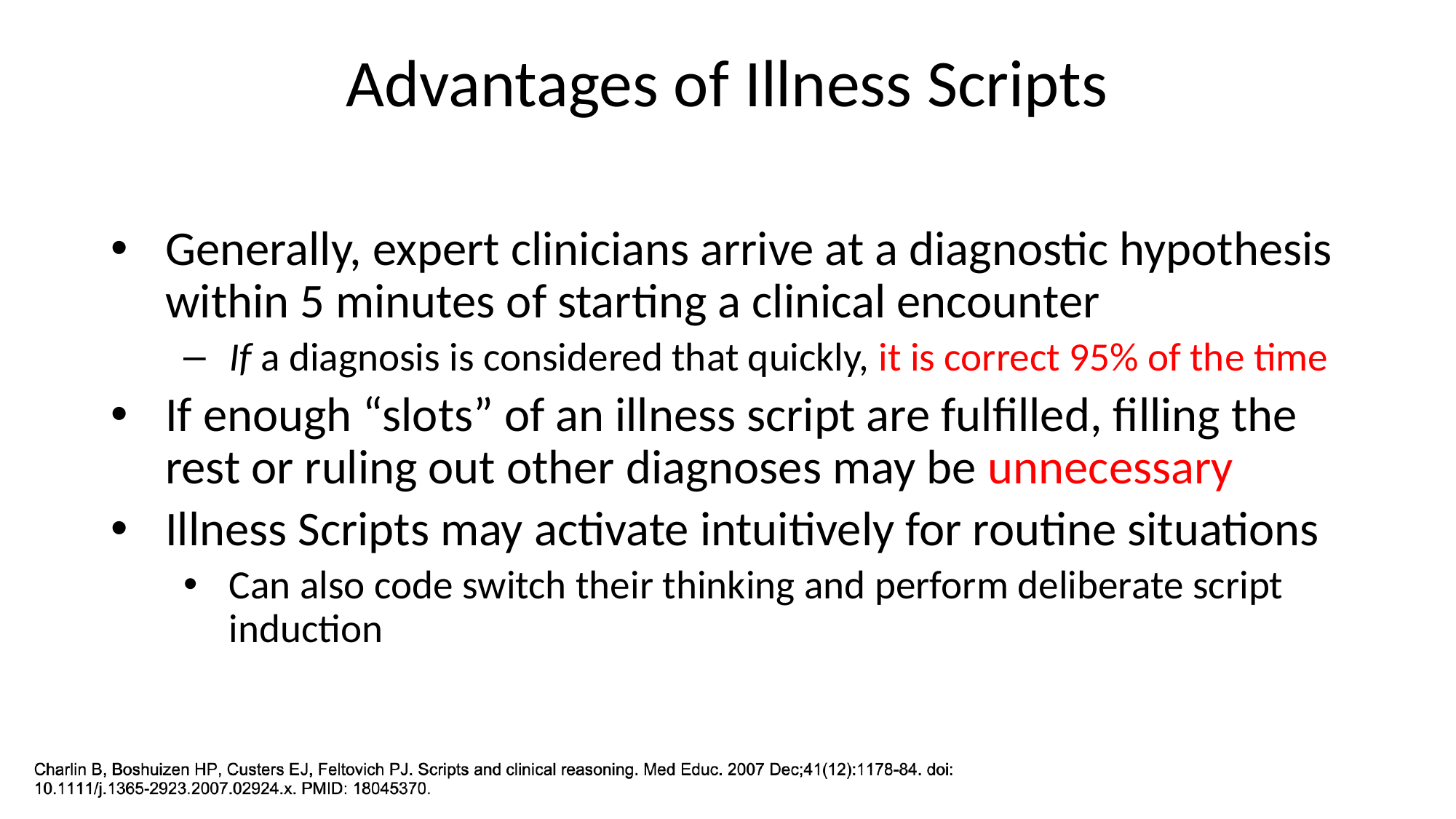

# Advantages of Illness Scripts
Generally, expert clinicians arrive at a diagnostic hypothesis within 5 minutes of starting a clinical encounter
If a diagnosis is considered that quickly, it is correct 95% of the time
If enough “slots” of an illness script are fulfilled, filling the rest or ruling out other diagnoses may be unnecessary
Illness Scripts may activate intuitively for routine situations
Can also code switch their thinking and perform deliberate script induction

## Slide 24
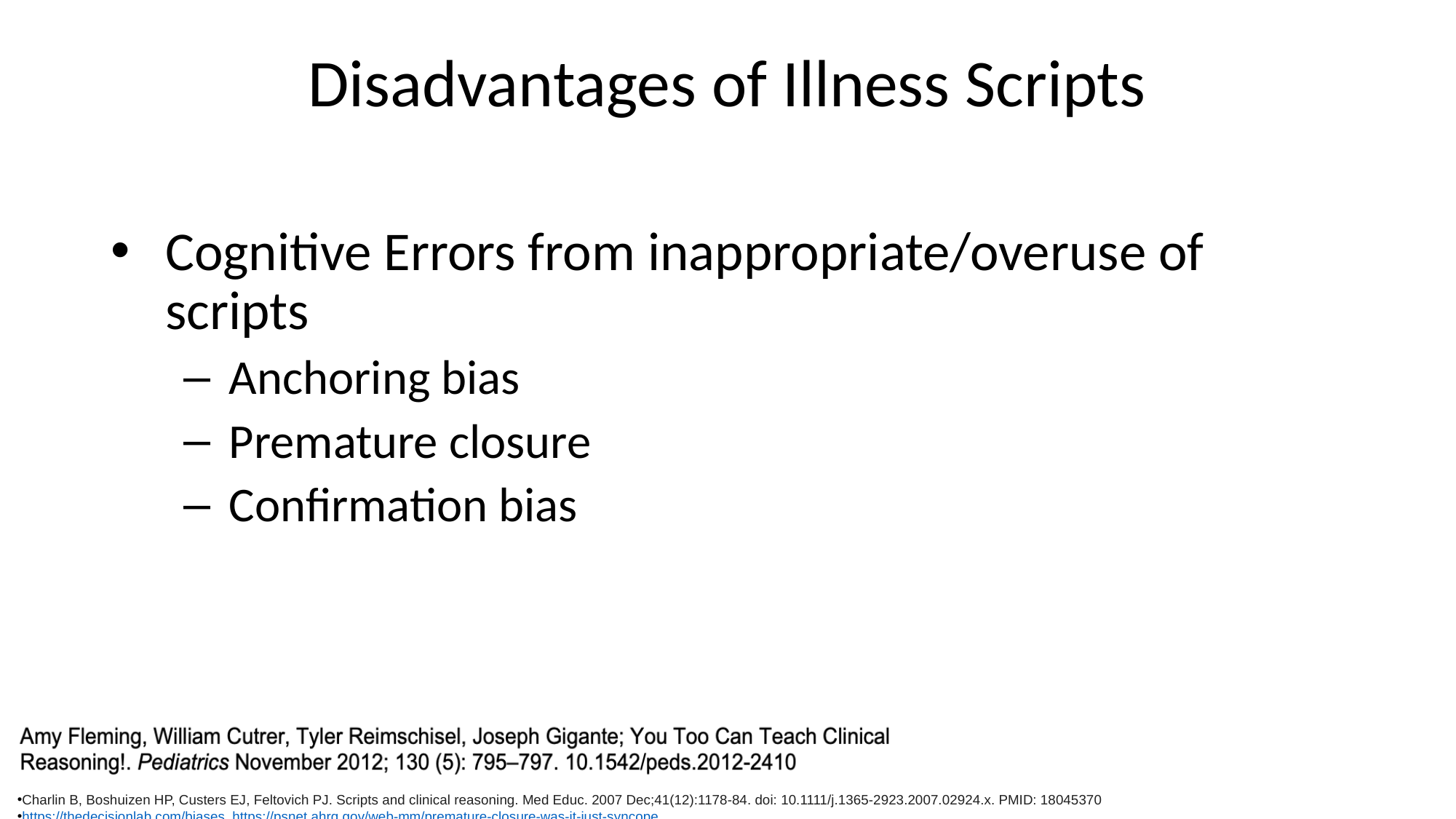

# Disadvantages of Illness Scripts
Cognitive Errors from inappropriate/overuse of scripts
Anchoring bias
Premature closure
Confirmation bias
Charlin B, Boshuizen HP, Custers EJ, Feltovich PJ. Scripts and clinical reasoning. Med Educ. 2007 Dec;41(12):1178-84. doi: 10.1111/j.1365-2923.2007.02924.x. PMID: 18045370​
https://thedecisionlab.com/biases, https://psnet.ahrq.gov/web-mm/premature-closure-was-it-just-syncope

## Slide 25
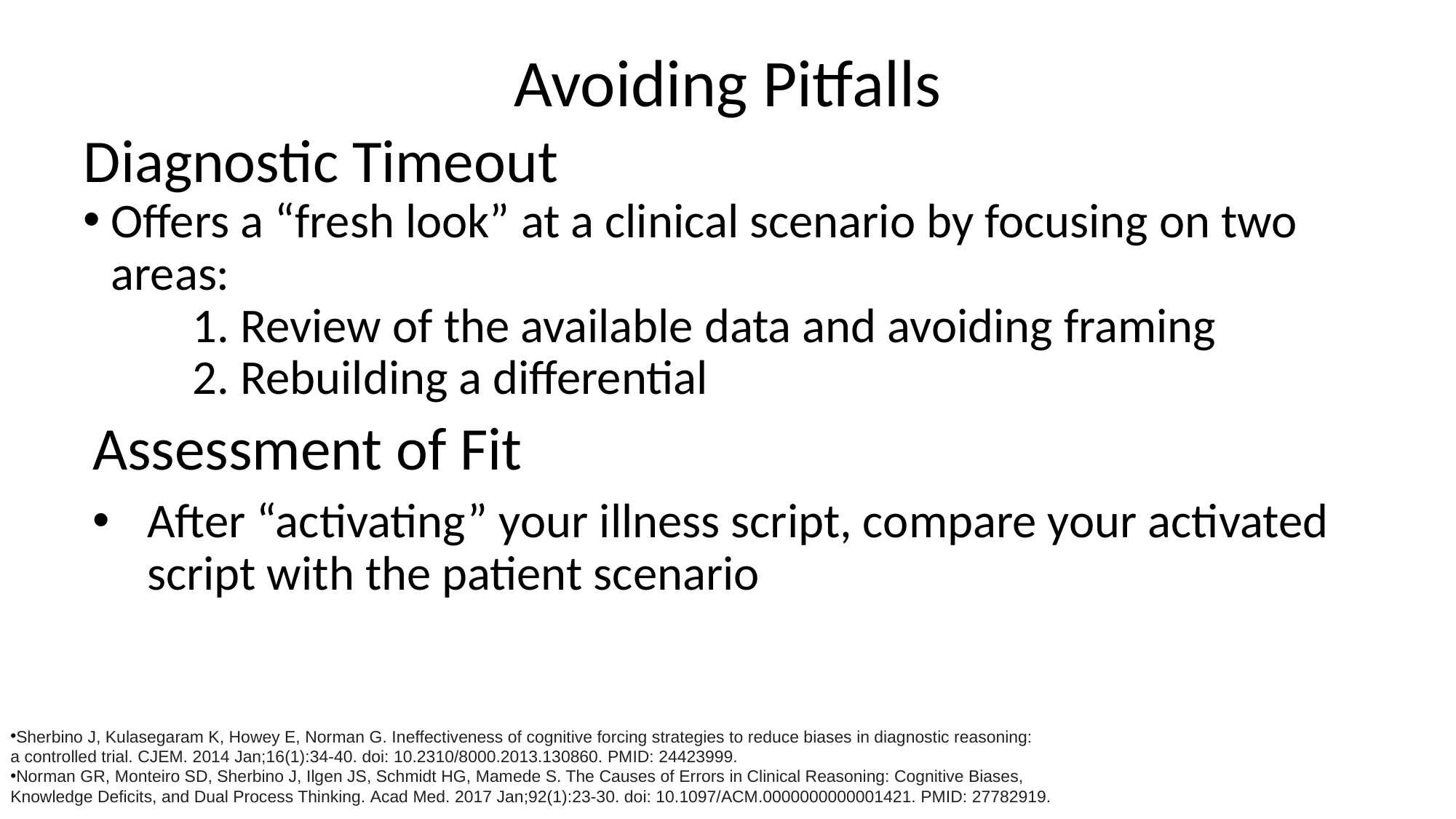

# Avoiding Pitfalls
Diagnostic Timeout
Offers a “fresh look” at a clinical scenario by focusing on two areas:
	1. Review of the available data and avoiding framing
	2. Rebuilding a differential
Assessment of Fit
After “activating” your illness script, compare your activated script with the patient scenario
Sherbino J, Kulasegaram K, Howey E, Norman G. Ineffectiveness of cognitive forcing strategies to reduce biases in diagnostic reasoning: a controlled trial. CJEM. 2014 Jan;16(1):34-40. doi: 10.2310/8000.2013.130860. PMID: 24423999.​​
Norman GR, Monteiro SD, Sherbino J, Ilgen JS, Schmidt HG, Mamede S. The Causes of Errors in Clinical Reasoning: Cognitive Biases, Knowledge Deficits, and Dual Process Thinking. Acad Med. 2017 Jan;92(1):23-30. doi: 10.1097/ACM.0000000000001421. PMID: 27782919.​

## Slide 26
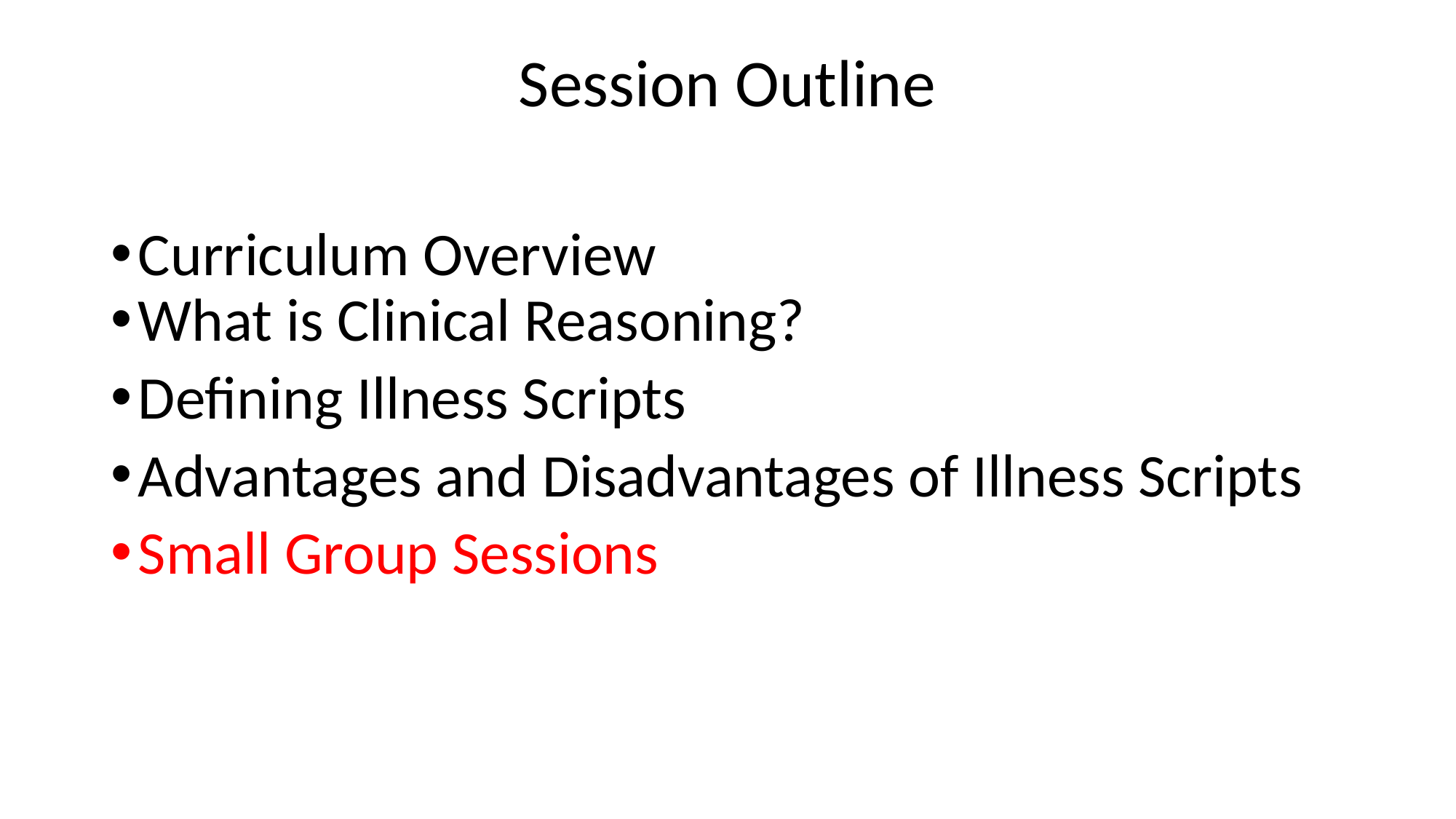

# Session Outline
Curriculum Overview
What is Clinical Reasoning?
Defining Illness Scripts
Advantages and Disadvantages of Illness Scripts
Small Group Sessions

## Slide 27
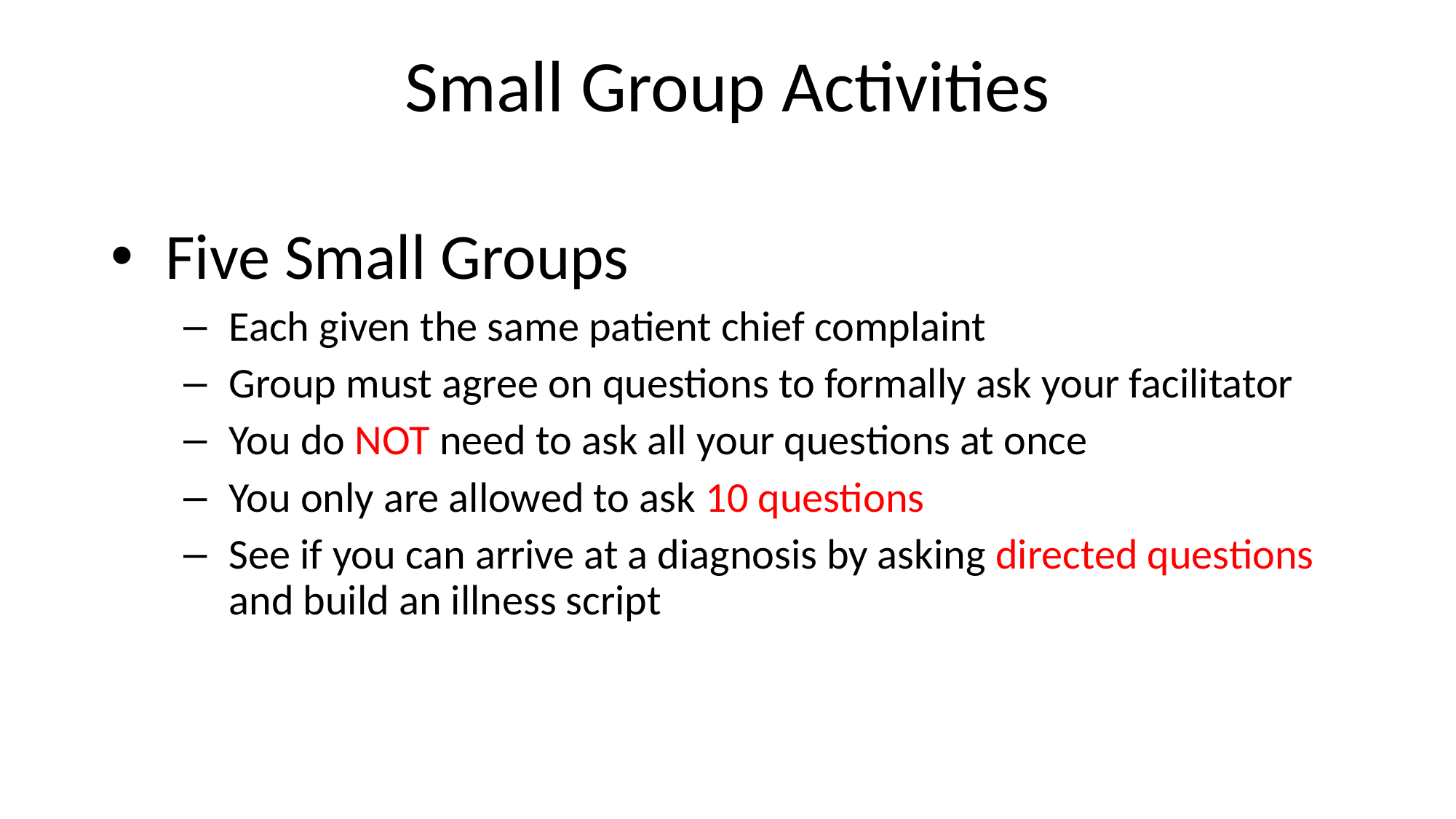

# Small Group Activities
Five Small Groups
Each given the same patient chief complaint
Group must agree on questions to formally ask your facilitator
You do NOT need to ask all your questions at once
You only are allowed to ask 10 questions
See if you can arrive at a diagnosis by asking directed questions and build an illness script

## Slide 28
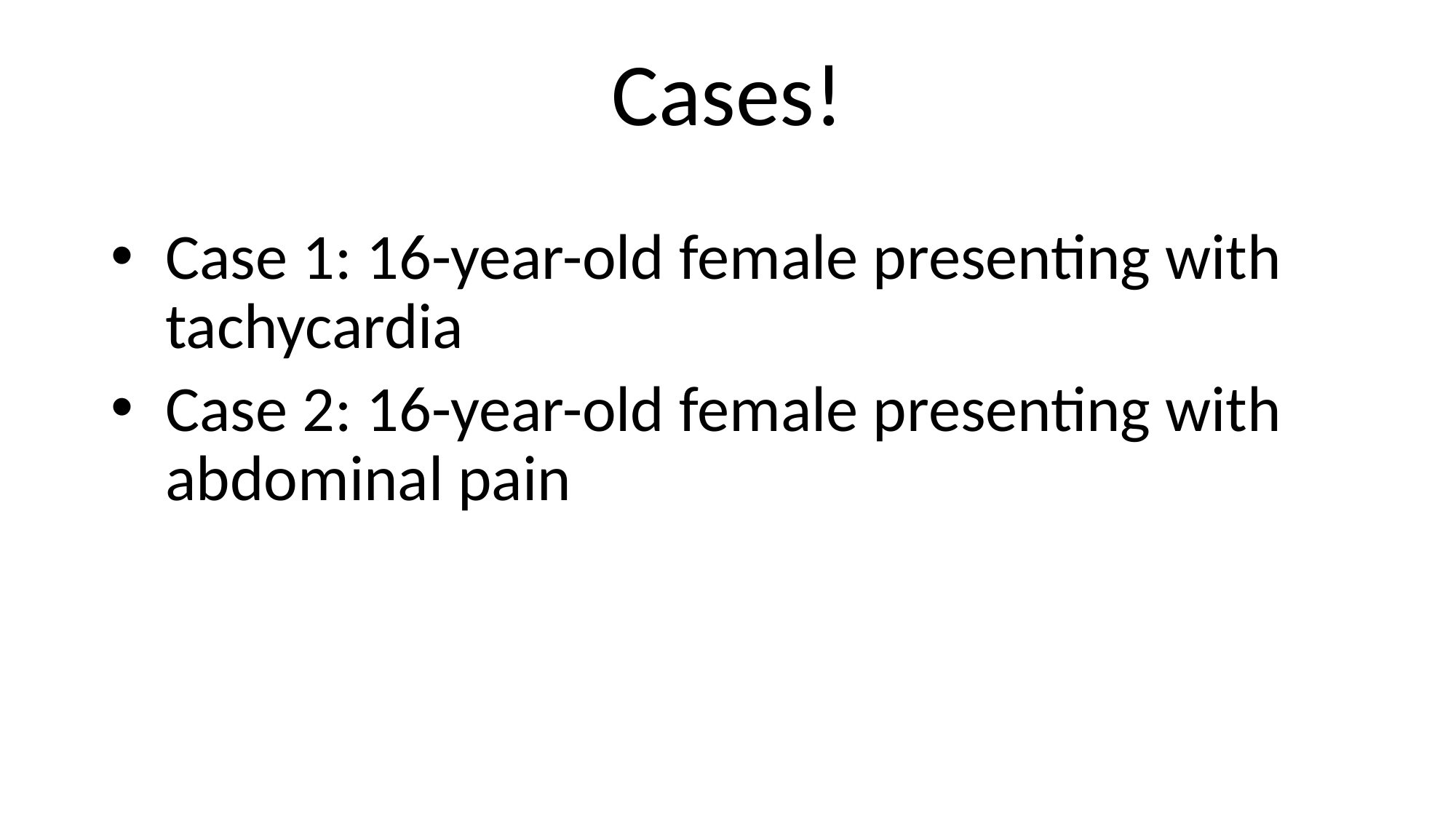

# Cases!
Case 1: 16-year-old female presenting with tachycardia
Case 2: 16-year-old female presenting with abdominal pain

## Slide 29
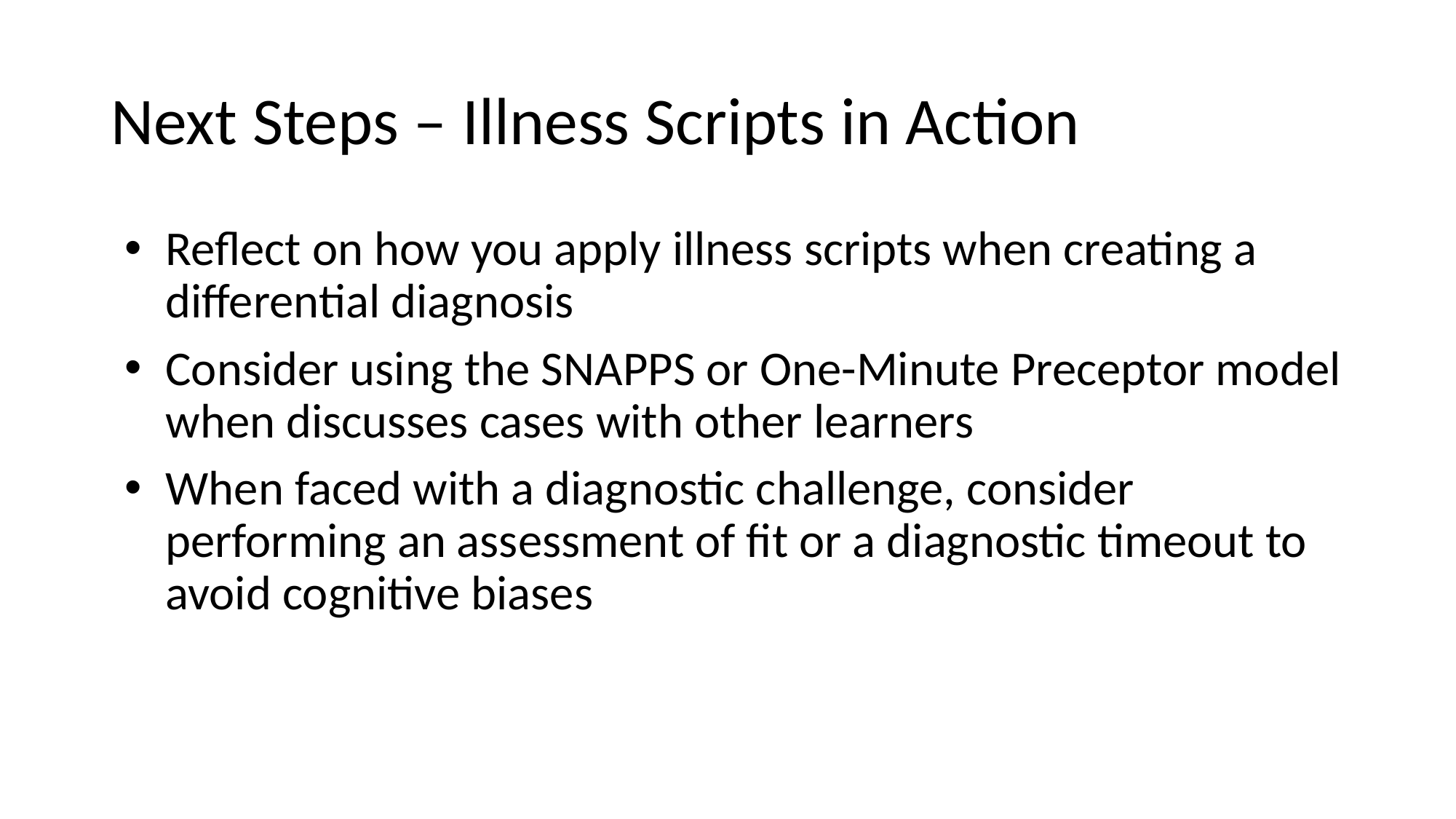

# Next Steps – Illness Scripts in Action
Reflect on how you apply illness scripts when creating a differential diagnosis
Consider using the SNAPPS or One-Minute Preceptor model when discusses cases with other learners
When faced with a diagnostic challenge, consider performing an assessment of fit or a diagnostic timeout to avoid cognitive biases

## Slide 30
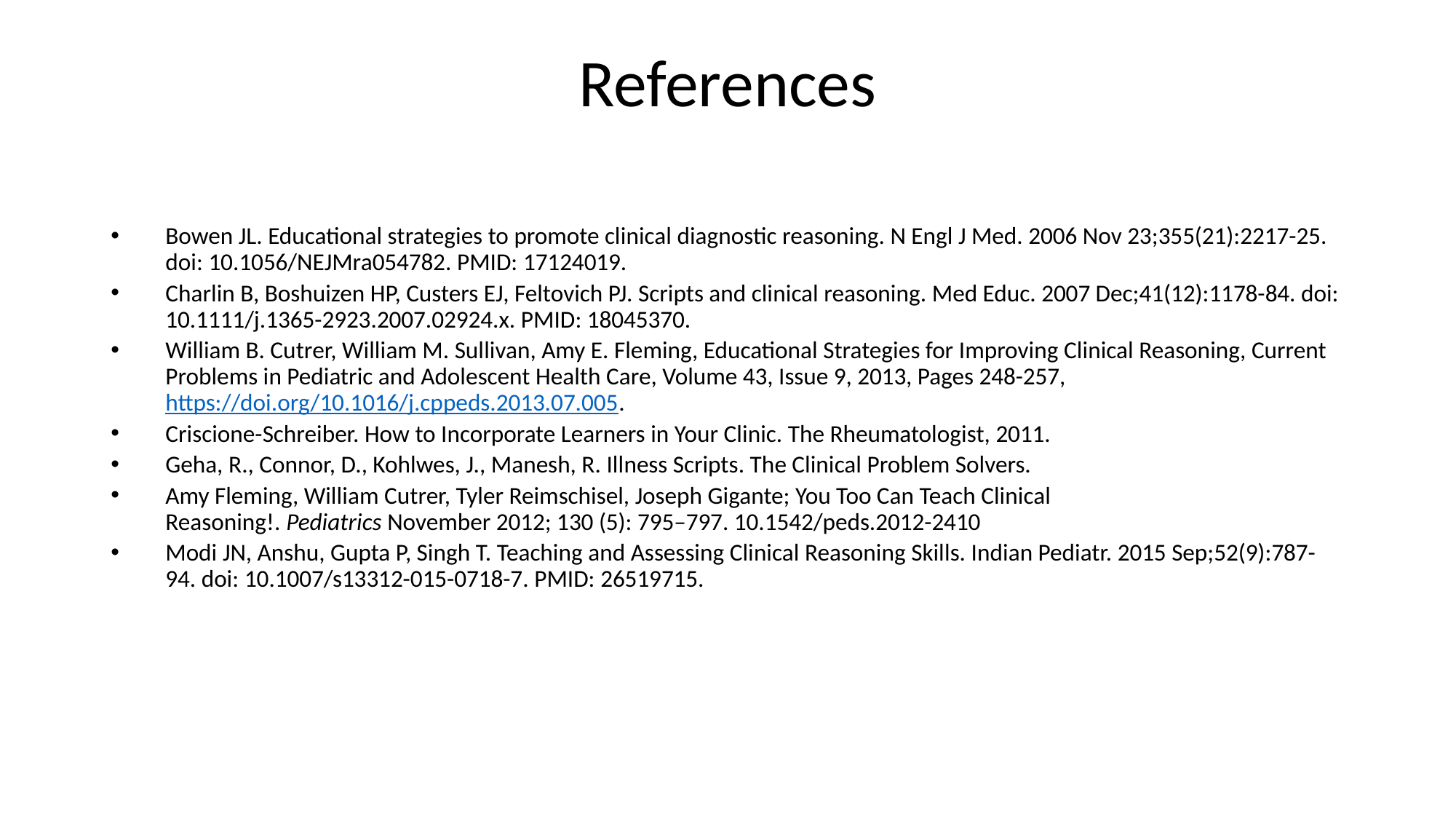

# References
Bowen JL. Educational strategies to promote clinical diagnostic reasoning. N Engl J Med. 2006 Nov 23;355(21):2217-25. doi: 10.1056/NEJMra054782. PMID: 17124019.
Charlin B, Boshuizen HP, Custers EJ, Feltovich PJ. Scripts and clinical reasoning. Med Educ. 2007 Dec;41(12):1178-84. doi: 10.1111/j.1365-2923.2007.02924.x. PMID: 18045370.
William B. Cutrer, William M. Sullivan, Amy E. Fleming, Educational Strategies for Improving Clinical Reasoning, Current Problems in Pediatric and Adolescent Health Care, Volume 43, Issue 9, 2013, Pages 248-257, https://doi.org/10.1016/j.cppeds.2013.07.005.
Criscione-Schreiber. How to Incorporate Learners in Your Clinic. The Rheumatologist, 2011.
Geha, R., Connor, D., Kohlwes, J., Manesh, R. Illness Scripts. The Clinical Problem Solvers.
Amy Fleming, William Cutrer, Tyler Reimschisel, Joseph Gigante; You Too Can Teach Clinical Reasoning!. Pediatrics November 2012; 130 (5): 795–797. 10.1542/peds.2012-2410
Modi JN, Anshu, Gupta P, Singh T. Teaching and Assessing Clinical Reasoning Skills. Indian Pediatr. 2015 Sep;52(9):787-94. doi: 10.1007/s13312-015-0718-7. PMID: 26519715.
