# Supplementary material for: A Pilot Longitudinal Clinical Reasoning Curriculum for Pediatric Residents
Source: MedEdPORTAL. 2024 Sep 25;20:11447. doi: 10.15766/mep_2374-8265.11447 (PMC11422513; doi:10.15766/mep_2374-8265.11447)
Supplement: Supplementary file 1 — Preimplementation Survey.docxCurriculum Goals, Objectives, and Timeline.docxSession 1 - Illness Scripts.pptxSession 1 - Small-Group Facilitator Guide.docxSession 2 - Illness Scripts 2.pptxSession 2 - Small-Group Facilitator Guide.docxSession 3 - Script Concordance.pptxSession 3 - Small-Group Facilitator Guide.docxSession 3 - Small-Group Handout.docxSession 4 - Pathophysiology.pptxSession 4 - Small-Group Facilitator Guide.docxSession 4 - Small-Group Handout.docxSession 5 - Review Game.pptxPostimplementation Survey.docx [file mep_2374-8265.11447-s001.zip › G. Session 3 - Script Concordance.pptx]

## Slide 1
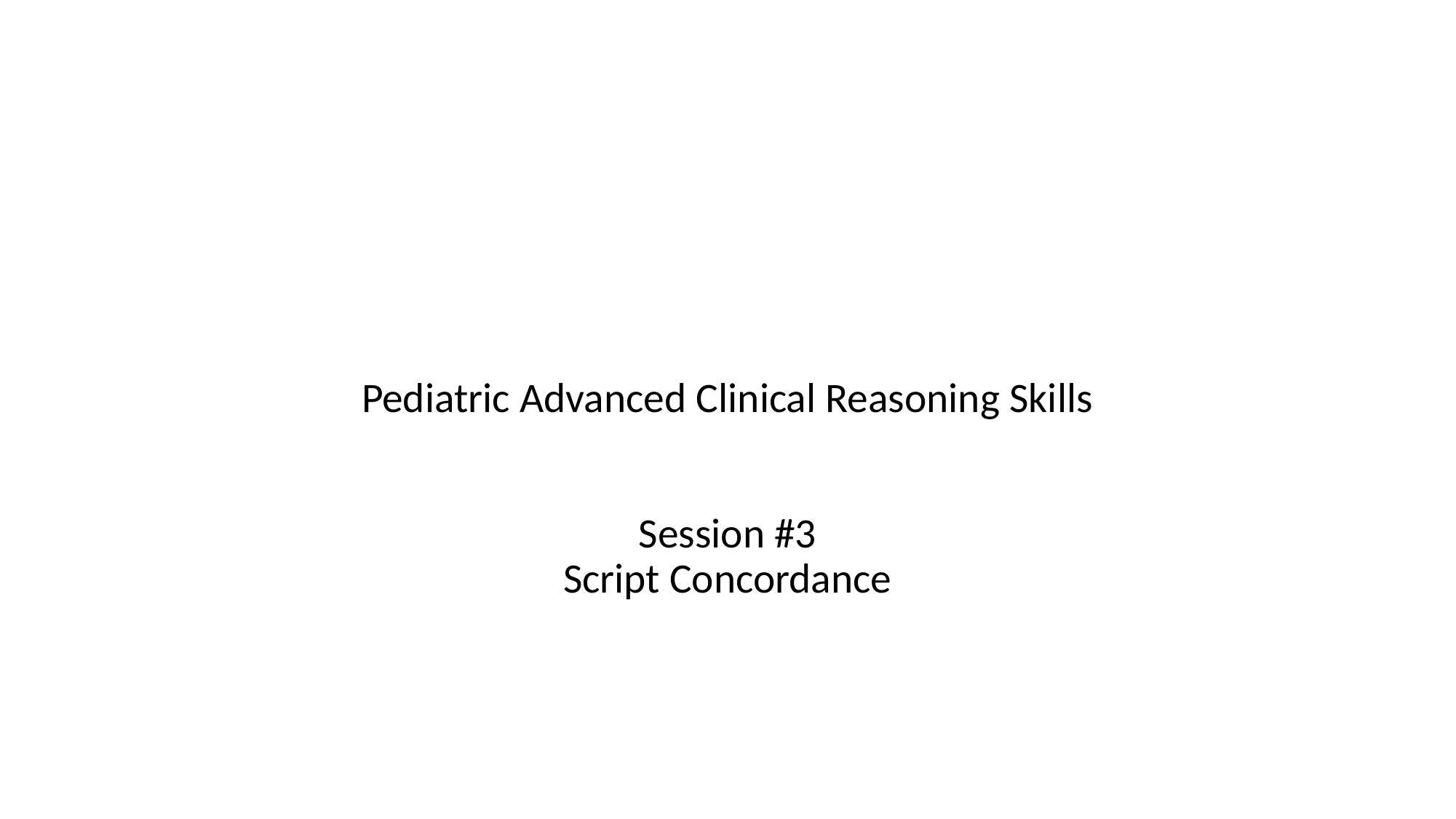

# Pediatric Advanced Clinical Reasoning Skills Session #3 Script Concordance

## Slide 2
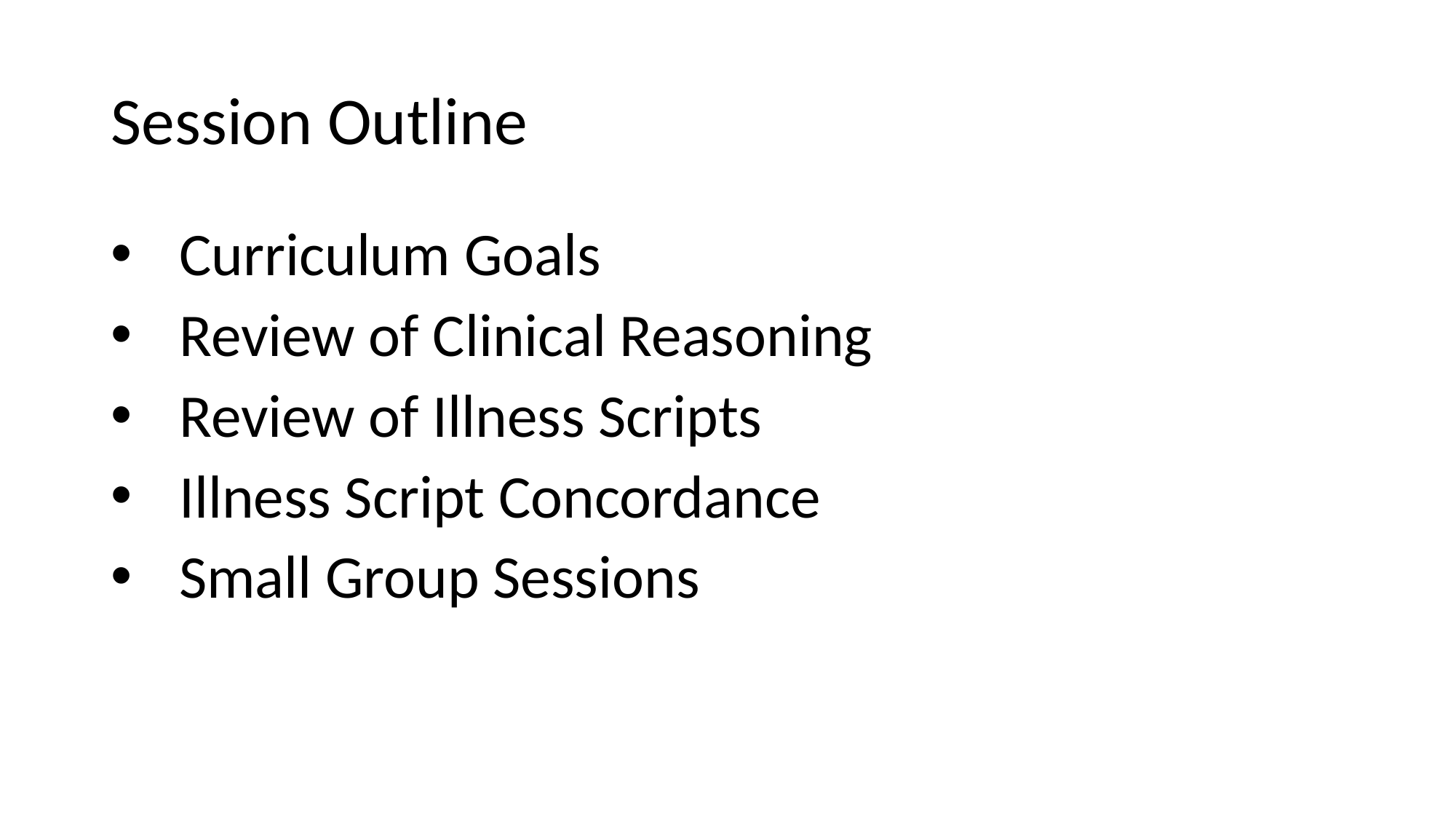

# Session Outline
Curriculum Goals
Review of Clinical Reasoning
Review of Illness Scripts
Illness Script Concordance
Small Group Sessions

## Slide 3
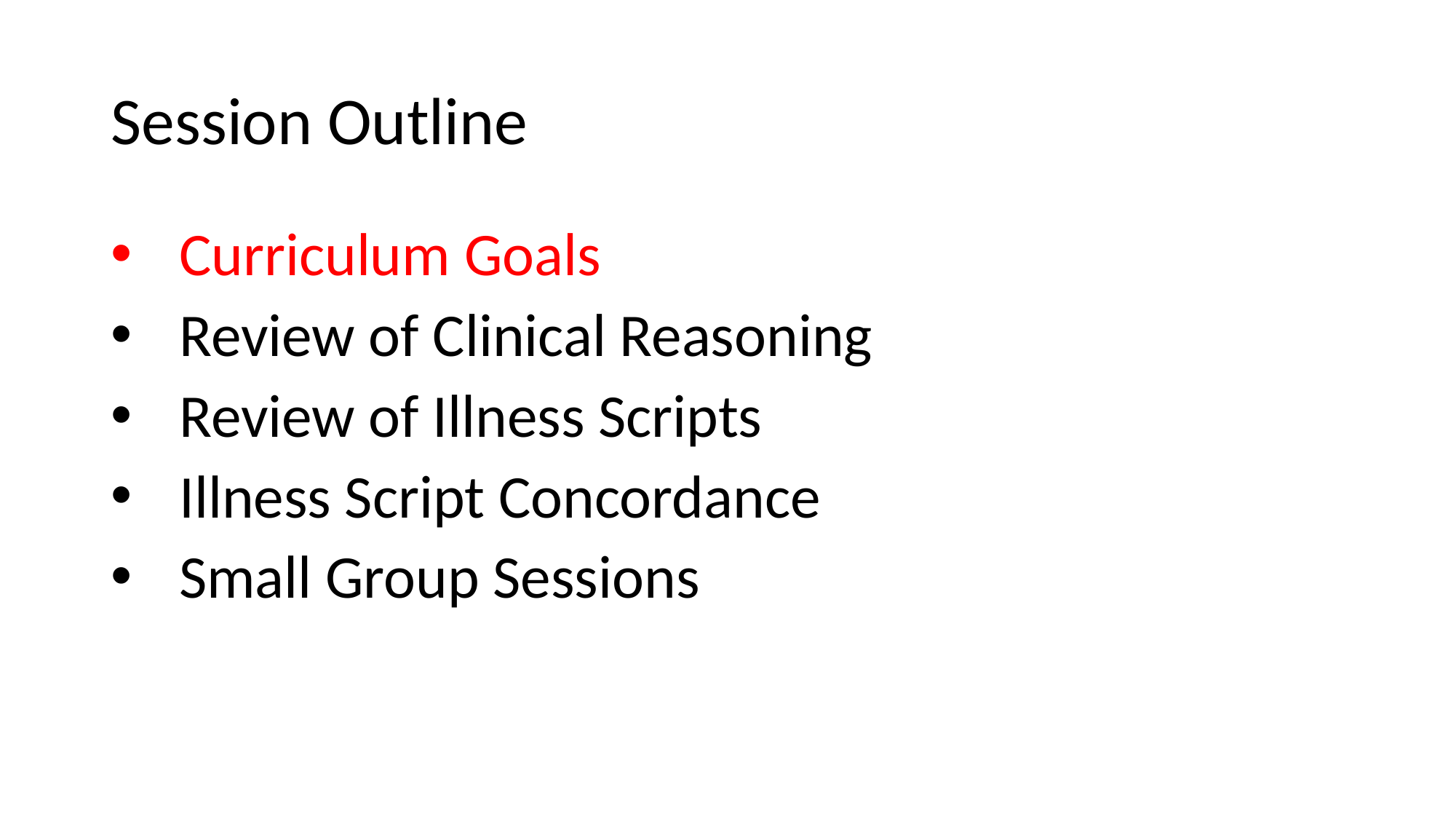

# Session Outline
Curriculum Goals
Review of Clinical Reasoning
Review of Illness Scripts
Illness Script Concordance
Small Group Sessions

## Slide 4
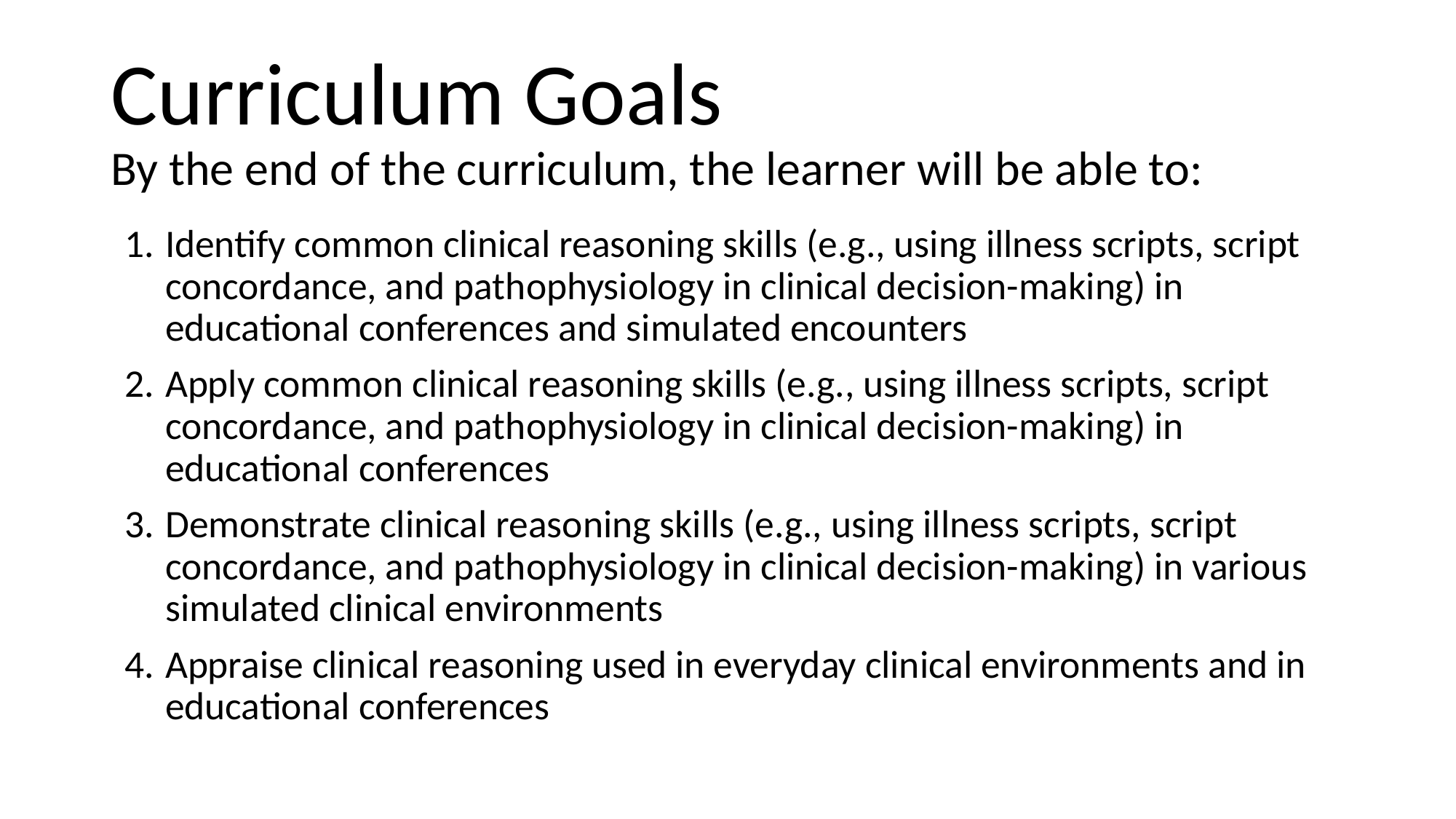

# Curriculum Goals By the end of the curriculum, the learner will be able to:
Identify common clinical reasoning skills (e.g., using illness scripts, script concordance, and pathophysiology in clinical decision-making) in educational conferences and simulated encounters
Apply common clinical reasoning skills (e.g., using illness scripts, script concordance, and pathophysiology in clinical decision-making) in educational conferences
Demonstrate clinical reasoning skills (e.g., using illness scripts, script concordance, and pathophysiology in clinical decision-making) in various simulated clinical environments
Appraise clinical reasoning used in everyday clinical environments and in educational conferences

## Slide 5
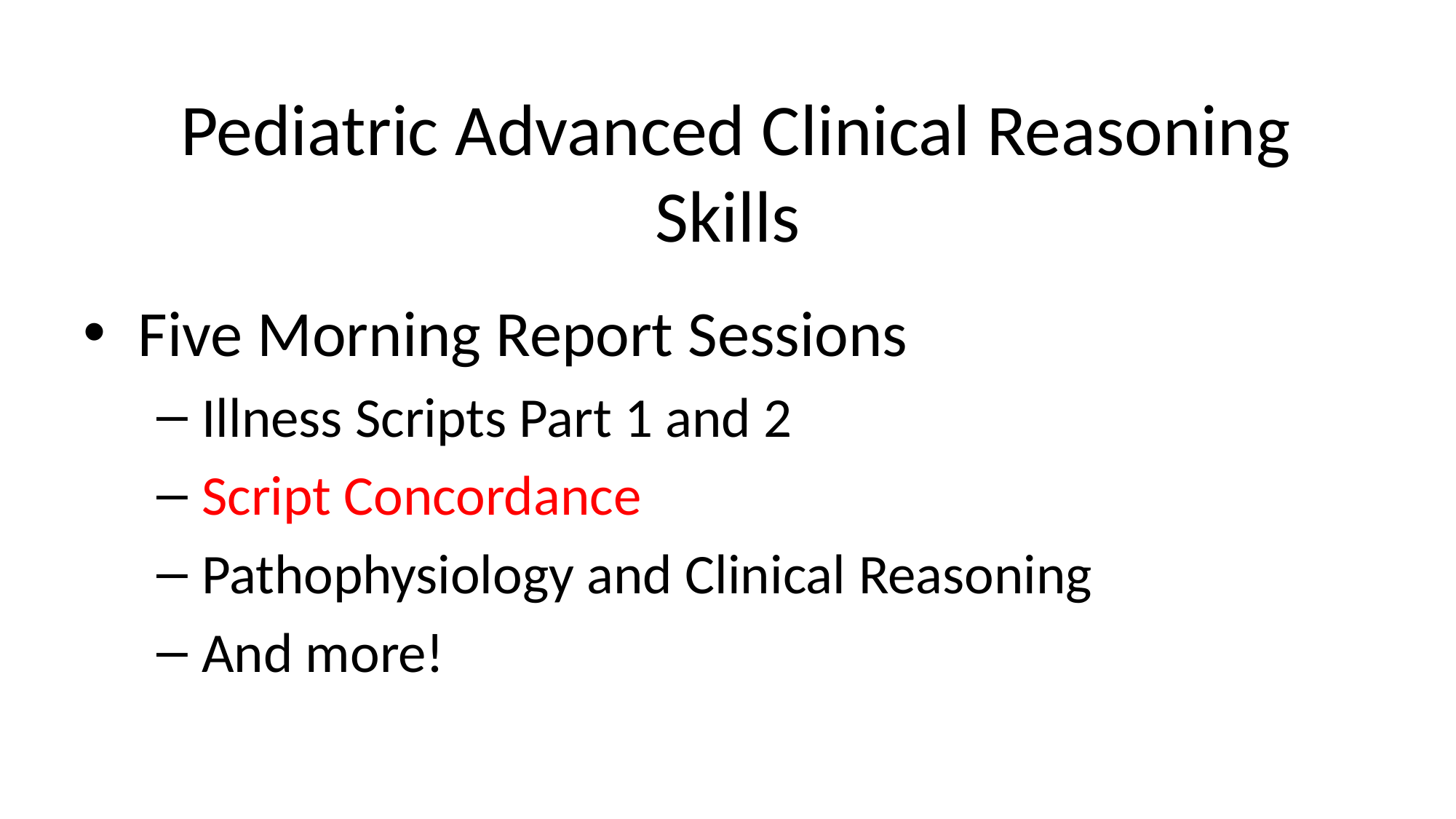

# Pediatric Advanced Clinical Reasoning Skills
Five Morning Report Sessions
Illness Scripts Part 1 and 2
Script Concordance
Pathophysiology and Clinical Reasoning
And more!

## Slide 6
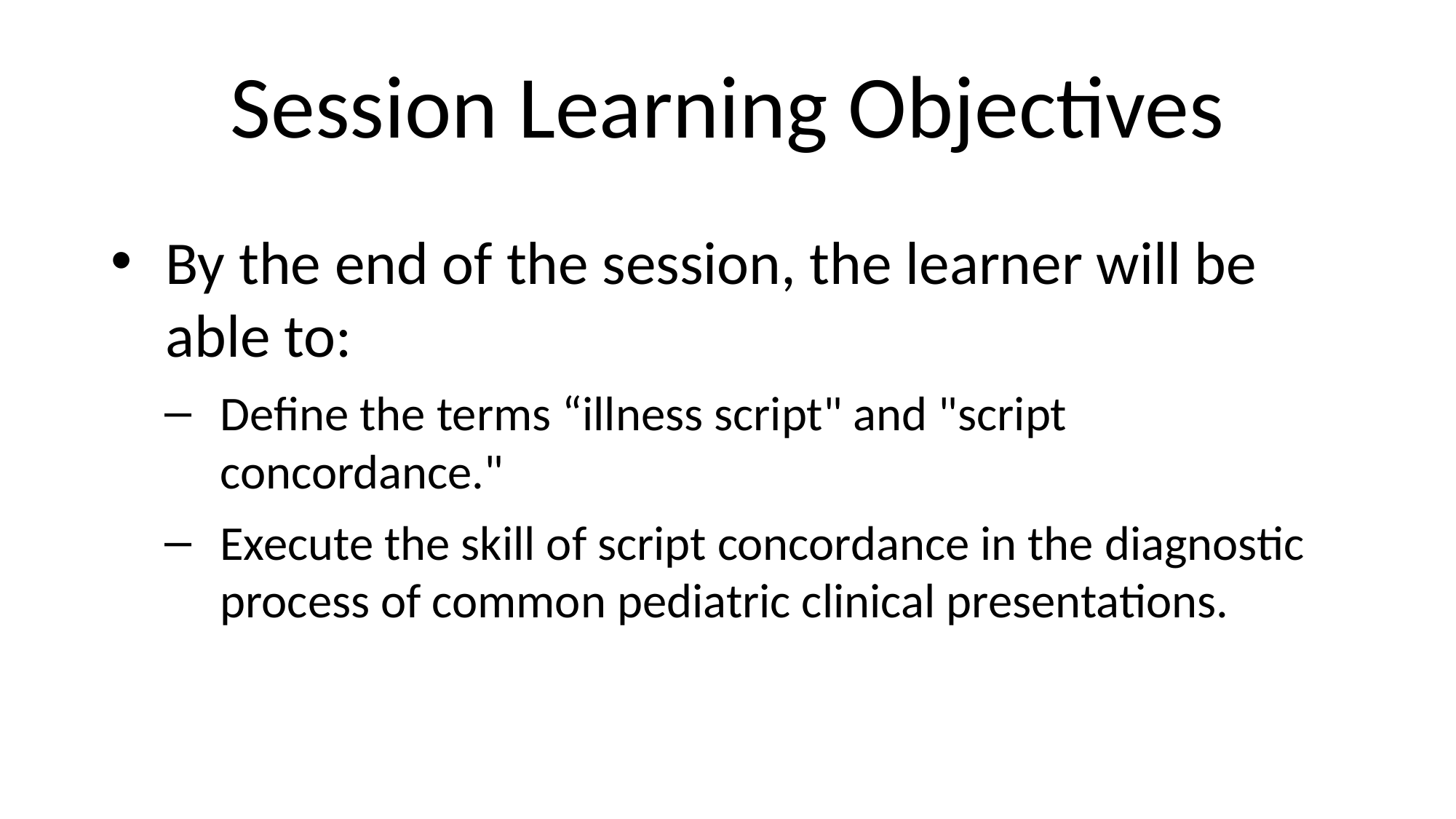

# Session Learning Objectives
By the end of the session, the learner will be able to:
Define the terms “illness script" and "script concordance."
Execute the skill of script concordance in the diagnostic process of common pediatric clinical presentations.

## Slide 7
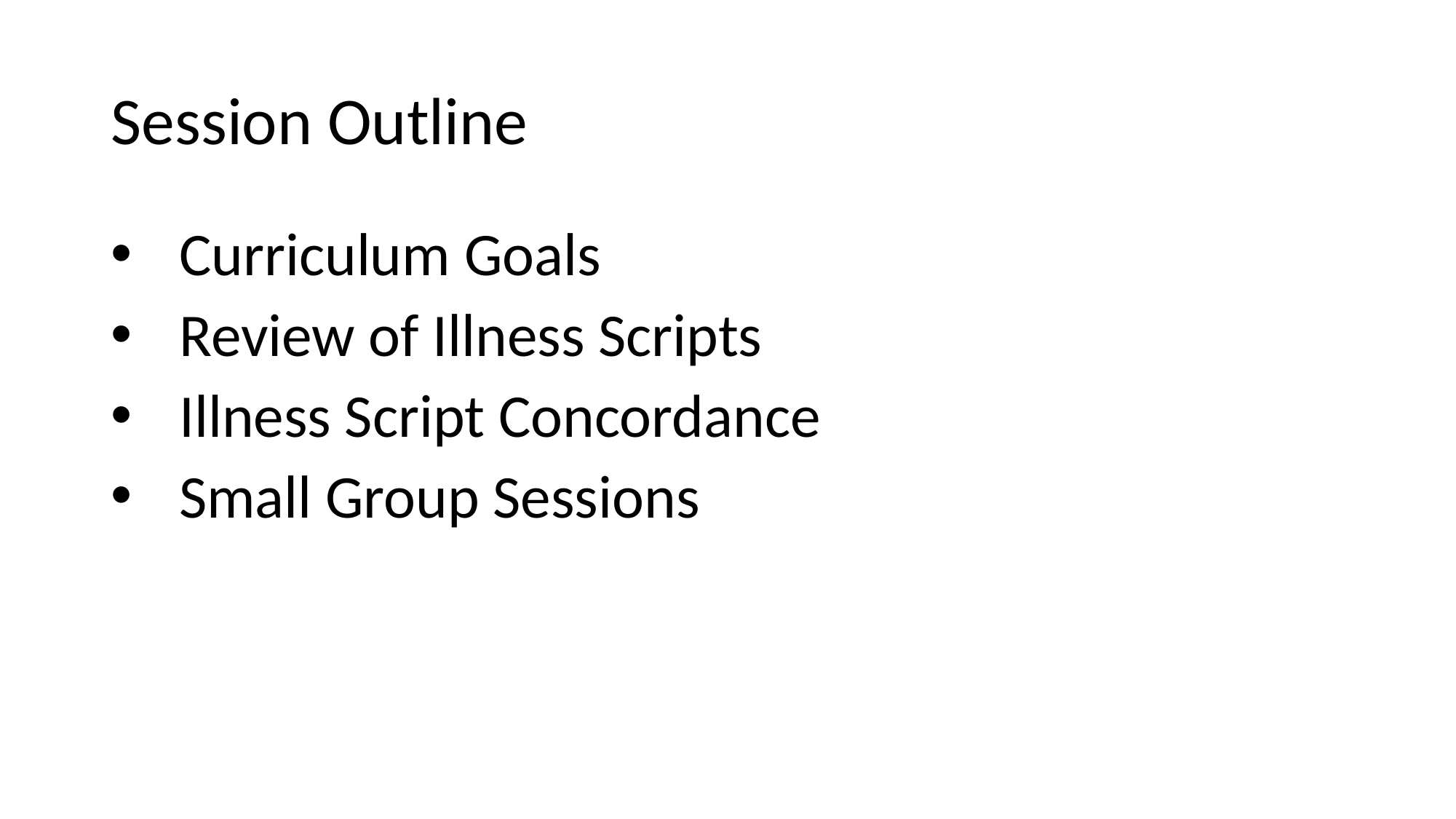

# Session Outline
Curriculum Goals
Review of Illness Scripts
Illness Script Concordance
Small Group Sessions

## Slide 8
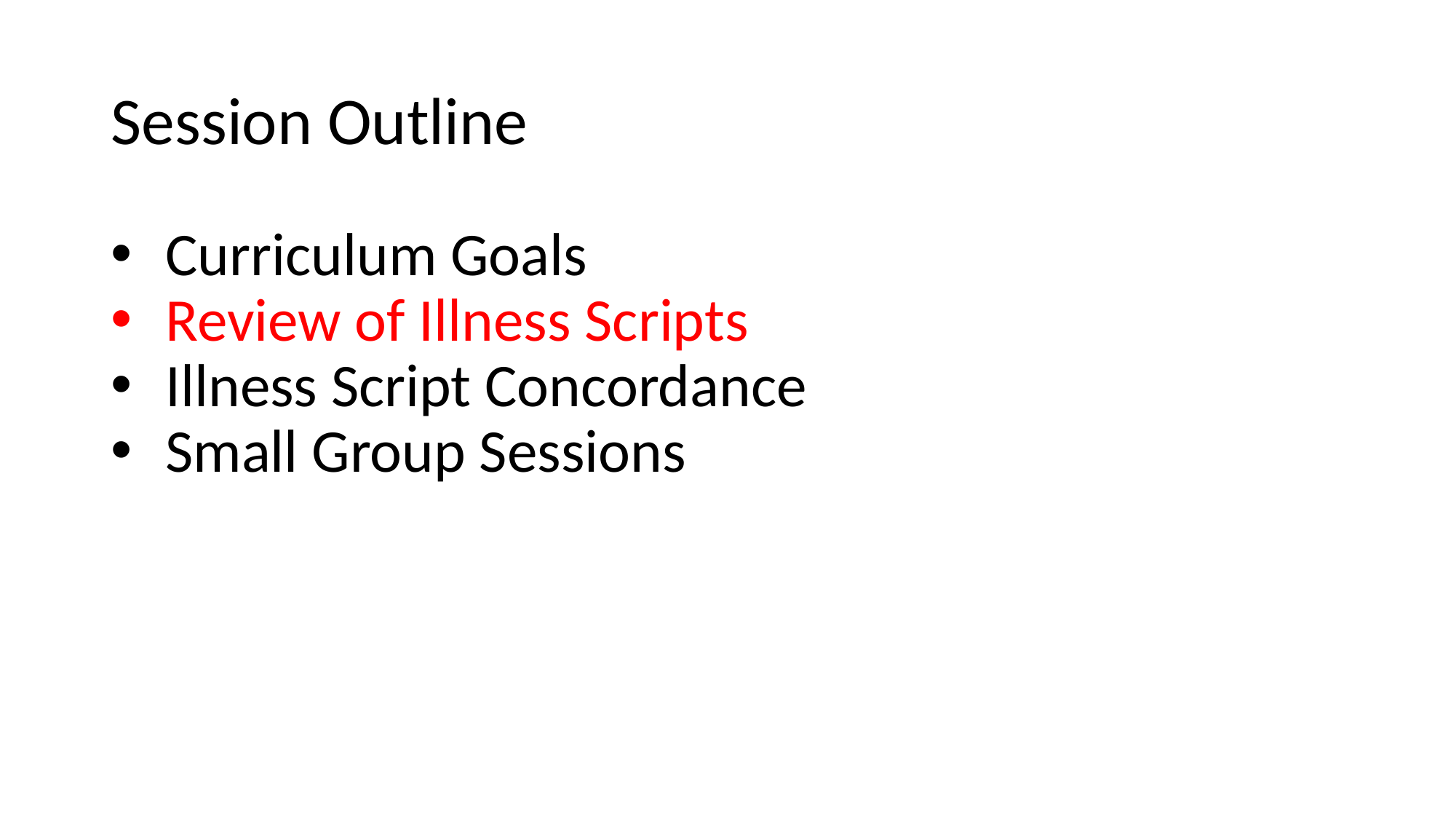

# Session Outline
Curriculum Goals
Review of Illness Scripts
Illness Script Concordance
Small Group Sessions

## Slide 9
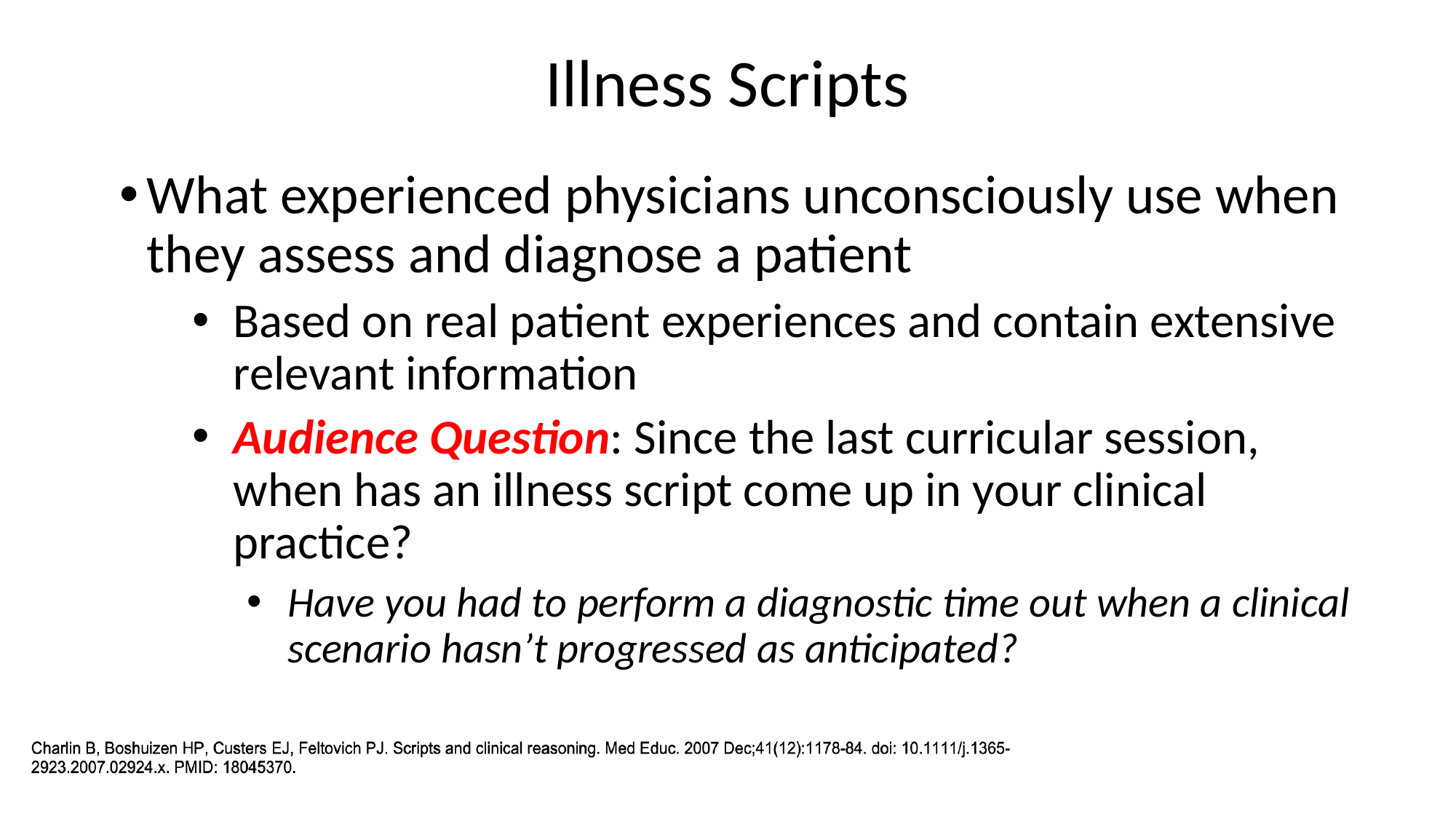

# Illness Scripts
What experienced physicians unconsciously use when they assess and diagnose a patient
Based on real patient experiences and contain extensive relevant information
Audience Question: Since the last curricular session, when has an illness script come up in your clinical practice?
Have you had to perform a diagnostic time out when a clinical scenario hasn’t progressed as anticipated?

## Slide 10
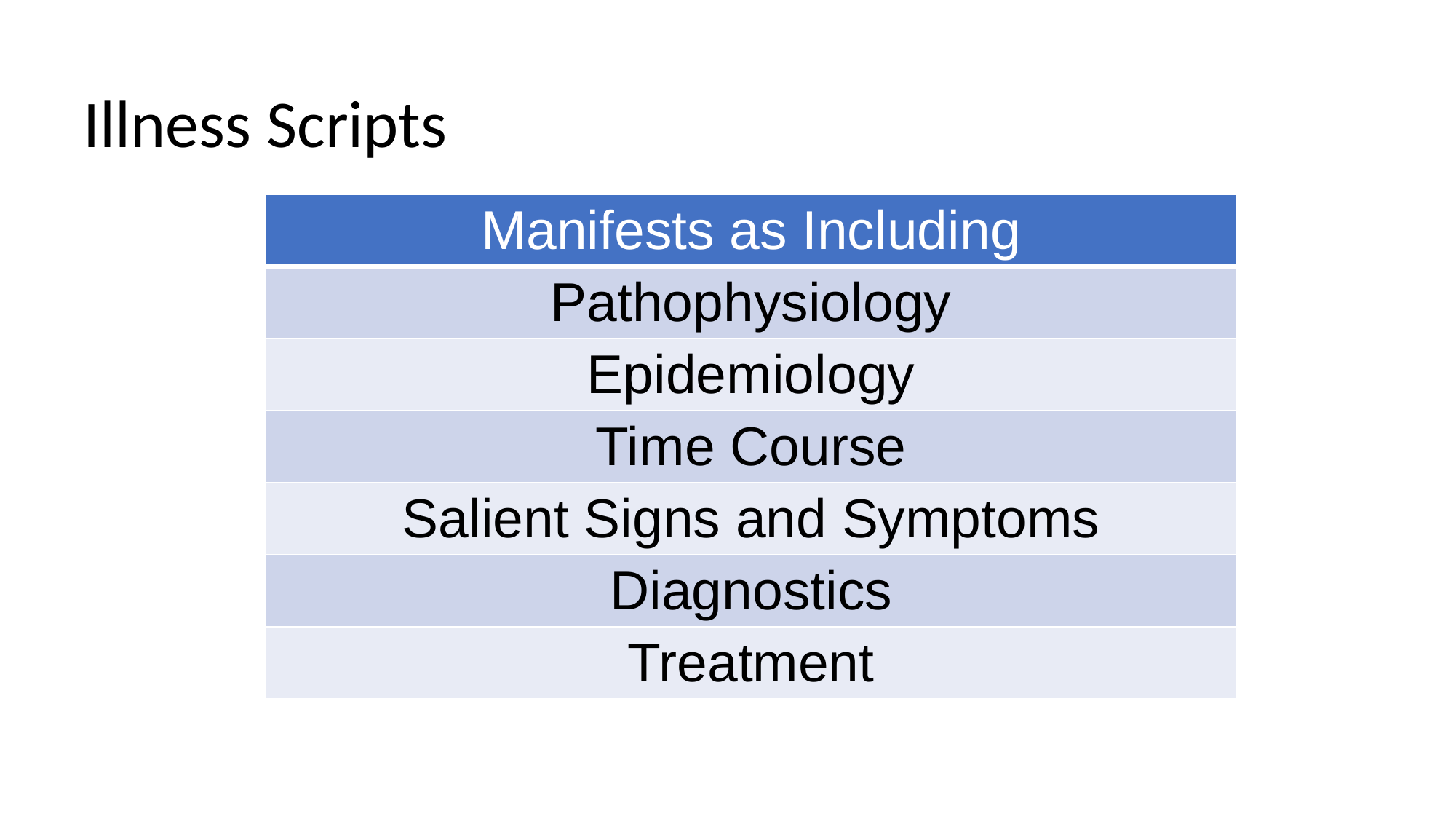

# Illness Scripts
| Manifests as Including |
| --- |
| Pathophysiology |
| Epidemiology |
| Time Course |
| Salient Signs and Symptoms |
| Diagnostics |
| Treatment |
| Must Include |
| --- |
| Predisposing Conditions |
| Pathophysiologic Insult |
| Clinical Consequences |

## Slide 11
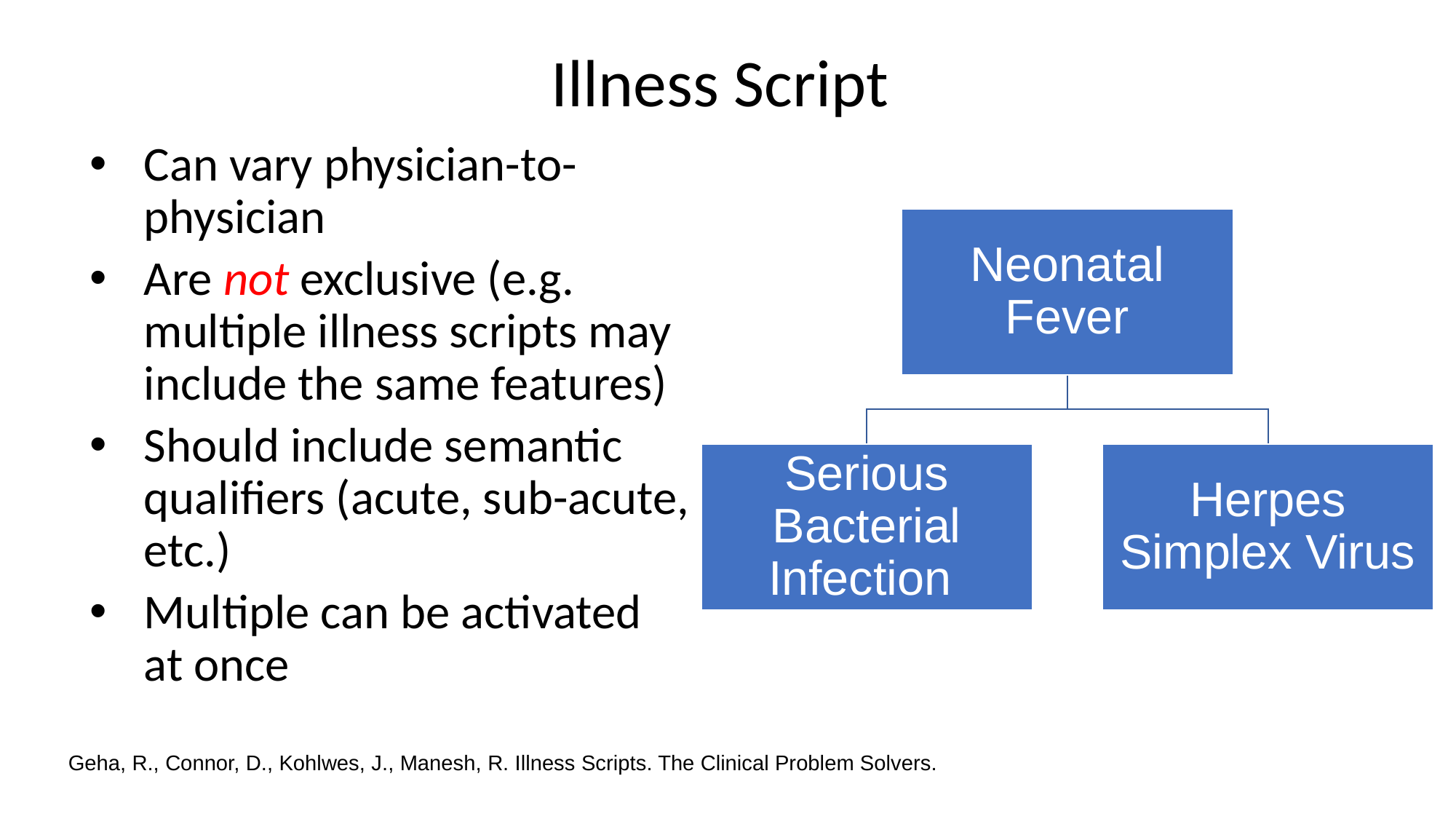

# Illness Script
Can vary physician-to-physician
Are not exclusive (e.g. multiple illness scripts may include the same features)
Should include semantic qualifiers (acute, sub-acute, etc.)
Multiple can be activated at once
Neonatal Fever
Serious Bacterial Infection
Herpes Simplex Virus
Geha, R., Connor, D., Kohlwes, J., Manesh, R. Illness Scripts. The Clinical Problem Solvers.

## Slide 12
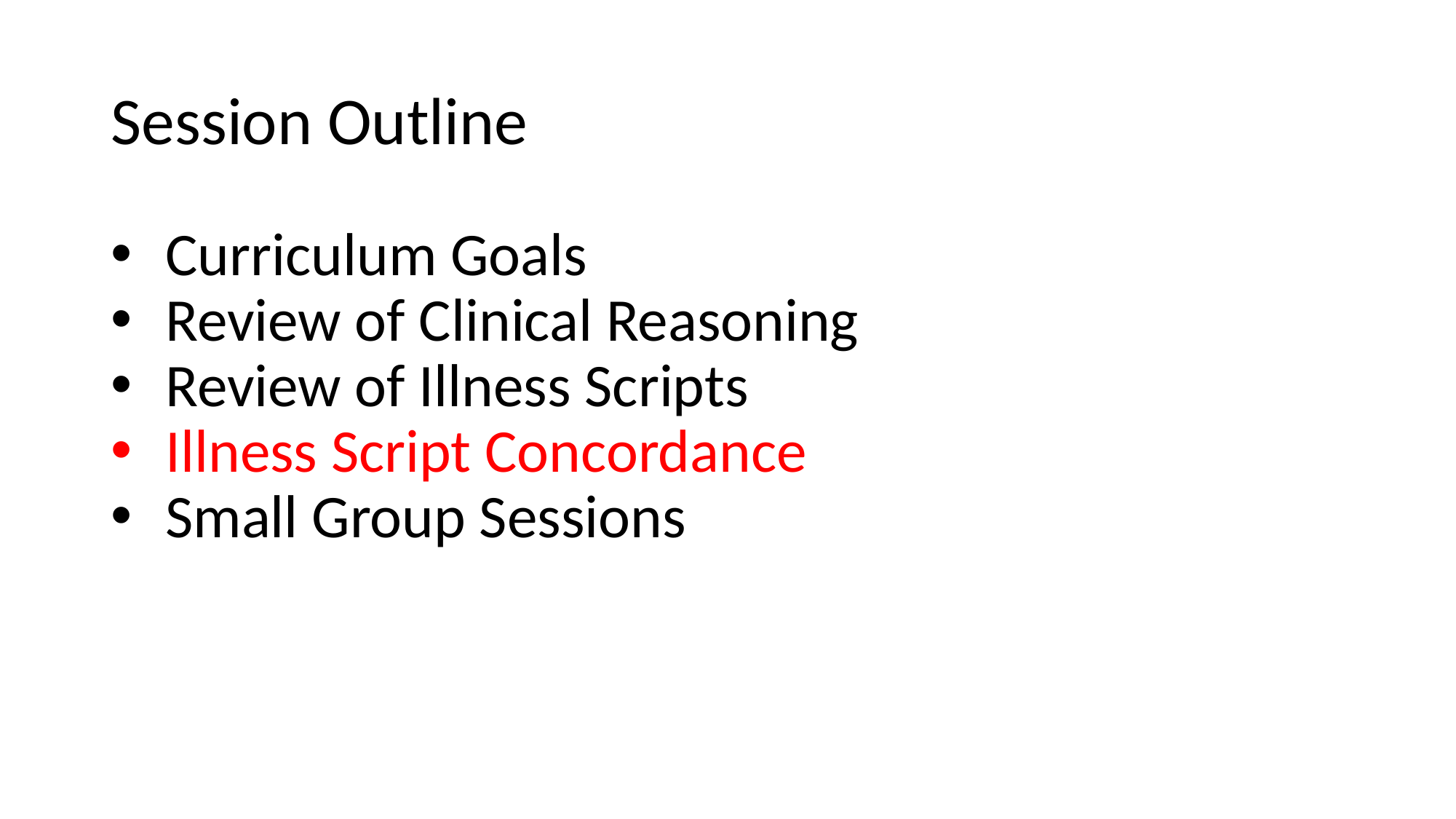

# Session Outline
Curriculum Goals
Review of Clinical Reasoning
Review of Illness Scripts
Illness Script Concordance
Small Group Sessions

## Slide 13
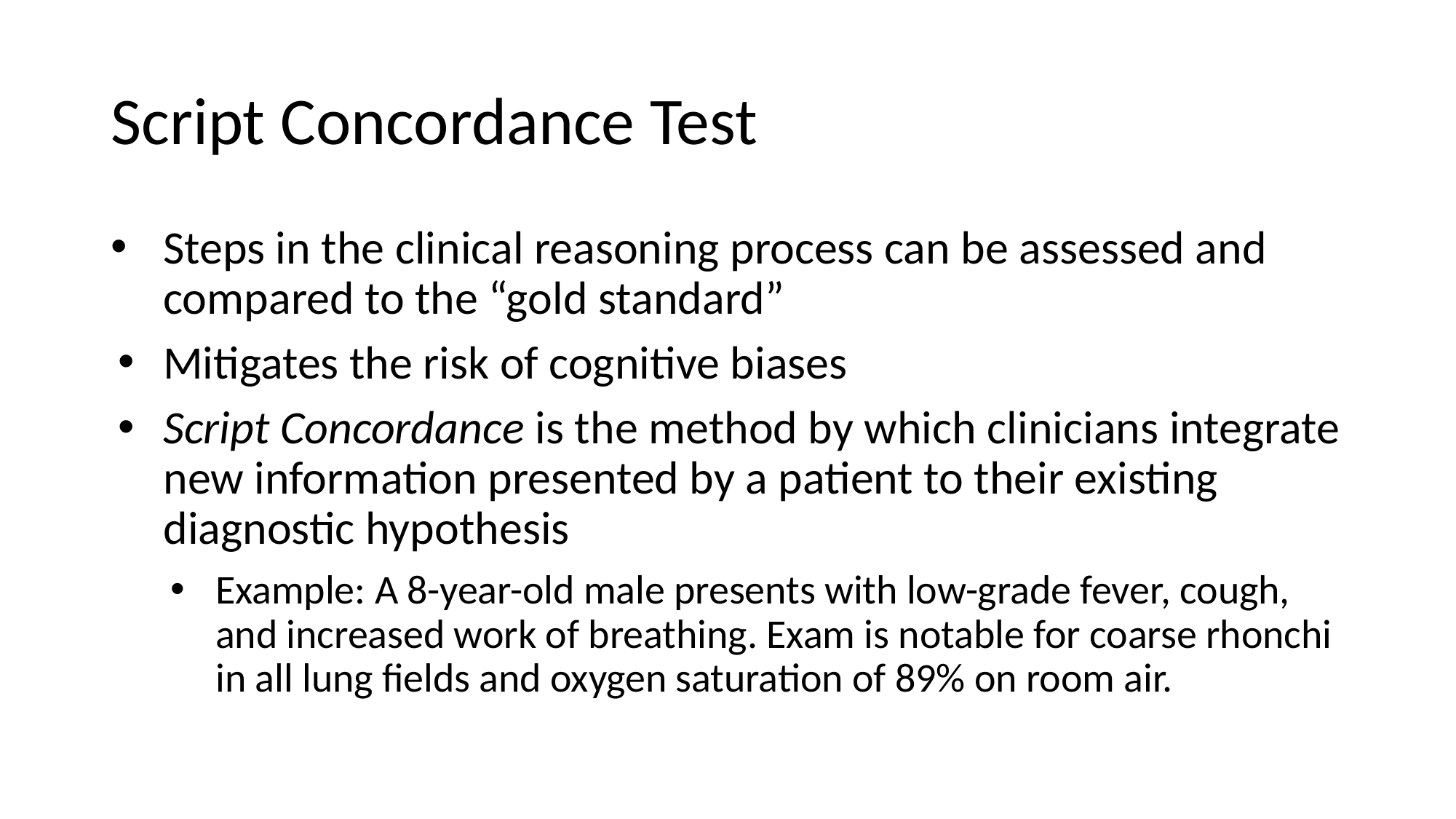

# Script Concordance Test
Steps in the clinical reasoning process can be assessed and compared to the “gold standard”
Mitigates the risk of cognitive biases
Script Concordance is the method by which clinicians integrate new information presented by a patient to their existing diagnostic hypothesis
Example: A 8-year-old male presents with low-grade fever, cough, and increased work of breathing. Exam is notable for coarse rhonchi in all lung fields and oxygen saturation of 89% on room air.

## Slide 14
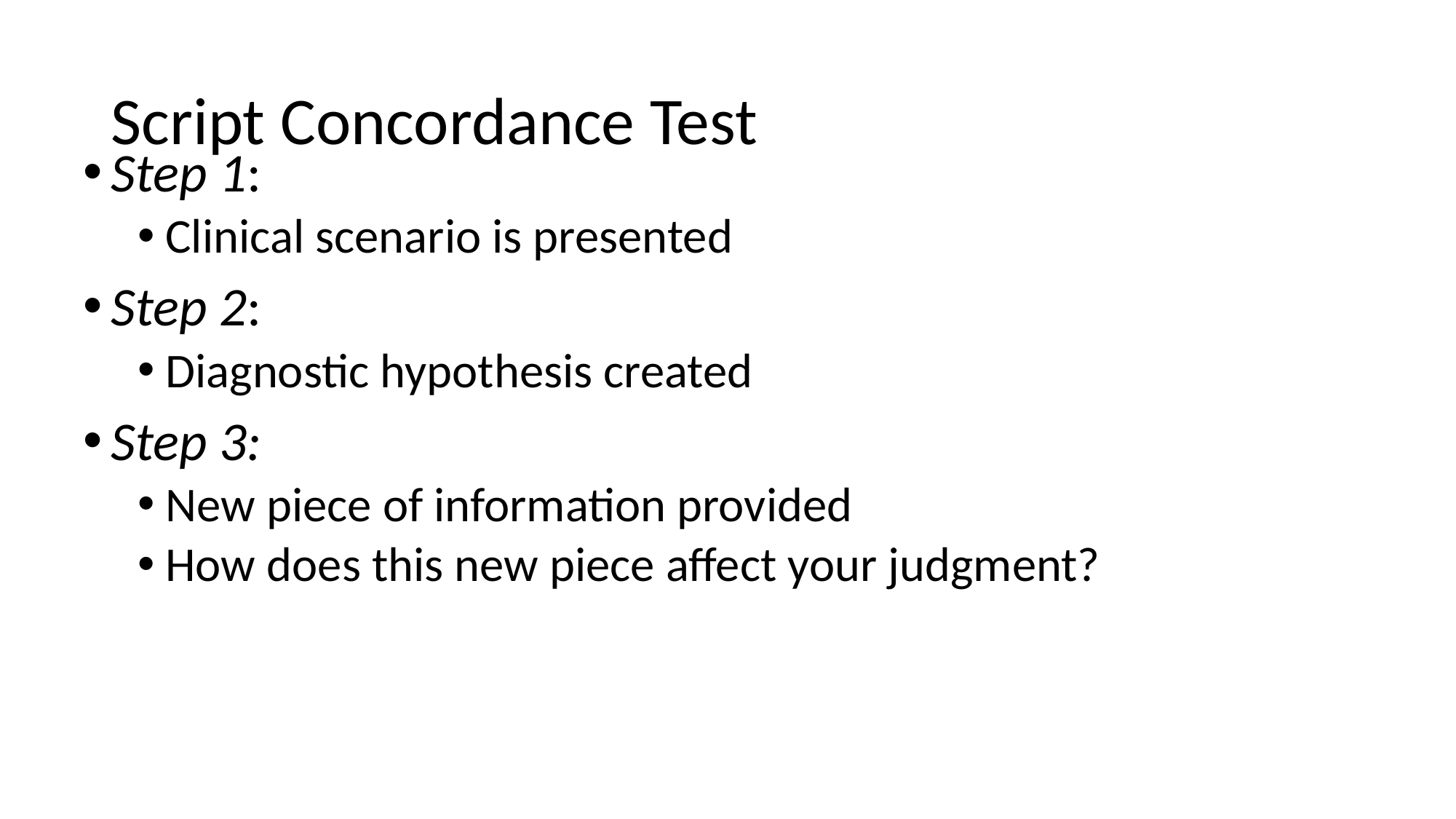

# Script Concordance Test
Step 1:
Clinical scenario is presented
Step 2:
Diagnostic hypothesis created
Step 3:
New piece of information provided
How does this new piece affect your judgment?

## Slide 15
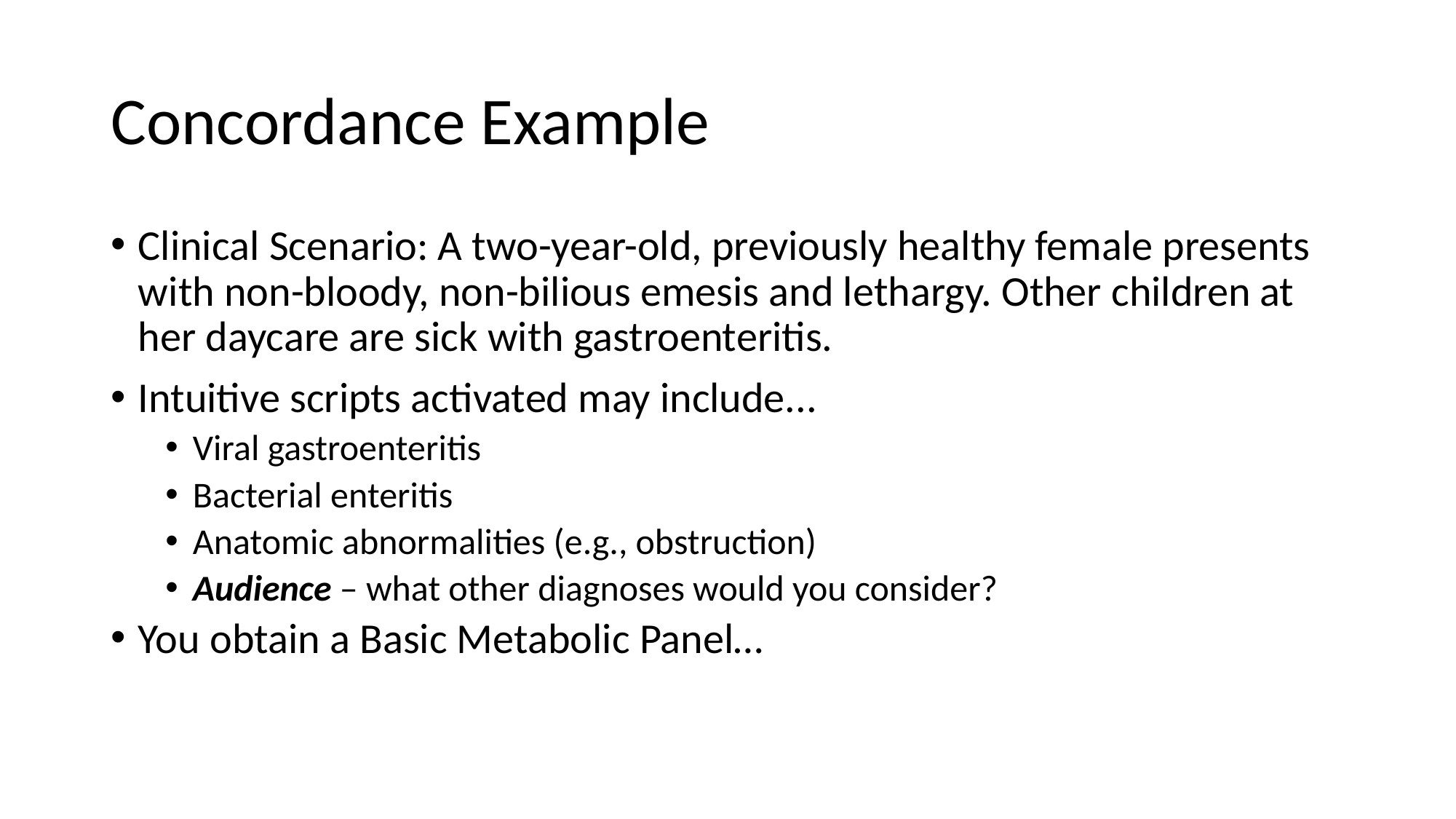

# Concordance Example
Clinical Scenario: A two-year-old, previously healthy female presents with non-bloody, non-bilious emesis and lethargy. Other children at her daycare are sick with gastroenteritis.
Intuitive scripts activated may include...
Viral gastroenteritis
Bacterial enteritis
Anatomic abnormalities (e.g., obstruction)
Audience – what other diagnoses would you consider?
You obtain a Basic Metabolic Panel…

## Slide 16
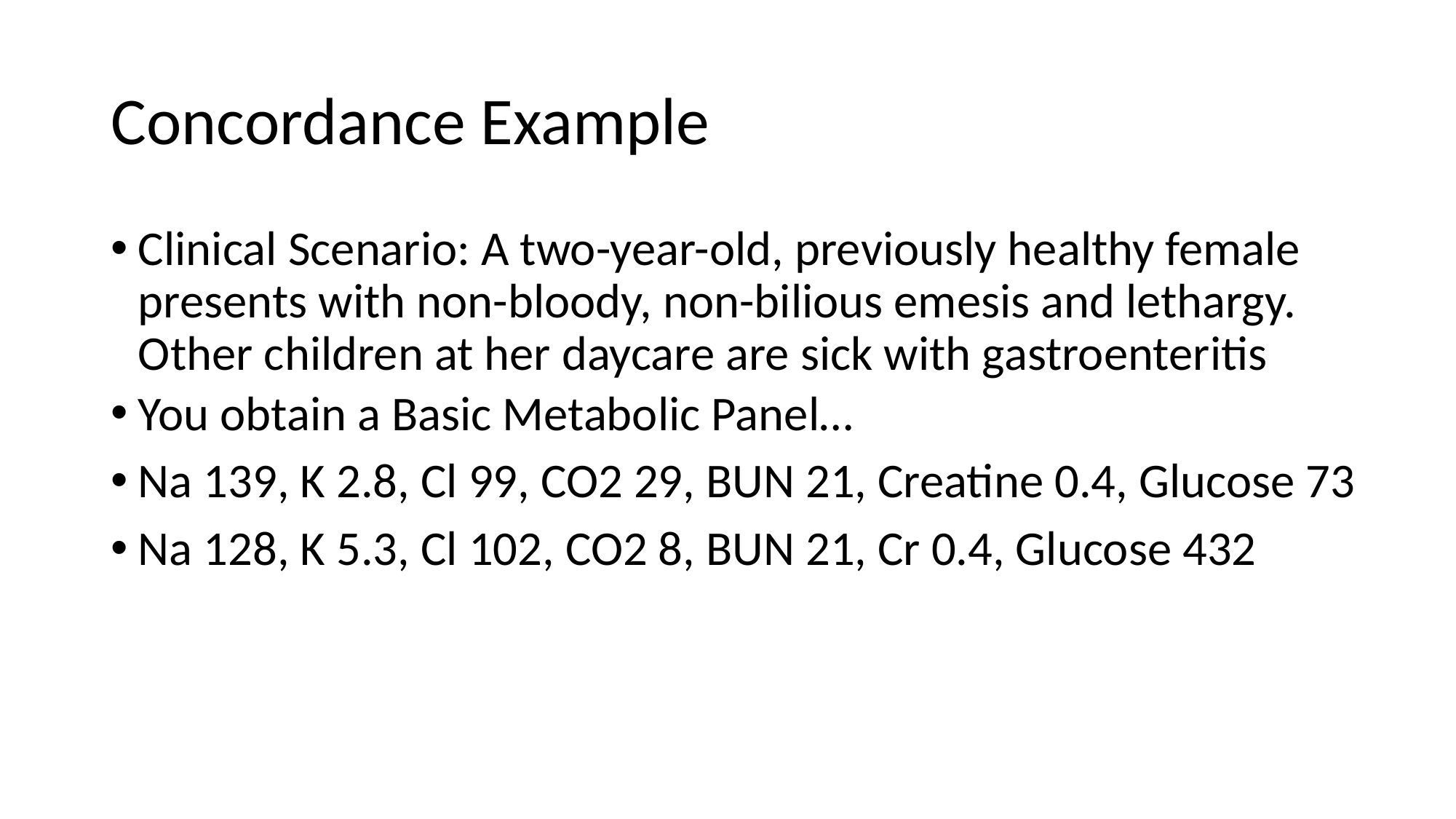

# Concordance Example
Clinical Scenario: A two-year-old, previously healthy female presents with non-bloody, non-bilious emesis and lethargy. Other children at her daycare are sick with gastroenteritis
You obtain a Basic Metabolic Panel…
Na 139, K 2.8, Cl 99, CO2 29, BUN 21, Creatine 0.4, Glucose 73
Na 128, K 5.3, Cl 102, CO2 8, BUN 21, Cr 0.4, Glucose 432

## Slide 17
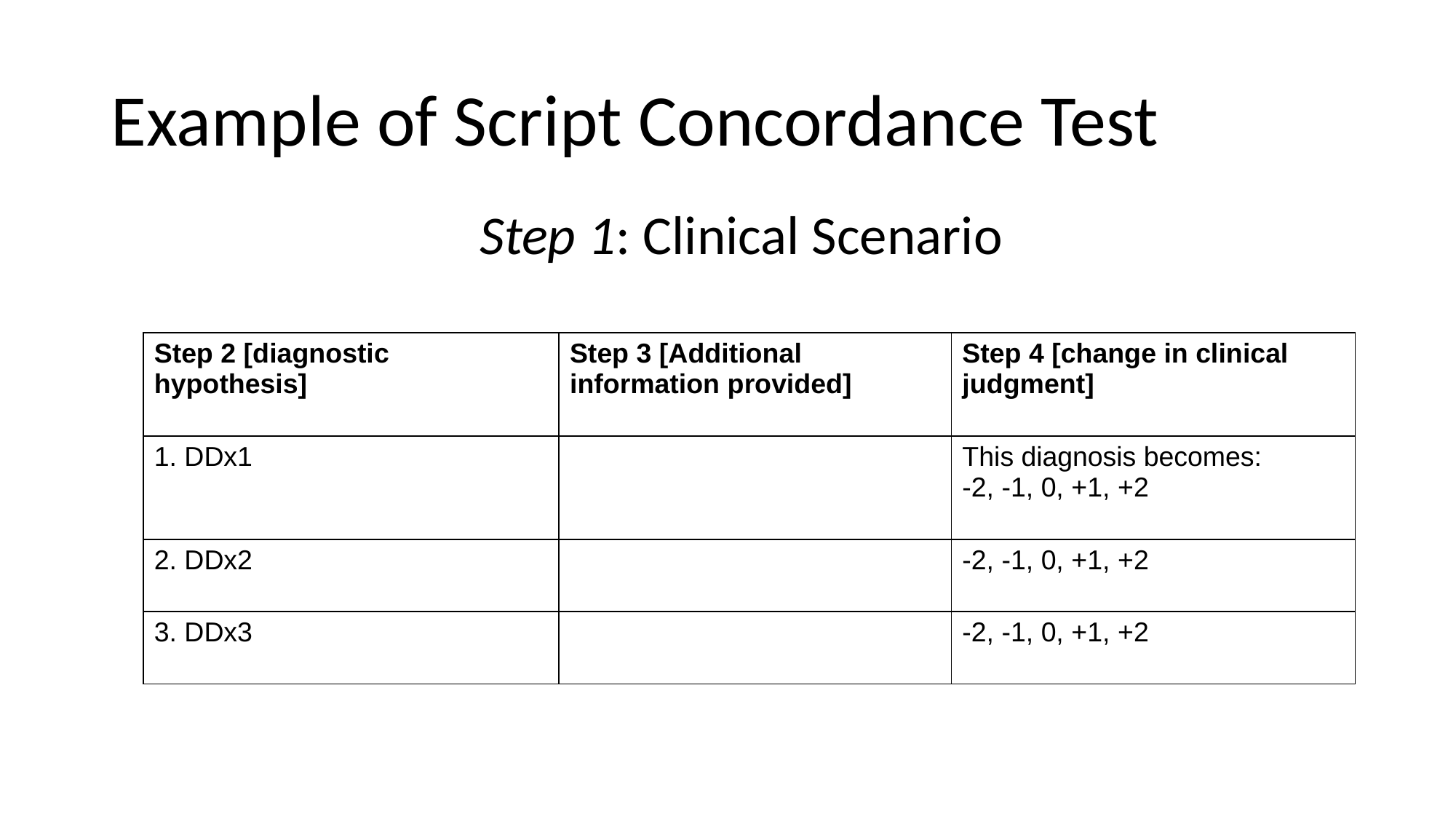

# Example of Script Concordance Test
Step 1: Clinical Scenario
| Step 2 [diagnostic hypothesis] | Step 3 [Additional information provided] | Step 4 [change in clinical judgment] |
| --- | --- | --- |
| 1. DDx1 | | This diagnosis becomes: -2, -1, 0, +1, +2 |
| 2. DDx2 | | -2, -1, 0, +1, +2 |
| 3. DDx3 | | -2, -1, 0, +1, +2 |

## Slide 18
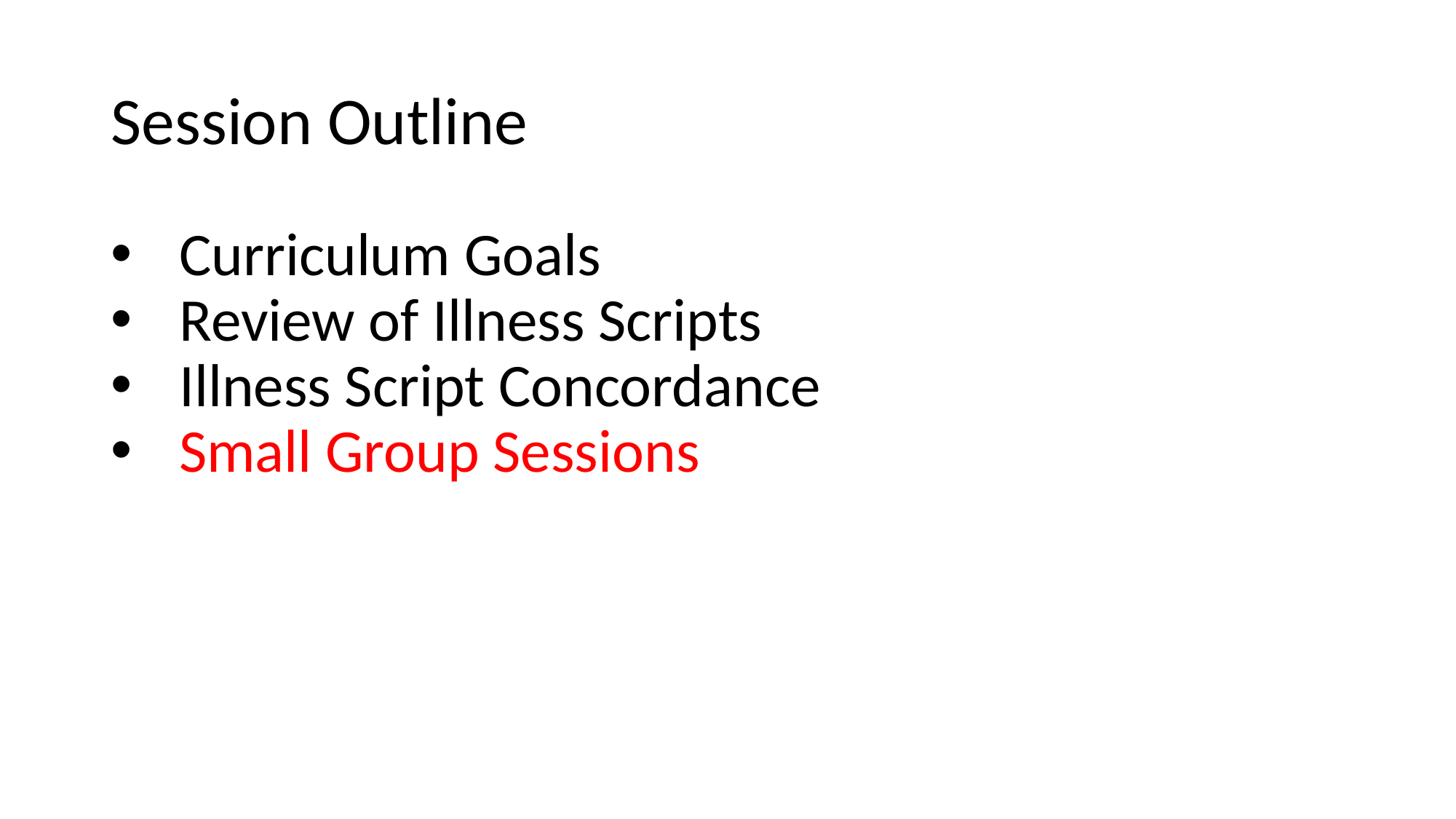

# Session Outline
Curriculum Goals
Review of Illness Scripts
Illness Script Concordance
Small Group Sessions

## Slide 19
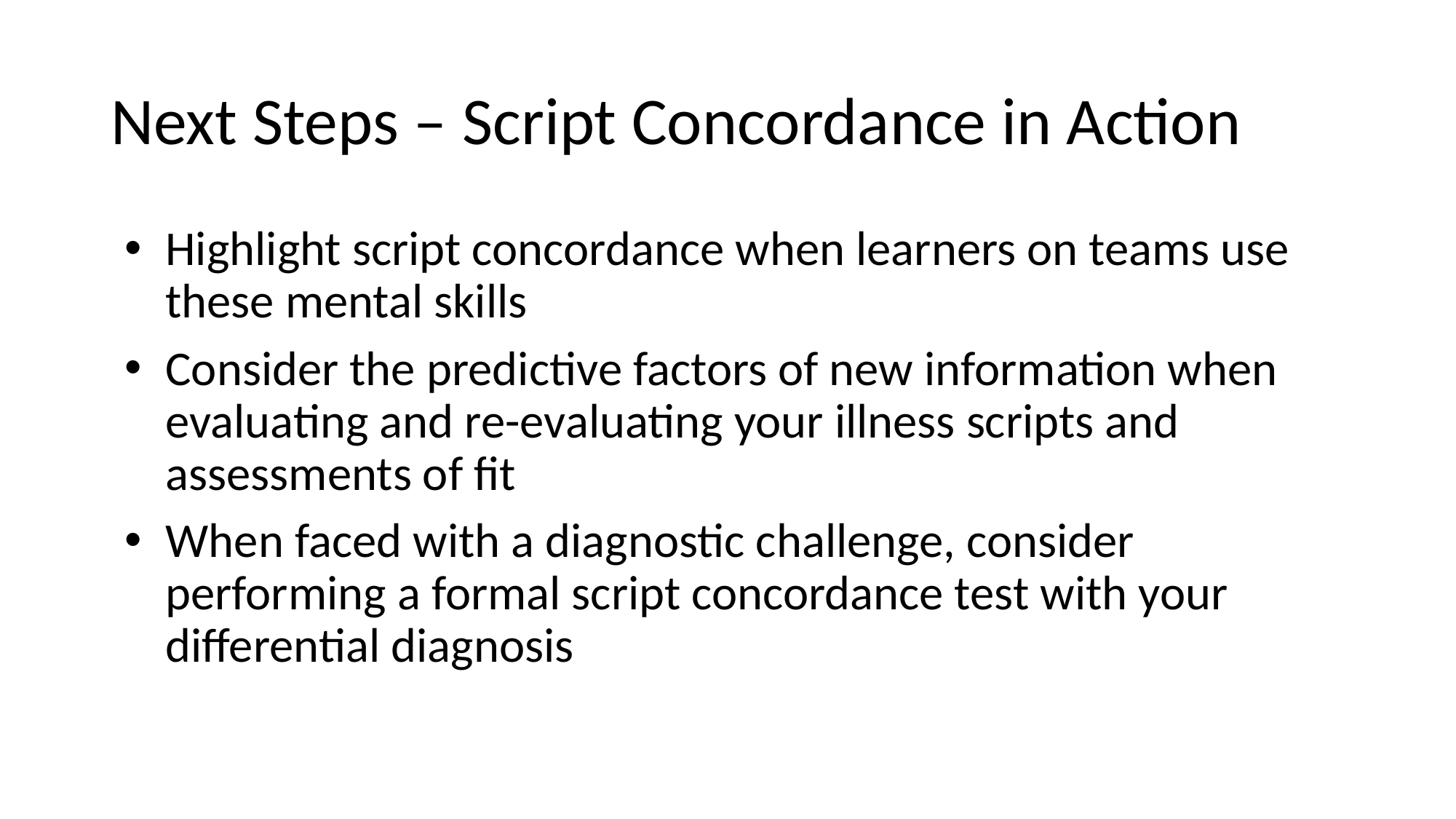

# Next Steps – Script Concordance in Action
Highlight script concordance when learners on teams use these mental skills
Consider the predictive factors of new information when evaluating and re-evaluating your illness scripts and assessments of fit
When faced with a diagnostic challenge, consider performing a formal script concordance test with your differential diagnosis

## Slide 20
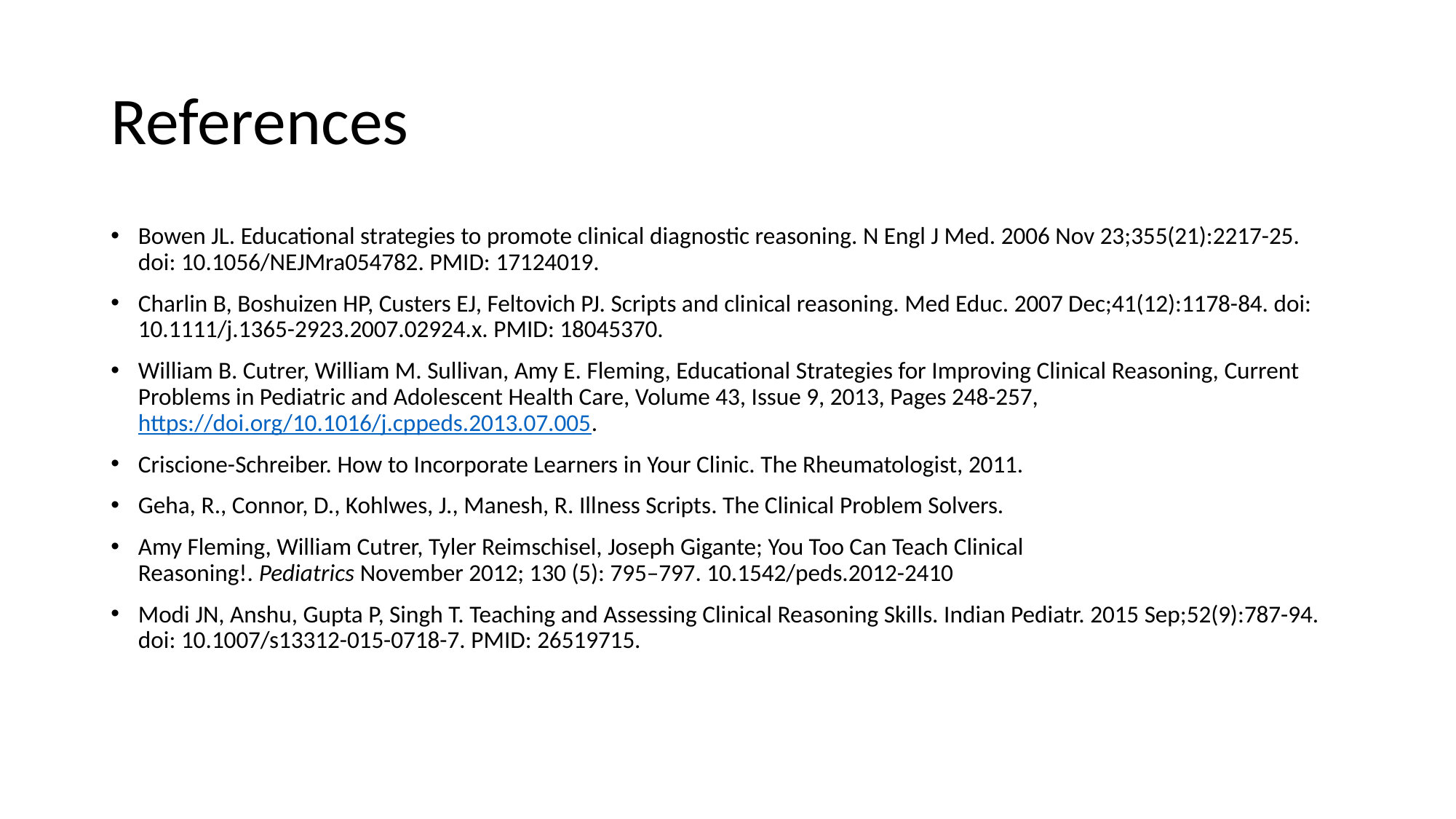

# References
Bowen JL. Educational strategies to promote clinical diagnostic reasoning. N Engl J Med. 2006 Nov 23;355(21):2217-25. doi: 10.1056/NEJMra054782. PMID: 17124019.
Charlin B, Boshuizen HP, Custers EJ, Feltovich PJ. Scripts and clinical reasoning. Med Educ. 2007 Dec;41(12):1178-84. doi: 10.1111/j.1365-2923.2007.02924.x. PMID: 18045370.
William B. Cutrer, William M. Sullivan, Amy E. Fleming, Educational Strategies for Improving Clinical Reasoning, Current Problems in Pediatric and Adolescent Health Care, Volume 43, Issue 9, 2013, Pages 248-257, https://doi.org/10.1016/j.cppeds.2013.07.005.
Criscione-Schreiber. How to Incorporate Learners in Your Clinic. The Rheumatologist, 2011.
Geha, R., Connor, D., Kohlwes, J., Manesh, R. Illness Scripts. The Clinical Problem Solvers.
Amy Fleming, William Cutrer, Tyler Reimschisel, Joseph Gigante; You Too Can Teach Clinical Reasoning!. Pediatrics November 2012; 130 (5): 795–797. 10.1542/peds.2012-2410
Modi JN, Anshu, Gupta P, Singh T. Teaching and Assessing Clinical Reasoning Skills. Indian Pediatr. 2015 Sep;52(9):787-94. doi: 10.1007/s13312-015-0718-7. PMID: 26519715.
