# Supplementary material for: A Pilot Longitudinal Clinical Reasoning Curriculum for Pediatric Residents
Source: MedEdPORTAL. 2024 Sep 25;20:11447. doi: 10.15766/mep_2374-8265.11447 (PMC11422513; doi:10.15766/mep_2374-8265.11447)
Supplement: Supplementary file 1 — Preimplementation Survey.docxCurriculum Goals, Objectives, and Timeline.docxSession 1 - Illness Scripts.pptxSession 1 - Small-Group Facilitator Guide.docxSession 2 - Illness Scripts 2.pptxSession 2 - Small-Group Facilitator Guide.docxSession 3 - Script Concordance.pptxSession 3 - Small-Group Facilitator Guide.docxSession 3 - Small-Group Handout.docxSession 4 - Pathophysiology.pptxSession 4 - Small-Group Facilitator Guide.docxSession 4 - Small-Group Handout.docxSession 5 - Review Game.pptxPostimplementation Survey.docx [file mep_2374-8265.11447-s001.zip › A. Preimplementation Survey.docx]

**Pre-Implementation Survey**. Disseminated via e-mail to resident physicians. Survey designed and results stored on Qualtrics.

| Question Prompt | Answer Choices |
| --- | --- |
| Indicate the last two letters of your last name and the last four digits of your phone number. | [Short Answer] |
| Indicate your current year of training (Post-Graduate Year, or PGY) | PGY1  PGY2  PGY3+ |
| Please indicate your agreement with the following statement: “I can define the term ‘illness script.’” | Disagree  Neutral  Agree |
| How often do you use illness scripts in your clinical practice? | Almost never  Rarely  Often  Almost every day  I don’t know what an illness script is |
| How comfortable are you anticipating abnormal results in diagnostic evaluations based upon your differential diagnosis? For example, expecting to see leukocytosis in the setting of presumed infection or expecting a high specific gravity on urinalysis in a patient with dehydration? | Extremely Uncomfortable  Somewhat Uncomfortable  Neither comfortable nor uncomfortable  Somewhat comfortable  Extremely comfortable |
| How comfortable are you modifying your differential diagnosis to guide further workup based upon new historical information? | Extremely Uncomfortable  Somewhat Uncomfortable  Neither comfortable nor uncomfortable  Somewhat comfortable  Extremely comfortable |
| How comfortable are you modifying your differential diagnosis to guide further workup based upon physical examination findings? | Extremely Uncomfortable  Somewhat Uncomfortable  Neither comfortable nor uncomfortable  Somewhat comfortable  Extremely comfortable |
| How comfortable are you modifying your differential diagnosis to guide further workup based upon new laboratory information? | Extremely Uncomfortable  Somewhat Uncomfortable  Neither comfortable nor uncomfortable  Somewhat comfortable  Extremely comfortable |
| How comfortable are you using organ system pathophysiology concepts to guide diagnostic reasoning?  For example, tachycardia occurs in the setting of hypovolemia to increase cardiac output to maintain end-organ perfusion.  Kussmaul Respirations occur in the setting of Diabetic Ketoacidosis due to respiratory compensation for metabolic acidosis. | Extremely Uncomfortable  Somewhat Uncomfortable  Neither comfortable nor uncomfortable  Somewhat comfortable  Extremely comfortable |
| These statements are quotes from the Accreditation Council for Graduate Medical Education milestone on Clinical Reasoning. Please select which statement you most identify with. | 1.Presents clinical facts (e.g. history, exam, consultations) in the order they were elicited  2. Generates an unfocused differential diagnosis based on the clinical facts  3. Organizes clinical facts to compare and contrast diagnoses being considered, resulting in a prioritized differential diagnosis  4. Integrates clinical facts into a unifying diagnosis(es); reevaluates diagnoses in real time to avoid diagnostic error  5. Role models and coaches the organization of clinical facts to develop a prioritized differential diagnosis, including life threatening diagnoses, atypical presentations, and complex clinical presentations |
| These statements are quotes from the Accreditation Council for Graduate Medical Education milestone on Differential Diagnosis. Please select which statement you most identify with. | 1. Lists basic evaluation (e.g. diagnostic testing and consultation) for common diagnoses, with prompting. Reports results of diagnostic studies.  2. Recommends broad evaluation based on an unfocused differential diagnosis. Identifies clinically significant diagnostic study results, with guidance.  3. Recommends focused evaluation based on a prioritized differential diagnosis. Interprets clinical significance of diagnostic study results.  4. Prioritizes and optimizes evaluation based on risks, benefits, indications, and alternatives to clarify the diagnosis(es).  5. Educates others about risks, benefits, indications, and alternatives to guide diagnostic decision-making. Teaches others to interpret clinically significant results and consider study limitations. |
