# Supplementary material for: A Pilot Longitudinal Clinical Reasoning Curriculum for Pediatric Residents
Source: MedEdPORTAL. 2024 Sep 25;20:11447. doi: 10.15766/mep_2374-8265.11447 (PMC11422513; doi:10.15766/mep_2374-8265.11447)
Supplement: Supplementary file 1 — Preimplementation Survey.docxCurriculum Goals, Objectives, and Timeline.docxSession 1 - Illness Scripts.pptxSession 1 - Small-Group Facilitator Guide.docxSession 2 - Illness Scripts 2.pptxSession 2 - Small-Group Facilitator Guide.docxSession 3 - Script Concordance.pptxSession 3 - Small-Group Facilitator Guide.docxSession 3 - Small-Group Handout.docxSession 4 - Pathophysiology.pptxSession 4 - Small-Group Facilitator Guide.docxSession 4 - Small-Group Handout.docxSession 5 - Review Game.pptxPostimplementation Survey.docx [file mep_2374-8265.11447-s001.zip › D. Session 1 - Small-Group Facilitator Guide.docx]

**Pediatric Advanced Clinical Reasoning Curriculum**

**Session #1 - Facilitator Guide**

**Suggested Timing for Session #1**

- Didactic Material
- Curriculum overview (Slides 1-5) - 2 minutes
- Overview of clinical reasoning (Slides 6-14) - 8 minutes
- Illness Scripts (Slides 15-24) - 10 minutes

**Small Group Activities (see Facilitator Guide Appendix D)**

- Explanation of activities (Slides 25-29) - 1 minute
- Activity 1 – 15 minutes
- Debrief activity 1 – 4 minutes
- Activity 2 – 15 minutes
- Debrief session – 5 minutes

**Directions for facilitators:** Each group is given the same chief complaint (16-year-old female with tachycardia for case 1, 16-year-old female with abdominal pain for case 2). They can ask 10 questions total. They do not have to ask all 10 questions and can share their working diagnosis at any time. The following should be used to answer their questions.

If they ask a question not listed below, the facilitator may answer in a way that would fit with the ultimate diagnosis. If the facilitator is unsure of how to answer, please answer “I don’t know” – the trainee group is allowed to ask a different question to replace the unanswered one.

**Case 1 - 16-year-old female with tachycardia**

**Group 1:**

Ultimate diagnosis: Thyrotoxicosis (**do not share this with the curriculum participants)**

| **Question** | **Answer** |
| --- | --- |
| Onset of Symptoms | One month prior to presentation |
| Describe the symptoms | Intermittent subjective tachycardia (“heart is racing” and palpitations (“my heart skips a beat”) |
| Any progression / why did they come in today? | Symptoms increasing in frequency, first noticed every 2 days, over the past 3 days have noticed every few hours |
| Change with activity? | Worsens with activity |
| Any weight changes? | 8-pound weight loss over illness course |
| Change in intake? | Maintained appetite, “if anything I feel like I’m eating more” |
| Any abnormal movements? | Has noticed shakiness over last two weeks (tremors) |
| Any bowel symptoms? | +Intermittent Non-bloody Diarrhea  No incontinence, no overnight stooling |
| Heat intolerance? | Yes, “always hot” |
| Visual changes? | No visual changes or exophthalmos |
| Any changes in hair? | Hair becoming more brittle and finer. Noticed more coming out with brushing |
| ROS | ROS negative for fever, SOB, headache, syncope, abdominal pain, chest pain, urinary symptoms, rash, bruising, arthralgia/arthritis, limp, menorrhagia |
| Past Medical History | None |
| Past Surgical History | None |
| Meds | None |
| Allergies | None |
| Immunizations | Up to date |
| Family History | Mom had her thyroid removed |
| Social History | Specifically denies ingested substances. Social history otherwise negative |

**Group 2 - 16-year-old female with tachycardia**

Ultimate diagnosis: Arrhythmia (Supraventricular Tachycardia) **- (do not share this with the curriculum participants)**

| **Question** | **Answer** |
| --- | --- |
| Onset of Symptoms | One week ago |
| Describe the symptoms | Discrete periods of feeling like heart was racing. Lasts a few minutes per episode. |
| When did they start? | One period was while playing soccer. Fainted after symptoms started, woke up immediately. |
| Any seizure-like activity? | No postictal state, incontinence, cyanosis, shaking, eye deviation, or tongue injury. |
| Any chest pain? | No chest pain but feels “weird.” Like heart is fluttering. |
| Any shortness-of-breath (SOB)? | Mild SOB during symptoms. |
| Any recent illnesses | No |
| ROS | ROS negative for fever, headache, vision changes, abdominal pain, rash, bruising, arthralgia, urinary changes, bowel/bladder changes. |
| Menstrual History | Normal periods, last menstrual period was 2 weeks ago. “PCP said they were normal.” |
| Meds | None |
| Allergies | None |
| Past Medical History | None |
| Past Surgical History | None |
| Immunizations | UTD |
| Family History | Uncle with “heart problems.” No further details. |
| Social History | Social history negative |

**Group 3 - 16-year-old female with tachycardia**

Ultimate Diagnosis: Anemia due to menorrhagia (**do not share this with the curriculum participants)**

| **Question** | **Answer** |
| --- | --- |
| Onset of Symptoms | Two months ago, ongoing, and progressive |
| Why presenting today? | After an episode of fainting while walking in school. At the nurse’s office, was found to have a heart rate of 120. |
| Any seizure-like activity? | No shaking, postictal period, cyanosis, incontinence, tongue injury, eye deviation (NO activity) |
| Any other symptoms? | Fatigue, quit the soccer team because of it. Mom felt she looked more pale. |
| Menstrual History | Last menstrual period was one week ago. Menarche at 11. Periods last 12 days. |
| Pad/Tampon use | Goes through a Jumbo Pad every 2 hours. Wakes up overnight to change pad. +Clots |
| ROS | Negative for fever, URI symptoms, increased WOB, chest pain, abdominal pain, diarrhea, constipation, rash, bruising. |
| Hematochezia or melena? | No |
| Urinary symptoms? | No gross hematuria |
| Other bleeding? | Nosebleeds intermittently. Lasts 35-40 minutes. Never seen a physician for them. |
| Any changes in hair? | Hair is becoming more brittle and finer. Noticed more coming out with brushing |
| ROS | ROS negative for fever, SOB, headache, syncope, abdominal pain, chest pain, urinary symptoms, rash, bruising, arthralgia/arthritis, limp, menorrhagia |
| Past Medical History | None |
| Past Surgical History | None |
| Meds | None |
| Allergies | None |
| Immunizations | UTD |
| Family History: | Mom with heavy periods. |
| Social History: | Sexually active with one male partner. Uses barrier contraception 10/10 times. Social history is otherwise negative. |

**Group 4 - 16-year-old female with tachycardia**

Ultimate diagnosis: Cocaine use with associated angina **(do not share this with the trainee group).**

| Question | Answer |
| --- | --- |
| Onset of Symptoms | Two hours prior to the presentation. Went to the nurse’s office for chest pain and was found to have a HR of 140. |
| Location? | Substernal chest pain and tightness. |
| Radiating? | No |
| Progression? | Nonprogressive but has not gotten better since it started. |
| Quantity of pain | 8/10 |
| Palpitations? | Heart feels like it is fluttering. |
| Any other symptoms? | Nurse said pupils looked big. +mild sweating and mild shakiness. |
| Ever happened before? | No |
| Any bleeding? | Two nosebleeds over the last two weeks. Stopped spontaneously and did not see a doctor. No other bleeding. |
| ROS | Negative for fever, SOB, headache, syncope, abdominal pain, chest pain, urinary symptoms, rash, bruising, arthralgia/arthritis, limp, menorrhagia |
| Past Medical History | None |
| Past Surgical History | None |
| Meds | None |
| Allergies | None |
| Immunizations | UTD |
| Family History | None |
| Social History | Endorses making new friends after having been bullied at previous school.  Recently quit soccer team to hang out with friends (18-years-old, just graduated). Denies sexual activity. Endorses cigarette use but denies other substance use. |

**Group 5 - 16-year-old female with tachycardia**

Diagnosis: Pulmonary Embolism (**do not share this with the curriculum participants)**

| Question | Answer |
| --- | --- |
| Onset of Symptoms | Three hours again |
| Describe the symptoms | Acute onset 5/10 chest pain, sharp |
| Location | Right of the sternum |
| Quality? | Hurts with deep breathes, non-radiating |
| Why did you come in? | Apple watch notified patient of high HR (130s) |
| Taking any meds to help? | Motrin did not help pain |
| Any respiratory symptoms? | No cough or increased work-of-breathing |
| ROS | Negative for fever, headache, vision changes, nausea, vomiting, abdominal pain, GI symptoms, urinary symptoms, bruising, bleeding |
| Fainting? | Felt lightheaded w/o fainting |
| Past Medical History | Menorrhagia |
| Past Surgical History | None |
| Meds | Combined oral contraceptive pill |
| Allergies | None |
| Immunizations | UTD |
| Family History | None |
| Social History | Has smoked 3-5 cigarettes w/ friends for the past few months. Social history is otherwise negative.  Recently returned via car ride from Disney World the night before symptoms started. |

**Case #2 – 16-year-old female with abdominal pain**

Group 1

Diagnosis - Appendicitis (**do not share this with the curriculum participants)**

| Question | Answer |
| --- | --- |
| Onset and Duration of Symptoms | Abruptly, approximately 24 hours prior to presentation |
| Location? | Right lower quadrant |
| Quality? | Sharp, aching |
| Radiating? | No |
| Progression? | Seems to be steadily worsening. Tough time sleeping last night due to pain. The pain initially was initially periumbilical but migrated to the RLQ over the last 12 hours |
| Quantity of pain | 9/10 |
| Aggravating Factor? | Changing position (especially hip flexion) |
| Relieving Factors & Treatments? | None. Ibuprofen minimally helpful. |
| Anorexia? | Yes |
| Any other symptoms? | Nausea, x1 episode of NBNB emesis, feeling warm but has not taken a temperature at home |
| Ever happened before? | No |
| Bowel movements | No bowel movements since onset of pain but daily soft stools prior to onset |
| ROS | Negative for recorded fever, headache, URI symptoms, CP, SOB, urinary symptoms, rash, bruising, arthralgia/arthritis, Limp (though RLQ pain with ambulation) |
| Past Medical History | Anxiety |
| Past Surgical History | None |
| Meds | None |
| Allergies | None |
| Immunizations | UTD |
| Family History | Mother with history of IBS and anxiety |
| Social History | Lives at home with parents, 2 siblings and a dog. Nobody at home with similar symptoms.    Sees a therapist weekly for anxiety. Denies sexual activity, has a boyfriend. Denies all substance use. |

**Group 2 - 16-year-old female with abdominal pain**

Diagnosis - Cholecystitis (**do not share this with the curriculum participants)**

| Question | Answer |
| --- | --- |
| Onset and Duration of Symptoms | Somewhat suddenly started 2-3 days ago |
| Location? | Right upper quadrant |
| Quality? | Sharp, waxes and wanes |
| Radiating? | Seems to radiate to right side/back/shoulder |
| Progression? | Waxes and wanes but steadily uncomfortable and seem to be worsening |
| Quantity of pain | 7/10 |
| Aggravating Factor? | Deep inspiration. Eating – especially larger meals. |
| Relieving Factors & Treatments? | Ibuprofen is helpful. |
| Anorexia? | No, but pain seems to worsen following meals and thus has been eating less |
| Any other symptoms? | Mild nausea, fever of 100.6 noted today |
| Ever happened before? | No |
| Bowel movements | Has been having one 1-2 soft BMs daily |
| ROS | Negative for headache, vomiting/diarrhea, URI symptoms, SOB, urinary symptoms, rash, bruising, arthralgia/arthritis    Positive for CP with deep inspiration |
| Past Medical History | Obesity, polycystic ovarian syndrome |
| Past Surgical History | T&A during grade school years |
| Meds | OCP |
| Allergies | None |
| Immunizations | UTD |
| Family History | Mother and Father both with history of T2DM |
| Social History | Splits time between mother and father’s home who are divorced. No siblings.    Grades have recently been poor due to virtual learning format. Denies current anxiety or depression. Has used marijuana socially a handful of times but denies other substance use |

**Group 3 - 16-year-old female with abdominal pain**

Diagnosis - Ovarian Torsion (**do not share this with the curriculum participants)**

| Question | Answer |
| --- | --- |
| Onset and Duration of Symptoms | Abruptly started 1 hour ago |
| Location? | RLQ |
| Quality? | Sharp, intense |
| Radiating? | No |
| Progression? | Unrelenting constant severe pain |
| Quantity of pain | 10/10 |
| Aggravating Factor? | Movement |
| Relieving Factors & Treatments? | None |
| Anorexia? | N/A |
| Any other symptoms? | x1 episode of NBNB emesis |
| Ever happened before? | No. Had 2-3 days of intermittent mild achiness also in the RLQ area |
| Bowel movements | Stools every 1-2 days. Sometimes hard BMs but no straining. Last BM yesterday. |
| ROS | Negative for fever, headache, URI symptoms, diarrhea, CP, SOB, urinary symptoms, rash, bruising, arthralgia/arthritis |
| Past Medical History | Intermittent asthma |
| Past Surgical History | None |
| Meds | Cetirizine, Albuterol inhaler PRN |
| Allergies | Seasonal |
| Immunizations | UTD |
| Family History | Father with history of childhood asthma and seasonal allergies |
| Social History | Lives with Mother, father, 1 sibling, 2 dogs.    Denies Depression/Anxiety. Denies all substance use. Sexually active with one male partner and intermittently uses condoms. |

**Group 4 - 16-year-old female with abdominal pain**

Diagnosis - Inflammatory Bowel Disease (**do not share this with the curriculum participants).**

| Question | Answer |
| --- | --- |
| Onset and Duration of Symptoms | Subacute started 7-10 days ago |
| Location? | Generalized |
| Quality? | Achiness, uncomfortable |
| Radiating? | No |
| Progression? | Steady, pain waxes and wanes |
| Quantity of pain | 4/10 |
| Aggravating Factor? | N/A |
| Relieving Factors & Treatments? | Bowel rest |
| Anorexia? | No |
| Any other symptoms? | Seen by PCP 4 days ago. 10-pound weight loss noted. Celiac panel negative. Has been having intermittent diarrhea, usually upon awakening x3 weeks – sometimes blood noted on toilet paper. Sometimes having urgency to stool. Has been feeling more fatigued than usual. |
| Ever happened before? | Has had intermittent mild abdominal pain in the past year but never like this. |
| Bowel movements | 3-4 stools per day. Often diarrhea consistency. Often with blood on toilet paper. Sometimes bloody appearance to stool/toilet water. |
| ROS | Negative for fever, headache, URI symptoms, CP, SOB, vomiting, urinary symptoms, rash, bruising, arthralgia/arthritis, hot/cold intolerance |
| Past Medical History | None |
| Past Surgical History | Appendectomy 8 years ago |
| Meds | None |
| Allergies | None |
| Immunizations | UTD |
| Family History | Mother with history of hypothyroidism |
| Social History | Lives with Mother, father, 2 siblings, 1 cat, one snake.    Some mild anxiety but denies depressive thoughts. Denies all substance use. Denies sexual activity. |

**Group 5 - 16-year-old female with abdominal pain**

Diagnosis - Gastroesophageal Reflux Disease/Gastritis (**do not share this with the curriculum participants)**

| Question | Answer |
| --- | --- |
| Onset and Duration of Symptoms | Fluctuating over the last six months |
| Location? | Mid-epigastrum |
| Quality? | Burning, tingling |
| Radiating? | Also feels pain higher up in the midline of the chest |
| Progression? | Not worsening overall in intensity. |
| Quantity of pain | 2/10 |
| Aggravating Factor? | Eating large meals, spicy foods |
| Relieving Factors & Treatments? | Ibuprofen does not help. Acetaminophen does not help. Tums helps |
| Anorexia? | No |
| Any other symptoms? | Mild sore throat |
| Ever happened before? | Every so often throughout the last few years |
| Bowel movements | Once daily, soft, non-bloody |
| ROS | Negative for recorded fever, headache, URI symptoms, chest pain, shortness of breath, urinary symptoms, rash, bruising, arthralgia/arthritis, vomiting, diarrhea, constipation |
| Past Medical History | Anxiety |
| Past Medical History | None |
| Meds | Tums, ibuprofen, acetaminophen PRN |
| Allergies | None |
| Immunizations | UTD |
| Family History | Mother with history of IBS and anxiety |
| Social History | Lives at home with parents, 2 siblings and a dog. Nobody at home with similar symptoms.    Sees a therapist weekly for anxiety. Denies sexual activity, has a boyfriend. Denies all substance use. |
