# Supplementary material for: A Pilot Longitudinal Clinical Reasoning Curriculum for Pediatric Residents
Source: MedEdPORTAL. 2024 Sep 25;20:11447. doi: 10.15766/mep_2374-8265.11447 (PMC11422513; doi:10.15766/mep_2374-8265.11447)
Supplement: Supplementary file 1 — Preimplementation Survey.docxCurriculum Goals, Objectives, and Timeline.docxSession 1 - Illness Scripts.pptxSession 1 - Small-Group Facilitator Guide.docxSession 2 - Illness Scripts 2.pptxSession 2 - Small-Group Facilitator Guide.docxSession 3 - Script Concordance.pptxSession 3 - Small-Group Facilitator Guide.docxSession 3 - Small-Group Handout.docxSession 4 - Pathophysiology.pptxSession 4 - Small-Group Facilitator Guide.docxSession 4 - Small-Group Handout.docxSession 5 - Review Game.pptxPostimplementation Survey.docx [file mep_2374-8265.11447-s001.zip › E. Session 2 - Illness Scripts 2.pptx]

## Slide 1
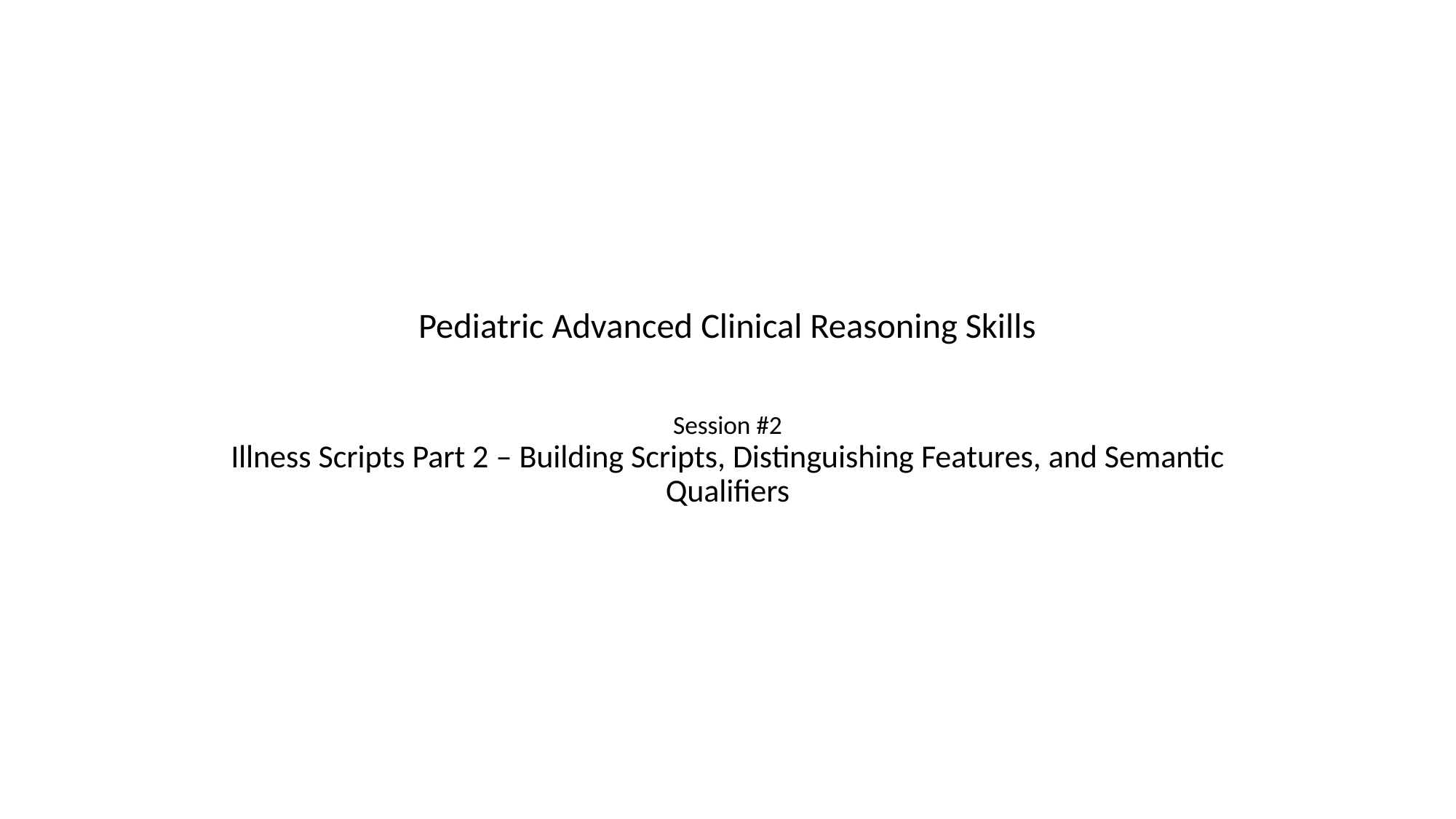

# Pediatric Advanced Clinical Reasoning Skills Session #2 Illness Scripts Part 2 – Building Scripts, Distinguishing Features, and Semantic Qualifiers

## Slide 2
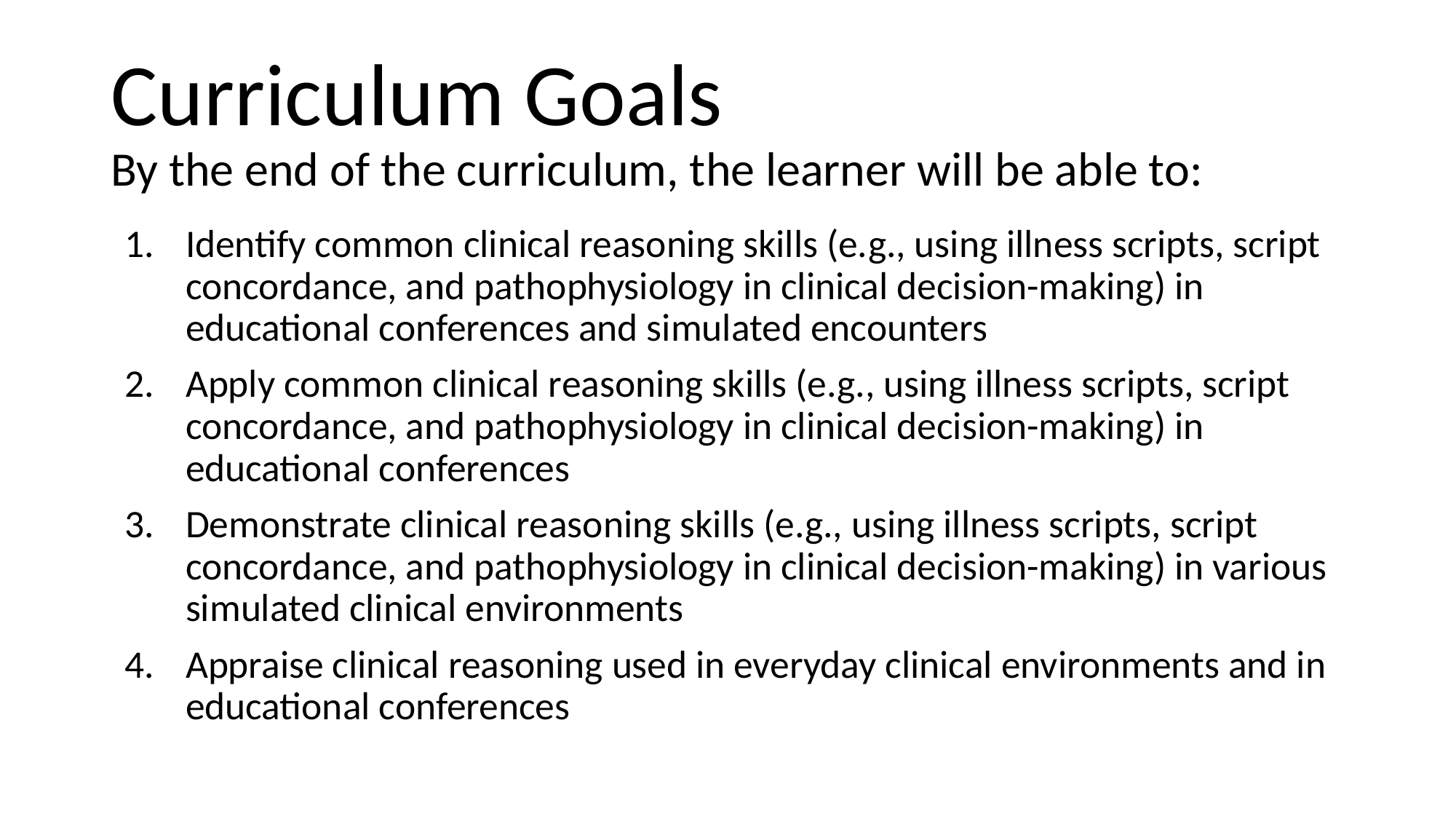

# Curriculum Goals By the end of the curriculum, the learner will be able to:
Identify common clinical reasoning skills (e.g., using illness scripts, script concordance, and pathophysiology in clinical decision-making) in educational conferences and simulated encounters
Apply common clinical reasoning skills (e.g., using illness scripts, script concordance, and pathophysiology in clinical decision-making) in educational conferences
Demonstrate clinical reasoning skills (e.g., using illness scripts, script concordance, and pathophysiology in clinical decision-making) in various simulated clinical environments
Appraise clinical reasoning used in everyday clinical environments and in educational conferences

## Slide 3
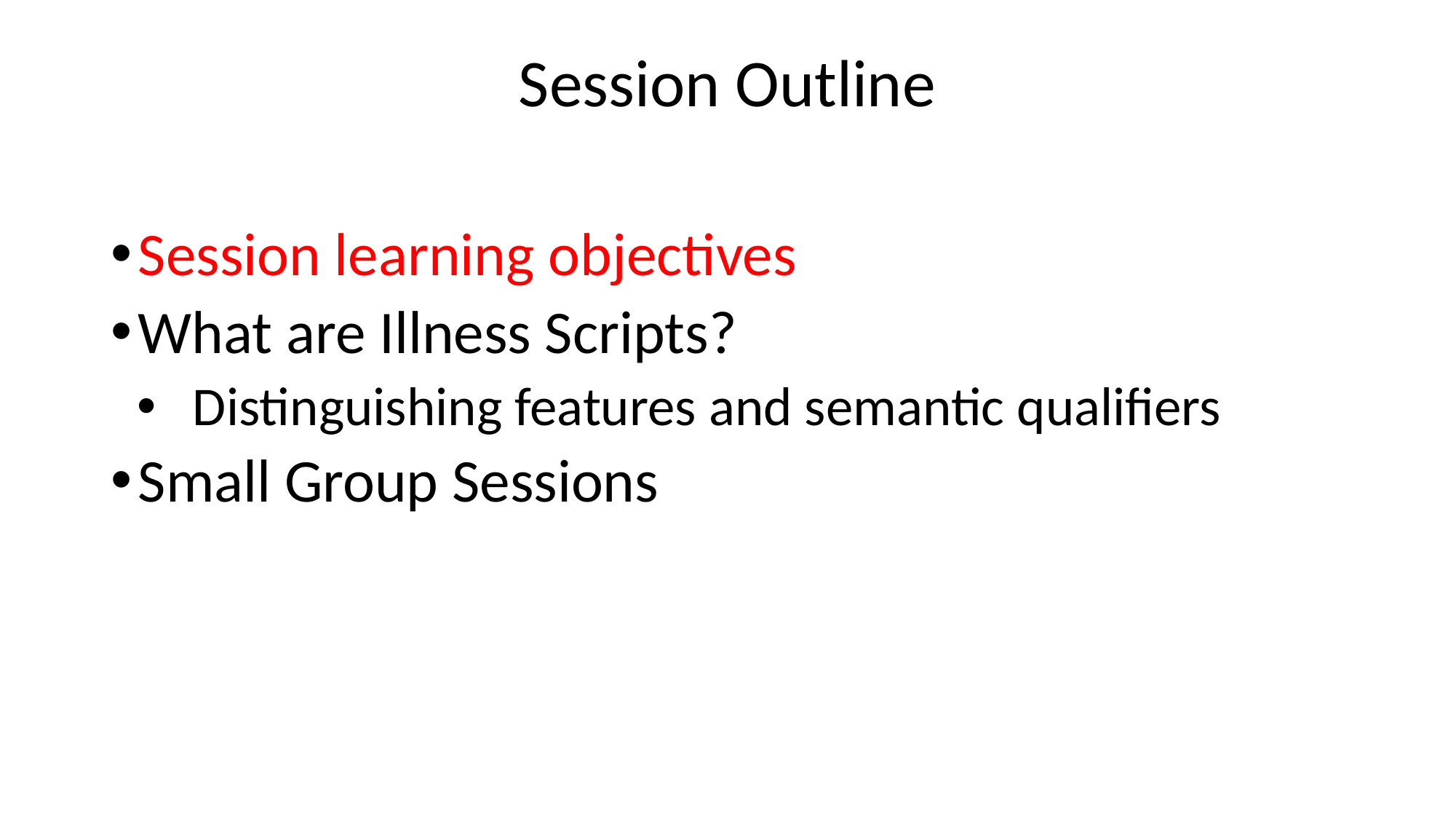

# Session Outline
Session learning objectives
What are Illness Scripts?
Distinguishing features and semantic qualifiers
Small Group Sessions

## Slide 4
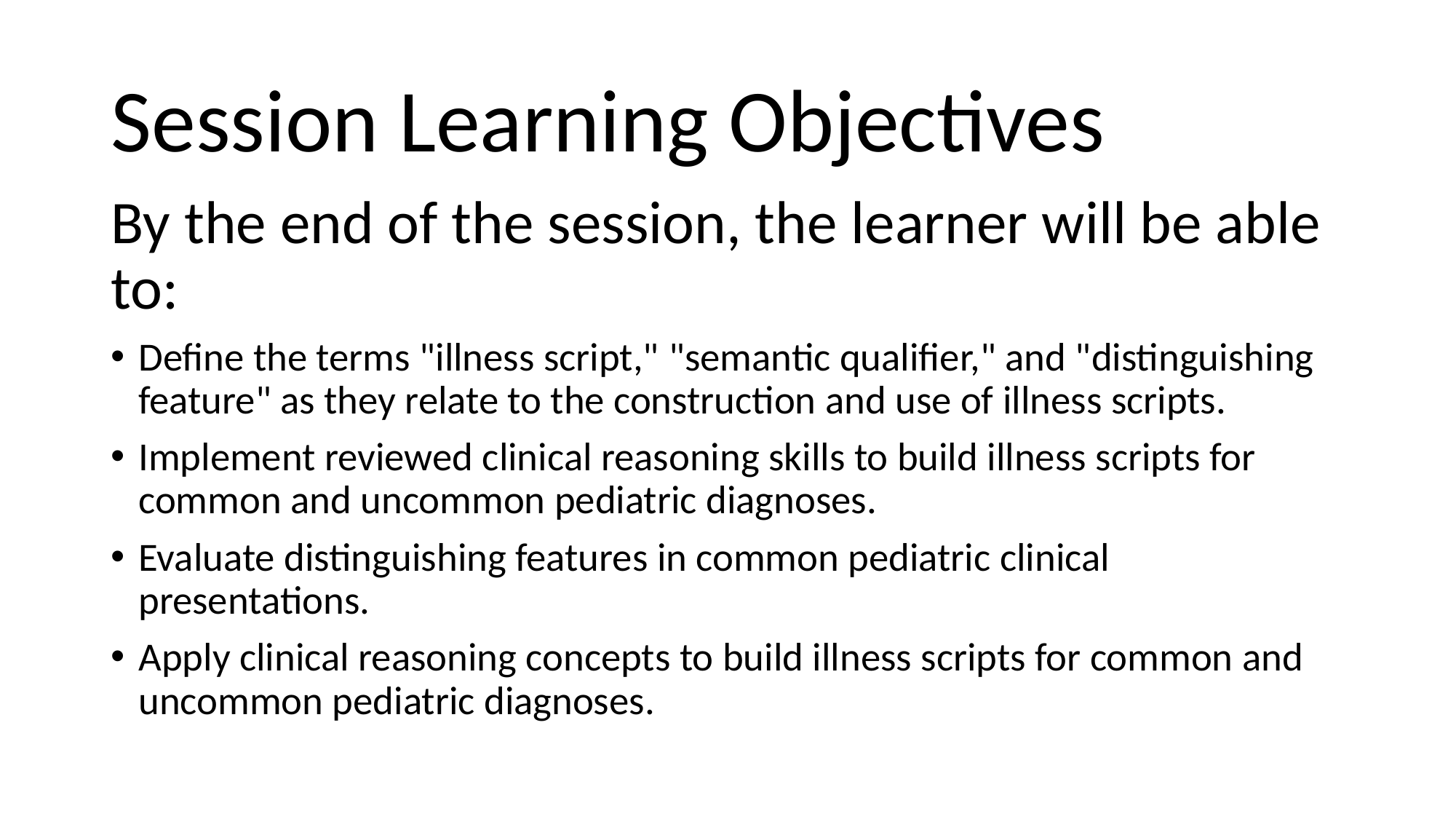

# Session Learning Objectives
By the end of the session, the learner will be able to:
Define the terms "illness script," "semantic qualifier," and "distinguishing feature" as they relate to the construction and use of illness scripts.
Implement reviewed clinical reasoning skills to build illness scripts for common and uncommon pediatric diagnoses.
Evaluate distinguishing features in common pediatric clinical presentations.
Apply clinical reasoning concepts to build illness scripts for common and uncommon pediatric diagnoses.

## Slide 5
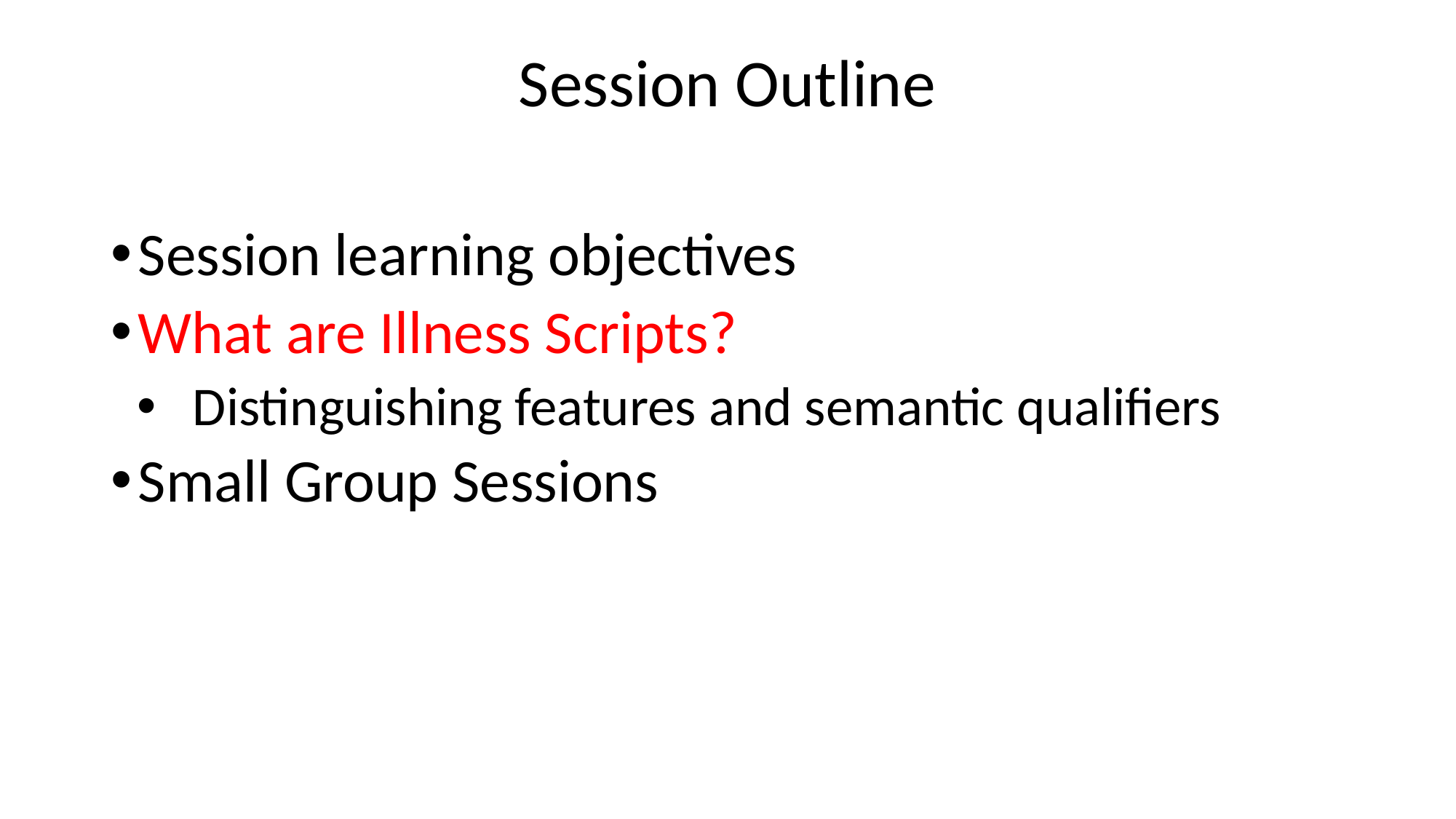

# Session Outline
Session learning objectives
What are Illness Scripts?
Distinguishing features and semantic qualifiers
Small Group Sessions

## Slide 6
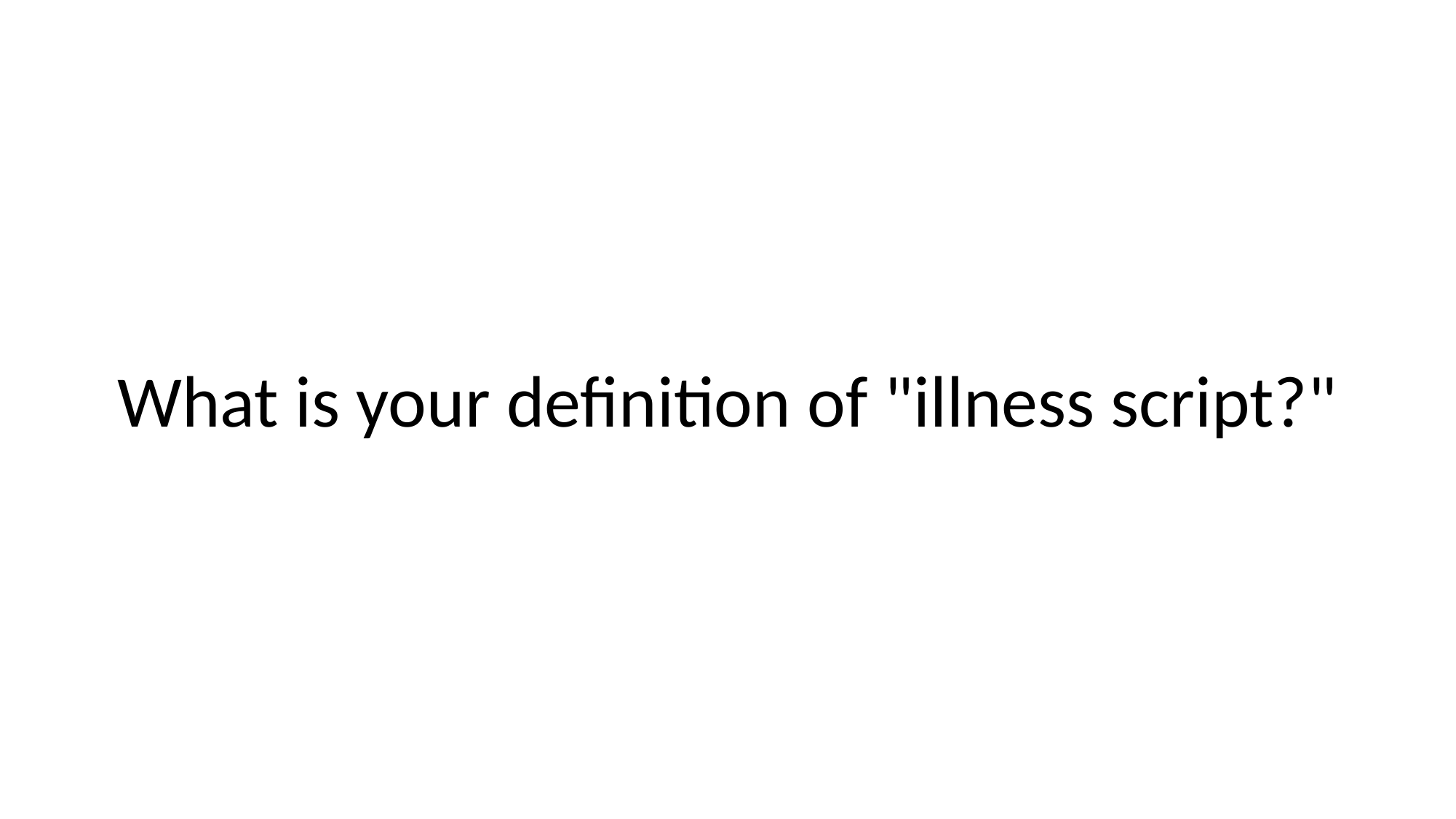

What is your definition of "illness script?"

## Slide 7
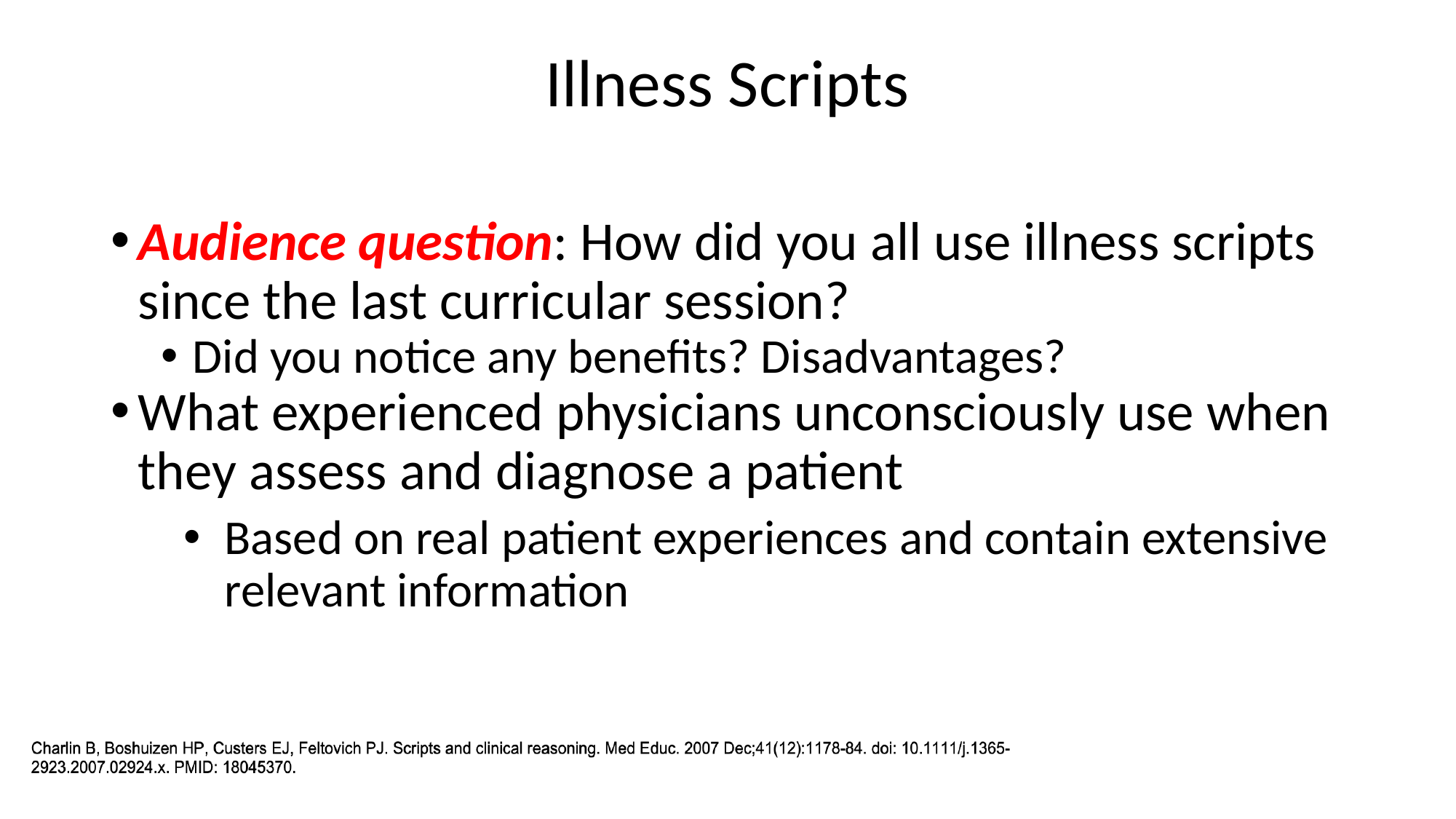

# Illness Scripts
Audience question: How did you all use illness scripts since the last curricular session?
Did you notice any benefits? Disadvantages?
What experienced physicians unconsciously use when they assess and diagnose a patient
Based on real patient experiences and contain extensive relevant information

## Slide 8
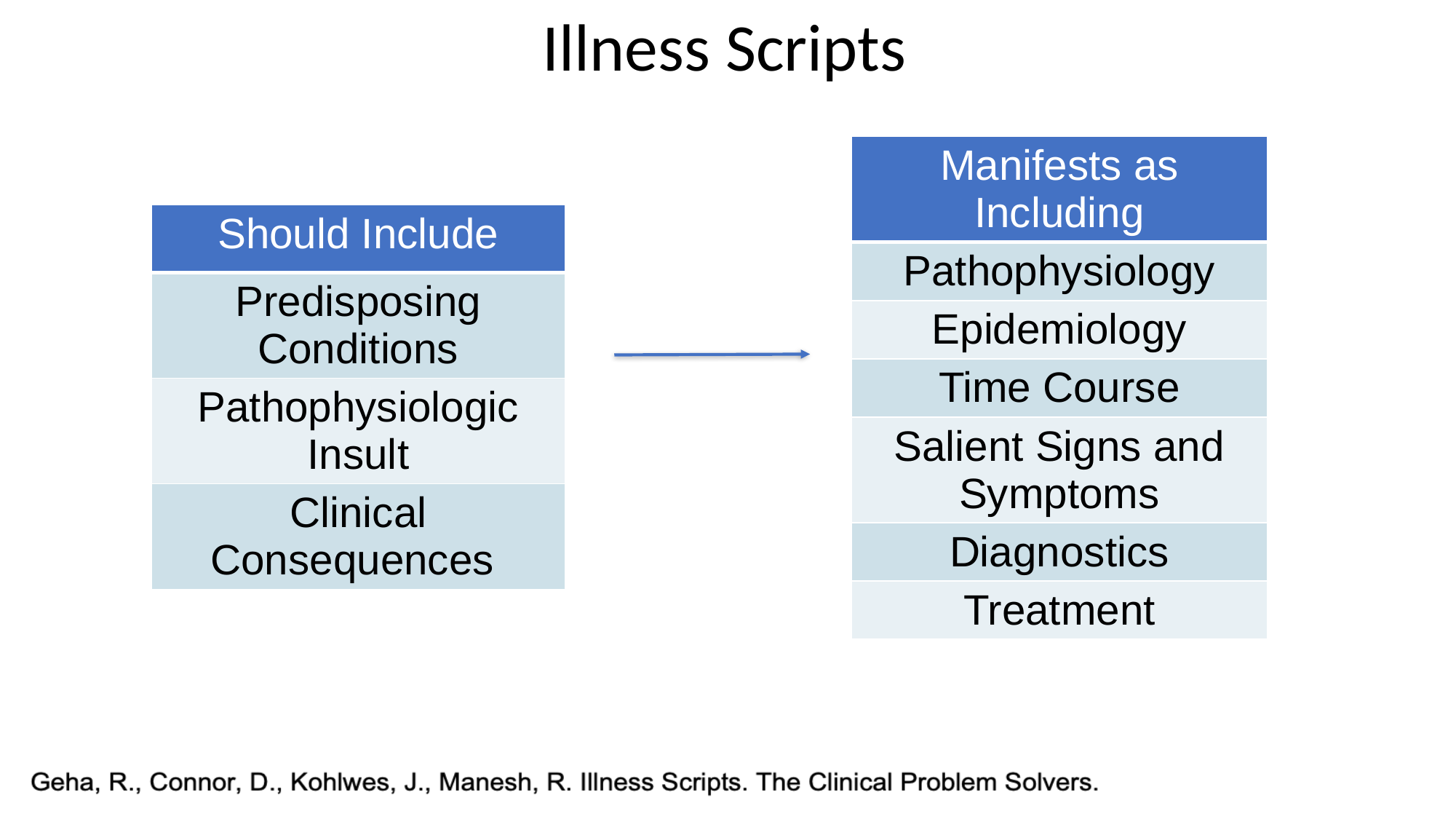

# Illness Scripts
| Manifests as Including |
| --- |
| Pathophysiology |
| Epidemiology |
| Time Course |
| Salient Signs and Symptoms |
| Diagnostics |
| Treatment |
| Should Include |
| --- |
| Predisposing Conditions |
| Pathophysiologic Insult |
| Clinical Consequences |

## Slide 9
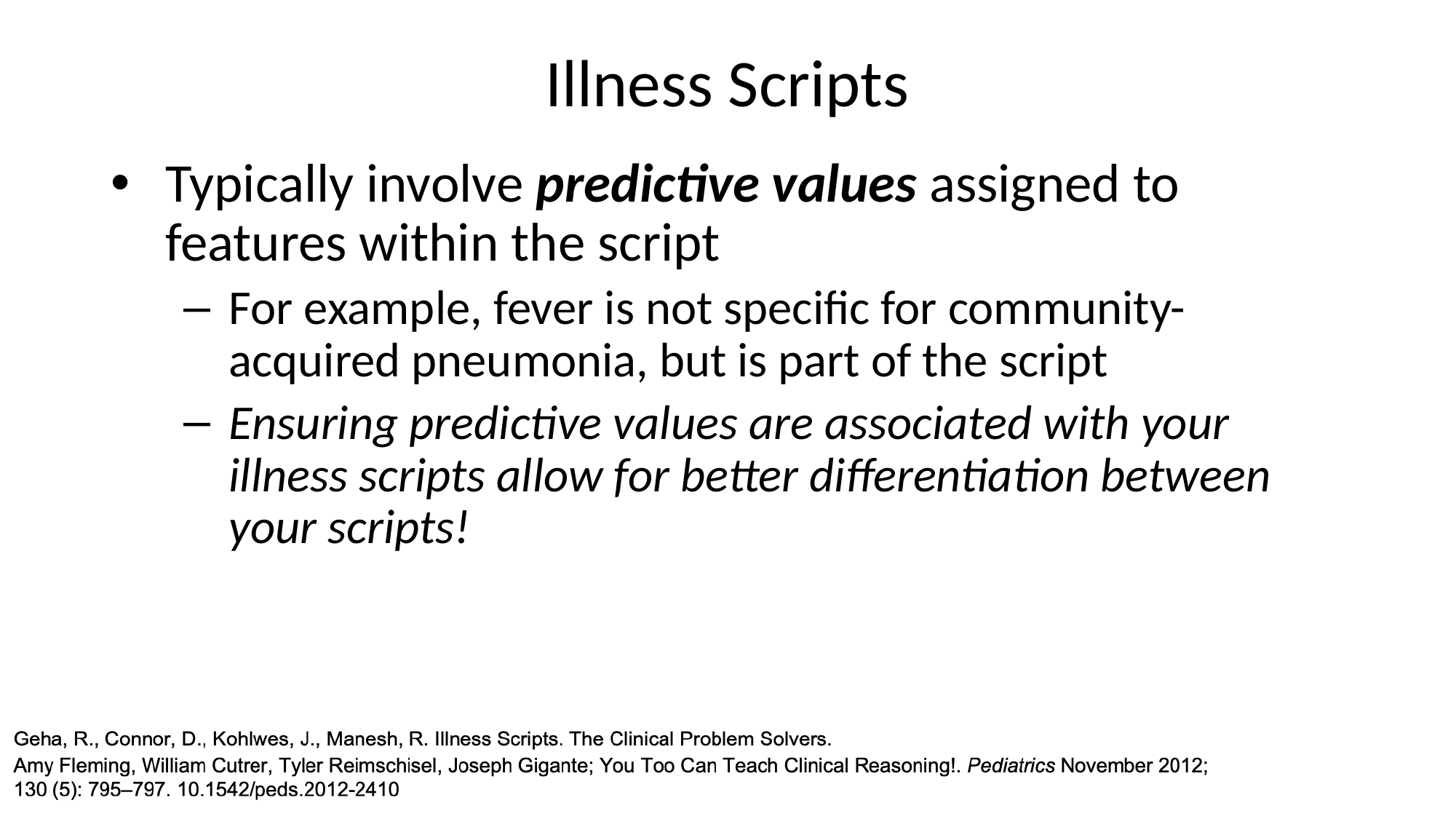

# Illness Scripts
Typically involve predictive values assigned to features within the script
For example, fever is not specific for community-acquired pneumonia, but is part of the script
Ensuring predictive values are associated with your illness scripts allow for better differentiation between your scripts!

## Slide 10
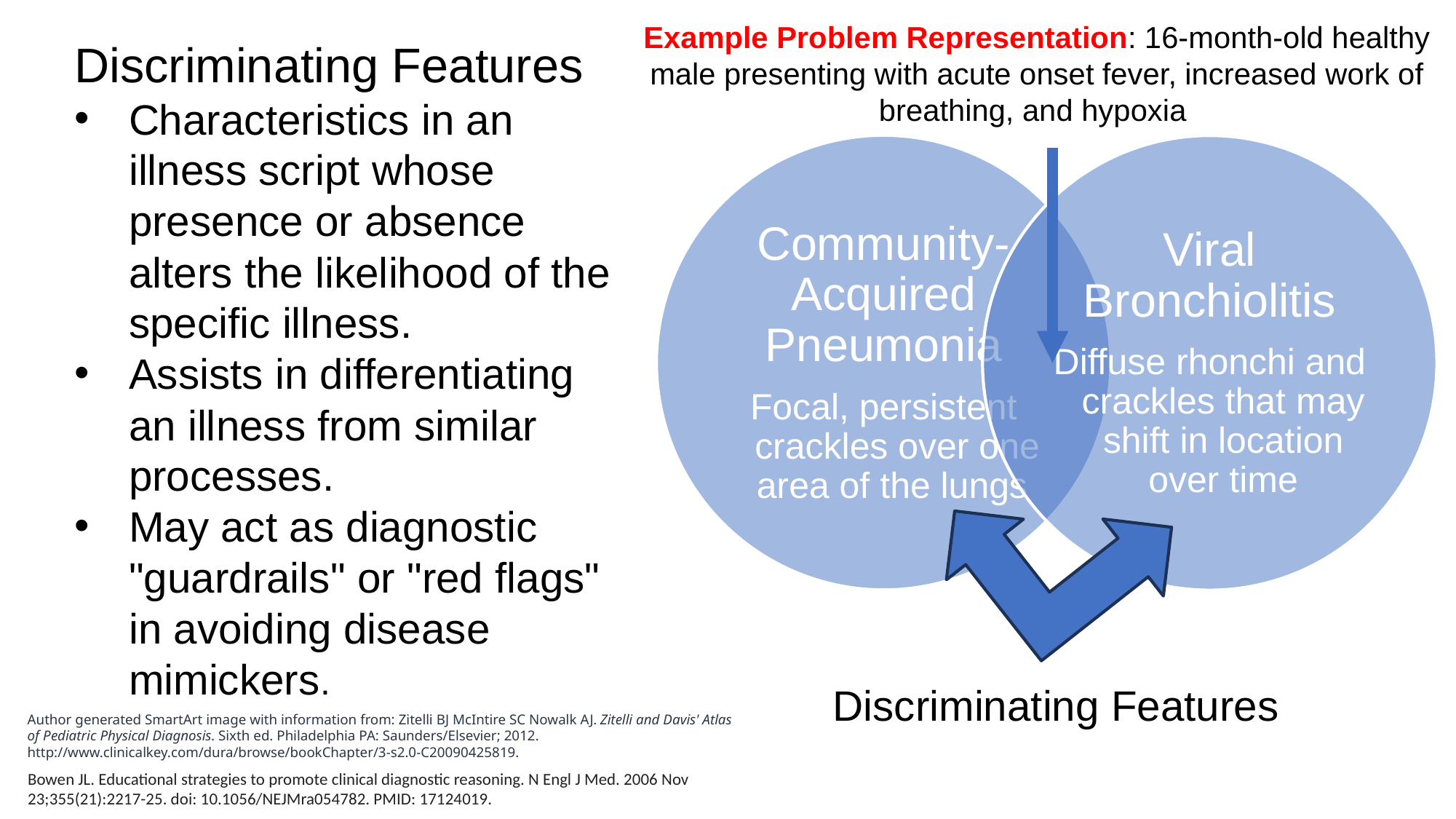

Example Problem Representation: 16-month-old healthy male presenting with acute onset fever, increased work of breathing, and hypoxia
Discriminating Features
Characteristics in an illness script whose presence or absence alters the likelihood of the specific illness.
Assists in differentiating an illness from similar processes.
May act as diagnostic "guardrails" or "red flags" in avoiding disease mimickers.
Discriminating Features
Author generated SmartArt image with information from: Zitelli BJ McIntire SC Nowalk AJ. Zitelli and Davis' Atlas of Pediatric Physical Diagnosis. Sixth ed. Philadelphia PA: Saunders/Elsevier; 2012. http://www.clinicalkey.com/dura/browse/bookChapter/3-s2.0-C20090425819.
Bowen JL. Educational strategies to promote clinical diagnostic reasoning. N Engl J Med. 2006 Nov 23;355(21):2217-25. doi: 10.1056/NEJMra054782. PMID: 17124019.

## Slide 11
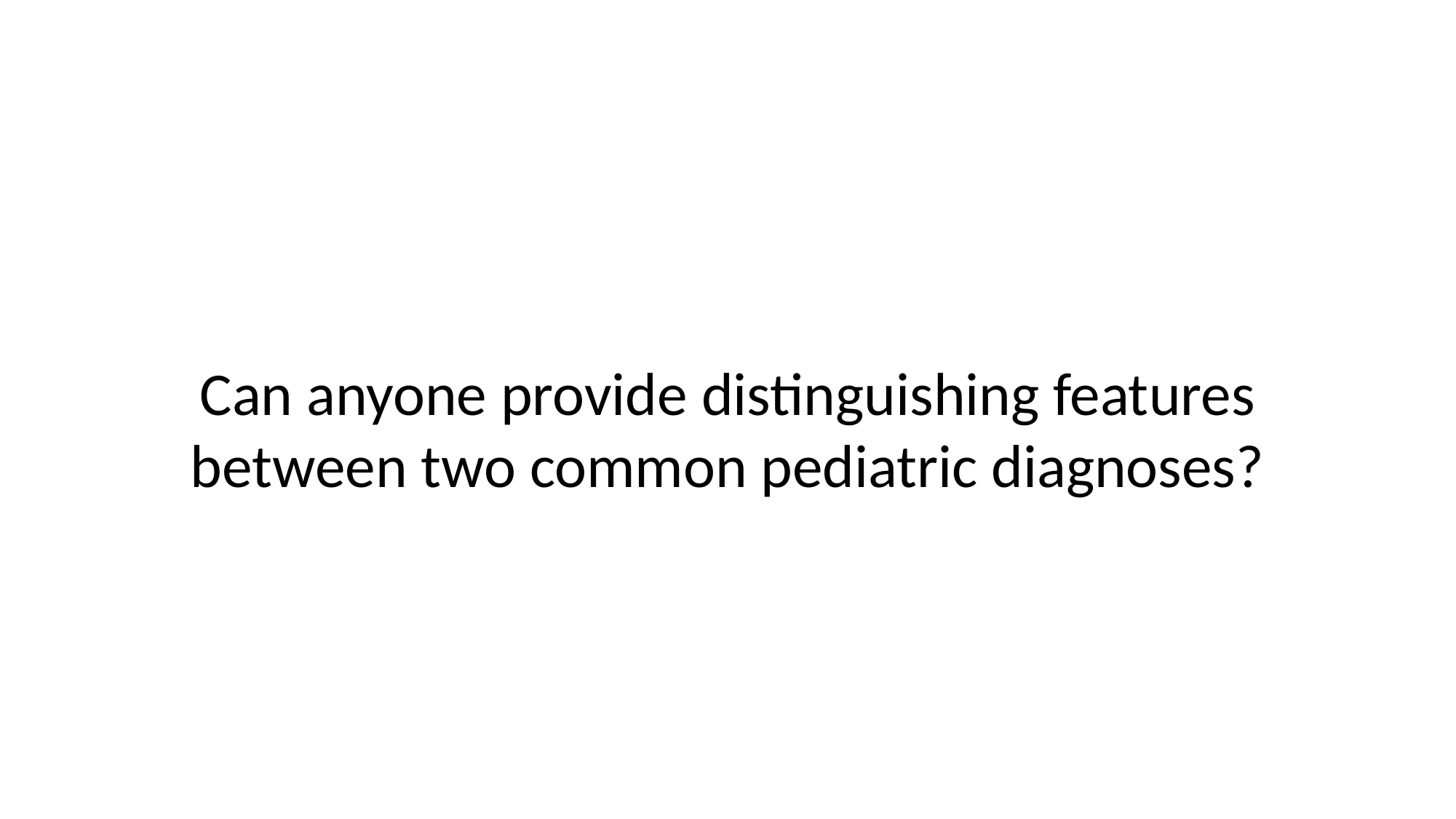

#
Can anyone provide distinguishing features between two common pediatric diagnoses?

## Slide 12
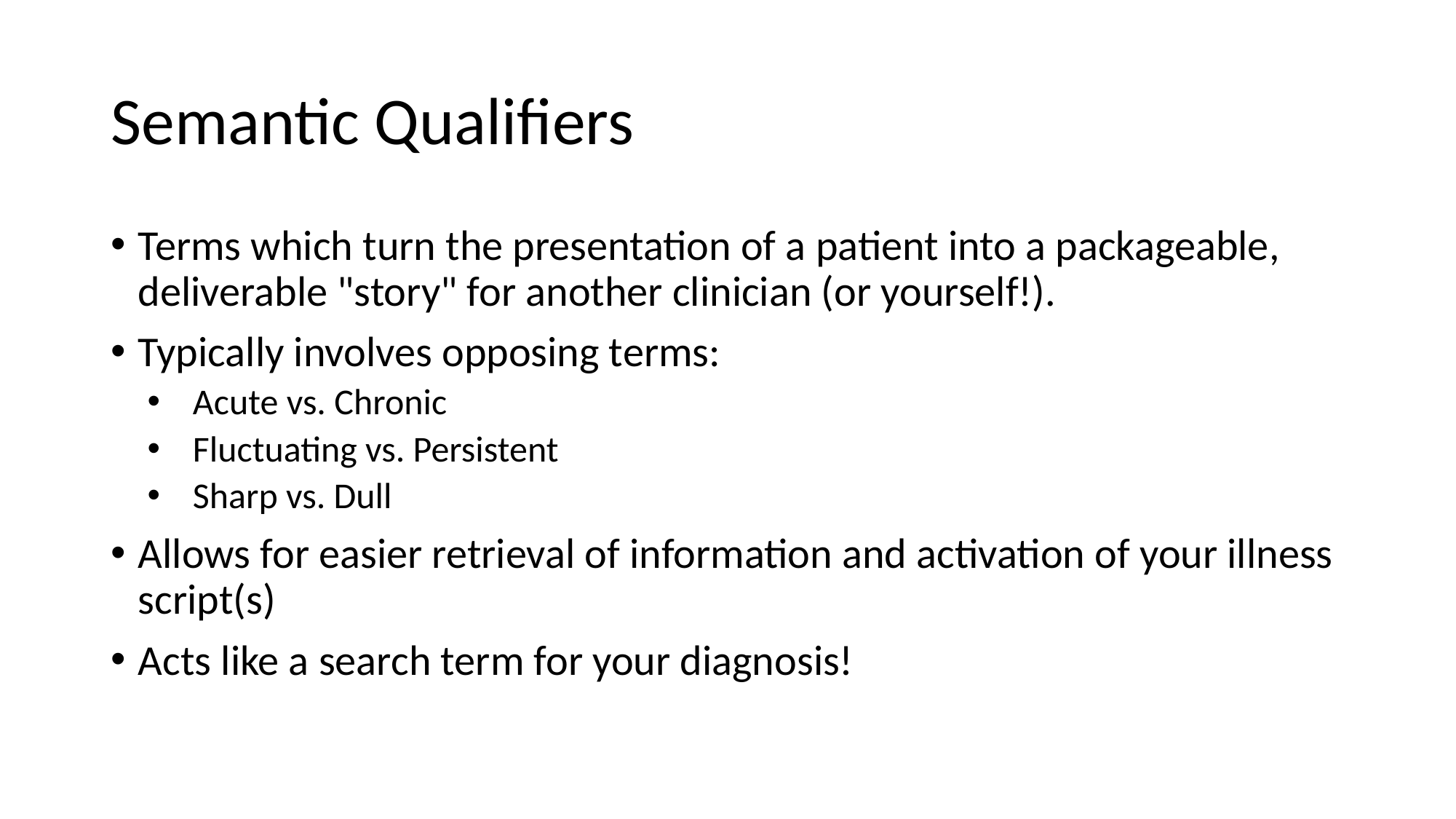

# Semantic Qualifiers
Terms which turn the presentation of a patient into a packageable, deliverable "story" for another clinician (or yourself!).
Typically involves opposing terms:
Acute vs. Chronic
Fluctuating vs. Persistent
Sharp vs. Dull
Allows for easier retrieval of information and activation of your illness script(s)
Acts like a search term for your diagnosis!

## Slide 13
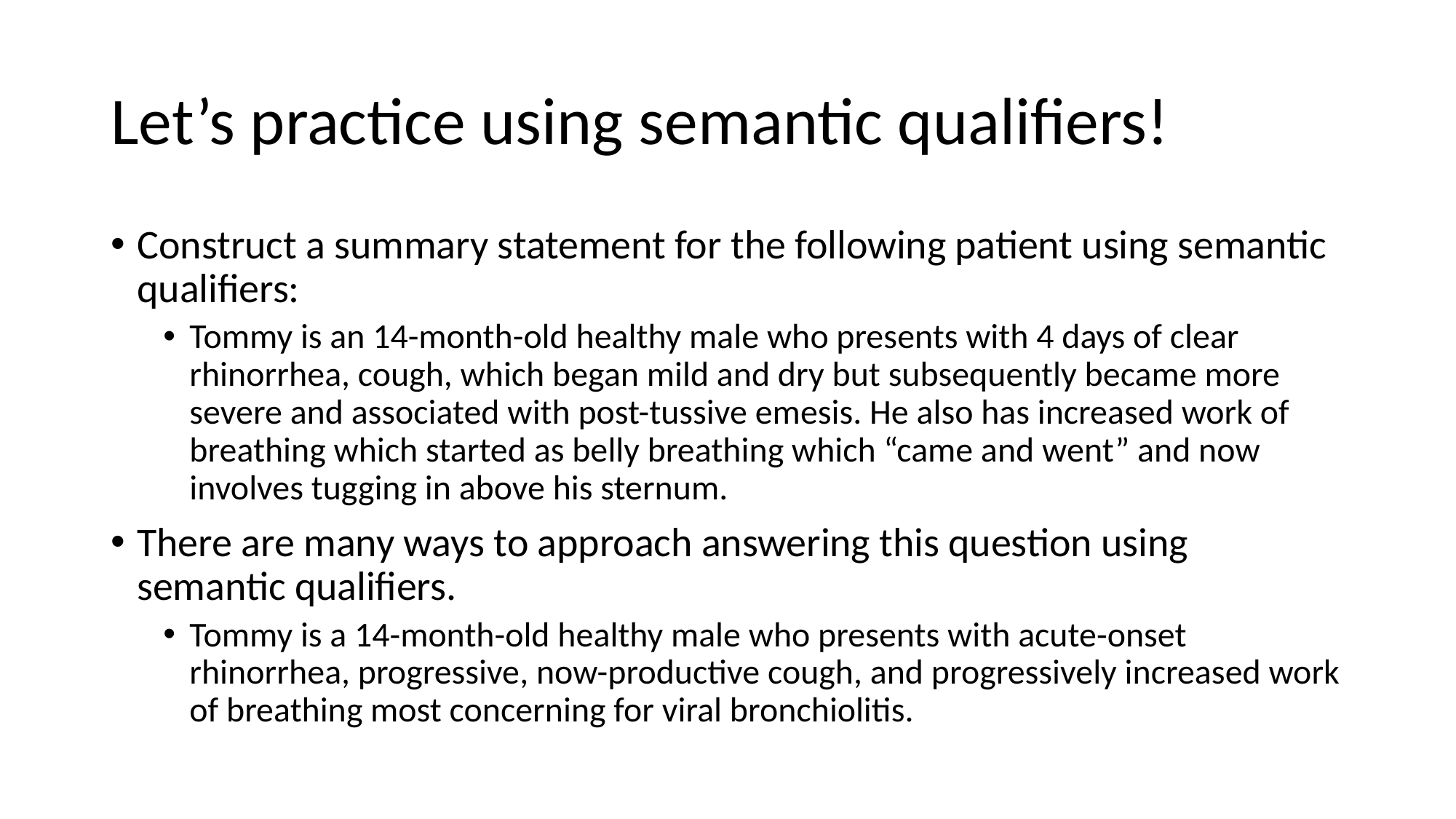

# Let’s practice using semantic qualifiers!
Construct a summary statement for the following patient using semantic qualifiers:
Tommy is an 14-month-old healthy male who presents with 4 days of clear rhinorrhea, cough, which began mild and dry but subsequently became more severe and associated with post-tussive emesis. He also has increased work of breathing which started as belly breathing which “came and went” and now involves tugging in above his sternum.
There are many ways to approach answering this question using semantic qualifiers.
Tommy is a 14-month-old healthy male who presents with acute-onset rhinorrhea, progressive, now-productive cough, and progressively increased work of breathing most concerning for viral bronchiolitis.

## Slide 14
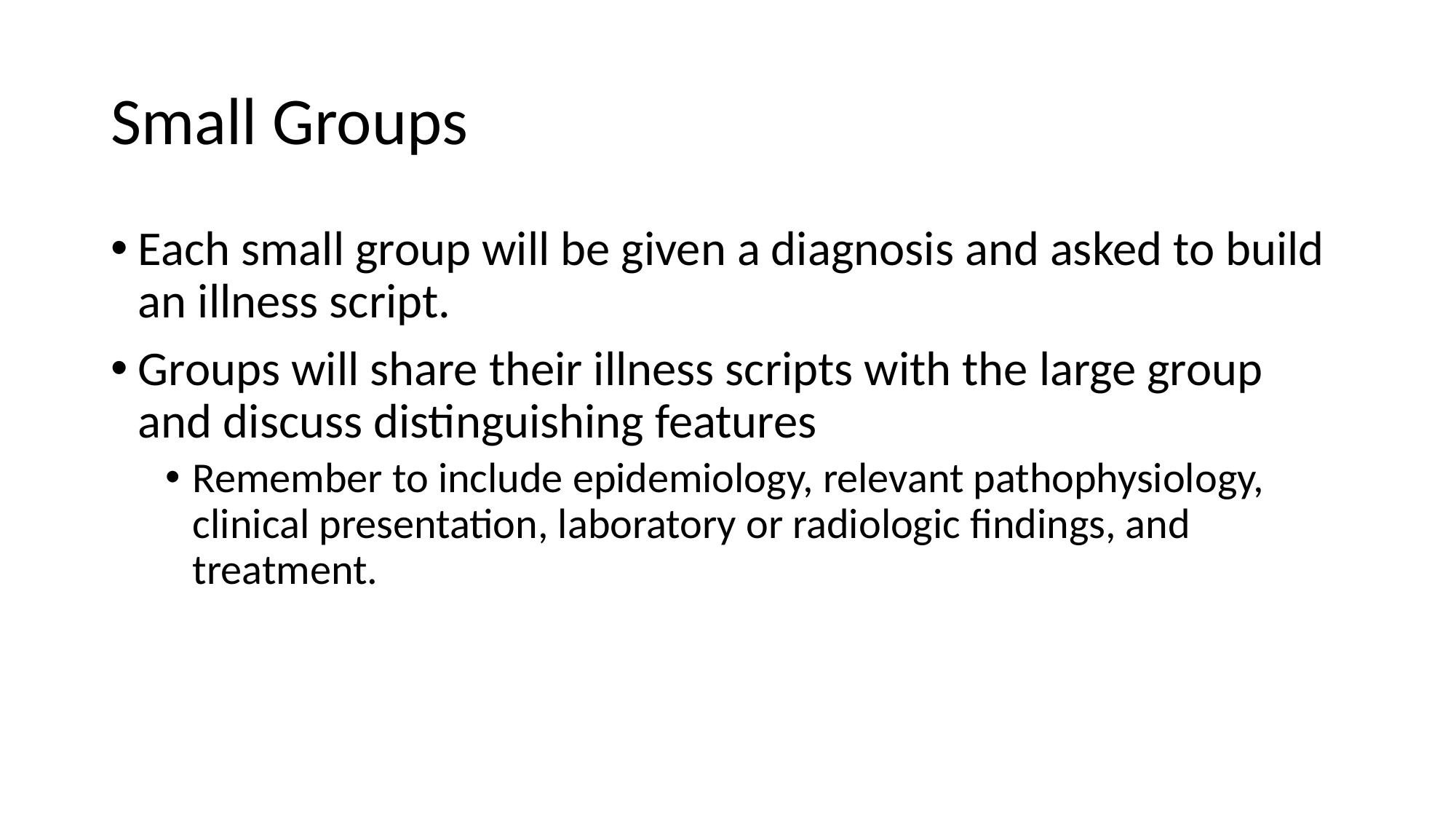

# Small Groups
Each small group will be given a diagnosis and asked to build an illness script.
Groups will share their illness scripts with the large group and discuss distinguishing features
Remember to include epidemiology, relevant pathophysiology, clinical presentation, laboratory or radiologic findings, and treatment.

## Slide 15
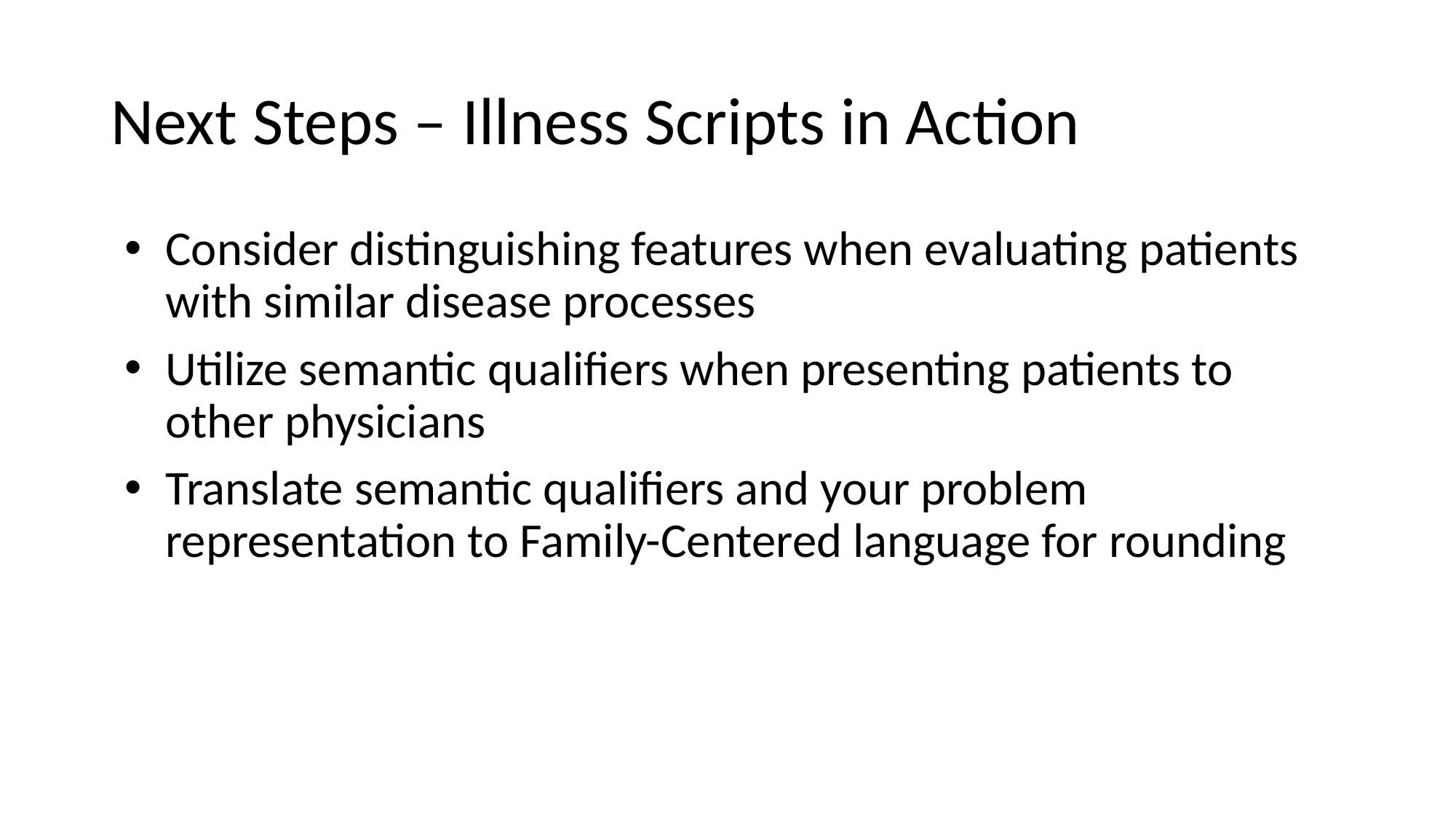

# Next Steps – Illness Scripts in Action
Consider distinguishing features when evaluating patients with similar disease processes
Utilize semantic qualifiers when presenting patients to other physicians
Translate semantic qualifiers and your problem representation to Family-Centered language for rounding

## Slide 16
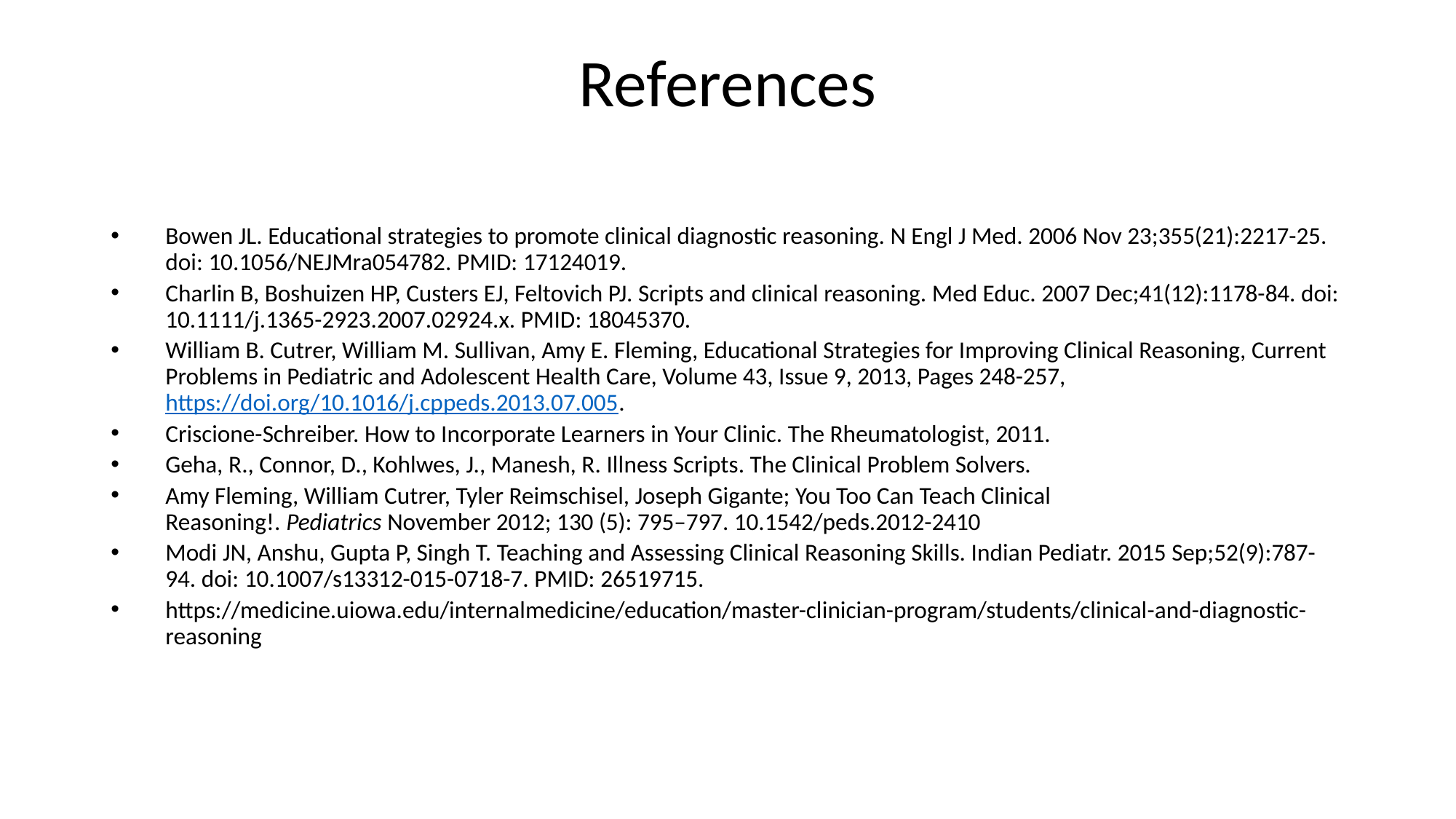

# References
Bowen JL. Educational strategies to promote clinical diagnostic reasoning. N Engl J Med. 2006 Nov 23;355(21):2217-25. doi: 10.1056/NEJMra054782. PMID: 17124019.
Charlin B, Boshuizen HP, Custers EJ, Feltovich PJ. Scripts and clinical reasoning. Med Educ. 2007 Dec;41(12):1178-84. doi: 10.1111/j.1365-2923.2007.02924.x. PMID: 18045370.
William B. Cutrer, William M. Sullivan, Amy E. Fleming, Educational Strategies for Improving Clinical Reasoning, Current Problems in Pediatric and Adolescent Health Care, Volume 43, Issue 9, 2013, Pages 248-257, https://doi.org/10.1016/j.cppeds.2013.07.005.
Criscione-Schreiber. How to Incorporate Learners in Your Clinic. The Rheumatologist, 2011.
Geha, R., Connor, D., Kohlwes, J., Manesh, R. Illness Scripts. The Clinical Problem Solvers.
Amy Fleming, William Cutrer, Tyler Reimschisel, Joseph Gigante; You Too Can Teach Clinical Reasoning!. Pediatrics November 2012; 130 (5): 795–797. 10.1542/peds.2012-2410
Modi JN, Anshu, Gupta P, Singh T. Teaching and Assessing Clinical Reasoning Skills. Indian Pediatr. 2015 Sep;52(9):787-94. doi: 10.1007/s13312-015-0718-7. PMID: 26519715.
https://medicine.uiowa.edu/internalmedicine/education/master-clinician-program/students/clinical-and-diagnostic-reasoning
