# Supplementary material for: A Pilot Longitudinal Clinical Reasoning Curriculum for Pediatric Residents
Source: MedEdPORTAL. 2024 Sep 25;20:11447. doi: 10.15766/mep_2374-8265.11447 (PMC11422513; doi:10.15766/mep_2374-8265.11447)
Supplement: Supplementary file 1 — Preimplementation Survey.docxCurriculum Goals, Objectives, and Timeline.docxSession 1 - Illness Scripts.pptxSession 1 - Small-Group Facilitator Guide.docxSession 2 - Illness Scripts 2.pptxSession 2 - Small-Group Facilitator Guide.docxSession 3 - Script Concordance.pptxSession 3 - Small-Group Facilitator Guide.docxSession 3 - Small-Group Handout.docxSession 4 - Pathophysiology.pptxSession 4 - Small-Group Facilitator Guide.docxSession 4 - Small-Group Handout.docxSession 5 - Review Game.pptxPostimplementation Survey.docx [file mep_2374-8265.11447-s001.zip › F. Session 2 - Small-Group Facilitator Guide.docx]

**Pediatric Advanced Clinical Reasoning Curriculum - Session #2**

**Facilitator Guide**

**Suggested timing for Session #2**

Didactic Material (Appendix E)

- Review of curriculum and introduction (Slides 1-5) - 4 minutes
- Review definition and use of illness scripts (Slides 6-10) - 6 minutes
- Define and discuss distinguishing features (Slides 11-12) - 4 minutes
- Define and discuss semantic qualifiers (Slides 13) - 4 minutes
- Small group explanation (Slide 14-16) - 2 minutes

Small Group Activities

- Activity 1 – 10 minutes
- Debrief activity 1 – 8 minutes
- Activity 2 – 10 minutes
- Debrief activity 2 – 12 minutes

**Goals for this session include:**

- Define the terms “semantic qualifier” and “distinguishing feature” as they relate to the construction and use of illness scripts.
- Implement reviewed clinical reasoning skills to build illness scripts for common and uncommon pediatric diagnoses.
- Evaluate distinguishing features in common pediatric clinical presentations.
- Identify differences in illness script structure/composition between common and uncommon diagnoses.

**Directions:**

- Trainees should be broken up into four small groups, each with a facilitator present.
- Each group should be assigned to build an illness script for one of the following conditions (Septic arthritis of the hip, Lyme arthritis, Transient Synovitis, or Juvenile Idiopathic Arthritis). Following completion of their illness script, they should present their illness script to the large group, highlighting discriminating features of epidemiology, pathophysiology, presentation, or management.
- Then, each small group should be tasked with building illness scripts for the 4 more rare conditions (Wilm’s Tumor, Biliary Atresia, Guillain-Barre Syndrome, and Thrombotic Thrombocytopenic Purpura).
  - Discuss with each group how building these illness scripts differed from the process of building scripts for more common conditions.
  - How would they compare an illness script for a condition such as TTP to one for a common condition such as Bronchiolitis?
    - Does the *use* of these different illness scripts differ?

**Other reflection questions for the large group:**

- What parts of the illness script would you add/subtract/modify if you wrote it for your group?
- What are some of the top differential diagnoses of your case and what discriminating features help distinguish between your case and the top differentials?
- What elements were important to *you* when you built your illness script? (*Note: this may vary physician-to-physician)*
- What were some of your challenges when building these illness scripts? Which ones were harder and why?

**SAMPLE ILLNESS SCRIPTS FOR FACILITATORS:**

**Features in these sample illness scripts can be used to guide groups who find themselves “stuck.” It is *not* the goal of this session to build a full, complete illness script, especially for more rare diagnoses. The goal is to include features from various domains of an illness script, including epidemiology, diagnostic workup, symptoms, etc. If groups do not spontaneously discuss items from different domains, facilitators may prompt groups to discuss them.**

**Septic Arthritis of the Hip:**

A rare but serious disorder commonly affecting younger children. Hip and knee are most likely to be involved. The mechanism may be due to direct inoculation of the joint (trauma, surgery, etc.) or hematogenous seeding of bacteremia. Additionally, it may involve extension into the joint from adjacent bone (osteomyelitis). There are many types of bacteria which may cause septic arthritis, and the microbiology changes depending on age of presentation. However, the classic presentation involves joint swelling, tenderness, and limited range of motion of the affected joint. The Kocher Criteria can be used for differentiating septic arthritis of the hip from transient synovitis to evaluate risk/benefit of performing a synovial fluid aspiration. One would look for fever, failure to bear weight, elevated WBC count, and elevated inflammatory markers. A discriminating feature of septic arthritis management is prompt initiation of antimicrobials – this is juxtaposed to other infectious etiologies such as osteomyelitis where antibiotics can be held until a sample is obtained if there is clinical stability. Synovial fluid will often show elevated WBC count and may show bacteria on gram stain.

**Lyme Arthritis**:

A disease process typically with a geographic distribution (Northeast, Midwest, and Western United States) due to zoonotic transmission from the *Ixodes* tick. Lyme arthritis is a late manifestation of Lyme Disease, typically presenting months after a tick bite (in untreated patients). The classic presentation involves a **subacute** monoarticular arthritis of the knee with swelling disproportionate to tenderness. May also feature fever, headache, myalgia, arthralgia, and fatigue. Patients usually, but not always, maintain the ability to bear weight (unlike in septic arthritis. They also have pain with range of motion. Laboratory evaluation typically shows normal to mildly elevated WBC, elevated ESR, and CRP. Joint aspiration may show elevated WBC, but typically less so than in bacterial septic arthritis.

**Transient Synovitis**:

Commonly seen in children aged 3-10 years old. Etiology proposed to be from preceding URI or bacterial (streptococcal) infection. The classic presentation includes children with recent or ongoing Upper Respiratory infection with subacute onset of arthralgia symptoms. May be “walking funny” or failing to bear weight. Examination should show unilateral hip pain with mild restriction of range of motion. May also see fever and mild elevation in inflammatory markers. Kocher Criteria can help differentiate between Transient Synovitis of the hip and septic arthritis. Symptoms should improve dramatically with NSAIDS, and typically improve within 24-48 hours of onset.

**Juvenile Idiopathic Arthritis**:

Onset is often early (before adolescent years). The most common phenotype (classic presentation) is Oligoarticular, and knees are the most common joints involved. The etiology is chronic and autoimmune/inflammatory. The diagnosis is sometimes difficult given it is a diagnosis of exclusion. May also see systemic symptoms such as prolonged, quotidian fever +/- an evanescent, salmon-colored rash. ANA can be positive and will often see elevated inflammatory markers.

**PART 2**

**Wilm’s Tumor**

Wilm’s Tumor (Nephroblastoma) commonly presents in children under 5 years old. It can be associated with genetic syndromes which have other features (e.g., genitourinary abnormalities, macrosomia, macroglossia, hemihypertrophy, aniridia, etc.). The classic presentation is with a subacute or chronic abdominal mass or swelling. Associated symptoms include abdominal pain, fever, hypertension, and hematuria (which may include clots). Physical examination reveals a firm, nontender, smooth mass that rarely crosses the midline. “Red Flag”' physical exam findings include rapidly enlarging abdominal girth or significant abdominal pain, which may indicate subcapsular hemorrhage. Imaging via ultrasonography may reveal hydronephrosis or medical renal disease, whereas CT or MRI may delineate the mass. Associated diagnostics should include chest imaging to examine for lung metastasis.

**Biliary Atresia**

Epidemiology includes neonates, with presenting symptoms often occurring in the first few weeks of life. The etiology of biliary atresia is unclear but may be related to genetic mutations or prenatal exposures (e.g., viruses, toxins). Clinical Presentation: Prenatal findings are often normal, whereas the first notable symptoms are typically acute or subacute jaundice, acholic stools, dark urine, and potentially hepatosplenomegaly (w/ chronic symptoms). Evaluation should include ultrasonography, which may show evidence of cholestasis, absence of the gallbladder, irregularly shaped gallbladder, etc. Liver biopsy should show histologic changes of obstruction and may differentiate between other causes of cholestasis (Alagille, A1AT deficiency, PFIC3, etc.). Gold standard of diagnosis of intraoperative cholangiogram, and Kasai Procedure is the gold standard treatment.

**Thrombotic Thrombocytopenic Purpura:**

A rare disorder (1/1,000,000) more commonly seen in older children and adolescents. Can be congenital but more commonly is acquired due to a deficiency in ADAMTS13 – decreased levels of ADAMTS13 causes accumulation of von Willebrand factor multimers, leading to thrombi in arterioles and capillaries -> microvascular ischemia. The classic presentation involves a pentad – fever, microangiopathic hemolytic anemia (MAHA), thrombocytopenia, neurologic symptoms, and renal dysfunction, although this is not common in every presentation. Symptoms include fatigue, dyspnea, bleeding, petechiae, dizziness, headaches, confusion, seizures, weakness, abdominal pain, pallor, and jaundice. Evaluation -- Complete Blood Count (showing hemolytic anemia, thrombocytopenia, and schistocytes), elevated LDH (Lactate dehydrogenase), and a BMP (elevated creatinine). ADAMTS13 level will be low. Management involves plasma exchange, which is a discriminating feature from a similar disorder (Hemolytic Uremic Syndrome) in which the management is supportive.

**Guillain Barre:**

A rare disorder likely occurring due to a preceding infection evoking an autoimmune response -> antibody production that is targeted at antigenic proteins on peripheral nerves.

Most common preceding infection association being campylobacter (others include: EBV, CMV, mycoplasma pneumoniae), most commonly 2-4 weeks after. There is also a slightly increased risk of GBS after influenza vaccination. Clinical presentation includes systemic symptoms (fatigue, weakness, pain). Neurological symptoms include paresthesia, weakness, ataxia, and pain. Weakness is typically SYMMETRICAL and ASCENDING – this is a discriminating feature of GBS and is concerning given potential for respiratory muscle involvement. Diagnosis: Lumbar puncture showing high protein (>45) with NORMAL CSF WBCs (albuminocytologic dissociation), contrast enhancement of spinal nerve roots/cauda equina on MRI, antiganglioside Ab in about 50%. Management includes intravenous immunoglobulin (IVIG) and close monitoring of respiratory status as up to 20% may require ventilation support.
