# Supplementary material for: A Pilot Longitudinal Clinical Reasoning Curriculum for Pediatric Residents
Source: MedEdPORTAL. 2024 Sep 25;20:11447. doi: 10.15766/mep_2374-8265.11447 (PMC11422513; doi:10.15766/mep_2374-8265.11447)
Supplement: Supplementary file 1 — Preimplementation Survey.docxCurriculum Goals, Objectives, and Timeline.docxSession 1 - Illness Scripts.pptxSession 1 - Small-Group Facilitator Guide.docxSession 2 - Illness Scripts 2.pptxSession 2 - Small-Group Facilitator Guide.docxSession 3 - Script Concordance.pptxSession 3 - Small-Group Facilitator Guide.docxSession 3 - Small-Group Handout.docxSession 4 - Pathophysiology.pptxSession 4 - Small-Group Facilitator Guide.docxSession 4 - Small-Group Handout.docxSession 5 - Review Game.pptxPostimplementation Survey.docx [file mep_2374-8265.11447-s001.zip › H. Session 3 - Small-Group Facilitator Guide.docx]

**Pediatric Advanced Clinical Reasoning Curriculum**

**Session 3 Facilitator Guide**

**Suggested timing for Session #3**

**Didactic Material (Appendix G)**

- Review curriculum and learning objectives (Slides 1-8) - 3 minutes
- Review illness scripts (Slides 9-12) - 8 minutes
- Define and provide examples of script concordance (Slides 13-17) - 9 minutes

**Small Group Activities**

- Explanation of activities (Slide 18-19) - 1 minute
- Activities **(Small Group Handout Appendix I) - 29 minutes**
- Debrief session - 10 minutes

**Structure**: provide the participants with a “one liner” patient presentation and ask them to write a differential. Key details (“statements” below) should be provided one-at-a-time. After each statement is provided, ask them to decide if it is concordant with diagnoses on their differential (some statements may be concordant with all, some, or none of the diagnoses they provide).

After all statements are provided, the facilitator should ask the discussion questions at the end of the document to prompt discussion about different participant perspectives on their individual differential diagnoses (DDX).

This activity can be adapted based on different sizes or structures of small groups. Small groups can first produce a differential diagnosis all together *or* individuals can create their own differential diagnoses and discuss them at the end.

**#1—A 4-year-old girl presents to the clinic with a history of fever and a sore throat for the past 3 days.**

- Example DDX: *strep throat, retropharyngeal abscess (RPA), viral pharyngitis, diphtheria*
- Statement #1: She has had high fevers over the past 2 days.
  - Strep throat, RPA
- Statement #2: She has had low grade fevers over the past 2 days.
  - Viral pharyngitis, diphtheria
- Statement #3: Palpable lymph nodes on neck
  - DDX: Strep throat, viral pharyngitis, RPA
- Statement #4: She developed a full body rash preceding the sore throat
  - DDX: Strep throat, viral pharyngitis
- Statement #5: She is incomplete on her immunizations.
  - DDX: Diphtheria
- Statement #6: On exam, she has tonsillar hypertrophy and erythema with a coating on them.
  - DDX: strep pharyngitis (white), diphtheria (gray)

**#2—A 14-year-old male presents to the emergency department after passing out at school earlier today.**

- Example DDX: *vasovagal syncope, cardiogenic syncope, non-epileptiform event, ingestion, seizure, narcolepsy*
- Statement #1: The patient recalls feeling blurred vision and diaphoretic prior to the event.
  - DDX: vasovagal syncope, ingestion
- Statement #2: The patient has had some of these similar spells before.
  - DDX: Vasovagal syncope, NEE, seizure, narcolepsy
- Statement #3: He appears “out of it” when arriving to the emergency room-
  - DDX: ingestion, seizure (post ictal state)
- Statement #4: He was playing soccer in gym class and passed out mid play on the field.
  - DDX: Cardiogenic syncope
- Statement #5: Classmates around him witnessed abnormal movements.
  - DDX: NEE, seizure, vasovagal syncope, ingestion (serotonin syndrome)
- Statement #6: Patient endorsed nausea at school and had episode of emesis shortly after.
  - DDX: ingestion, vasovagal

**#3—A 6-month-old former full term baby girl presents with lethargy**

- *Example DDX: sepsis (meningitis), dehydration, non-accidental trauma (NAT), hypoglycemia, intussusception, ingestion, status epilepticus, SVT, sinus venous thrombosis*
- Statement #1: She was in her usual state of health until this morning and suddenly acted differently after morning nap.
  - DDX: NAT, ingestions, status
- Statement #2: She had multiple episodes of vomiting over the last day with decreased feeding.
  - DDX: dehydration, hypoglycemia, NAT, intussusception
- Statement #3: On exam, she is unable to arouse and with an O2 saturation of 88%
  - DDX: sepsis, status epilepticus, ingestion, NAT
- Statement #4: Parents report blood in her stools over the past 2 days
  - DDX: intussusception, NAT, sepsis
- Statement #5: She is tachycardic on exam with weak femoral pulses and Systolic Blood Pressure of 60mmHg
  - DDX: sepsis, NAT, dehydration, prolonged SVT,
- Statement #6: She has abnormal rhythmic shaking of her 4 extremities
  - DDX: Sepsis, status, NAT (intracranial hemorrhage +seizures), sinus venous thrombosis

**#4—A 15-year-old male with autism presents with abdominal pain**

- Example DDX: *gastroenteritis, inflammatory bowel disease, constipation, pancreatitis, ulcer, obstruction, appendicitis, testicular torsion, lead poisoning*
- Statement #1: He has multiple episodes of emesis a day.
  - DDX: torsion, pancreatitis, obstruction
- Statement #2: Has not had a bowel movement in 1 week
  - DDX: Obstruction, constipation
- Statement #3: He has epigastric tenderness on examination.
  - DDX: pancreatitis, ulcer
- Statement #4: He endorses decreased appetite.
  - DDX: IBD, gastroenteritis, appendicitis
- Statement #5: Pain is described as colicky in nature.
  - DDX: mesenteric adenitis, gastroenteritis
- Statement #6: He has a history of anemia
  - DDX: obstruction (bezoar w/ pica vibes), lead poisoning, IBD

**#5—A 4-year-old girl presents with walk refusal for a 2-day duration**

- *Example DDX: transient synovitis, toddler’s fracture, leukemia, septic arthritis, osteomyelitis, NAT, myositis, Lyme arthritis*
- Statement #1: She fell during her intense soccer game and has distal tibial tenderness
  - DDX: Toddler’s fracture
- Statement #2: She had a recent cold with congestion and fevers two days ago and is unable to point out where her pain is on exam.
  - DDX: transient synovitis, viral myositis
- Statement #3: She points to both of her legs when asked where the pain is. No source of swelling and no findings on examination aside from diffuse tenderness to palpation of her legs.
  - DDX: myositis, leukemia
- Statement #4: She has had fevers with a maximum temperature of 102F measured at home.
  - DDX: leukemia, septic arthritis, osteomyelitis
- Statement #5: Her knee is diffusely edematous with limited range of motion
  - DDX: septic arthritis, Lyme arthritis
- Statement #6: She has diffuse purpura on examination.
  - DDX: NAT, leukemia

**Questions for small groups:**

1. Can you think of other clarifying statements that would a) be common to multiple of your differential diagnoses and b) be unique to one of your differential diagnoses?
2. How can illness scripts and script concordance help you when you are writing your impression in documentation and/or presenting the patient?
3. What are limitations to illness scripts?
4. What are limitations to script concordance building as you are evaluating a patient?
5. Are illness scripts limited to diagnoses that you have seen before? How can you adapt the skill of illness scripts to new diagnoses?
