# Supplementary material for: A Pilot Longitudinal Clinical Reasoning Curriculum for Pediatric Residents
Source: MedEdPORTAL. 2024 Sep 25;20:11447. doi: 10.15766/mep_2374-8265.11447 (PMC11422513; doi:10.15766/mep_2374-8265.11447)
Supplement: Supplementary file 1 — Preimplementation Survey.docxCurriculum Goals, Objectives, and Timeline.docxSession 1 - Illness Scripts.pptxSession 1 - Small-Group Facilitator Guide.docxSession 2 - Illness Scripts 2.pptxSession 2 - Small-Group Facilitator Guide.docxSession 3 - Script Concordance.pptxSession 3 - Small-Group Facilitator Guide.docxSession 3 - Small-Group Handout.docxSession 4 - Pathophysiology.pptxSession 4 - Small-Group Facilitator Guide.docxSession 4 - Small-Group Handout.docxSession 5 - Review Game.pptxPostimplementation Survey.docx [file mep_2374-8265.11447-s001.zip › I. Session 3 - Small-Group Handout.docx]

**Pediatric Advanced Clinical Reasoning Curriculum**

**Session 3 Handout - Script Concordance**

**Directions**:

The facilitator will provide a “one-liner” patient presentation. Based on the one-liner, write down potential diagnoses that fit with the presentation at the top of each column in the table.

The facilitator will provide statements of additional information in each presentation. The statements are meant to be *independent* (e.g., they are happening in isolated patient encounters with the same one-liner). For each statement, rate each diagnosis you provided as more or less likely. The scale of -2 (ruled out),, -1 (less likely), 0 (neutral), +1 (more likely), or +2 (a confirmatory feature or much more likely) may be used.

***Example:***

18-month old previously healthy male presenting with increased work of breathing.

| Statements | Differential Diagnoses | | | |
| --- | --- | --- | --- | --- |
|  | Bronchiolitis | Foreign Body Aspiration | Reactive Airway Disease | Viral Croup |
| The symptoms began abruptly after the patient was playing alone in another room. | 0 | +1 | 0 | 0 |
| There is inspiratory stridor | 0 | +2 | -1 | +2 |
| There is musical, polyphonic expiratory wheezing | +1 | -1 | +1 | 0 |
| Symptoms improve dramatically after albuterol administration | 0 | -1 | +2 | -1 |

| Statements | Differential Diagnoses | | | |
| --- | --- | --- | --- | --- |
|  |  |  |  |  |
|  |  |  |  |  |
|  |  |  |  |  |
|  |  |  |  |  |
|  |  |  |  |  |
|  |  |  |  |  |
|  |  |  |  |  |

| Statements | Differential Diagnoses | | | |
| --- | --- | --- | --- | --- |
|  |  |  |  |  |
|  |  |  |  |  |
|  |  |  |  |  |
|  |  |  |  |  |
|  |  |  |  |  |
|  |  |  |  |  |
|  |  |  |  |  |

| Statements | Differential Diagnoses | | | |
| --- | --- | --- | --- | --- |
|  |  |  |  |  |
|  |  |  |  |  |
|  |  |  |  |  |
|  |  |  |  |  |
|  |  |  |  |  |
|  |  |  |  |  |
|  |  |  |  |  |

| Statements | Differential Diagnoses | | | |
| --- | --- | --- | --- | --- |
|  |  |  |  |  |
|  |  |  |  |  |
|  |  |  |  |  |
|  |  |  |  |  |
|  |  |  |  |  |
|  |  |  |  |  |
|  |  |  |  |  |

| Statements | Differential Diagnoses | | | |
| --- | --- | --- | --- | --- |
|  |  |  |  |  |
|  |  |  |  |  |
|  |  |  |  |  |
|  |  |  |  |  |
|  |  |  |  |  |
|  |  |  |  |  |
|  |  |  |  |  |
