# Supplementary material for: A Pilot Longitudinal Clinical Reasoning Curriculum for Pediatric Residents
Source: MedEdPORTAL. 2024 Sep 25;20:11447. doi: 10.15766/mep_2374-8265.11447 (PMC11422513; doi:10.15766/mep_2374-8265.11447)
Supplement: Supplementary file 1 — Preimplementation Survey.docxCurriculum Goals, Objectives, and Timeline.docxSession 1 - Illness Scripts.pptxSession 1 - Small-Group Facilitator Guide.docxSession 2 - Illness Scripts 2.pptxSession 2 - Small-Group Facilitator Guide.docxSession 3 - Script Concordance.pptxSession 3 - Small-Group Facilitator Guide.docxSession 3 - Small-Group Handout.docxSession 4 - Pathophysiology.pptxSession 4 - Small-Group Facilitator Guide.docxSession 4 - Small-Group Handout.docxSession 5 - Review Game.pptxPostimplementation Survey.docx [file mep_2374-8265.11447-s001.zip › K. Session 4 - Small-Group Facilitator Guide.docx]

Clinical Reasoning Curriculum: Session #4 – Pathophysiology

Facilitator’s Guide

Activity 1 – Acidosis

Suggested timing for Session #4

**Didactic Material (Appendix J)**

- Review of previous sessions (Slides 1-5) - 3 minutes
- Pathophysiology and clinical reasoning (Slides 6-15) - 10 minutes
- Examples of pathophysiology in clinical context (Slides 16-22) - 7 minutes

**Small Group Activities**

- Explanation of activities (Slide 23-24) - 1 minute
- Small group activities **(Small Group Handout Appendix L) - 39 minutes**
- Debrief session – 10 minutes

**Directions**: Evaluate the Basic Metabolic Panels below and match the panel with a suspected diagnosis shown on the right. After you match the BMPs, split up the 5 BMPs among your group and have each member explain electrolyte derangements and discuss any specific management steps that may resolve the abnormalities.

**Clinical Scenario: 2-year-old previously healthy male presenting with altered mental status**

**DIAGNOSES**:

1. ____ 1) Addison’s Disease

131 110 20 (5) Uremia



5.4 14 0.8 64 2) Diabetic Ketoacidosis

Serum Osmolality (Osm): 296 (4) Methanol Intoxication

(3) Acute Diarrhea

2. _________

129 102 7



4.9 7 0.8 546

Serum Osm: 297

3. __________

153 125 29



2.7 16 0.8 81

Serum Osm: 318

4. __________

139 107 15



3.2 14 0.5 97

Serum Osm: 303

5. __________

131 100 74



6.4 14 3.7 120

Serum Osm: 295

Explanations:

In **Addison’s Disease (Primary Adrenal Insufficiency**), there is a lack of glucocorticoid and mineralocorticoid effects – primary treatment is with fluid resuscitation and dextrose for adrenal crises, while hydrocortisone replaces the endogenous glucocorticoids for long-term management.

In **Uremia**, there is an increase in circulating blood urea nitrogen due to renal failure. Failing kidneys do not effectively retain bicarbonate or sodium, contributing to a non-anion gap metabolic acidosis. Also, hyperkalemia occurs due to failure to secrete potassium (and extracellular shifts of potassium in the setting of acidosis). Dialysis is often required in this scenario to correct the electrolyte derangements, while acute management should also be focused on preventing cardiac sequelae of hyperkalemia.

In **Diabetic Ketoacidosis (DKA)**, there is an absolute insulin deficiency, causing increased ketosis, leading to an anion gap metabolic acidosis with the presence of keto-acids. Hyperglycemia contributes to pseudohyponatremia. Hyperkalemia often occurs due to extracellular shifts of potassium in the setting of profound acidosis. Management steps include insulin replacement (to stop the formation of ketones, decrease glucose, and drive intracellular potassium shifts) and fluid resuscitation.

In **Methanol Intoxication**, there is typically a serum osmol gap (Remember, calculated serum osmolality is 2*Na + BUN/2.8 + Glucose/18) due to the presence of non-measured osmotic cations. There is additionally an anion-gap metabolic acidosis due to this. Treatment involves fomepizole.

In diarrhea, there is a loss of bicarbonate in the GI tract and potassium. There can be a non-anion gap metabolic acidosis due to this process, as well as hypernatremia due to the presence of a Free Water Deficit. Management involves correcting hypokalemia and fluid resuscitation.

Activity 2 - Liver Injury

You are on the night team getting sign-out when you get a call from the lab about a 16-year-old male recently admitted for suspected viral gastroenteritis in the setting of one day of abdominal pain, vomiting, and dehydration – you are notified of critical AST and ALT values of 2100 and 2200, respectively.

Do you agree with the admitting diagnosis? Why or why not?

- Viral Gastroenteritis is less likely in this case as it is not known to cause significant hepatocellular injury. Some common viruses, such as Adenovirus and Enterovirus, may cause liver injury.

The lab also tells you that the patient’s Basic Metabolic Panel is showing some abnormalities.

What does emesis do to electrolytes? Create a BMP with anticipated values.

- Emesis results in a loss of: Fluid, HCl, Na & K -> this can cause a hypokalemic, hypochloremia contraction alkalosis (pH may vary based on concurrent alkalosis/acidosis).

Which labs assess the following?

Synthetic liver function:

- Serum albumin, Serum Protein (hepatic injury can suppress synthesis of these hepatic-origin globulins).
- PT/INR (deficiency of vitamin K dependent factors 2,7,9,10 can occur in severe liver disease or fulminant hepatic failure, can also occur from decreased intake, or malabsorption which may occur in the setting of cholestasis – the later scenarios benefit from Vitamin K repletion).
- Ammonia (Created by gut bacteria and converted to urea in the liver. The inability to perform this process can cause increased levels).

-Hepatocellular injury:

- Aminotransferase levels (enzymes of hepatocellular origin, released in instances of direct parenchymal injury).
  1. ALT is liver specific.
  2. AST is derived from other organs besides the liver (muscle).

-Hepatobiliary injury / Cholestasis:

- Inflammation and obstruction of the biliary tract cause regurgitation of bile components into the serum causing elevated levels of: GGTP, direct and total bilirubin, alkaline phosphatase – elevation in these labs can be sensitive for hepatocellular disease or hepatic excretory dysfunction.

Which signs and symptoms may signify increasing severity of existing liver disease and why?

- Encephalopathy (rising ammonia concentrations combine with glutamate and via glutamine synthase produce glutamine which causes cerebral edema, intracranial hypertension, and neuronal dysfunction.
- Esophageal varices (hematemesis) and/or ascites (indicative of portal hypertension).
- Jaundice (cholestasis)

Which kind of liver injuries can cause 1000-fold rises in aminotransferases?

- Acute viral hepatitis, toxic injury, hypoxia, hypoperfusion, metabolic disease.

What are some illness scripts you might expect to hear from this patient with further questioning?

- Example 1: 16-year-old male patient with a history of depression, previous suicidal ideation, presenting with subacute onset of vomiting and RUQ abdominal pain after being well the day prior. (*Tylenol intoxication).*
- Example 2: 16-year-old male presenting with one day of abdominal pain & vomiting in the setting of multiple days of jaundice, fatigue, nausea, and flu-like symptoms. (*Hepatitis A).*

What are the different imaging modalities you could use in this case and how would they help you?

- Abdominal Film: May suggest hepatomegaly but is not sensitive. May see liver density changes or even air-filled bowel loops displaced if a liver mass is present. You can also see calcifications and gas collections within the liver, biliary tree, or portal circulation though rare.
- Ultrasound: Provides information about the size, composition and blood flow of the liver and the hepatobiliary tree. Dopplers can demonstrate portal vein patency and collateral circulation.
- CT scan: Similar to U/S but less suitable for under 2 years old (less intra-abdominal fat for contrast, smaller structures, need for sedation). Can be more accurate for mass identification
- MRI: Useful alternative to CT that avoids radiation. When used with cholangiography it is excellent for examining the hepatobiliary tree

Back to the patient: This patient is a 16-year-old male with a history of depression presenting with 36 hours of abdominal pain, vomiting, & jaundice who is found to be febrile to 101F and GCS of 13.

What is your differential diagnosis? *Intoxication, acute viral hepatitis, ascending cholangitis.*

What are your next steps in management and work-up? *Examples include assessment of mental status, Airway/Breathing/Circulation, empiric antibiotics, and fluid resuscitation.* *Would recommend evaluating liver synthetic function and hepatobiliary injury.*

How does N-acetylcysteine (NAC) work? NAC replenishes intracellular glutathione. It is commonly used in the treatment of acetaminophen toxicity, as glutathione is diminished in acetaminophen metabolism and is one of the etiologies for hepatocellular damage in this intoxication.

***Activity 3 - Blood Gas Analyses***

*For reference ranges on Venous Blood Gases (VBG):*

*pH: 7.32 – 7.42*

*pCO2: 41-51*

*pO2: 25-40*

*HCO3: 22-26*

**Scenario #1:** You are working in the emergency department and are told to go quickly evaluate a 5-yer-old male with asthma before waiting for vitals because nursing was concerned about his work of breathing. He is tachypneic to 42 breaths per minute with a heart rate of 150 bpm. He has diffuse wheezing and accessory muscle use. You decide to administer an hour of continuous albuterol.

- If obtained: what would the appropriate VBG results be for this patient (please fill in the blanks below):
  - ____/_____/_____/_____
  - Example: 7.47/35/45/22 (respiratory alkalosis due to tachypnea)

**Scenario #2: Another patient with known asthma had worsening respiratory distress while receiving continuous albuterol.** You hear a respiratory therapist mention that they should get intubation supplies ready as a precaution.

- How would this patient’s exam differ from the patient above?
  - Respiratory failure would often involve fatigue, lethargy, and altered mental status. Patients may exhibit less overt distress and have decreased respiratory rates with low air entry on auscultation.
  - Decreasing heart rate may signal an impending cardiac arrest.
- What changes would you expect in the VBG above (Place arrows next to each)
  - pH/ pCO2/ pO2/ HCO3
  - With respiratory failure, you would expect respiratory acidosis (decreased pH) + hypercapnia (increased CO2).
- Why are patients with asthma difficult to manage when intubated?
  - There are significant risks with intubating patients with asthma exacerbations due to increased air trapping. The necessity for high PEEP/PIP can lead to pneumothorax and decreased venous return, ultimately leading to decreased cardiac output (hypotension) and cardiac arrest.

**Scenario #3:** A 4-year-old with complex medical history including hypoxic-ischemic encephalopathy, tracheostomy and ventilator-dependence, g-tube dependence, dysautonomia, and seizures presents with a fever and feeding intolerance. Given her complex history a VBG was obtained and is shown below:

- **7.29/61/42/30**
  - Circle the acid-base derangement occurring in this patient:
    - Metabolic acidosis with respiratory compensation
    - Metabolic alkalosis with respiratory compensation
    - Respiratory Acidosis with metabolic compensation
    - Respiratory Alkalosis with metabolic compensation
  - Is this an acute or chronic process going on?
    - Respiratory acidosis with metabolic compensation
    - Chronic – There is metabolic compensation via increased synthesis/retention of bicarbonate. You would expect pH to decrease by 0.03 for every 10 mmHg rise in CO2. Metabolic compensation is often a chronic process (versus respiratory compensation which can be acute).
- How would this sample compare if it were an arterial blood gas (ABG)?
  - Higher O2, lower CO2, less acidosis.
  - ABG considered gold standard, however we often rely on VBGs due to easier to obtain
- What would a scenario be in which this patient would have acute CO2 retention? How would the VBG differ from her initial one before?
  - Pulmonary infectious process, ventilator malfunction, large mucous plug
  - Acute CO2 retention: pH decreases by 0.08 for every 10 mmHg increase in CO2

**Scenario #4:** You are on an overnight in the PICU and you get the following calls about patients who are receiving various forms of respiratory support:

- Mechanical ventilation (SIMV w/ Pressure control, PEEP 6, Rate 22, FiO2 50%)- latest ABG **7.20/67/42/24**
  - What changes, if any, would you make to their ventilation?
    - Would recommend increasing respiratory rate to increase ventilation due to hypercapnia
- Patient on mechanical ventilation (SIMV w/ pressure control, PEEP 6, Rate 20, FiO2 45%)- latest ABG showing **7.35/45/65/20**
  - What changes, if any, would you make to their ventilation based on this phone call?
    - Decrease PEEP to 5, could also consider weaning FiO2 40%
    - Limitations to using VBG to wean ventilation: PvO2 does not correlate as well with PAO2.
- A patient on the following settings: SIMV w/ pressure control, PEEP 5, Rate 22, FiO2 70%) with the latest VBG: **7.38/48/40/24**
  - What changes would you make to this patient’s ventilation?
    - Decrease FiO2
- An 8-month-old female s/p PICU stay due to prolonged bronchiolitis course requiring intubation. This patient was extubated last week to high-flow nasal cannula and gradually weaned to 1L O2 via nasal cannula. Weaned to room air overnight. VBG showed the following at time of transfer to the floor.
  - **7.49/38/40/32**
  - What process is going on here?
    - Respiratory acidosis
    - Respiratory alkalosis
    - Metabolic acidosis
    - Metabolic alkalosis
  - Metabolic alkalosis
  - What is a potential explanation for this process?
    - The patient may have been on diuretics in the PICU; diuretics can cause increased excretion of chloride relative to bicarbonate, therefore increased bicarbonate--> alkalosis (*contraction alkalosis).*

**Scenario #5:** Here is a VBG that you obtained on a patient admitted for 4 days with pneumonia and intermittently on nasal cannula:

**7.36/58/41/34**

- What process is going on here?
  - Respiratory acidosis, partially compensated
  - Respiratory acidosis, fully compensated
  - Respiratory alkalosis, partially compensated
  - Respiratory acidosis, fully compensated
- How can you determine if it is compensated or not?
  - Respiratory acidosis, fully compensated
  - Here we see metabolic compensation in the form of increased retention of bicarbonate.
- Winter’s formula: Predicted PACO2= 1.5(bicarb) +8 +/-2
