# Supplementary material for: A Pilot Longitudinal Clinical Reasoning Curriculum for Pediatric Residents
Source: MedEdPORTAL. 2024 Sep 25;20:11447. doi: 10.15766/mep_2374-8265.11447 (PMC11422513; doi:10.15766/mep_2374-8265.11447)
Supplement: Supplementary file 1 — Preimplementation Survey.docxCurriculum Goals, Objectives, and Timeline.docxSession 1 - Illness Scripts.pptxSession 1 - Small-Group Facilitator Guide.docxSession 2 - Illness Scripts 2.pptxSession 2 - Small-Group Facilitator Guide.docxSession 3 - Script Concordance.pptxSession 3 - Small-Group Facilitator Guide.docxSession 3 - Small-Group Handout.docxSession 4 - Pathophysiology.pptxSession 4 - Small-Group Facilitator Guide.docxSession 4 - Small-Group Handout.docxSession 5 - Review Game.pptxPostimplementation Survey.docx [file mep_2374-8265.11447-s001.zip › L. Session 4 - Small-Group Handout.docx]

**Clinical Reasoning Curriculum: Session #4 – Pathophysiology Small Group Worksheets**

Activity 1 – Acidosis

**Directions**: Evaluate the Basic Metabolic Panels below and match the panel with a suspected diagnosis shown on the right. After you match the BMPs, split up the 5 BMPs among your group and have each member explain electrolyte derangements and discuss any specific management steps that may resolve the abnormalities.

**Clinical Scenario:** 2-year-old previously healthy male presenting with altered mental status

**DIAGNOSES**:

1. ______ Addison’s Disease

131 110 20 Uremia



5.4 14 0.8 64 Diabetic Ketoacidosis

Serum Osmolality (Osm): 296 Methanol Intoxication Acute Diarrhea

2. _________

129 102 7



4.9 7 0.8 546

Serum Osm: 297

3. __________

153 125 29



2.7 16 0.8 81

Serum Osm: 318

4. __________

139 107 15



3.2 14 0.5 97

Serum Osm: 303

5. __________

131 100 74



6.4 14 3.7 120

Serum Osm: 295

**Activity 2 - Liver Injury**

You are on the night team getting sign-out when you get a call from the lab about a 16-year-old male recently admitted for suspected viral gastroenteritis in the setting of one day of abdominal pain, vomiting, and dehydration – you are notified of critical AST and ALT values of 2100 and 2200, respectively.

1. Do you agree with the admitting diagnosis? Why or why not?
2. The lab also tells you that the patient’s Basic Metabolic Panel is showing some abnormalities. What does emesis do to electrolytes? Create a BMP with anticipated values.
3. Which labs assess the following?
   1. Synthetic liver function:
   2. Hepatocellular injury:
   3. Hepatobiliary injury / Cholestasis:
4. Which signs and symptoms may signify increasing severity of existing liver disease and why?
5. Which kind of liver injuries can cause 1000-fold rises in aminotransferases?
6. What are some illness scripts you might expect to hear from this patient with further questioning?
7. What are the different imaging modalities you could use in this case and how would they help you?
8. ***Back to the patient:*** This patient is a 16-year-old male with a history of depression presenting with 36 hours of abdominal pain, vomiting, & jaundice who is found to be febrile to 101F and GCS of 13.
9. What is your differential diagnosis?
10. What are your next steps in management and work-up?
11. How does N-acetylcysteine (NAC) work and what is a common use for NAC?

**Activity 3 - Blood Gas Analyses**

*For reference ranges on Venous Blood Gases (VBG):*

*pH: 7.32 – 7.42*

*pCO2: 41-51*

*pO2: 25-40*

*HCO3: 22-26*

**Scenario #1:** You are working in the emergency department and are told to go quickly evaluate a 5-year-old male with asthma before waiting for vitals because nursing was concerned about his work of breathing. He is tachypneic to 42 breaths per minute with a heart rate of 150 bpm. He has diffuse wheezing and accessory muscle use. You decide to administer an hour of continuous albuterol.

- If obtained: what would the appropriate VBG results be for this patient (please fill in the blanks below):
  - ____/_____/_____/_____

**Scenario #2: Another patient with known asthma had worsening respiratory distress while receiving continuous albuterol.** You hear a respiratory therapist mention that they should get intubation supplies ready as a precaution.

- How would this patient’s exam differ from the patient above?
- What changes would you expect in the VBG above (Place arrows next to each)
  - pH/ pCO2/ pO2/ HCO3
- Why are patients with asthma difficult to manage when intubated?

**Scenario #3:** A 4-year-old with complex medical history including hypoxic-ischemic encephalopathy, tracheostomy and ventilator-dependence, g-tube dependence, dysautonomia, and seizures presents with a fever and feeding intolerance. Given her complex history a VBG was obtained and is shown below:

- **7.29/61/42/30**
  - Circle the acid-base derangement occurring in this patient:
    - Metabolic acidosis with respiratory compensation
    - Metabolic alkalosis with respiratory compensation
    - Respiratory acidosis with metabolic compensation
    - Respiratory alkalosis with metabolic compensation
  - Is this an acute or chronic process going on?
- How would this sample compare if it were an arterial blood gas (ABG)?
- What would a scenario be in which this patient would have acute CO2 retention? How would the VBG differ from her initial one before?

**Scenario #4:** You are on an overnight in the PICU and you get the following calls about patients who are receiving various forms of respiratory support:

- Mechanical ventilation (SIMV w/ Pressure control, PEEP 6, Rate 22, FiO2 50%)- latest ABG **7.20/67/42/24**
  - What changes, if any, would you make to their ventilation?
- Patient on mechanical ventilation (SIMV w/ pressure control, PEEP 6, Rate 20, FiO2 45%)- latest ABG showing **7.35/45/65/20**
  - What changes, if any, would you make to their ventilation based on this phone call?
- A patient on the following settings: SIMV w/ pressure control, PEEP 5, Rate 22, FiO2 70%) with the latest VBG: **7.38/48/40/24**
  - What changes would you make if any to this patient’s ventilation?
- An 8-month-old female who is post-PICU admission due to prolonged bronchiolitis course requiring intubation. This patient was extubated last week to high-flow nasal cannula and gradually weaned to 1L O2 via nasal cannula. Weaned to room air overnight. VBG showed the following at time of transfer to the floor.
  - **7.49/38/40/32**
  - What process is going on here?
    - Respiratory acidosis
    - Respiratory alkalosis
    - Metabolic acidosis
    - Metabolic alkalosis
  - What is a potential explanation for this process?

**Scenario #5:** Here is a VBG that you obtained on a patient admitted for 4 days with pneumonia and intermittently on nasal cannula:

**7.36/58/41/34**

- What process is going on here?
  - Respiratory acidosis, partially compensated
  - Respiratory acidosis, fully compensated
  - Respiratory alkalosis, partially compensated
  - Respiratory acidosis, fully compensated
- How can you determine if it is compensated or not?
