# Supplementary material for: A Pilot Longitudinal Clinical Reasoning Curriculum for Pediatric Residents
Source: MedEdPORTAL. 2024 Sep 25;20:11447. doi: 10.15766/mep_2374-8265.11447 (PMC11422513; doi:10.15766/mep_2374-8265.11447)
Supplement: Supplementary file 1 — Preimplementation Survey.docxCurriculum Goals, Objectives, and Timeline.docxSession 1 - Illness Scripts.pptxSession 1 - Small-Group Facilitator Guide.docxSession 2 - Illness Scripts 2.pptxSession 2 - Small-Group Facilitator Guide.docxSession 3 - Script Concordance.pptxSession 3 - Small-Group Facilitator Guide.docxSession 3 - Small-Group Handout.docxSession 4 - Pathophysiology.pptxSession 4 - Small-Group Facilitator Guide.docxSession 4 - Small-Group Handout.docxSession 5 - Review Game.pptxPostimplementation Survey.docx [file mep_2374-8265.11447-s001.zip › _Educational Summary Report_11447.pdf]

MedEdPORTAL® The AAMC Journal of Teaching and Learning Resources

Original Publication Open Access

A Pilot Longitudinal Clinical Reasoning Curriculum for Pediatric Residents

James Bowen, MD*, Catherine Polak, MD, Joanna Thomson, MD, MPH, Lisa Herrmann, MD, MEd

*Corresponding author: james.bowen@cchmc.org

Abstract

Introduction: Clinical reasoning (CR) is required for physicians. Pediatric residents often gain CR skills through experiential learning. Currently, deliberate education on CR targeted toward pediatric residents is inconsistent. Our objective was to implement a pilot CR curriculum, including five hour-long sessions, and evaluate its impact on self-identified CR Milestones and comfort with CR skills. **Methods:** We used Kern's six steps for curriculum development to develop our curriculum. Five morning report sessions included didactics and small-group activities. Pre/post surveys assessed resident self-identified level on ACGME Milestones related to CR skills (Patient Care 4 [PC4] and Medical Knowledge 2 [MK2]) and comfort with CR skills. The postsurvey assessed resident attitudes toward the sessions. Paired samples for Milestone and comfort-based questions were analyzed using Wilcoxon signed rank tests. Attitude questions were reported with descriptive statistics. **Results:** Each of the five curricular sessions was attended by 40-50 pediatric residents. Seventy-one trainees (58% of residency) and 51 trainees (42% of residency) completed the pre- and postsurveys, respectively, with 20 paired samples. Self-assessment of PC4 ($p = .006$) and resident comfort with all measured CR skills increased significantly. Of trainees who attended at least one session ($n = 44$), most reported finding the sessions helpful (97%), relevant to their clinical work (97%), and impactful on their clinical practice (73%). **Discussion:** Following exposure to this CR curriculum, pediatric residents reported increased self-identified competency levels on the evaluated Milestones and improved comfort with CR skills. Dedicated CR education may advance pediatric resident understanding of and comfort with CR.

Keywords:
Illness Script, Residency, Script Concordance, Clinical Reasoning/Diagnostic Reasoning, Games, Pediatrics

Educational Objectives

By the end of this pilot, learners will be able to:

1. Identify common clinical reasoning skills (e.g., using illness scripts, script concordance, and pathophysiology in clinical decision-making) in educational conferences and simulated encounters.
2. Apply common clinical reasoning skills (e.g., using illness scripts, script concordance, and pathophysiology in clinical decision-making) in educational conferences.
3. Demonstrate clinical reasoning skills (e.g., using illness scripts, script concordance, and pathophysiology in clinical decision-making) in various simulated clinical environments.
4. Appraise clinical reasoning used in everyday clinical environments and in educational conferences.

Citation:
Bowen J, Polak C, Thomson J, Herrmann L. A pilot longitudinal clinical reasoning curriculum for pediatric residents. *MedEdPORTAL*. 2024;20:11447. https://doi.org/10.15766/mep_2374-8265.11447

Introduction

The term *clinical reasoning* (CR) encompasses a variety of skills essential to expert clinicians. Broadly, CR is defined as the ability to integrate different types of information presented by a patient, weigh evidence, reflect upon the diagnostic process, and arrive at a leading hypothesis and management plan.[1-3] CR skills are paramount to the diagnostic process and have been shown to decrease diagnostic errors and allow physicians to more quickly reach diagnostic goals.[2] While CR skills are frequently defined and taught early in a clinician's medical education (i.e., medical school), they are inconsistently reinforced in graduate medical education (GME). CR skills represent a commonly cited deficiency for resident trainees and have been shown to take more faculty time to remediate.[4] Given the importance of CR skills, the Accreditation Council for Graduate Medical Education (ACGME) and American Board of Pediatrics have incorporated components of CR into the Pediatric Milestones, the Program Requirements for Pediatric Residencies, and entrustable professional activities.[5-7] However, there is a paucity of published curricula on CR skills for pediatric resident physicians, and it is unclear how these skills are emphasized during residency.

Copyright © 2024 Bowen et al. This is an open-access publication distributed under the terms of the Creative Commons Attribution license.

Current published CR curricula and assessment models exist for medical students and in GME; however, tools for GME have largely been implemented and evaluated in adult medical specialties (e.g., internal medicine).[8-13] While previous research has illustrated the positive impact of deliberate CR education for internal medicine trainees, this has not been extensively studied in pediatrics.[14] CR is essential to pediatric resident development and clinical practice, but CR assessment and education may be impeded by aspects of pediatric GME. For example, family-centered rounding is ubiquitous in pediatrics and, although well documented to benefit patient care, may cloud CR assessment and pose challenges to open discussion of CR on rounds.[15,16] Additionally, clinical pathways are common in pediatric institutions; while they promote standardized care, they reduce resident autonomy and opportunity to practice CR.[17,18]

Incorporation of CR training into residency can be challenging due to limited available time for learners, a paucity of experts available to teach the skills, and a nonuniform understanding of the importance of CR for pediatric residents.[19] We sought to address this gap by developing a curriculum to introduce CR to pediatric residents, with topics including illness scripts, script concordance, and pathophysiology. While this curriculum does not comprehensively include all aspects of CR, it provides a base framework and can serve to supplement existing CR education or as an adaptable tool to emphasize CR.

The content of the curriculum was informed by a local preimplementation needs assessment, trainee deficiencies observed by residency program leadership and attending physicians, and existing literature regarding teaching CR.[1,20,21] Program leadership noted downward trends in the ACGME Pediatric Patient Care Milestone 4 (PC4) and Medical Knowledge Milestone 2 (MK2) and in resident in-training exam scores across academic years. While the root cause of these trends was likely multifactorial, leadership felt that deficits in CR contributed to these changes and could be intervened upon. Curriculum content was designed according to the automaticity and skill expertise conceptual framework, which posits that learning involves a cognitive phase (including defining skills); an associative phase, involving the incorporation of the defined knowledge into everyday experiences; and an autonomous phase, where the learner can utilize the emphasized knowledge independently and retrieve the knowledge more quickly.[22] Each curricular session included reflective practice to encourage reflection on action[23] (i.e., the skills practiced in each session) in order to promote incorporation of skills in everyday clinical practice. Finally, content was mapped to PC4 and MK2, as well as to entrustable professional activities 4 and 11, which include CR competencies.[7,24,25]

Our curriculum is meant to provide a foundation for pediatric CR education. It may supplement existing education in a program or introduce CR skills. The ACGME requires protected educational time for trainees, and programs often incorporate 6-10 hours of protected educational time per week[25]; the curriculum is adaptable to be easily integrated into existing conference structures for individual programs. The curriculum is most suitable for medical students, interns, resident physicians, and advanced practice providers. Although the curriculum has been developed specifically for pediatrics, the concepts are relevant to all medical trainees and could be adapted to other specialties. Participants should have a basic understanding of pathophysiology and knowledge of differential diagnosis building, but do not require experience in CR prior to participation in the curriculum. The curriculum can be facilitated by faculty, chief residents, or senior-level residents who have a basic understanding of the emphasized CR domains.

Methods

Setting

We implemented this curriculum through the pediatric residency program at our large tertiary-care academic children's hospital. The curriculum was presented during hour-long sessions typically reserved for case-based patient presentations. Morning report sessions were protected time for residents on both inpatient and outpatient rotations. While attendance was encouraged, it was not required. The curriculum consisted of five hour-long sessions, which we presented via a hybrid structure, in person and via teleconferencing. The hybrid model was chosen given attendance restrictions due to COVID-19; if space allows, purely in-person conferences are ideal for this curriculum to optimize interactive portions of each session. The curriculum required a space for a medium-to-large group (our sessions included 40-50 trainees and three to five facilitators per session, though they could be employed with fewer learners), a computer with Microsoft PowerPoint (and a teleconferencing service such as Microsoft Teams for hybrid virtual/in-person sessions if desired), a projector, and screen. Because each session involved small-group learning, the ideal arrangement of seats was in an open layout with six to 10 chairs grouped together.

We presented the curriculum over the course of 6 months via spaced learning to promote knowledge retention.[26] The timeline can be modified based on the needs of the program or learners, but we would recommend at least 3 weeks between curricular sessions to encourage reflection, allow practice of

Copyright © 2024 Bowen et al. This is an open-access publication distributed under the terms of the Creative Commons Attribution license.

the emphasized skills in clinical settings, and promote spaced learning.

Curriculum participants included all levels (PGY 1-PGY 5 of pediatric, internal medicine-pediatric, and triple-board [i.e., adult psychiatry, child psychiatry, and pediatrics] residents; $N = 118$), in addition to senior-level medical students (MS 3 and MS 4) and rotating residents (family medicine and psychiatry). While multiple types of learners participated in the curriculum, pediatric residents were the primary target audience, and thus, evaluation focused on this group.

Curricular Design

We used Kern's six steps for curriculum development to inform our curriculum design.[27] Chief residents and program directors performed a targeted needs assessment through informal interviews with approximately 20 trainees and direct observations from existing case-based educational conferences (e.g., senior resident physicians showing difficulty with hypothesis-driven history-taking or information synthesis). The identified skill gaps most closely aligned with the ACGME PC4 and MK2 Milestones. Institutional knowledge of topics covered in other areas and a preimplementation survey (Appendix A), which included questions to assess the perceived needs of residents, informed the global curriculum goals and objectives (Appendix B). Pediatric chief residents designed the curriculum, and pediatric program directors reviewed the session materials for accuracy and clarity. Our curriculum goals were to teach residents common CR concepts and increase their comfort with the CR skills included in their everyday clinical practice. The objectives of each session were to familiarize learners with common CR concepts, allow learners to identify gaps in their own CR, practice the skills in a variety of simulated clinical environments to increase comfort in the associated skills, and identify opportunities for incorporation of CR in clinical practice. Activities were also designed to allow for practice of skills that would map to ACGME Milestones pertaining to CR (Appendix B).[7,24,25] The content domains were informed by the curricular needs assessment and a literature review of CR skill education in other areas, such as in undergraduate medical education,[1,3,9,20,21] and were based on needs of the pediatric residents observed by residency leadership. Additional content included case scenarios commonly seen in pediatrics to encourage transfer of learning between the curriculum and real patient encounters. Appendix B summarizes the flow of each session.

Curriculum Implementation

The chief residents who designed the curriculum presented the materials and acted as small-group facilitators. Curriculum topics included CR as a concept and competency in addition to specific CR skills. The first four sessions began with 20 minutes of didactics followed by small-group activities. The first three sessions focused on illness scripts: Session 1 began with a didactic (Appendix C) that included the definition of illness scripts, provided examples of script usage, and offered advantages and disadvantages of using them in clinical practice, followed by a small-group activity (Appendix D), which allowed participants to practice hypothesis-driven information gathering by using their existing illness scripts. The didactic portion of session 2 (Appendix E) focused on building illness scripts and distinguishing features, after which small-group activities (Appendix F) allowed participants to practice building and comparing their own novel scripts. Session 3 also began with a didactic (Appendix G) centered on script concordance. Small-group activities included in session 3 (Appendices H and I) allowed for practice of script concordance in common clinical presentations.

Session 4 pivoted to the use of pathophysiology in clinical practice, beginning with a didactic (Appendix J) followed by cases (Appendices K and L) that allowed participants to work through diagnostic and management reasoning while focusing on biomedical concepts. Finally, session 5 (Appendix M) was designed as a quiz-style game to further diversify the teaching methods used in the curriculum via gamification of the content discussed in the preceding four sessions.[26] Additionally, this session began with a reflective portion[23] to summarize participants' key takeaways from the curriculum. Appendices include all presented content, are delineated by session, and should be used for associated activities. PowerPoint files include didactic materials, and document files feature facilitator guides/answer keys and handouts for small groups with description of activities.

Presenter Mode for PowerPoint was used, as notes were included for facilitators through the content. Facilitator appendices included suggested timing of the didactics and small-group activities as well as small-group instructions for both learners and facilitators.

Sessions were designed to include didactics to provide background material, define relevant CR terms and skills, and outline the session activities. We devoted 30 minutes to small-group activities meant to reinforce the skills and allow for discussion on how to incorporate the emphasized skills into daily clinical practice. Five to 10 minutes were allotted for small-group activities and discussions at the end of each session.

Following each session, the PowerPoint slide deck was uploaded to the pediatric residency shared drive on Microsoft Teams for

Copyright © 2024 Bowen et al. This is an open-access publication distributed under the terms of the Creative Commons Attribution license.

access by participants, as well as trainees unable to attend the session.

Curricular Evaluation

A preimplementation survey (Appendix A) included questions that guided the curriculum's development and served as a baseline for resident comfort with CR skills. Survey questions were informed by a literature review on CR teaching methods[1,2,9,13] and perceived gaps in the current residency curriculum. The pre/post surveys were reviewed by three chief residents and two experts in CR, including a pediatric hospital medicine attending physician and a pediatric residency program director, to ensure content validity. Construct validity was enhanced via cognitive interviewing with three participants outside of our sample. The surveys included participant self-identification of perceived level of competency on ACGME Milestones related to CR, as well as understanding of and comfort with various CR domains scored on a 5-point Likert scale (1 = *extremely uncomfortable,* 5 = *extremely comfortable*). The postcurriculum survey (Appendix N) included identical ACGME Milestone and comfort-based questions, as well as questions surveying attitudes toward the curriculum and barriers to attendance. The surveys were voluntary and were electronically disseminated by chief residents in September 2021 (prior to the curriculum's initiation) and in May 2022 (postsurvey). Participants were assigned deidentified study IDs to anonymize responses and enable matching of pre/post surveys.

We delivered the curriculum from October 2021 to April 2022. In addition to questions regarding feedback included in the postevaluation survey, chief residents solicited informal feedback at the end of each session through open discussion to assess applicability of the session.

Data Analysis

Matched survey data on Milestone self-assessment and comfort with CR skills were compared using Wilcoxon signed rank tests. Questions regarding attitude towards sessions were dichotomized—strongly agree and agree versus neither agree/disagree, disagree, and strongly disagree—and reported via descriptive statistics. Statistical analyses were performed using Statistical Analysis Software version 9.4.

Results

Each session was attended by 40-50 residents in addition to five to 10 medical students. Seventy-one trainees (58% of total residency) completed the preimplementation survey, and 51 (42% of total residency) completed the postcurriculum survey. Twenty paired samples (eight PGY 1s, seven PGY 2s, and five PGY 3s; 17% of total residency) were evaluated. All 20 of these participants noted attending at least three of the curriculum sessions.

Self-assessment of the ACGME Milestone PC4 competency level (Figure 1) increased significantly ($p = .006$); self-assessment of Milestone MK2 (Figure 2) did not show statistical significance ($p = .14$) following curriculum implementation. Comfort with using the CR skills addressed in the curriculum demonstrated significant improvement after curriculum participation (Table), including defining ($p = .008$) and using illness scripts ($p = .002$), anticipating abnormal results ($p = .002$), employing pathophysiology ($p = .02$), and modifying differential diagnoses based on historical ($p = .001$), physical exam ($p = .002$), and laboratory findings ($p = .006$). Most trainees who attended at least one session ($n = 44$) reported finding the sessions helpful ($n = 43$, 97%), relevant to their clinical work ($n = 43$, 97%), and impactful on their clinical practice ($n = 32$, 73%).

Qualitative feedback for the sessions was generally positive, with learners praising the unique format of the case-based activities and noting that the small-group activities were "fun" and that they typically learned new concepts during each session. Formative feedback included "[recommending the sessions] as part of our residency orientation" and that "some cases were more basic than I expected."

Most respondents endorsed conflicting clinical responsibilities as the primary barrier to attending sessions ($n = 39$, 76%). Five respondents (10%) felt there were no barriers to attendance, while a limited number felt the curriculum was not relevant to their work ($n = 1$, 2%) or did not know the curriculum was being offered ($n = 1$, 2%). Qualitative feedback on barriers to attendance included being off service, rotating in other fields (e.g., internal medicine or psychiatry), or being on parental leave.

Discussion

We developed a CR curriculum to supplement existing education in a large pediatric residency program and to address perceived gaps in knowledge of and comfort with CR observed by residency leadership. Preimplementation data illustrated a lack of knowledge and comfort using CR; this suggests that the experiential learning method of attaining CR proficiency may be insufficient in instilling perceived comfort in trainee CR skills, especially in settings where patient census can vary considerably (e.g., in the setting of COVID-19 and lower pediatric patient admissions or general seasonality of common pediatric

Copyright © 2024 Bowen et al. This is an open-access publication distributed under the terms of the Creative Commons Attribution license.

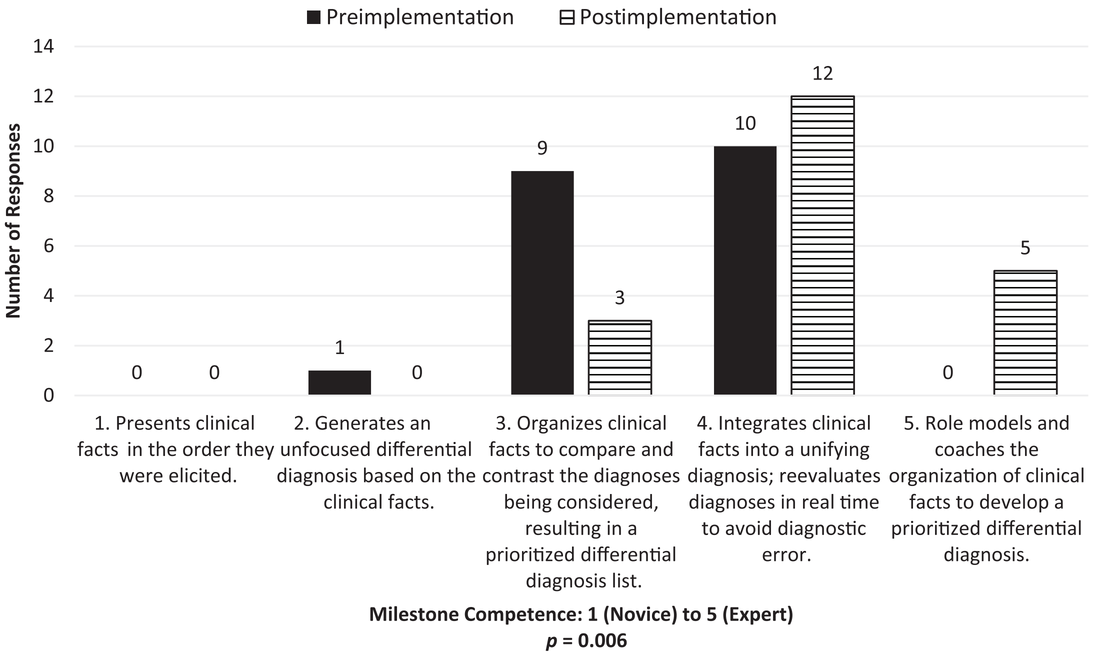

Figure 1. Pre- and postimplementation survey results for trainee self-identification of ACGME Patient Care Milestone 4 competence levels.

diagnoses). Curriculum goals, objectives, and content were designed to advance trainee knowledge of and comfort with common CR skills. This curriculum is unique in that incorporated skills have been chosen specifically for pediatric residents, with associated cases being drawn from common pediatric diagnoses and best practices (e.g., clinical pathways). Overall, the implementation of the curriculum was successful in that all the sessions were held as planned and each adhered to the

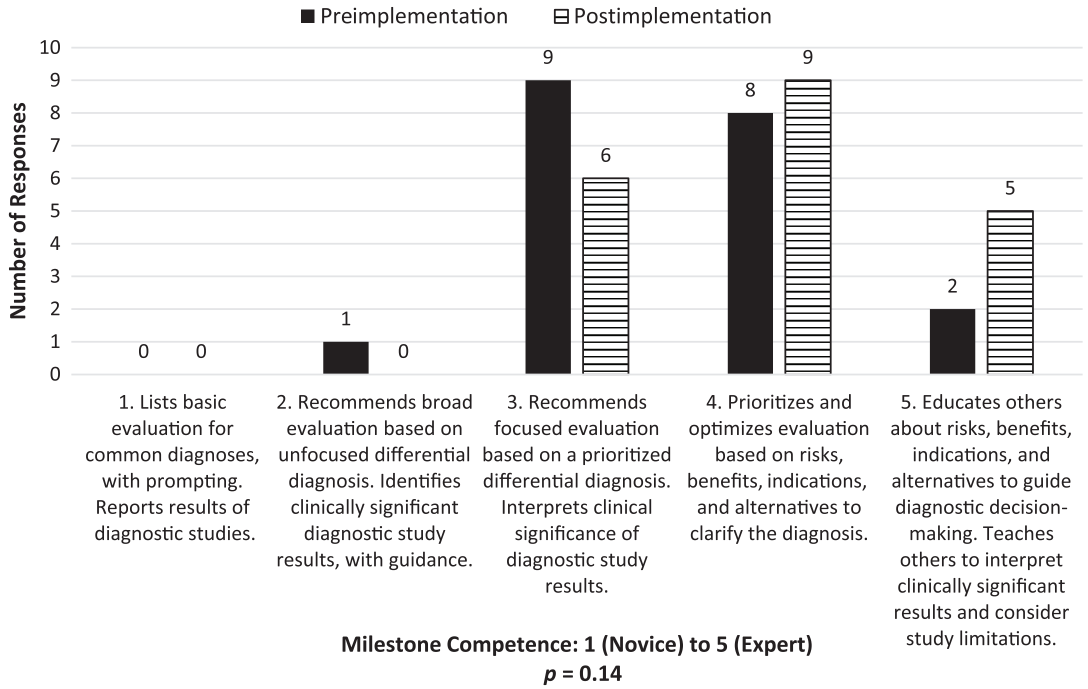

Figure 2. Pre- and postimplementation survey results for trainee self-identification of Medical Knowledge 2 competence levels.

Copyright © 2024 Bowen et al. This is an open-access publication distributed under the terms of the Creative Commons Attribution license.

Table. Pre- and Postimplementation Survey Results for Clinical Reasoning Skill and Comfort-Based Questions

Item[a]	No. (%)		
	Preimplementation	Postimplementation	*p*
Can you define "illness script"?[b]	12 (60)	20 (100)	.008
How often do you employ illness scripts in your clinical practice?[c]	2 (10)	9 (45)	.002
Comfort anticipating abnormal results in diagnostic evaluations based upon your differential diagnosis[d]	4 (20)	13 (65)	.002
Comfort modifying differential diagnosis to guide further workup based upon new historical information[d]	0 (0)	10 (50)	.001
Comfort modifying your differential diagnosis to guide further workup based upon physical examination findings[d]	2 (10)	2 (10)	.002
Comfort modifying your differential diagnosis to guide further workup based upon new laboratory information[d]	2 (10)	11 (55)	.006
Comfort employing organ system pathophysiology concepts to guide diagnostic reasoning[d]	1 (5)	6 (30)	.02

[a]Top responses for each survey item are reported. Matched samples ($N = 20$, 17% of total residency) included responses from eight PGY 1s, seven PGY 2s, and five PGY 3s.
[b]Top response: Yes.
[c]Top response: Almost every day.
[d]Top response: Extremely comfortable.

designed structure and timing. Furthermore, resident comfort with various CR domains increased significantly following exposure to the curriculum.

A strength of our curriculum is the variety of learning methods employed, including didactics, interactive activities, and a review game. Additionally, the curriculum is adaptable to other medical specialties; both the content and the timing of dissemination can be modified. Having a mixed audience of learners promoted overall learning and allowed senior residents to observe differences in CR among other learners, providing opportunities to assess CR skills in simulated scenarios. While the mixed audience was chosen for feasibility of curriculum implementation, the curriculum could be disseminated to audiences divided by level of training.

Challenges around curriculum dissemination mostly centered on attendance. Because CR is so paramount, we hoped for >75% attendance by the residents. In reflection, this goal was not realistically attainable given residents rotating at other sites, on night shifts, or on parental leave. This challenge is common for curricula that rely on GME morning or noon conferences and could be mitigated by including the curriculum in other settings, such as resident orientation. While spaced learning allowed time for participant implementation of skills, disseminating the full curriculum over a shorter period (e.g., 1-2 weeks) might improve the number of residents who attend all five sessions.

Our curriculum evaluation was not without limitations. Impact of the curriculum on learners outside of the pediatric residency was unclear as they were not included in our evaluation; these learners rotated at our site for only 4 weeks and thus would have attended only one session at most. Formal curricular evaluation was limited to lower levels (i.e., reactions, learning) of Kirkpatrick's model of evaluation.[28] This was partly due to the lack of standardized methods for evaluating pediatric resident CR in residency programs. This is an established barrier to evaluating CR curricula at the GME level, as it remains challenging to assess CR competence and behavior.[19] While facilitators observed participants demonstrating CR skills in simulated activities,[29] illustrating potential behavioral change (level three of Kirkpatrick's model), we did not perform direct observations of residents' behaviors in clinical settings. Additional limitations include lower response rates for paired survey evaluation, which left results susceptible to response bias. The postsurvey was voluntary and disseminated after the curriculum; we hypothesize that the response rate would increase by soliciting responses while participants are still present after each session. Time and clinical experience between evaluations may act as confounders and independently increase trainee comfort. This pilot curriculum was evaluated at one pediatric center, so results may not be generalizable to other institutions. Future evaluation could include expansion of participant demographics, including their previous experiences in CR, as this may impact engagement with the curriculum and allow instructors to modify their teaching methods or content.

We believe this curriculum can provide an excellent base, or supplementation, for CR education for pediatric residency programs of a variety of sizes and can be adapted to meet individual program needs. While we did not directly assess CR competence due to lack of available methods, the positive shift in self-identified competency level for ACGME Milestones may reflect a behavioral change in residents because of increased comfort in their skills. Increased comfort with CR may allow translation of these skills to test taking (e.g., in-training examinations or the General Pediatrics Certifying Examination). In the Annual Program Evaluation at the end of the academic year, program directors noted that initial skill deficits exhibited in other

Copyright © 2024 Bowen et al. This is an open-access publication distributed under the terms of the Creative Commons Attribution license.

case-based conferences improved following the curriculum's implementation, felt residents were more apt to openly discuss their CR process, and planned to continue this curriculum on an annual basis.

Our results suggest that dedicated CR education may advance perceived competency of and comfort with CR skills beyond what is gained with experiential learning alone. Curricular activities to promote CR skills taught in medical school but not emphasized in this curriculum may be incorporated into this curriculum based on the needs of individual settings. Additionally, identification and standardization of methods to assess CR competency will be paramount to allow for more robust evaluation of curricular work surrounding CR.

Appendices

A. Preimplementation Survey.docx
B. Curriculum Goals, Objectives, and Timeline.docx
C. Session 1 - Illness Scripts.pptx
D. Session 1 - Small-Group Facilitator Guide.docx
E. Session 2 - Illness Scripts 2.pptx
F. Session 2 - Small-Group Facilitator Guide.docx
G. Session 3 - Script Concordance.pptx
H. Session 3 - Small-Group Facilitator Guide.docx
I. Session 3 - Small-Group Handout.docx
J. Session 4 - Pathophysiology.pptx
K. Session 4 - Small-Group Facilitator Guide.docx
L. Session 4 - Small-Group Handout.docx
M. Session 5 - Review Game.pptx
N. Postimplementation Survey.docx

All appendices are peer reviewed as integral parts of the Original Publication.

James Bowen, MD: Clinical Fellow, Department of Pediatric Hospital Medicine, Cincinnati Children's Hospital Medical Center; ORCID: https://orcid.org/0000-0002-8199-9076

Catherine Polak, MD: Assistant Professor of Pediatrics, Department of Pediatric Hospital Medicine, UPMC Children's Hospital of Pittsburgh

Joanna Thomson, MD, MPH: Associate Professor of Pediatrics, Department of Pediatrics, University of Cincinnati College of Medicine; Attending Physician, Division of Hospital Medicine, Cincinnati Children's Hospital Medical Center

Lisa Herrmann, MD, MEd: Associate Professor of Pediatrics, Department of Pediatrics, University of Cincinnati College of Medicine; Attending Physician, Division of Hospital Medicine, Cincinnati Children's Hospital Medical Center

Disclosures
None to report.

Funding/Support
None to report.

Prior Presentations
Bowen J, Mack M, Marshall M, White J, Polak C, Thomson J. Evaluating a longitudinal clinical reasoning curriculum for pediatric residents. Presented at: Association for Pediatric Program Directors Annual Spring Meeting; March 28-31, 2023; Atlanta, GA.

Bowen J, Mack M, Marshall M, White J, Polak C, Thomson J. Evaluating a longitudinal clinical reasoning curriculum for pediatric residents. Presented at: Pediatric Academic Societies Meeting; April 27-May 1, 2023; Washington, DC.

Bowen J, Mack M, Marshall M, White J, Polak C, Thomson J. Evaluating a longitudinal clinical reasoning curriculum for pediatric residents. Presented at: Pediatric Hospital Medicine Conference; August 3-6, 2023; Philadelphia, PA.

Ethical Approval
Reported as not applicable.

References

1. Bowen JL. Educational strategies to promote clinical diagnostic reasoning. *N Engl J Med*. 2006;355(21):2217-2225. https://doi.org/10.1056/NEJMra054782
2. Cutrer WB, Sullivan WM, Fleming AE. Educational strategies for improving clinical reasoning. *Curr Probl Pediatr Adolesc Health Care*. 2013;43(9):248-257. https://doi.org/10.1016/j.cppeds.2013.07.005
3. Modi JN, Anshu Gupta P, Singh T. Teaching and assessing clinical reasoning skills. *Indian Pediatr*. 2015;52(9):787-794. https://doi.org/10.1007/s13312-015-0718-7
4. Guerrasio J, Garrity MJ, Aagaard EM. Learner deficits and academic outcomes of medical students, residents, fellows, and attending physicians referred to a remediation program, 2006–2012. *Acad Med*. 2014;89(2):352-358. https://doi.org/10.1097/ACM.0000000000000122
5. Pediatrics: Milestones. Accreditation Council for Graduate Medical Education. Accessed September 9, 2024. https://www.acgme.org/specialties/pediatrics/milestones/
6. Schumacher DJ, West DC, Schwartz A, et al; Association of Pediatric Program Directors Longitudinal Educational Assessment Research Network General Pediatrics Entrustable Professional

Copyright © 2024 Bowen et al. This is an open-access publication distributed under the terms of the Creative Commons Attribution license.

Activities Study Group. Longitudinal assessment of resident performance using entrustable professional activities. *JAMA Netw Open*. 2020;3(1):e1919316. https://doi.org/10.1001/jamanetworkopen.2019.19316

7. Entrustable professional activities for general pediatrics. American Board of Pediatrics. Updated May 29, 2024. Accessed September 9, 2024. https://www.abp.org/content/entrustable-professional-activities-general-pediatrics
8. Levin M, Cennimo D, Chen S, Lamba S. Teaching clinical reasoning to medical students: a case-based illness script worksheet approach. *MedEdPORTAL*. 2016;12:10445. https://doi.org/10.15766/mep_2374-8265.10445
9. Bhardwaj P, Black EW, Fantone JC III, Lopez M, Kelly M. Script Concordance Tests for formative clinical reasoning and problem-solving assessment in general pediatrics. *MedEdPORTAL*. 2022;18:11274. https://doi.org/10.15766/mep_2374-8265.11274
10. Duca NS, Glod S. Bridging the gap between the classroom and the clerkship: a clinical reasoning curriculum for third-year medical students. *MedEdPORTAL*. 2019;15:10800. https://doi.org/10.15766/mep_2374-8265.10800
11. Weinstein A, Pinto-Powell R. Introductory clinical reasoning curriculum. *MedEdPORTAL*. 2016;12:10370. https://doi.org/10.15766/mep_2374-8265.10370
12. Schaye V, Miller L, Kudlowitz D, et al. Development of a clinical reasoning documentation assessment tool for resident and fellow admission notes: a shared mental model for feedback. *J Gen Intern Med*. 2022;37(3):507-512. https://doi.org/10.1007/s11606-021-06805-6
13. Daniel M, Rencic J, Durning SJ, et al. Clinical reasoning assessment methods: a scoping review and practical guidance. *Acad Med*. 2019;94(6):902-912. https://doi.org/10.1097/ACM.0000000000002618
14. Schaye V, Eliasz KL, Janjigian M, Stern DT. Theory-guided teaching: implementation of a clinical reasoning curriculum in residents. *Med Teach*. 2019;41(10):1192-1199. https://doi.org/10.1080/0142159X.2019.1626977
15. Pinto JM, Chu D, Petrova A. Pediatric residents' perceptions of family-centered rounds as part of postgraduate training. *Clin Pediatr (Phila)*. 2014;53(1):66-70. https://doi.org/10.1177/0009922813501377
16. Calardo SJ, Tomlinson L, Bhatia D, Weis A, Port C. Optimizing resident education during family-centered rounds: an educational improvement initiative. *Pediatr Qual Saf*. 2022;7(6):e614. https://doi.org/10.1097/pq9.0000000000000614
17. Congdon M, Clancy CB, Balmer DF, et al. Diagnostic reasoning of resident physicians in the age of clinical pathways. *J Grad Med Educ*. 2022;14(4):466-474. https://doi.org/10.4300/JGME-D-21-01032.1
18. Fatemi Y, Costello A, Lieberman L, et al. Clinical pathways and diagnostic reasoning: a qualitative study of pediatric residents' and hospitalists' perceptions. *J Hosp Med*. 2023;18(2):139-146. https://doi.org/10.1002/jhm.13010
19. Sudacka M, Adler M, Durning SJ, et al. Why is it so difficult to implement a longitudinal clinical reasoning curriculum? A multicenter interview study on the barriers perceived by European health professions educators. *BMC Med Educ*. 2021;21:575. https://doi.org/10.1186/s12909-021-02960-w
20. Woods NN. Science is fundamental: the role of biomedical knowledge in clinical reasoning. *Med Educ*. 2007;41(12):1173-1177. https://doi.org/10.1111/j.1365-2923.2007.02911.x
21. Charlin B, Boshuizen HPA, Custers EJ, Feltovich PJ. Scripts and clinical reasoning. *Med Educ*. 2007;41(12):1178-1184. https://doi.org/10.1111/j.1365-2923.2007.02924.x
22. Zackoff MW, Real FJ, Abramson EL, Li STT, Klein MD, Gusic ME. Enhancing educational scholarship through conceptual frameworks: a challenge and roadmap for medical educators. *Acad Pediatr*. 2019;19(2):135-141. https://doi.org/10.1016/j.acap.2018.08.003
23. Husebø SE, O'Regan S, Nestel D. Reflective practice and its role in simulation. *Clin Simul Nurs*. 2015;11(8):368-375. https://doi.org/10.1016/j.ecns.2015.04.005
24. *Entrustable Professional Activities: EPA 11 for General Pediatrics*. American Board of Pediatrics; 2021. Accessed September 9, 2024. https://www.abp.org/sites/abp/files/pdf/gen_peds_epa_11.pdf
25. Pediatrics: program requirements, FAQs, and applications. Accreditation Council for Graduate Medical Education. Accessed September 9, 2024. https://www.acgme.org/specialties/pediatrics/program-requirements-and-faqs-and-applications/
26. van Gaalen AEJ, Brouwer J, Schönrock-Adema J, Bouwkamp-Timmer T, Jaarsma ADC, Georgiadis JR. Gamification of health professions education: a systematic review. *Adv Health Sci Educ Theory Pract*. 2021;26(2):683-711. https://doi.org/10.1007/s10459-020-10000-3
27. Thomas PA, Kern DE, Hughes MT, Chen BY, eds. *Curriculum Development for Medical Education: A Six-Step Approach.* 3rd ed. Johns Hopkins University Press; 2016.
28. Smidt A, Balandin S, Sigafoos J, Reed VA. The Kirkpatrick model: a useful tool for evaluating training outcomes. *J Intellect Dev Disabil*. 2009;34(3):266-274. https://doi.org/10.1080/13668250903093125
29. Patocka C, Pandya A, Brennan E, et al. The impact of just-in-time simulation training for healthcare professionals on learning and performance outcomes: a systematic review. *Simul Healthc*. 2024;19(1S):S32-S40. https://doi.org/10.1097/SIH.0000000000000764

Received: March 8, 2024
Accepted: May 9, 2024
Published: September 25, 2024

Copyright © 2024 Bowen et al. This is an open-access publication distributed under the terms of the Creative Commons Attribution license.
